# Clinical Heterogeneity Among *LRRK2* Variants in Parkinson's Disease: A Meta-Analysis

**DOI:** 10.3389/fnagi.2018.00283

**Published:** 2018-09-19

**Authors:** Li Shu, Yuan Zhang, Hongxu Pan, Qian Xu, Jifeng Guo, Beisha Tang, Qiying Sun

**Affiliations:** ^1^Department of Neurology, Xiangya Hospital, Central South University, Changsha, China; ^2^National Clinical Research Center for Geriatric Disorders, Changsha, China; ^3^Key Laboratory of Hunan Province in Neurodegenerative Disorders, Central South University, Changsha, China; ^4^Parkinson's Disease Center of Beijing Institute for Brain Disorders, Beijing, China; ^5^Collaborative Innovation Center for Brain Science, Shanghai, China; ^6^Collaborative Innovation Center for Genetics and Development, Shanghai, China; ^7^Department of Geriatrics, Xiangya Hospital, Central South University, Changsha, China; ^8^Center for Medical Genetics, School of Life Sciences, Central South University, Changsha, China

**Keywords:** Parkinson's disease, *LRRK2*, phenotype, clinical, meta-analysis

## Abstract

**Background:** Parkinson's disease (PD) is one of the most common neurodegenerative diseases. Variants in the *LRRK2* gene have been shown to be associated with PD. However, the clinical characteristics of *LRRK2*-related PD are heterogeneous. In our study, we performed a comprehensive pooled analysis of the association between specific *LRRK2* variants and clinical features of PD.

**Methods:** Articles from the Medline, Embase, and Cochrane databases were included in the meta-analysis. Strict inclusion criteria were applied, and detailed information was extracted from the final original articles included. Revman 5.3 software was used for publication biases and pooled and sensitivity analyses.

**Results:** In all, 66 studies having the clinical manifestations of PD patients with G2019S, G2385R, R1628P, and R1441G were included for the final analysis. The prominent clinical features of *LRRK2-*G2019S-related PD patients were female sex, higher rates of early-onset PD (EOPD), and family history (OR: 0.77 [male], 1.37, 2.62; *p* < 0.00001, 0.02, < 0.00001). PD patients with G2019S were more likely to have high scores of Schwab & England (MD: 1.49; *p* < 0.00001), low GDS scores, high UPSIT scores (MD: 0.43, 4.70; *p* = 0.01, < 0.00001), and good response to L-dopa (OR: 2.33; *p* < 0.0001). Further, G2019S carriers had higher LEDD (MD: 115.20; *p* < 0.00001) and were more likely to develop motor complications, such as dyskinesia and motor fluctuations (OR: 2.18, 2.02; *p* < 0.00001, 0.04) than non-carriers. G2385R carriers were more likely to have family history (OR: 2.10; *p* = 0.007) than non-G2385R carriers and lower H-Y and higher MMSE scores (MD: −0.13, 1.02; *p* = 0.02, 0.0007). G2385R carriers had higher LEDD and tended to develop motor complications, such as motor fluctuations (MD: 53.22, OR: 3.17; *p* = 0.01, < 0.00001) than non-carriers. Other clinical presentations did not feature G2019S or G2385R. We observed no distinct clinical features for R1628P or R1441G. Our subgroup analyses in different ethnic group for specific variant also presented with relevant clinical characteristics of PD patients.

**Conclusions:** Clinical heterogeneity was observed among *LRRK2*-associated PD in different variants in total and in different ethnic groups, especially for G2019S and G2385R.

## Introduction

Parkinson's disease (PD) is the second-most common neurodegenerative disease, with major clinical features comprising motor symptoms (MS) and non-motor symptoms (NMS). MS are characterized by four cardinal symptoms: bradykinesia, resting tremor, rigidity, and postural instability. NMS include olfactory dysfunction, constipation, depression, and sleep disturbance (Konno et al., 2018). Levodopa (L-dopa) is a classic treatment for parkinsonism; however, this drug is known to induce motor complications, such as dyskinesia and motor fluctuations that may affect the quality of life of PD patients (Olanow and Stocchi, 2017; Picconi et al., 2017).

In recent times, the pathogenesis of PD often remains unclear. Genetic factor, environmental factor and aging all contribute to PD pathogenesis (Liu et al., 2016; Yan et al., 2017; Zhang et al., 2018). Leucine-rich repeat kinase 2 (*LRRK2*) is considered the most common genetic cause of PD (Paisan-Ruiz, 2009; Guo et al., 2015; Li et al., 2015); an increasing number of studies have focused on the genotype and phenotype analysis of *LRRK2* and PD. Whether the clinical features of *LRRK2*-associated PD differ from those of idiopathic PD (IPD) is still debatable. Some researchers believe that *LRRK2*-related PD has similar clinical onset features to IPD, such as resting tremor, good response to L-dopa, and a benign clinical course (Orr-Urtreger et al., 2007; Paisan-Ruiz, 2009; Zheng et al., 2015). However, others have reported that *LRRK2*-related PD has distinct features that differ from those of IPD and vary between different genotypes (Marras et al., 2016). For example, original studies have reported that G2019S carriers are more likely to be women, less likely to develop olfactory dysfunction, and more likely to have dyskinesia and dystonia than non-carriers (Marras et al., 2016). G2385R carriers have been observed to exhibit a tendency toward motor fluctuations and are more likely to have postural instability gait disorder phenotype (Oosterveld et al., 2015). Additionally, some researchers carried out analyses of *LRRK2*-associated clinical features by combining different variants, without considering the different clinical features among the different variants (Paisan-Ruiz et al., 2013). Considering the heterogeneous risk of *LRRK2* variants in PD, it is thus vital to provide evidence, via pooled analysis, to identify specific *LRRK2* variants associated with clinical phenotypes.

Our previous comprehensive meta-analysis demonstrated the importance of *LRRK2* SNPs, such as G2385R, G2019S, R1628P in PD (data unpublished). To further explore the role of *LRRK2* SNPs in PD clinical features, here, we conducted a complete analysis of clinical features in specific *LRRK2* variants related to PD.

## Methods

### Literature search

Medline database in PubMed, Embase database in Ovid, and the Cochrane databases were electronically searched by the authors for publications in English. The key words used were “Parkinson^*^,” “PD,” “*LRRK2*,” and “PARK8.” The data were assessed online on February 10, 2018. Overlapping articles from different databases were excluded. Two researchers (Li Shu and Yuan Zhang) performed the search independently. In case of disagreements, a third researcher (Qiying Sun) was consulted to arrive at a consensus.

### Selection criteria

The PICOS (participants, interventions, controls, outcomes, and study types) principle was applied in the inclusion process.

Participants: all PD patients were diagnosed according to widely accepted criteria (Hughes et al., 1992) and carried specific *LRRK2* variants.

Interventions: genetic analyses were conducted using genomic DNA by PCR-based methods or other accepted methods.

Controls: controls were PD patients without specific *LRRK2* variants.

Outcomes: available data to calculate the number of carriers and non-carriers of the responsive phenotypes.

Study types: original case only study, case-control study, or cohort study.

### Data extraction

**Figure 1:**
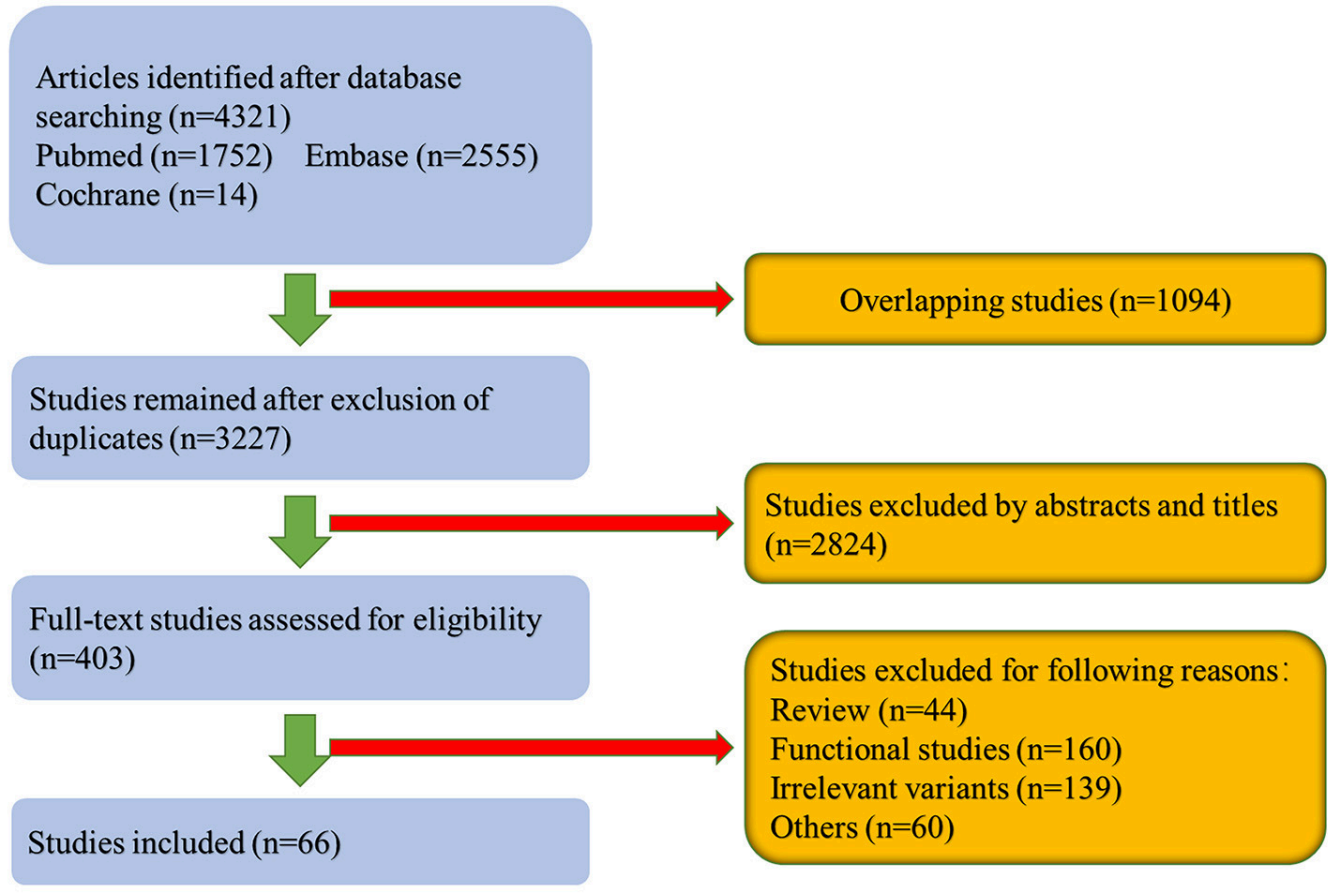
Flowchart showing the literature screening process.

Complete data including first author, publication year, ethnicity, country, gene, variants, numbers of cases, and their responsive clinical features were extracted by two researchers (Li Shu and Yuan Zhang). If there were disputes in the process, a third author was asked to solve the problem (Qiying Sun; Table 1; Supplementary Table 1). The process of data extraction is shown in the flowchart (Figure 1). Briefly, we included studies that defined age at onset ≤ 50 years as early-onset PD (EOPD) and age at onset >50 years as late-onset PD (LOPD).

**Table 1:** The publications included for phenotype analysis.

**References**	**Ethnicity**	**Country**	**Variants**	**No. patients**
Bras et al., 2005	European/West Asians	Portugal	G2019S	128
Lesage et al., 2006	Africans	North Africa	G2019S	106
Gaig et al., 2006	European/West Asians	Spain	G2019S	302
Di Fonzo et al., 2006	East Asians	China	G2385R	608
Goldwurm et al., 2006	European/West Asians	Italy	G2019S	1,092
Kay et al., 2006	Mixed	America	G2019S	1,518
Clark et al., 2006	Mixed	America	G2019S	504
Ishihara et al., 2007	Africans	Tunis	G2019S	201
Fung et al., 2006	East Asians	Taiwan	G2385R	305
Farrer et al., 2007	East Asians	Taiwan	G2385R	410
Tan et al., 2007	East Asians	Singapore	G2385R	62
Funayama et al., 2007	East Asians	Japan	G2385R	448
Orr-Urtreger et al., 2007	European/West Asians	Israel	G2019S	344
Li et al., 2007	East Asians	China	G2385R	235
An et al., 2008	East Asians	China	G2385R	600
Gan-Or et al., 2008	European/West Asians	Israel	G2019S	128
Chan et al., 2008	East Asians	China	G2385R	34
Hulihan et al., 2008	Africans	Tunis	G2019S	238
Pankratz et al., 2008	Mixed	North America	G2019S	840
Mata et al., 2009	Hispanics	Peru,Uruguay	G2019S,R1441C	360
Lesage et al., 2008	Africans	North Africa	G2019S	136
Latourelle et al., 2008	Mixed	America	G2019S,R1441C	1,025
Gan-Or et al., 2008	European/West Asians	Isreal	G2019S	477
Zhang et al., 2009	East Asians	China	R1628P	600
Yu et al., 2009	East Asians	China	R1628P	328
Kim et al., 2010	East Asians	Korea	G2385R	923
Alcalay et al., 2009	Mixed	America	G2019S	691
Belarbi et al., 2010	Africans	Algeria	G2019S	106
Shanker et al., 2011	European/West Asians	Isreal	G2019S	42
Hashad et al., 2011	Africans	Egypt	G2019S	113
Saunders-Pullman et al., 2011a	Mixed	Israel and America	G2019S	61
Marras et al., 2011	Mixed	Canada, Germany and Brazil	G2019S	109
Ben Sassi et al., 2012	Africans	Tunis	G2019S	110
Yahalom et al., 2012	European/West Asians	Israel	G2019S	349
Yan et al., 2012	East Asians	China	G2385R	354
Fu et al., 2013	East Asians	China	G2385R, R1628P	446
Sierra et al., 2013	European/West Asians	Spain	G2019S	79
Gatto et al., 2013	Hispanics	Argentina	G2019S	55
Cai et al., 2013	East Asians	China	G2385R, R1628P	510
Tijero et al., 2013	European/West Asians	Spain	G2019S, R1441C	19
Greenbaum et al., 2013	European/West Asians	Israel	G2019S	39
Gao et al., 2013	East Asians	China	G2385R	175
Mirelman et al., 2013	European/West Asians	Israel	G2019S	100
Alcalay et al., 2013	Mixed	Israel and America	G2019S	488
Trinh et al., 2014	Africans	Tunis	G2019S	570
Yahalom et al., 2014	European/West Asians	Israel	G2019S	405
Pulkes et al., 2014	East Asians	Thailand	R1628P	485
Estanga et al., 2014	European/West Asians	Spain	R1441G	60
Gaig et al., 2014	European/West Asians	Spain	G2019S	66
Alcalay et al., 2015	Mixed	MJFF center	G2019S	236
Nabli et al., 2015	Africans	Tunis	G2019S	58
Saunders-Pullman et al., 2014	Mixed	America or Israel	G2019S	252
Somme et al., 2015	European/West Asians	Spain	G2019S, R1441C	54
Marder et al., 2015	Mixed	MJFF center	G2019S	474
Vilas et al., 2015	European/West Asians	Spain	G2019S	57
Saunders-Pullman et al., 2015	Mixed	America	G2019S	286
Marras et al., 2016	Mixed	MJFF center	G2019S, G2385R	1,602
Sun et al., 2016	East Asians	China	G2385R	301
Dagan et al., 2016	European/West Asians	Israel	G2019S	211
Cao et al., 2016	East Asians	China	G2385R	68
Pal et al., 2016	Mixed	CORE-PD	G2019S	76
Hong et al., 2017	East Asians	Korea	G2385R	299
Bouhouche et al., 2017	Africans	Morocco	G2019S	100
da Silva et al., 2017	Hispanics	Brazil	G2019S	110
San Luciano et al., 2017	Mixed	MJFF center	G2019S	1,289
Saunders-Pullman et al., 2018	Mixed	Israel, USA	G2019S	545

### Statistical analysis

Revman 5.3 software was used for all statistical analyses. Pooled odds ratio (ORs) or pooled mean difference (MD) and 95% CIs were calculated to estimate dichotomous data or continuous data about the importance of polymorphisms to the risk of phenotypes. Q statistic and I^2^ statistic indicated heterogeneity of the analysis. If the heterogeneity was not significant (*p* > 0.1, *I*^2^ < 50%), a fixed model (FM) was used for further analysis. However, if the heterogeneity was significant (*p* < 0.1, *I*^2^ > 50%), a random model (RM) was applied. Publication biases were measured using funnel plots. Sensitivity analyses were performed by eliminating papers one at a time, and the changes in the total results were observed.

## Results

### The selection process and presentation of the final results

A flowchart depicting the publication search process is shown in Figure 1. A total of 4,307 articles were retrieved after searching the databases. After excluding 1,080 overlapping articles from different databases as well as 3,161 articles that did not meet the selection criteria, 66 studies comprising 23,402 patients were considered for the final meta-analysis of phenotypes of specific *LRRK2* variants related to PD. Forty-six articles comprising 16,016 patients were included for analysis of the clinical features of *LRRK2*-G2019S-related PD. Seventeen articles involving 6,767 patients were included for analysis of clinical presentations of *LRRK2*-G2385R-related PD; out of these, 5 *LRRK2*-R1628P–related articles comprising 2,369 patients and 5 *LRRK2*-R1441G–related articles comprising 1,222 patients were included in the final meta-analysis. The characteristics of the included studies in each analysis are shown in Table 1; Supplementary Table 1. The total results of meta-analyses on these four variants were shown in Table 2; Supplementary Figure 1; Supplementary Table 2. Besides, subgroup analyses of each variant were conducted in different ethnic groups (Africans, European/West Asians, Hispanics, East Asians and Mixed: composed of at least two different groups) according to ethnic classifications by Risch, N et al. (Risch et al., 2002; Supplementary Tables 3,4; Supplementary Figure 3).

**Table 2:** The results of phenotype-association analysis of each variant of *LRRK2*.

**LRRK2 phenotypes or rating scales**	**G2019S**	**G2385R**	**R1628P**	**R1441G**
**DEMOGRAPHIC INFORMATION**				
Asymmetrical onset	−	−	NA	NA
Age at onset	−	−	−	NA
Early onset	+	−	−	NA
Male	+	−	−	–
Family history	+	+	NA	NA
**FIRST SYMPTOMS**				
FS-Bradykinesia	−	−	NA	NA
FS-Resting tremor	−	−	−	NA
FS-Rigidity	−	−	NA	NA
FS-Postural instability or Gait difficulty	−	−	NA	NA
FS-Dystonia	−	NA	NA	NA
FS-Micrographia	−	NA	NA	NA
**MOTOR SYMPTOMS**				
Bradykinesia	−	NA	NA	NA
Resting tremor	−	−	NA	NA
Rigidity	−	−	NA	NA
Postural instability or gait difficulty	−	NA	NA	NA
**MOTOR PHENOTYPE CLASSIFICATIONS**				
T-Akinetic-rigid/PIGD	−	NA	NA	NA
T-Mixed/Intermediate	−	NA	NA	NA
T-Tremor-dominant	−	NA	NA	NA
**SCALES EVALUATING DISEASE SEVERITIES**				
UPDRS I	−	−	NA	NA
UPDRS II	−	−	NA	NA
UPDRS III	−	−	NA	–
H-Y	−	+	−	NA
Schwab & England	+	NA	NA	NA
**MOTOR COMPLICATIONS**				
Dyskinesia	+	−	NA	NA
Motor fluctuations	+	+	NA	NA
**NEUROPSYCHIATRIC DISTURBANCES**				
Anxiety	−	NA	NA	NA
Depression	−	−	NA	NA
GDS15	+	NA	NA	NA
Hallucination	−	NA	NA	NA
**AUTONOMIC DISTURBANCES**				
SCOPA-AUT	−	NA	NA	NA
**COGNITIVE IMPAIRMENTS**				
Cognitive impairments	−	NA	NA	NA
MMSE	−	+	NA	NA
MoCA	−	NA	NA	NA
**SLEEP DISTURBANCES**				
Sleep disturbances	−	NA	NA	NA
**SENSORY COMPLAINTS**				
Olfactory disturbances	−	NA	NA	NA
UPSIT scores	+	NA	NA	NA
**TREATMENTS**				
Good response to l-dopa	+	NA	NA	NA
LEDD	+	+	NA	NA
**ENVIRONMENTAL FACTORS**				
Smoke	+	NA	NA	NA

*+, clinical symptoms related to a specific variant; −, clinical symptoms not related to a specific variant; NA, not available*.

### The clinical characteristics of LRRK2-G2019S carriers

In terms of the clinical features of specific variants, the present meta-analysis showed unique clinical manifestations in G2019S, G2385R, R1628P, and R1441G separately. In total, 40 specific clinical features or rating scales belonging to 13 classifications (Park and Stacy, 2009) were included in our meta-analysis of G2019S-related clinical characteristics (Table 2; Supplementary Table 2).

Our data show that G2019S carriers were predominantly female and had higher rates of EOPD and family history (OR: 0.77 [male], 1.37, 2.62; *p* < 0.00001, 0.02, < 0.00001). With respect to NMS, G2019S carriers tended to have lower Geriatric Depression Scale (GDS) scores and higher University of Pennsylvania Smell Identification Test (UPSIT) scores than non-carriers (MD: 0.43, 4.70; *p* = 0.01, < 0.00001). In terms of the response to treatment, the G2019S carriers showed good response to L-dopa (OR: 2.33; *p* < 0.0001) and higher Schwab and England Activity of Daily Living Scale scores (Schwab & England; MD: 1.49; *p* < 0.00001). Further, the G2019S carriers received a statistically higher Levodopa equivalent daily dose (LEDD) (MD: 115.20; *p* < 0.00001) and were more likely to develop motor complications, such as dyskinesia and motor fluctuations than non-carriers (OR: 2.18, 2.02; *p* < 0.00001, 0.04). Other clinical presentations did not feature G2019S.

In subgroup analyses by ethnicity, in Africans, the higher scores of UPDRSIII, more likely to develop dyskinesia were found in PD patients with G2019S variant than without G2019S variant (MD: 4.79, OR: 2.61; *p*: 0.0005, < 0.0001). In European/West Asians, the G2019S carriers tended to have earlier age at onset, be female, have higher rates of EOPD, family history and higher LEDD than non-carriers (MD: −2.44, OR: 0.63 [male], 1.48, 2.98, MD: 102.43; *p*: 0.001, < 0.0001, 0.01, < 0.00001, 0.02). In Hispanics, family history characterized G2019S carriers (OR: 4.66; *p*: 0.0003). In mixed ethnic group, the G2019S carriers were more likely to be female, have family history (OR: 0.77 [male], 2.22; *p*: < 0.00001, < 0.00001). And the carriers tended to develop akinetic-rigid motor phenotype, dyskinesia, have lower GDS scores, better response to levodopa (l-dopa), higher LEDD and smoking rates (OR: 1.85, 2.37, MD: 0.44, 2.80, 129.87, OR: 1.57; *p*: 0.0007, < 0.00001, 0.01, < 0.0001, < 0.00001, 0.0002). There were not enough data to analyze G2019S-related clinical features in East Asians in PD (Supplementary Tables 3, 4).

### Clinical characteristics of LRRK2-G2385R carriers

In total, 20 specific clinical features or rating scales belonging to eight classifications (Park and Stacy, 2009) were included in our meta-analysis of G2385R-related clinical characteristics (Table 2; Supplementary Table 2). In the analysis of G2385R-related clinical features, we demonstrated that PD patients with G2385R variants were more likely to have family history (OR: 2.10; *p* = 0.007). With respect to the NMS, G2385R carriers had higher Mini Mental State Examination (MMSE) scores than non-carriers (MD: 1.02; *p* = 0.0007). In terms of MS, G2385R carriers had lower Hoehn and Yahr rating (H-Y) than non-carriers (MD: −0.13; *p* = 0.02). In terms of treatment, G2385R carriers received statistically higher LEDD (MD: 53.22; *p* = 0.01) and were more likely to develop motor fluctuations than non-carriers (OR: 3.17; *p* < 0.00001). In terms of the other clinical features of G2385R, no statistically significant differences were observed between carriers and non-carriers.

In subgroup analyses by ethnicity, in East Asians, the PD patients with G2385R variant tended to have family history, develop motor fluctuations and have higher MMSE scores (OR: 2.1, 3.84, MD: 1.02; *p*: 0.007, < 0.00001, 0.0007). There were not enough data to analyze G2385R-related clinical features in other ethnic groups in PD (Supplementary Tables 3, 4).

### The clinical characteristics of LRRK2-R1628P or R1441G carriers

In the analysis of R1628P-related clinical features, we included demographic information, such as age at onset, EOPD rates, sex, first symptoms, such as resting tremor, and H-Y rating. In the analysis of R1441G- related clinical characteristics, gender and UPDRSIII scores were included in the pooled analysis. No significant differences between carriers and non-carriers were observed in terms of the clinical characteristics of R1628P and R1441G. The forest plots of each analysis are shown in Supplementary Figure 1. There were no positive results of the two variants in subgroup analyses by ethnicity (Supplementary Tables 3, 4).

### Statistical sensitivity and bias analysis

Articles included in the analysis focused on the relationships between the common polymorphisms and PD phenotypes. Most funnel plots of all analyses were symmetric, which indicated that there was little publication bias in the meta-analysis, except for some phenotypes (Supplementary Figures 2, 4). According to the sensitivity analysis, the pooled OR and 95% confidence interval (CI) did not change significantly when deleting each included article one at a time. The pooled OR for each analysis was stable.

## Discussion

The present meta-analysis was a comprehensive pooled analysis of specific variants in *LRRK2* and their associated clinical features. Detailed genotype and phenotype data were all completely included in the meta-analysis for comprehensive exploration of the important role of *LRRK2* variants in PD risk.

We found that carriers of *LRRK2* variants had distinct clinical features compared with non-carriers. Unique features differed between *LRRK2* variants. In previous studies of *LRRK2*-related PD, researchers characterized *LRRK2*-related clinical features in patients carrying any of the *LRRK2* variants, while ignoring the unique clinical features of each specific variant (Vilas et al., 2016; De Rosa et al., 2018). For example, De Rosa A et al. considered similar cognitive functions between carriers of *LRRK2* G2019S or R1441G and non-carriers (De Rosa et al., 2018). However, studies of specific variants, such as R1441G reported lower likelihood of developing significant cognitive dysfunction than in IPD (Somme et al., 2015). Therefore, it is necessary to discuss the unique clinical features of *LRRK2*-related PD based on each specific variant.

With respect to demographic features, G2019S carriers were more likely to be female and have higher rates of EOPD and family history, while G2385R carriers were more likely to have family history. The reason that *LRRK2*-G2019S carriers had a higher rates of family history is that the mutation is a pathogenic non-synonymous amino acid substitution and may be a cause of familial parkinsonism besides its role in sporadic PD as a SNP like G2385R or R1628P (Mata et al., 2005). While the heritability of EOPD is high, the carriers of *LRRK2*-G2019S had higher rates of EOPD than non-carriers (Clark et al., 2006). The gender differences between *LRRK2*-G2019S carriers and non-carriers in PD may due to a heavier genetic load of female than male in PD as manifested by our analysis. Female who developed PD tended to have higher rates of genetic PD which manifested a higher rates of family history than male (Saunders-Pullman et al., 2011b). In our previous research of *LRRK2* in PD, we recommended screening for specific race-associated variants, such as G2019S in Caucasian and G2385R in Asian populations (data unpublished). This previous analysis further suggested that specific demographic features, such as female sex, EOPD, or family history may be used to select a targeted population for *LRRK2* screening when conducting or planning research.

Previous research indicated a more benign clinical course of G2019S-related PD compared with that for IPD; for example, lower incidence of falls, dyskinesia, cognition, and olfaction dysfunctions (Healy et al., 2008; Haugarvoll and Wszolek, 2009; Marras et al., 2011). The results of the present pooled analysis were consistent with previous results, in that G2019S carriers tended to have better quality of life as reflected by the Schwab & England scale, are less likely to be depressed, less prone to olfactory dysfunction, and show better response to L-dopa than non-carriers. The present finding that G2019S carriers exhibit less severe olfactory dysfunction was consistent with previous findings showing that abnormal olfaction function was present in up to 49% of patients, which is much lower than in IPD. (Healy et al., 2008). Other studies have suggested that *LRRK2*-G2019S is associated with abnormal olfactory function as a result of effects on Lewy body pathology in the rhinencephalon (Silveira-Moriyama et al., 2008; Kalia et al., 2015). G2385R carriers also presented with consistent benign clinical features, such as lower H-Y rating than non-carriers. With respect to NMS, G2385R carriers were less likely to have cognitive impairments than IPD patients, as reflected by the higher MMSE scores. We also did subgroup analyses by ethnicity and found clinical features of PD patients with *LRRK2* variants especially G2019S and G2385R in specific ethnic groups. Although there were articles discussing about the genotype-phenotype correlations of *LRRK2* in PD (Kestenbaum and Alcalay, 2017; Koros et al., 2017), our analysis is a pilot study which controlled the race variable and found clinical features of PD with *LRRK2* variants in different ethnic groups.

The impact of pharmacogenetics on the efficacy and side effects of treatment is widely studied in the context of PD therapeutics; susceptible variants or genes have been shown to be associated with the appropriate therapeutic dosage of L-dopa or relevant motor complications. Genetic variants, such as *DRD3* G3127A in the dopamine receptor (DR) gene have been shown to be involved in L-dopa-induced dyskinesia (Comi et al., 2017). G2385R in *LRRK2* was previously found to be significantly associated with motor complications in female PD patients (Gao et al., 2013). Our analysis demonstrated that G2019S or G2385R carriers tend to develop motor complications with significant differences, consistent with higher LEDD, relative to non-carriers (Cacabelos, 2017). As motor complications are known to severely damage the life quality of PD patients, it is our duty, as clinicians, to minimize complications, such as dyskinesia and motor fluctuations. Our results enable deeper understanding of the underlying genetic characteristics of PD by highlighting G2019S or G2385R variants in *LRRK2* as predictors for the development of motor complications, in addition to the well-established factors, such as young age at onset, higher L-dopa dose, and low body weight (Warren Olanow et al., 2013). Motor complications should be carefully considered when such patients are treated with high doses of L-dopa.

The present comprehensive analysis provides strong support for the distinct clinical features associated with different *LRRK2* variants, which indicate a phenotype-genotype correlation in PD. There were phenotype-genotype correlations of other PD causative genes, such as *Parkin, PINK1*, and *DJ1*. Besides widely known clinical features of these genes like good treatment response and dyskinesia, systematic review found discrepancies from reviews or original articles. For example, *Parkin* mutation carriers tended to present with late age at onset and not have sleep benefit (Kasten et al., 2018). We were additionally able to predict the clinical course of PD in patients with a specific *LRRK2* variant and treat these patients more precisely with early intervention to delay disease progression and control complications. The heterogeneous clinical symptoms for *LRRK2*-G2019S or G2385R-related PD may indicate a distinct pathophysiology in variant carriers; however, the underlying mechanisms remain elusive.

Given the nature of case-control original articles, the present meta-analysis has some inevitable limitations. First, because of the lack of sufficient data and small sample size, we were unable to perform a meta-analysis of the relationship between other widely researched variants, such as A419V and R1398H (data unpublished) and the phenotypes of PD. Therefore, more comprehensive data are needed to perfect this meta-analysis. Second, heterogeneities existed among original studies in our pooled analysis; with a greater availability of original articles, it is advisable to adjust the pooled results and aim for more robust evidence. Third, co-occurrence and interaction between the factors could not be analyzed in the present meta-analysis, which may have confounded the results.

## Conclusion

Clinical heterogeneity in *LRRK2*-associated PD among different variants, especially for G2019S and G2385R, was found to occur. We observed no distinct clinical features for R1628P or R1441G. The prominent clinical features of *LRRK2-*G2019S-related PD patients were female sex and higher rates of EOPD and family history. Further, G2019S carriers were less likely to be depressive and have olfactory dysfunctions, had better response to L-dopa and better quality of life than non-carriers. Furthermore, carriers tended to be treated with higher dose of L-dopa and were more likely to develop motor complications, such as dyskinesia and motor fluctuations. With respect to the clinical symptoms of G2385R carriers, this group was more likely to have more family history, lower H-Y rating, and was less likely to develop cognitive dysfunctions than non-carriers. High-dose L-dopa treatment and related motor fluctuations were more likely to occur in PD patients carrying the G2385R variant. Other clinical presentations did not feature G2019S or G2385R. No distinct clinical features were found in R1628P or R1441G variants. Our subgroup analyses in different ethnic group also presented with relevant clinical characteristics of PD patients with G2019S and G2385R but not of R1628P or R1441G.

## Author contributions

LS and YZ have contributed equally to this work and are co-first authors. LS, YZ, and QS chose the topic and designed the experiments; LS, YZ, and QS performed the analysis; LS, YZ, QS, and BT analyzed the data; LS, YZ, and QS wrote the manuscript; HP, QX, and JG: data management and figure modification.

### Conflict of interest statement

The authors declare that the research was conducted in the absence of any commercial or financial relationships that could be construed as a potential conflict of interest.
