# Supplementary material for: Clinical Heterogeneity Among LRRK2 Variants in Parkinson's Disease: A Meta-Analysis
Source: Front Aging Neurosci. 2018 Sep 19;10:283. doi: 10.3389/fnagi.2018.00283 (PMC6156433; doi:10.3389/fnagi.2018.00283)
Supplement: Supplementary file 1 [file Table_1.DOCX]

Supplementary Material

**Clinical heterogeneities among *LRRK2* variants in Parkinson’s disease: a meta-analysis**

**Li Shu^2 †^, Yuan Zhang^2 †^, Hongxu Pan^2^, Qian Xu^2, 3,4^, Jifeng Guo^2,3,4,6,7,8^, Beisha Tang^1, 2,3,4,5,6,7,8^, Qiying Sun^1,3,4*^**

**^†^** These authors have contributed equally to this work and are co-first authors.

^*^ **Correspondence**: Qiying Sun [sunqiying2015@163.com](mailto:sunqiying2015@163.com)

Supplementary Table 1 The characteristics of all included publications for phenotype analysis. *: clinical features analyzed in our meta-analysis.

| Publication year | First author | Variants | Phenotypes researched |
| --- | --- | --- | --- |
| 2005 | Jose Miguel Bras | G2019S | Family history,Cognitive impairments |
| 2006 | Lesage S | G2019S | Age at onset,Male,FS-Bradykinesia,FS-Resting tremor,FS-Dystonia,FS-Micrographia,Bradykinesia,Resting tremor,Rigidity,UPDRSⅢ,Dyskinesia,MMSE,Good response to l-dopa,LEDD |
| 2006 | Gaig, C. | G2019S | Family history |
| 2006 | Di Fonzo, A. | G2385R | Early onset,Male |
| 2006 | Goldwurm, S. | G2019S | Early onset,Family history |
| 2006 | DENISE M. KAY | G2019S | Early onset,Male,Family history |
| 2006 | L.N. Clark | G2019S | Asymmetrical onset,Age at onset,Early onset,Male,Family history,FS-Bradykinesia,FS-Resting tremor,FS-Rigidity,FS-Postural instability or Gait difficulty,Bradykinesia,Resting tremor,Rigidity,Postural instability or Gait difficulty,UPDRSⅢ,H-Y,Good response to l-dopa |
| 2007 | Ishihara, L. | G2019S | Age at onset,T-Akinetic-rigid/PIGD,T-Mixed/Intermediate,T-Tremor-dominant,UPDRSⅢ,H-Y,Schwab & England |
| 2006 | Hon-Chung Fung | G2385R | Asymmetrical onset,Age at onset,Resting tremor,Rigidity |
| 2007 | Matthew J. Farrer | G2385R | Age at onset,Male,Family history,FS-Bradykinesia,Resting tremor,Rigidity,UPDRSⅢ,H-Y,Dyskinesia,Motor fluctuations,LEDD |
| 2007 | Eng-King Tan | G2385R | Age at onset,Male,Family history,UPDRSⅢ,Dyskinesia,LEDD |
| 2006 | Manabu Funayama | G2385R | Age at onset,Early onset |
| 2007 | A. Orr-Urtreger(1) | G2019S | Age at onset,Early onset,Male,Family history,Bradykinesia,Resting tremor,Rigidity,Postural instability or Gait difficulty |
| 2007 | Cao Li | G2385R | Age at onset,Early onset,Male,Family history,FS-Bradykinesia,FS-Resting tremor,FS-Rigidity,FS-Postural instability or Gait difficulty,UPDRSⅠ,UPDRSⅡ,UPDRSⅢ,H-Y,Dyskinesia |
| 2008 | X.-K. An | G2385R | Age at onset,Early onset,Male,FS-Resting tremor,H-Y |
| 2008 | Gan-Or, Z. | G2019S | Age at onset,Early onset,Male,Family history |
| 2008 | Daniel Kam Yin Chan | G2385R | Asymmetrical onset,Male,Resting tremor,Rigidity,Motor fluctuations,Depression |
| 2008 | Mary M Hulihan | G2019S | Male,FS-Resting tremor,FS-Postural instability or Gait difficulty,FS-Dystonia,T-Akinetic-rigid/PIGD,T-Mixed/Intermediate,T-Tremor-dominant |
| 2008 | Pankratz, N. | G2019S | Depression |
| 2009 | Mata, I. F.(1) | G2019S,R1441C | Age at onset,Male,Family history/Male |
| 2008 | S. Lesage | G2019S | Age at onset,Male,FS-Bradykinesia,FS-Resting tremor,FS-Dystonia,FS-Micrographia,Bradykinesia,Resting tremor,Rigidity,UPDRSⅢ,Dyskinesia,Motor fluctuations,MMSE,LEDD |
| 2008 | Latourelle, J. C. | G2019S,R1441C | Male,Family history/Male |
| 2010 | Gan-Or, Z. | G2019S | Early onset,Male,Family history,FS-Bradykinesia,FS-Resting tremor,FS-Rigidity,FS-Postural instability or Gait difficulty |
| 2009 | Zijuan Zhang | R1628P | Age of onset,Early onset,FS-Resting tremor,H-Y |
| 2009 | Lihua Yu | R1628P | Age of onset,Early onset,Male,FS-Resting tremor,H-Y |
| 2010 | Jong-Min Kim | G2385R | Age at onset,Male, |
| 2009 | RN Alcalay | G2019S | Asymmetrical onset,Age at onset,Male,Family history,Resting tremor,T-Akinetic-rigid/PIGD,T-Tremor-dominant,UPDRSⅢ,Hallucination,MMSE,LEDD, |
| 2010 | Soreya Belarbi | G2019S | Male,Anxiety,Depression,Hallucination,Cognitive impairments,Sleep disturbances, |
| 2010 | Vicki Shanker | G2019S | Age at onset,Male,UPDRSⅡ,UPDRSⅢ,MMSE, |
| 2011 | Hashad, D. I. | G2019S | Asymmetrical onset,Age at onset,Early onset,FS-Bradykinesia,FS-Resting tremor,FS-Rigidity,UPDRSⅠ,UPDRSⅡ,UPDRSⅢ,H-Y, |
| 2011 | R. Saunders-Pullman | G2019S | Age at onset,Male,UPDRSⅢ,Olfactory disturbances,UPSIT score,Smoke |
| 2011 | C. Marras | G2019S | Age at onset,Male,FS-Bradykinesia,FS-Resting tremor,FS-Rigidity,FS-Postural instability or Gait difficulty,FS-Micrographia,Good response to l-dopa,LEDD, |
| 2012 | Ben Sassi, S. | G2019S | Age at onset,Male,T-Akinetic-rigid/PIGD,T-Mixed/Intermediate,T-Tremor-dominant,UPDRSⅢ,H-Y,Schwab & England,Depression,GDS15,Cognitive impairments,MMSE,MoCA,LEDD, |
| 2012 | Gilad Yahalom | G2019S | Age at onset,Family history,Dyskinesia, |
| 2012 | HUIRU YAN | G2385R | Early onset,Male, |
| 2013 | Xiaoli Fu | G2385R,R1628P | Age at onset,Early onset,Male,Family history,FS-Bradykinesia,FS-Resting tremor,FS-Rigidity,FS-Postural instability or Gait difficulty/Age of onset,Early onset,Male,FS-Resting tremor |
| 2013 | Maria Sierra | G2019S | Age at onset,Male,UPDRSⅢ, |
| 2013 | Emilia Mabel Gatto | G2019S | Age at onset,Male,Family history,Resting tremor,Motor fluctuations,Hallucination, |
| 2013 | Jiangping Cai | G2385R,R1628P | Age at onset,Early onset,Male,FS-Bradykinesia,FS-Resting tremor,FS-Rigidity,FS-Postural instability or Gait difficulty,UPDRSⅠ,UPDRSⅡ,UPDRSⅢ,H-Y/Age of onset,Early onset,Male,FS-Resting tremor,H-Y |
| 2013 | Beatriz Tijero | G2019S,R1441C | UPDRSⅠ,UPDRSⅡ,UPDRSⅢ,SCOPA-AUT/UPDRSⅢ |
| 2013 | Greenbaum, L. | G2019S | Age at onset,Male, |
| 2013 | Chao Gao | G2385R | Age at onset,Early onset,Male,Family history,MMSE, |
| 2013 | Mirelman, A. | G2019S | Male,Dyskinesia,GDS15,LEDD, |
| 2013 | Roy N. Alcalay | G2019S | Age at onset,Male,Family history,FS-Resting tremor,T-Akinetic-rigid/PIGD,UPDRSⅢ,Dyskinesia,GDS15,MoCA,Good response to l-dopa,LEDD, |
| 2013 | Trinh, J. | G2019S | Age at onset,Male,FS-Resting tremor,FS-Rigidity,FS-Postural instability or Gait difficulty,FS-Dystonia,T-Akinetic-rigid/PIGD,T-Mixed/Intermediate,T-Tremor-dominant,UPDRSⅡ |
| 2014 | Gilad Yahalom | G2019S | Age at onset,Early onset,Male,Family history, |
| 2014 | Pulkes, T. | R1628P | Age of onset,H-Y |
| 2014 | A. Estanga | R1441G | Male,UPDRSⅢ |
| 2014 | Carles Gaig | G2019S | Male,Bradykinesia,Resting tremor,Postural instability or Gait difficulty,UPDRSⅡ,UPDRSⅢ,H-Y,Schwab & England,Dyskinesia,Motor fluctuations,Anxiety,Depression,Hallucination,SCOPA-AUT,Sleep disturbances,Olfactory disturbances,UPSIT score,LEDD |
| 2015 | Alcalay, R. N. | G2019S | Age at onset,Male,T-Akinetic-rigid/PIGD,UPDRSⅢ,GDS15,MoCA,LEDD |
| 2015 | Nabli, F. | G2019S | Male,T-Akinetic-rigid/PIGD,T-Mixed/Intermediate,T-Tremor-dominant,UPDRSⅡ,UPDRSⅢ,H-Y,Dyskinesia,Motor fluctuations, |
| 2014 | Rachel Saunders-Pullman | G2019S | Male,UPDRSⅢ,MoCA,Olfactory disturbances,UPSIT score,Smoke |
| 2015 | Somme, J. H. | G2019S,R1441C | Male,UPDRSⅠ,UPDRSⅡ,UPDRSⅢ,Anxiety,Depression,Hallucination,Sleep disturbances,LEDD/Male,UPDRSⅢ |
| 2015 | Marder, K. | G2019S | Age at onset,Male, |
| 2015 | Vilas, D. | G2019S | Male, |
| 2015 | Saunders-Pullman, R. | G2019S | Age at onset,Male,UPDRSⅢ,Sleep disturbances, |
| 2016 | Marras, C. | G2019S,G2385R | Age at onset,Male,UPDRSⅡ,UPDRSⅢ,Schwab & England,Dyskinesia,Motor fluctuations,Olfactory disturbances,LEDD,Smoke/Age at onset,Male,UPDRSⅡ,UPDRSⅢ,Dyskinesia,Motor fluctuations,LEDD |
| 2016 | Sun, Qian | G2385R | Asymmetrical onset,Age at onset,Male,UPDRSⅠ,UPDRSⅡ,UPDRSⅢ,H-Y,Dyskinesia,Motor fluctuations,Depression,MMSE,LEDD |
| 2016 | Dagan, E. | G2019S | Male,Family history,Resting tremor,Rigidity,Postural instability or Gait difficulty,Cognitive impairments |
| 2016 | Cao, M. | G2385R | Male,H-Y |
| 2016 | Pal, G. D. | G2019S | Age at onset,UPDRSⅢ,Dyskinesia,MMSE |
| 2017 | Hong, J. H. | G2385R | Male,UPDRSⅢ,Depression,MMSE |
| 2017 | Bouhouche, A. | G2019S | Age at onset,Male,Family history,FS-Bradykinesia,FS-Resting tremor,Postural instability or Gait difficulty,T-Akinetic-rigid/PIGD,T-Mixed/Intermediate,T-Tremor-dominant,Dyskinesia,Motor fluctuations,Hallucination,Cognitive impairments,Sleep disturbances |
| 2017 | da Silva, C. P. | G2019S | Age at onset,Male,Family history,FS-Bradykinesia,FS-Resting tremor,FS-Rigidity,Bradykinesia,Resting tremor,Rigidity,Postural instability or Gait difficulty,Dyskinesia,Motor fluctuations,Depression,Hallucination,Cognitive impairments,Sleep disturbances,Olfactory disturbances,Good response to l-dopa |
| 2017 | San Luciano, M. | G2019S | Asymmetrical onset,Age at onset,Male,Family history,Dyskinesia,GDS15,SCOPA-AUT,MoCA,Olfactory disturbances |
| 2018 | Saunders-Pullman, R. | G2019S | Age at onset,Male,T-Akinetic-rigid/PIGD,T-Mixed/Intermediate,T-Tremor-dominant,UPDRSⅠ,UPDRSⅡ,UPDRSⅢ,H-Y,Schwab & England,MoCA,LEDD |
