# Supplementary material for: Clinical Heterogeneity Among LRRK2 Variants in Parkinson's Disease: A Meta-Analysis
Source: Front Aging Neurosci. 2018 Sep 19;10:283. doi: 10.3389/fnagi.2018.00283 (PMC6156433; doi:10.3389/fnagi.2018.00283)
Supplement: Supplementary file 2 [file Table_2.DOCX]

Supplementary Material

**Clinical heterogeneities among *LRRK2* variants in Parkinson’s disease: a meta-analysis**

**Li Shu^2 †^, Yuan Zhang^2 †^, Hongxu Pan^2^, Qian Xu^2, 3,4^, Jifeng Guo^2,3,4,6,7,8^, Beisha Tang^1, 2,3,4,5,6,7,8^, Qiying Sun^1,3,4*^**

**^†^** These authors have contributed equally to this work and are co-first authors.

^*^ **Correspondence**: Qiying Sun [sunqiying2015@163.com](mailto:sunqiying2015@163.com)

**Supplementary Table 2 The results of phenotype association analysis of each variants of *LRRK2*. Abbreviation: FS: first symptoms; LEDD, Levodopa equivalent daily doses; NA, not available. All results were shown as OR [95%CI] and *p* values. Bold OR or MD, 95%CI and *p* values reflected statistically significance results.**

|  | *LRRK2* phenotypes or rating scales | G2019S | | G2385R | | R1628P | | R1441G | |
| --- | --- | --- | --- | --- | --- | --- | --- | --- | --- |
| Demographic information |  |  |  |  |  |  |  |  |  |
|  | Asymmetrical onset | 1.06 [0.86, 1.32] | 0.57 | 0.29 [0.02, 4.33] | 0.37 | NA | NA | NA | NA |
|  | Age at onset | -0.46 [-1.64, 0.72] | 0.44 | -0.66 [-2.01, 0.70] | 0.34 | -1.11 [-3.30, 1.07] | 0.32 | NA | NA |
|  | Early onset | **1.37 [1.05, 1.79]** | **0.02** | 1.14 [0.89, 1.46] | 0.31 | 0.95 [0.49, 1.83] | 0.87 | NA | NA |
|  | Male | **0.77 [0.70, 0.84]** | **<0.00001** | 0.98 [0.73, 1.34] | 0.92 | 1.54 [0.99, 2.38] | 0.06 | 0.72 [0.35, 1.47] | 0.36 |
|  | Family history | **2.62 [2.25, 3.06]** | **<0.00001** | **2.10 [1.22, 3.59]** | **0.007** | NA | NA | NA | NA |
| First symptoms |  |  |  |  |  |  |  |  |  |
|  | FS-Bradykinesia | 1.16 [0.59, 2.26] | 0.67 | 0.92 [0.45, 1.85] | 0.8 | NA | NA | NA | NA |
|  | FS-Resting tremor | 0.70 [0.49, 1.02] | 0.06 | 1.12 [0.81, 1.54] | 0.51 | 0.85 [0.60, 1.21] | 0.36 | NA | NA |
|  | FS-Rigidity | 1.42 [0.96, 2.12] | 0.08 | 1.16 [0.71, 1.89] | 0.55 | NA | NA | NA | NA |
|  | FS-Postural instability or Gait difficulty | 1.16 [0.81, 1.67] | 0.42 | 1.00 [0.40, 2.48] | 1 | NA | NA | NA | NA |
|  | FS-Dystonia | 1.65 [0.77, 3.55] | 0.2 | NA | NA | NA | NA | NA | NA |
|  | FS-Micrographia | 0.29 [0.02, 3.53] | 0.33 | NA | NA | NA | NA | NA | NA |
| Motor symptoms |  |  |  |  |  |  |  |  |  |
|  | Bradykinesia | 0.98 [0.58, 1.65] | 0.94 | NA | NA | NA | NA | NA | NA |
|  | Resting tremor | 1.11 [0.80, 1.55] | 0.54 | 1.38 [0.68, 2.82] | 0.37 | NA | NA | NA | NA |
|  | Rigidity | 1.22 [0.77, 1.94] | 0.4 | 1.53 [0.50, 4.68] | 0.45 | NA | NA | NA | NA |
|  | Postural instability or Gait difficulty | 1.18 [0.84, 1.66] | 0.33 | NA | NA | NA | NA | NA | NA |
| Motor phenotype classifications |  |  |  |  |  |  |  |  |  |
|  | T-Akinetic-rigid/PIGD | 1.23 [0.88, 1.73] | 0.22 | NA | NA | NA | NA | NA | NA |
|  | T-Mixed/Intermediate | 1.09 [0.75, 1.60] | 0.64 | NA | NA | NA | NA | NA | NA |
|  | T-Tremor-dominant | 0.77 [0.49, 1.21] | 0.26 | NA | NA | NA | NA | NA | NA |
| Scales evaluating disease severities |  |  |  |  |  |  |  |  |  |
|  | UPDRSⅠ | -0.64 [-1.48, 0.20] | 0.14 | -0.16 [-0.51, 0.19] | 0.38 | NA | NA | NA | NA |
|  | UPDRSⅡ | -0.38 [-1.13, 0.36] | 0.31 | 0.08 [-3.12, 3.27] | 0.96 | NA | NA | NA | NA |
|  | UPDRSⅢ | 0.99 [-0.72, 2.71] | 0.26 | -0.00 [-4.99, 4.99] | 1 | NA | NA | -1.77 [-5.28, 1.75] | 0.32 |
|  | H-Y | -0.06 [-0.29, 0.18] | 0.64 | **-0.13 [-0.24, -0.02]** | **0.02** | 0.12 [-0.25, 0.49] | 0.52 | NA | NA |
|  | Schwab & England | **1.49 [1.41, 1.57]** | **<0.00001** | NA | NA | NA | NA | NA | NA |
| Motor complications |  |  |  |  |  |  |  |  |  |
|  | Dyskinesia | **2.18 [1.79, 2.67]** | **<0.00001** | 0.78 [0.54, 1.14] | 0.2 | NA | NA | NA | NA |
|  | Motor fluctuations | **2.02 [1.03, 3.97]** | **0.04** | **3.17 [2.30, 4.38]** | **<0.00001** | NA | NA | NA | NA |
| Neuropsychiatric disturbances |  |  |  |  |  |  |  |  |  |
|  | Anxiety | 1.19 [0.58, 2.44] | 0.63 | NA | NA | NA | NA | NA | NA |
|  | Depression | 1.33 [0.66, 2.69] | 0.42 | 1.42 [0.88, 2.29] | 0.15 | NA | NA | NA | NA |
|  | GDS15 | **0.43 [0.09, 0.77]** | **0.01** | NA | NA | NA | NA | NA | NA |
|  | Hallucination | 1.07 [0.67, 1.71] | 0.78 | NA | NA | NA | NA | NA | NA |
| Autonomic disturbances |  |  |  |  |  |  |  |  |  |
|  | SCOPA-AUT | 0.29 [-0.76, 1.34] | 0.59 | NA | NA | NA | NA | NA | NA |
| Cognitive impairments |  |  |  |  |  |  |  |  |  |
|  | Cognitive impairments | 1.49 [0.99, 2.24] | 0.06 | NA | NA | NA | NA | NA | NA |
|  | MMSE | -0.01 [-0.45, 0.43] | 0.95 | **1.02 [0.43, 1.62]** | **0.0007** | NA | NA | NA | NA |
|  | MoCA | 0.12 [-0.17, 0.40] | 0.42 | NA | NA | NA | NA | NA | NA |
| Sleep disturbances |  |  |  |  |  |  |  |  |  |
|  | Sleep disturbances | 1.17 [0.56, 2.41] | 0.68 | NA | NA | NA | NA | NA | NA |
| Sensory complaints |  |  |  |  |  |  |  |  |  |
|  | Olfactory disturbances | 0.81 [0.28, 2.36] | 0.7 | NA | NA | NA | NA | NA | NA |
|  | UPSIT scores | **4.70 [3.18, 6.22]** | **<0.00001** | NA | NA | NA | NA | NA | NA |
| Treatments |  |  |  |  |  |  |  |  |  |
|  | Good response to l-dopa | **2.33 [1.54, 3.54]** | **<0.0001** | NA | NA | NA | NA | NA | NA |
|  | LEDD | **115.20 [84.47, 145.93]** | **<0.00001** | **53.22 [11.67, 94.77]** | **0.01** | NA | NA | NA | NA |
| Environmental factors |  |  |  |  |  |  |  |  |  |
|  | Smoke | **1.57 [1.24, 1.99]** | **0.0002** | NA | NA | NA | NA | NA | NA |
