# Supplementary material for: Clinical Heterogeneity Among LRRK2 Variants in Parkinson's Disease: A Meta-Analysis
Source: Front Aging Neurosci. 2018 Sep 19;10:283. doi: 10.3389/fnagi.2018.00283 (PMC6156433; doi:10.3389/fnagi.2018.00283)
Supplement: Supplementary file 3 [file Table_3.DOCX]

Supplementary Material

**Clinical heterogeneities among *LRRK2* variants in Parkinson’s disease: a meta-analysis**

**Li Shu^2 †^, Yuan Zhang^2 †^, Hongxu Pan^2^, Qian Xu^2, 3,4^, Jifeng Guo^2,3,4,6,7,8^, Beisha Tang^1, 2,3,4,5,6,7,8^, Qiying Sun^1,3,4*^**

**^†^** These authors have contributed equally to this work and are co-first authors.

^*^ **Correspondence**: Qiying Sun [sunqiying2015@163.com](mailto:sunqiying2015@163.com)

**Supplementary Table 3 The results of phenotype-association analysis of each variant of *LRRK2* in different ethnic groups*.* Abbreviations: Africans, A; European/West Asians, E/W; Hispanics, H; East Asians, EA; Mixed, M; +, clinical symptoms related to a specific variant; -, clinical symptoms not related to a specific variant; NA, not available.**

| Classifications of *LRRK2* phenotypes | *LRRK2* phenotypes or rating scales | G2019S | | | | | G2385R | | | | | R1628P | | | | | R1441G | | | | |
| --- | --- | --- | --- | --- | --- | --- | --- | --- | --- | --- | --- | --- | --- | --- | --- | --- | --- | --- | --- | --- | --- |
|  |  | A | E/W | H | EA | M | A | E/W | H | EA | M | A | E/W | H | EA | M | A | E/W | H | EA | M |
| Demographic information |  |  |  |  |  |  |  |  |  |  |  |  |  |  |  |  |  |  |  |  |  |
|  | Asymmetrical onset | NA | NA | NA | NA | **-** | NA | NA | NA | - | NA | NA | NA | NA | NA | NA | NA | NA | NA | NA | NA |
|  | Age at onset | - | + | - | NA | **-** | NA | NA | NA | - | NA | NA | NA | NA | - | NA | NA | NA | NA | NA | NA |
|  | Early onset | NA | + | NA | NA | NA | NA | NA | NA | - | NA | NA | NA | NA | - | NA | NA | NA | NA | NA | NA |
|  | Male | - | **+** | - | NA | **+** | NA | NA | NA | - | NA | NA | NA | NA | - | NA | NA | NA | NA | NA | NA |
|  | Family history | NA | **+** | **+** | NA | **+** | NA | NA | NA | **+** | NA | NA | NA | NA | NA | NA | NA | NA | NA | NA | NA |
| First symptoms |  |  |  |  |  |  |  |  |  |  |  |  |  |  |  |  |  |  |  |  |  |
|  | FS-Bradykinesia | - | NA | NA | NA | NA | NA | NA | NA | - | NA | NA | NA | NA | NA | NA | NA | NA | NA | NA | NA |
|  | FS-Resting tremor | - | NA | NA | NA | - | NA | NA | NA | - | NA | NA | NA | NA | - | NA | NA | NA | NA | NA | NA |
|  | FS-Rigidity | NA | NA | NA | NA | NA | NA | NA | NA | - | NA | NA | NA | NA | NA | NA | NA | NA | NA | NA | NA |
|  | FS-Postural instability or Gait difficulty | NA | NA | NA | NA | NA | NA | NA | NA | - | NA | NA | NA | NA | NA | NA | NA | NA | NA | NA | NA |
|  | FS-Dystonia | - | NA | NA | NA | NA | NA | NA | NA | NA | NA | NA | NA | NA | NA | NA | NA | NA | NA | NA | NA |
|  | FS-Micrographia | NA | NA | NA | NA | NA | NA | NA | NA | NA | NA | NA | NA | NA | NA | NA | NA | NA | NA | NA | NA |
| Motor symptoms |  |  |  |  |  |  |  |  |  |  |  |  |  |  |  |  |  |  |  |  |  |
|  | Bradykinesia | NA | - | NA | NA | NA | NA | NA | NA | NA | NA | NA | NA | NA | NA | NA | NA | NA | NA | NA | NA |
|  | Resting tremor | NA | - | NA | NA | NA | NA | NA | NA | - | NA | NA | NA | NA | NA | NA | NA | NA | NA | NA | NA |
|  | Rigidity | NA | - | NA | NA | NA | NA | NA | NA | - | NA | NA | NA | NA | NA | NA | NA | NA | NA | NA | NA |
|  | Postural instability or Gait difficulty | NA | - | NA | NA | NA | NA | NA | NA | NA | NA | NA | NA | NA | NA | NA | NA | NA | NA | NA | NA |
| Motor phenotype classifications |  |  |  |  |  |  |  |  |  |  |  |  |  |  |  |  |  |  |  |  |  |
|  | T-Akinetic-rigid/PIGD | - | NA | NA | NA | **+** | NA | NA | NA | NA | NA | NA | NA | NA | NA | NA | NA | NA | NA | NA | NA |
|  | T-Mixed/Intermediate | - | NA | NA | NA | NA | NA | NA | NA | NA | NA | NA | NA | NA | NA | NA | NA | NA | NA | NA | NA |
|  | T-Tremor-dominant | - | NA | NA | NA | NA | NA | NA | NA | NA | NA | NA | NA | NA | NA | NA | NA | NA | NA | NA | NA |
| Scales evaluating disease severities |  |  |  |  |  |  |  |  |  |  |  |  |  |  |  |  |  |  |  |  |  |
|  | UPDRSⅠ | NA | NA | NA | NA | NA | NA | NA | NA | - | NA | NA | NA | NA | NA | NA | NA | NA | NA | NA | NA |
|  | UPDRSⅡ | - | - | NA | NA | NA | NA | NA | NA | - | NA | NA | NA | NA | NA | NA | NA | NA | NA | NA | NA |
|  | UPDRSⅢ | **+** | - | NA | NA | - | NA | NA | NA | - | NA | NA | NA | NA | NA | NA | NA | - | NA | NA | NA |
|  | H-Y | - | NA | NA | NA | NA | NA | NA | NA | **+** | NA | NA | NA | NA | - | NA | NA | NA | NA | NA | NA |
|  | Schwab & England | NA | NA | NA | NA | NA | NA | NA | NA | NA | NA | NA | NA | NA | NA | NA | NA | NA | NA | NA | NA |
| Motor complications |  |  |  |  |  |  |  |  |  |  |  |  |  |  |  |  |  |  |  |  |  |
|  | Dyskinesia | **+** | - | NA | NA | **+** | NA | NA | NA | - | NA | NA | NA | NA | NA | NA | NA | NA | NA | NA | NA |
|  | Motor fluctuations | - | NA | NA | NA | NA | NA | NA | NA | **+** | NA | NA | NA | NA | NA | NA | NA | NA | NA | NA | NA |
| Neuropsychiatric disturbances |  |  |  |  |  |  |  |  |  |  |  |  |  |  |  |  |  |  |  |  |  |
|  | Anxiety | NA | NA | NA | NA | NA | NA | NA | NA | NA | NA | NA | NA | NA | NA | NA | NA | NA | NA | NA | NA |
|  | Depression | NA | NA | NA | NA | NA | NA | NA | NA | - | NA | NA | NA | NA | NA | NA | NA | NA | NA | NA | NA |
|  | GDS15 | NA | NA | NA | NA | **+** | NA | NA | NA | NA | NA | NA | NA | NA | NA | NA | NA | NA | NA | NA | NA |
|  | Hallucination | NA | NA | NA | NA | NA | NA | NA | NA | NA | NA | NA | NA | NA | NA | NA | NA | NA | NA | NA | NA |
| Autonomic disturbances |  |  |  |  |  |  |  |  |  |  |  |  |  |  |  |  |  |  |  |  |  |
|  | SCOPA-AUT | NA | NA | NA | NA | NA | NA | NA | NA | NA | NA | NA | NA | NA | NA | NA | NA | NA | NA | NA | NA |
| Cognitive impairments |  |  |  |  |  |  |  |  |  |  |  |  |  |  |  |  |  |  |  |  |  |
|  | Cognitive impairments | - | NA | NA | NA | NA | NA | NA | NA | NA | NA | NA | NA | NA | NA | NA | NA | NA | NA | NA | NA |
|  | MMSE | - | NA | NA | NA | NA | NA | NA | NA | **+** | NA | NA | NA | NA | NA | NA | NA | NA | NA | NA | NA |
|  | MoCA | NA | NA | NA | NA | - | NA | NA | NA | NA | NA | NA | NA | NA | NA | NA | NA | NA | NA | NA | NA |
| Sleep disturbances |  |  |  |  |  |  |  |  |  |  |  |  |  |  |  |  |  |  |  |  |  |
|  | Sleep disturbances | NA | NA | NA | NA | NA | NA | NA | NA | NA | NA | NA | NA | NA | NA | NA | NA | NA | NA | NA | NA |
| Sensory complaints |  |  |  |  |  |  |  |  |  |  |  |  |  |  |  |  |  |  |  |  |  |
|  | Olfactory disturbances | NA | NA | NA | NA | - | NA | NA | NA | NA | NA | NA | NA | NA | NA | NA | NA | NA | NA | NA | NA |
|  | UPSIT scores | NA | NA | NA | NA | NA | NA | NA | NA | NA | NA | NA | NA | NA | NA | NA | NA | NA | NA | NA | NA |
| Treatments |  |  |  |  |  |  |  |  |  |  |  |  |  |  |  |  |  |  |  |  |  |
|  | Good response to l-dopa | NA | NA | NA | NA | **+** | NA | NA | NA | NA | NA | NA | NA | NA | NA | NA | NA | NA | NA | NA | NA |
|  | LEDD | **+** | **+** | NA | NA | **+** | NA | NA | NA | - | NA | NA | NA | NA | NA | NA | NA | NA | NA | NA | NA |
| Environmental factors |  |  |  |  |  |  |  |  |  |  |  |  |  |  |  |  |  |  |  |  |  |
|  | Smoke | NA | NA | NA | NA | **+** | NA | NA | NA | NA | NA | NA | NA | NA | NA | NA | NA | NA | NA | NA | NA |
