# Supplementary material for: Clinical Heterogeneity Among LRRK2 Variants in Parkinson's Disease: A Meta-Analysis
Source: Front Aging Neurosci. 2018 Sep 19;10:283. doi: 10.3389/fnagi.2018.00283 (PMC6156433; doi:10.3389/fnagi.2018.00283)
Supplement: Supplementary file 4 [file Table_4.DOCX]

Supplementary Material

**Clinical heterogeneities among *LRRK2* variants in Parkinson’s disease: a meta-analysis**

**Li Shu^2 †^, Yuan Zhang^2 †^, Hongxu Pan^2^, Qian Xu^2, 3,4^, Jifeng Guo^2,3,4,6,7,8^, Beisha Tang^1, 2,3,4,5,6,7,8^, Qiying Sun^1,3,4*^**

**^†^** These authors have contributed equally to this work and are co-first authors.

^*^ **Correspondence**: Qiying Sun [sunqiying2015@163.com](mailto:sunqiying2015@163.com)

**Supplementary Table 4 The results of phenotype association analysis of each variants of *LRRK2* in different ethnic groups. Abbreviation: Africans, A; European/West Asians, E/W; Hispanics, H; East Asians, EA; Mixed, M; FS: first symptoms; LEDD, Levodopa equivalent daily doses; NA, not available. All results were shown as OR/MD [95%CI] and *p* values. Bold OR, 95%CI and *p* values reflected statistically significance results.**

| Classifications of *LRRK2* phenotypes | *LRRK2* phenotypes or rating scales | OR/MD [95%CI]/P | G2019S | | | | | G2385R | | | | | R1628P | | | | | R1441G | | | | |
| --- | --- | --- | --- | --- | --- | --- | --- | --- | --- | --- | --- | --- | --- | --- | --- | --- | --- | --- | --- | --- | --- | --- |
|  |  |  | A | E/W | H | EA | M | A | E/W | H | EA | M | A | E/W | H | EA | M | A | E/W | H | EA | M |
| Demographic information |  |  |  |  |  |  |  |  |  |  |  |  |  |  |  |  |  |  |  |  |  |  |
|  | Asymmetrical onset | OR [95%CI] | NA | NA | NA | NA | 1.08 [0.87, 1.33] | NA | NA | NA | 0.29 [0.02, 4.33] | NA | NA | NA | NA | NA | NA | NA | NA | NA | NA | NA |
|  |  | P | NA | NA | NA | NA | 0.51 | NA | NA | NA | 0.37 | NA | NA | NA | NA | NA | NA | NA | NA | NA | NA | NA |
|  | Age at onset | MD [95%CI] | 1.22 [-1.32, 3.76] | **-2.44 [-3.93, -0.96]** | -1.78 [-12.61, 9.05] | NA | -0.52 [-2.08, 1.03] | NA | NA | NA | -0.35 [-1.79, 1.09] | NA | NA | NA | NA | -1.11 [-3.30, 1.07] | NA | NA | NA | NA | NA | NA |
|  |  | P | 0.35 | **0.001** | 0.75 | NA | 0.51 | NA | NA | NA | 0.64 | NA | NA | NA | NA | 0.32 | NA | NA | NA | NA | NA | NA |
|  | Early onset | OR [95%CI] | NA | **1.48 [1.09, 2.01]** | NA | NA | NA | NA | NA | NA | 1.14 [0.89, 1.46] | NA | NA | NA | NA | 0.95 [0.49, 1.83] | NA | NA | NA | NA | NA | NA |
|  |  | P | NA | **0.01** | NA | NA | NA | NA | NA | NA | 0.31 | NA | NA | NA | NA | 0.87 | NA | NA | NA | NA | NA | NA |
|  | Male | OR [95%CI] | 0.93 [0.75, 1.16] | **0.63 [0.51, 0.78]** | 0.82 [0.37, 1.80] | NA | **0.77 [0.69, 0.86]** | NA | NA | NA | 0.97 [0.68, 1.38] | NA | NA | NA | NA | 1.54 [0.99, 2.38] | NA | NA | NA | NA | NA | NA |
|  |  | P | 0.54 | **<0.0001** | 0.62 | NA | **<0.00001** | NA | NA | NA | 0.88 | NA | NA | NA | NA | 0.06 | NA | NA | NA | NA | NA | NA |
|  | Family history | OR [95%CI] | NA | **2.98 [2.36, 3.76]** | **4.66 [2.02, 10.73]** | NA | **2.22 [1.79, 2.75]** | NA | NA | NA | **2.10 [1.22, 3.59]** | NA | NA | NA | NA | NA | NA | NA | NA | NA | NA | NA |
|  |  | P | NA | **<0.00001** | **0.0003** | NA | **<0.00001** | NA | NA | NA | **0.007** | NA | NA | NA | NA | NA | NA | NA | NA | NA | NA | NA |
| First symptoms |  |  |  |  |  |  |  |  |  |  |  |  |  |  |  |  |  |  |  |  |  |  |
|  | FS-Bradykinesia | OR [95%CI] | 1.46 [0.77, 2.78] | NA | NA | NA | NA | NA | NA | NA | 0.92 [0.45, 1.85] | NA | NA | NA | NA | NA | NA | NA | NA | NA | NA | NA |
|  |  | P | 0.25 | NA | NA | NA | NA | NA | NA | NA | 0.8 | NA | NA | NA | NA | NA | NA | NA | NA | NA | NA | NA |
|  | FS-Resting tremor | OR [95%CI] | 0.68 [0.37, 1.26] | NA | NA | NA | 0.92 [0.36, 2.38] | NA | NA | NA | 1.12 [0.81, 1.54] | NA | NA | NA | NA | 0.85 [0.60, 1.21] | NA | NA | NA | NA | NA | NA |
|  |  | P | 0.22 | NA | NA | NA | 0.86 | NA | NA | NA | 0.51 | NA | NA | NA | NA | 0.36 | NA | NA | NA | NA | NA | NA |
|  | FS-Rigidity | OR [95%CI] | NA | NA | NA | NA | NA | NA | NA | NA | 1.16 [0.71, 1.89] | NA | NA | NA | NA | NA | NA | NA | NA | NA | NA | NA |
|  |  | P | NA | NA | NA | NA | NA | NA | NA | NA | 0.55 | NA | NA | NA | NA | NA | NA | NA | NA | NA | NA | NA |
|  | FS-Postural instability or Gait difficulty | OR [95%CI] | NA | NA | NA | NA | NA | NA | NA | NA | 1.00 [0.40, 2.48] | NA | NA | NA | NA | NA | NA | NA | NA | NA | NA | NA |
|  |  | P | NA | NA | NA | NA | NA | NA | NA | NA | 1 | NA | NA | NA | NA | NA | NA | NA | NA | NA | NA | NA |
|  | FS-Dystonia | OR [95%CI] | 1.65 [0.77, 3.55] | NA | NA | NA | NA | NA | NA | NA | NA | NA | NA | NA | NA | NA | NA | NA | NA | NA | NA | NA |
|  |  | P | 0.2 | NA | NA | NA | NA | NA | NA | NA | NA | NA | NA | NA | NA | NA | NA | NA | NA | NA | NA | NA |
|  | FS-Micrographia | OR [95%CI] | NA | NA | NA | NA | NA | NA | NA | NA | NA | NA | NA | NA | NA | NA | NA | NA | NA | NA | NA | NA |
|  |  | P | NA | NA | NA | NA | NA | NA | NA | NA | NA | NA | NA | NA | NA | NA | NA | NA | NA | NA | NA | NA |
| Motor symptoms |  |  |  |  |  |  |  |  |  |  |  |  |  |  |  |  |  |  |  |  |  |  |
|  | Bradykinesia | OR [95%CI] | NA | 0.85 [0.41, 1.76] | NA | NA | NA | NA | NA | NA | NA | NA | NA | NA | NA | NA | NA | NA | NA | NA | NA | NA |
|  |  | P | NA | 0.66 | NA | NA | NA | NA | NA | NA | NA | NA | NA | NA | NA | NA | NA | NA | NA | NA | NA | NA |
|  | Resting tremor | OR [95%CI] | NA | 1.30 [0.80, 2.13] | NA | NA | NA | NA | NA | NA | 1.38 [0.68, 2.82] | NA | NA | NA | NA | NA | NA | NA | NA | NA | NA | NA |
|  |  | P | NA | 0.29 | NA | NA | NA | NA | NA | NA | 0.37 | NA | NA | NA | NA | NA | NA | NA | NA | NA | NA | NA |
|  | Rigidity | OR [95%CI] | NA | 1.12 [0.67, 1.89] | NA | NA | NA | NA | NA | NA | 1.53 [0.50, 4.68] | NA | NA | NA | NA | NA | NA | NA | NA | NA | NA | NA |
|  |  | P | NA | 0.66 | NA | NA | NA | NA | NA | NA | 0.45 | NA | NA | NA | NA | NA | NA | NA | NA | NA | NA | NA |
|  | Postural instability or Gait difficulty | OR [95%CI] | NA | 1.43 [0.92, 2.23] | NA | NA | NA | NA | NA | NA | NA | NA | NA | NA | NA | NA | NA | NA | NA | NA | NA | NA |
|  |  | P | NA | 0.11 | NA | NA | NA | NA | NA | NA | NA | NA | NA | NA | NA | NA | NA | NA | NA | NA | NA | NA |
| Motor phenotype classifications |  |  |  |  |  |  |  |  |  |  |  |  |  |  |  |  |  |  |  |  |  |  |
|  | T-Akinetic-rigid/PIGD | OR [95%CI] | 0.84 [0.63, 1.12] | NA | NA | NA | **1.85 [1.30, 2.64]** | NA | NA | NA | NA | NA | NA | NA | NA | NA | NA | NA | NA | NA | NA | NA |
|  |  | P | 0.24 | NA | NA | NA | **0.0007** | NA | NA | NA | NA | NA | NA | NA | NA | NA | NA | NA | NA | NA | NA | NA |
|  | T-Mixed/Intermediate | OR [95%CI] | 1.24 [0.89, 1.73] | NA | NA | NA | NA | NA | NA | NA | NA | NA | NA | NA | NA | NA | NA | NA | NA | NA | NA | NA |
|  |  | P | 0.21 | NA | NA | NA | NA | NA | NA | NA | NA | NA | NA | NA | NA | NA | NA | NA | NA | NA | NA | NA |
|  | T-Tremor-dominant | OR [95%CI] | 0.84 [0.49, 1.44] | NA | NA | NA | NA | NA | NA | NA | NA | NA | NA | NA | NA | NA | NA | NA | NA | NA | NA | NA |
|  |  | P | 0.53 | NA | NA | NA | NA | NA | NA | NA | NA | NA | NA | NA | NA | NA | NA | NA | NA | NA | NA | NA |
| Scales evaluating disease severities |  |  |  |  |  |  |  |  |  |  |  |  |  |  |  |  |  | NA | NA | NA | NA | NA |
|  | UPDRSⅠ | MD [95%CI] | NA | NA | NA | NA | NA | NA | NA | NA | -0.16 [-0.51, 0.19] | NA | NA | NA | NA | NA | NA | NA | NA | NA | NA | NA |
|  |  | P | NA | NA | NA | NA | NA | NA | NA | NA | 0.38 | NA | NA | NA | NA | NA | NA | NA | NA | NA | NA | NA |
|  | UPDRSⅡ | MD [95%CI] | 0.10 [-0.03, 0.22] | 0.20 [-2.00, 2.40] | NA | NA | NA | NA | NA | NA | -1.03 [-2.91, 0.85] | NA | NA | NA | NA | NA | NA | NA | NA | NA | NA | NA |
|  |  | P | 0.13 | 0.86 | NA | NA | NA | NA | NA | NA | 0.28 | NA | NA | NA | NA | NA | NA | NA | NA | NA | NA | NA |
|  | UPDRSⅢ | MD [95%CI] | **4.79 [2.08, 7.50]** | 0.17 [-1.94, 2.29] | NA | NA | -0.31 [-2.24, 1.62] | NA | NA | NA | -1.18 [-2.48, 0.11] | NA | NA | NA | NA | NA | NA | NA | -1.77 [-5.28, 1.75] | NA | NA | NA |
|  |  | P | **0.0005** | 0.87 | NA | NA | 0.76 | NA | NA | NA | 0.07 | NA | NA | NA | NA | NA | NA | NA | 0.32 | NA | NA | NA |
|  | H-Y | MD [95%CI] | -0.23 [-0.62, 0.17] | NA | NA | NA | NA | NA | NA | NA | -0.13 [-0.24, -0.02] | NA | NA | NA | NA | 0.12 [-0.25, 0.49] | NA | NA | NA | NA | NA | NA |
|  |  | P | 0.27 | NA | NA | NA | NA | NA | NA | NA | 0.02 | NA | NA | NA | NA | 0.52 | NA | NA | NA | NA | NA | NA |
|  | Schwab & England | MD [95%CI] | NA | NA | NA | NA | NA | NA | NA | NA | NA | NA | NA | NA | NA | NA | NA | NA | NA | NA | NA | NA |
|  |  | P | NA | NA | NA | NA | NA | NA | NA | NA | NA | NA | NA | NA | NA | NA | NA | NA | NA | NA | NA | NA |
| Motor complications |  |  |  |  |  |  |  |  |  |  |  |  |  |  |  |  |  | NA | NA | NA | NA | NA |
|  | Dyskinesia | OR [95%CI] | **2.61 [1.63, 4.17]** | 1.44 [0.89, 2.35] | NA | NA | **2.37 [1.84, 3.05]** | NA | NA | NA | 1.00 [0.61, 1.66] | NA | NA | NA | NA | NA | NA | NA | NA | NA | NA | NA |
|  |  | P | **<0.0001** | 0.14 | NA | NA | **<0.00001** | NA | NA | NA | 0.99 | NA | NA | NA | NA | NA | NA | NA | NA | NA | NA | NA |
|  | Motor fluctuations | OR [95%CI] | 1.65 [0.90, 3.01] | NA | NA | NA | NA | NA | NA | NA | **3.84 [2.30, 6.41]** | NA | NA | NA | NA | NA | NA | NA | NA | NA | NA | NA |
|  |  | P | 0.1 | NA | NA | NA | NA | NA | NA | NA | **<0.00001** | NA | NA | NA | NA | NA | NA | NA | NA | NA | NA | NA |
| Neuropsychiatric disturbances |  |  |  |  |  |  |  |  |  |  |  |  |  |  |  |  |  |  |  |  |  |  |
|  | Anxiety | OR [95%CI] | NA | NA | NA | NA | NA | NA | NA | NA | NA | NA | NA | NA | NA | NA | NA | NA | NA | NA | NA | NA |
|  |  | P | NA | NA | NA | NA | NA | NA | NA | NA | NA | NA | NA | NA | NA | NA | NA | NA | NA | NA | NA | NA |
|  | Depression | OR [95%CI] | NA | NA | NA | NA | NA | NA | NA | NA | 1.42 [0.88, 2.29] | NA | NA | NA | NA | NA | NA | NA | NA | NA | NA | NA |
|  |  | P | NA | NA | NA | NA | NA | NA | NA | NA | 0.15 | NA | NA | NA | NA | NA | NA | NA | NA | NA | NA | NA |
|  | GDS15 | MD [95%CI] | NA | NA | NA | NA | **0.44 [0.09, 0.79]** | NA | NA | NA | NA | NA | NA | NA | NA | NA | NA | NA | NA | NA | NA | NA |
|  |  | P | NA | NA | NA | NA | **0.01** | NA | NA | NA | NA | NA | NA | NA | NA | NA | NA | NA | NA | NA | NA | NA |
|  | Hallucination | OR [95%CI] | NA | NA | NA | NA | NA | NA | NA | NA | NA | NA | NA | NA | NA | NA | NA | NA | NA | NA | NA | NA |
|  |  | P | NA | NA | NA | NA | NA | NA | NA | NA | NA | NA | NA | NA | NA | NA | NA | NA | NA | NA | NA | NA |
| Autonomic disturbances |  |  |  |  |  |  |  |  |  |  |  |  |  |  |  |  |  |  |  |  |  |  |
|  | SCOPA-AUT | MD [95%CI] | NA | NA | NA | NA | NA | NA | NA | NA | NA | NA | NA | NA | NA | NA | NA | NA | NA | NA | NA | NA |
|  |  | P | NA | NA | NA | NA | NA | NA | NA | NA | NA | NA | NA | NA | NA | NA | NA | NA | NA | NA | NA | NA |
| Cognitive impairments |  |  |  |  |  |  |  |  |  |  |  |  |  |  |  |  |  |  |  |  |  |  |
|  | Cognitive impairments | OR [95%CI] | 1.59 [0.97, 2.63] | NA | NA | NA | NA | NA | NA | NA | NA | NA | NA | NA | NA | NA | NA | NA | NA | NA | NA | NA |
|  |  | P | 0.07 | NA | NA | NA | NA | NA | NA | NA | NA | NA | NA | NA | NA | NA | NA | NA | NA | NA | NA | NA |
|  | MMSE | MD [95%CI] | -0.33 [-1.19, 0.52] | NA | NA | NA | NA | NA | NA | NA | **1.02 [0.43, 1.62]** | NA | NA | NA | NA | NA | NA | NA | NA | NA | NA | NA |
|  |  | P | 0.45 | NA | NA | NA | NA | NA | NA | NA | **0.0007** | NA | NA | NA | NA | NA | NA | NA | NA | NA | NA | NA |
|  | MoCA | MD [95%CI] | NA | NA | NA | NA | 0.12 [-0.17, 0.40] | NA | NA | NA | NA | NA | NA | NA | NA | NA | NA | NA | NA | NA | NA | NA |
|  |  | P | NA | NA | NA | NA | 0.42 | NA | NA | NA | NA | NA | NA | NA | NA | NA | NA | NA | NA | NA | NA | NA |
| Sleep disturbances |  |  |  |  |  |  |  |  |  |  |  |  |  |  |  |  |  |  |  |  |  |  |
|  | Sleep disturbances | OR [95%CI] | NA | NA | NA | NA | NA | NA | NA | NA | NA | NA | NA | NA | NA | NA | NA | NA | NA | NA | NA | NA |
|  |  | P | NA | NA | NA | NA | NA | NA | NA | NA | NA | NA | NA | NA | NA | NA | NA | NA | NA | NA | NA | NA |
| Sensory complaints |  |  |  |  |  |  |  |  |  |  |  |  |  |  |  |  |  |  |  |  |  |  |
|  | Olfactory disturbances | OR [95%CI] | NA | NA | NA | NA | 0.97 [0.25, 3.75] | NA | NA | NA | NA | NA | NA | NA | NA | NA | NA | NA | NA | NA | NA | NA |
|  |  | P | NA | NA | NA | NA | 0.96 | NA | NA | NA | NA | NA | NA | NA | NA | NA | NA | NA | NA | NA | NA | NA |
|  | UPSIT scores | MD [95%CI] | NA | NA | NA | NA | NA | NA | NA | NA | NA | NA | NA | NA | NA | NA | NA | NA | NA | NA | NA | NA |
|  |  | P | NA | NA | NA | NA | NA | NA | NA | NA | NA | NA | NA | NA | NA | NA | NA | NA | NA | NA | NA | NA |
| Treatments |  |  |  |  |  |  |  |  |  |  |  |  |  |  |  |  |  |  |  |  |  |  |
|  | Good response to l-dopa | OR [95%CI] | NA | NA | NA | NA | **2.80 [1.72, 4.57]** | NA | NA | NA | NA | NA | NA | NA | NA | NA | NA | NA | NA | NA | NA | NA |
|  |  | P | NA | NA | NA | NA | **<0.0001** | NA | NA | NA | NA | NA | NA | NA | NA | NA | NA | NA | NA | NA | NA | NA |
|  | LEDD | MD [95%CI] | 67.16 [-5.33, 139.65] | **102.43 [15.14, 189.71]** | NA | NA | **129.87 [93.04, 166.69]** | NA | NA | NA | 32.47 [-22.84, 87.78] | NA | NA | NA | NA | NA | NA | NA | NA | NA | NA | NA |
|  |  | P | 0.07 | **0.02** | NA | NA | **<0.00001** | NA | NA | NA | 0.25 | NA | NA | NA | NA | NA | NA | NA | NA | NA | NA | NA |
| Environmental factors |  |  |  |  |  |  |  |  |  |  |  |  |  |  |  |  |  |  |  |  |  |  |
|  | Smoke | OR [95%CI] | NA | NA | NA | NA | **1.57 [1.24, 1.99]** | NA | NA | NA | NA | NA | NA | NA | NA | NA | NA | NA | NA | NA | NA | NA |
|  |  | P | NA | NA | NA | NA | **0.0002** | NA | NA | NA | NA | NA | NA | NA | NA | NA | NA | NA | NA | NA | NA | NA |
