# Supplementary material for: Clinical Heterogeneity Among LRRK2 Variants in Parkinson's Disease: A Meta-Analysis
Source: Front Aging Neurosci. 2018 Sep 19;10:283. doi: 10.3389/fnagi.2018.00283 (PMC6156433; doi:10.3389/fnagi.2018.00283)
Supplement: Supplementary file 5 [file Image_1.PDF]

frontiers

Supplementary Material

Clinical heterogeneities among *LRRK2* variants in Parkinson's disease: a meta-analysis

Li Shu[2] †, Yuan Zhang[2] †, Hongxu Pan[2], Qian Xu[2, 3,4], Jifeng Guo[2,3,4,6,7,8], Beisha Tang[1, 2,3,4,5,6,7,8], Qiying Sun[1,3,4*]

† These authors have contributed equally to this work and are co-first authors.

* **Correspondence**: Qiying Sun sunqiying2015@163.com

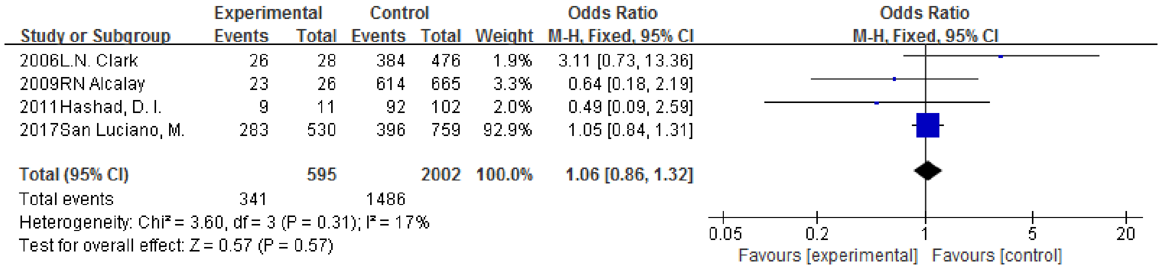

1.1 Asymmetrical onset of G2019S

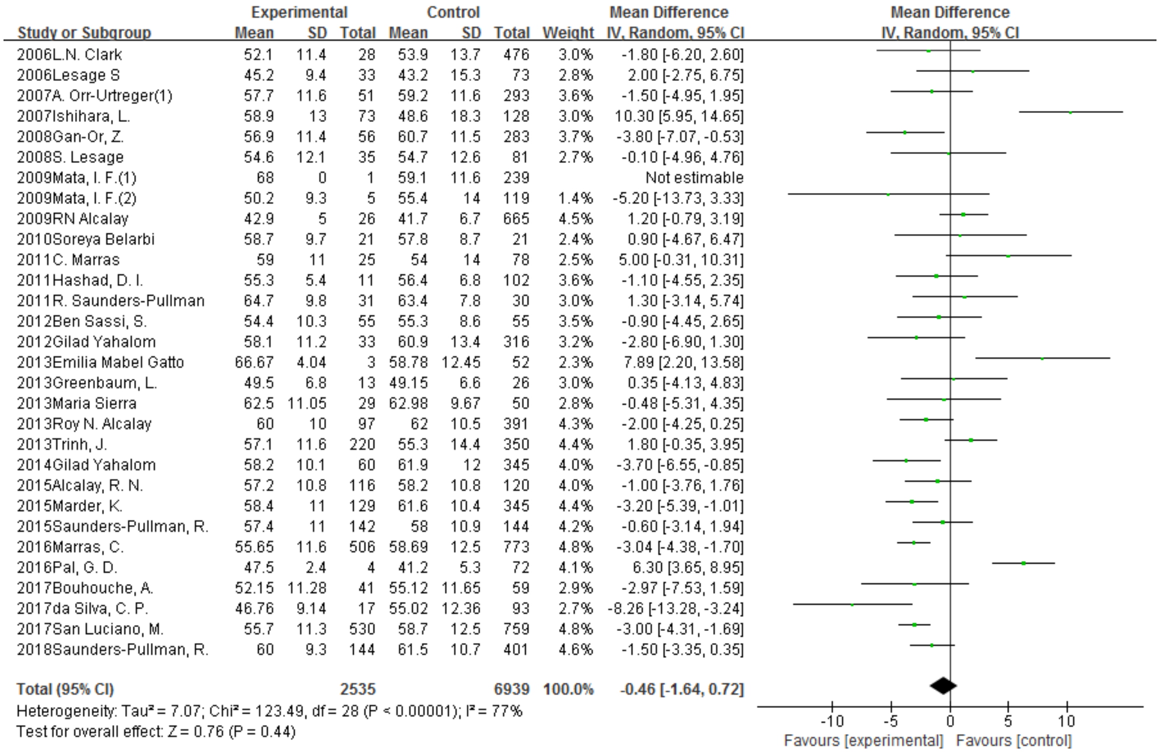

1.2 Age at onset of G2019S

Study or Subgroup	Experimental Events	Experimental Total	Control Events	Control Total	Weight	Odds Ratio M-H, Fixed, 95% CI
2006DENISE M. KAY	7	20	412	1498	8.1%	1.42 [0.56, 3.58]
2006Goldwurm, S.	8	19	379	1072	8.8%	1.33 [0.53, 3.33]
2006L.N. Clark	12	28	233	476	17.0%	0.78 [0.36, 1.69]
2007A. Orr-Urtreger(1)	14	51	71	293	17.6%	1.18 [0.61, 2.31]
2007A. Orr-Urtreger(2)	3	7	26	121	1.9%	2.74 [0.58, 13.02]
2008Gan-Or, Z.	15	56	45	283	12.5%	1.93 [0.99, 3.79]
2010Gan-Or, Z.	17	75	58	402	16.3%	1.74 [0.95, 3.19]
2011Hashad, D. I.	2	11	10	102	1.8%	2.04 [0.39, 10.81]
2014Gilad Yahalom	11	60	57	345	15.9%	1.13 [0.56, 2.31]
**Total (95% CI)**		**327**		**4592**	**100.0%**	**1.37 [1.05, 1.79]**
Total events	89		1291			

Heterogeneity: Chi² = 5.09, df = 8 (P = 0.75); I² = 0%
Test for overall effect: Z = 2.29 (P = 0.02)

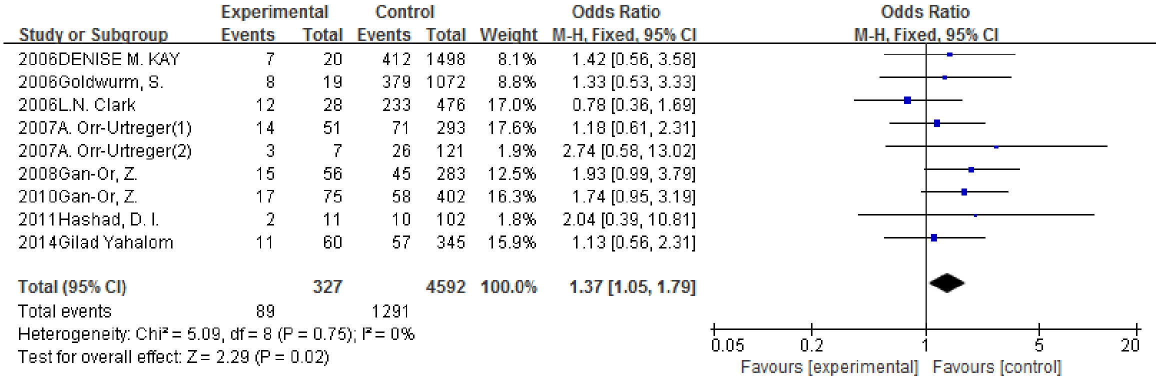

1.3 EOPD of G2019S

Study or Subgroup	Experimental Events	Experimental Total	Control Events	Control Total	Weight	Odds Ratio M-H, Fixed, 95% CI
2006DENISE M. KAY	13	20	1010	1498	0.9%	0.90 [0.36, 2.26]
2006L.N. Clark	11	28	293	476	1.8%	0.40 [0.19, 0.88]
2006Lesage S	13	33	41	73	1.4%	0.51 [0.22, 1.17]
2007A. Orr-Urtreger(1)	25	51	191	293	2.6%	0.51 [0.28, 0.94]
2007A. Orr-Urtreger(2)	5	7	82	121	0.2%	1.19 [0.22, 6.40]
2008Gan-Or, Z.	28	56	187	283	2.8%	0.51 [0.29, 0.92]
2008Latourelle, J. C.	32	60	549	965	2.7%	0.87 [0.51, 1.46]
2008Mary M Hulihan	39	72	77	166	1.9%	1.37 [0.78, 2.38]
2008S. Lesage	21	47	53	89	1.8%	0.55 [0.27, 1.12]
2009Mata, I. F.(1)	1	1	121	239	0.0%	2.93 [0.12, 72.54]
2009Mata, I. F.(2)	3	5	58	119	0.2%	1.58 [0.25, 9.78]
2009RN Alcalay	14	26	411	665	1.3%	0.72 [0.33, 1.58]
2010Gan-Or, Z.	37	75	262	402	3.8%	0.52 [0.32, 0.86]
2010Soreya Belarbi	11	23	34	48	1.0%	0.38 [0.14, 1.06]
2010Vicki Shanker	10	21	10	21	0.5%	1.00 [0.30, 3.36]
2011C. Marras	12	25	49	84	1.1%	0.66 [0.27, 1.62]
2011R. Saunders-Pullman	15	31	17	30	0.8%	0.72 [0.26, 1.97]
2012Ben Sassi, S.	34	55	30	55	1.0%	1.35 [0.63, 2.89]
2013Emilia Mabel Gatto	2	3	23	52	0.1%	2.52 [0.22, 29.58]
2013Greenbaum, L.	8	13	17	26	0.4%	0.85 [0.21, 3.36]
2013Maria Sierra	16	29	29	50	0.9%	0.89 [0.35, 2.24]
2013Mirelman, A.	27	50	24	50	1.0%	1.27 [0.58, 2.79]
2013Roy N. Alcalay	47	97	243	391	4.5%	0.57 [0.37, 0.90]
2013Trinh, J.	124	220	187	350	5.7%	1.13 [0.80, 1.58]
2014Carles Gaig	15	33	15	33	0.7%	1.00 [0.38, 2.64]
2014Gilad Yahalom	30	60	211	345	2.9%	0.64 [0.37, 1.10]
2014Rachel Saunders-Pullman	70	126	78	126	3.2%	0.77 [0.47, 1.27]
2015Alcalay, R. N.	64	116	76	120	3.1%	0.71 [0.42, 1.20]
2015Marder, K.	64	129	215	345	5.4%	0.60 [0.40, 0.90]
2015Nabli, F.	32	58	29	54	1.2%	1.06 [0.50, 2.23]
2015Saunders-Pullman, R.	77	142	93	144	3.9%	0.65 [0.40, 1.04]
2015Somme, J. H.	6	12	18	27	0.5%	0.50 [0.13, 2.00]
2015Vilas, D.	12	26	21	31	0.9%	0.41 [0.14, 1.20]
2016Dagan, E.	9	22	114	189	1.3%	0.46 [0.19, 1.12]
2016Marras, C.	266	516	465	790	16.2%	0.74 [0.59, 0.93]
2017Bouhouche, A.	19	41	37	59	1.5%	0.51 [0.23, 1.15]
2017da Silva, C. P.	7	17	57	93	0.9%	0.44 [0.15, 1.27]
2017San Luciano, M.	276	530	448	759	16.1%	0.75 [0.60, 0.94]
2018Saunders-Pullman, R.	72	144	144	401	3.5%	1.78 [1.21, 2.62]
**Total (95% CI)**		**3020**		**10062**	**100.0%**	**0.77 [0.70, 0.84]**
Total events	1567		6019			

Heterogeneity: Chi² = 56.77, df = 38 (P = 0.03); I² = 33%
Test for overall effect: Z = 5.77 (P < 0.00001)

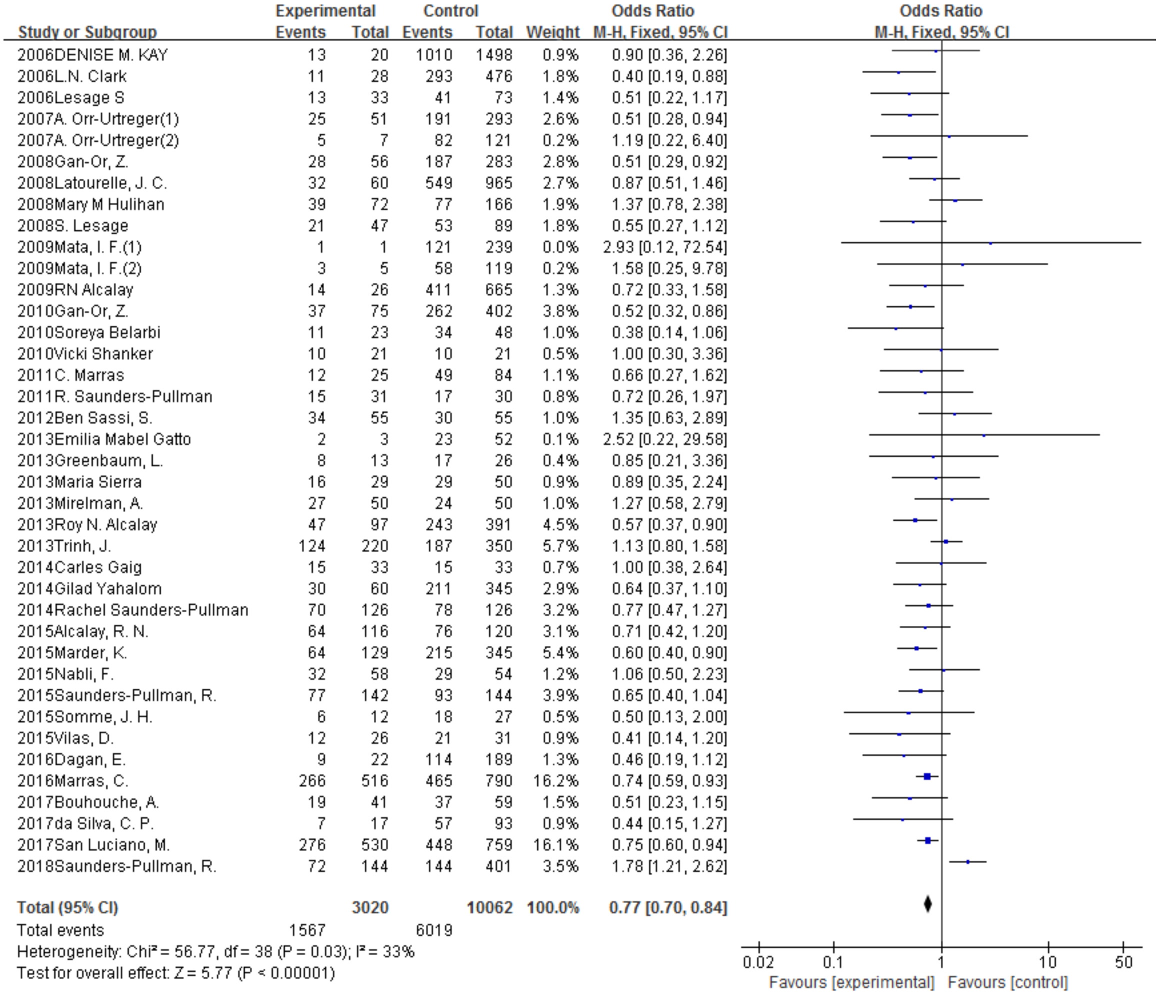

1.4 Gender-male of G2019S

Study or Subgroup	Experimental Events	Experimental Total	Control Events	Control Total	Weight	Odds Ratio M-H, Fixed, 95% CI
2005Jose Miguel Bras	6	11	20	117	0.8%	5.82 [1.62, 20.95]
2006DENISE M. KAY	11	20	344	1498	2.1%	4.10 [1.69, 9.98]
2006Gaig, C.	6	13	85	286	2.1%	2.03 [0.66, 6.21]
2006Goldwurm, S.	10	19	226	1073	2.0%	4.16 [1.67, 10.37]
2006L.N. Clark	7	26	54	453	2.2%	2.72 [1.09, 6.78]
2007A. Orr-Urtreger(1)	25	51	71	293	5.6%	3.01 [1.63, 5.54]
2007A. Orr-Urtreger(2)	3	7	32	121	1.0%	2.09 [0.44, 9.83]
2008Gan-Or, Z.	24	56	64	283	6.3%	2.57 [1.41, 4.67]
2008Latourelle, J. C.	54	60	845	965	5.2%	1.28 [0.54, 3.04]
2009Mata, I. F.(1)	0	1	4	239	0.0%	17.44 [0.62, 489.20]
2009Mata, I. F.(2)	1	5	28	119	0.9%	0.81 [0.09, 7.57]
2009RN Alcalay	5	26	99	665	3.2%	1.36 [0.50, 3.69]
2010Gan-Or, Z.	34	75	85	402	7.7%	3.09 [1.85, 5.17]
2012Gilad Yahalom	16	33	70	316	3.6%	3.31 [1.59, 6.88]
2013Emilia Mabel Gatto	3	3	13	50	0.1%	19.44 [0.94, 401.55]
2013Roy N. Alcalay	34	97	69	391	9.3%	2.52 [1.54, 4.12]
2014Gilad Yahalom	26	60	73	345	6.4%	2.85 [1.61, 5.05]
2016Dagan, E.	8	21	32	177	2.2%	2.79 [1.07, 7.28]
2017Bouhouche, A.	22	41	11	59	2.2%	5.05 [2.06, 12.40]
2017da Silva, C. P.	9	17	15	93	1.1%	5.85 [1.95, 17.59]
2017San Luciano, M.	151	530	116	759	35.8%	2.21 [1.68, 2.90]
**Total (95% CI)**		**1172**		**8704**	**100.0%**	**2.62 [2.25, 3.06]**
Total events	455		2356			

Heterogeneity: Chi² = 18.74, df = 20 (P = 0.54); I² = 0%

Test for overall effect: Z = 12.38 (P < 0.00001)

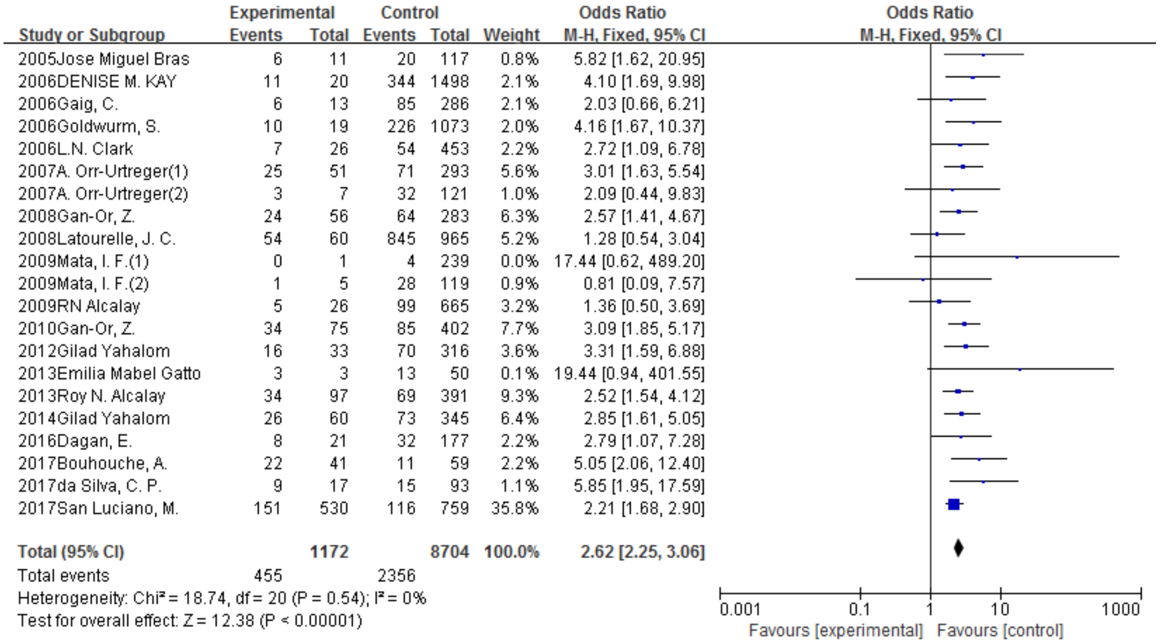

1.5 Family history of G2019S

Study or Subgroup	Experimental Events	Experimental Total	Control Events	Control Total	Weight	Odds Ratio M-H, Random, 95% CI
2006L.N. Clark	1	28	25	476	8.1%	0.67 [0.09, 5.12]
2006Lesage S	17	31	34	63	20.8%	1.04 [0.44, 2.46]
2008S. Lesage	8	33	16	71	19.0%	1.10 [0.42, 2.91]
2010Gan-Or, Z.	8	75	61	402	22.1%	0.67 [0.31, 1.46]
2011C. Marras	0	25	14	84	4.7%	0.10 [0.01, 1.66]
2011Hashad, D. I.	11	11	102	102		Not estimable
2017Bouhouche, A.	14	41	9	59	19.2%	2.88 [1.10, 7.52]
2017da Silva, C. P.	2	17	1	90	6.0%	11.87 [1.01, 139.18]
**Total (95% CI)**		**261**		**1347**	**100.0%**	**1.16 [0.59, 2.26]**
Total events	61		262			

Heterogeneity: Tau² = 0.37; Chi² = 12.31, df = 6 (P = 0.06); I² = 51%

Test for overall effect: Z = 0.42 (P = 0.67)

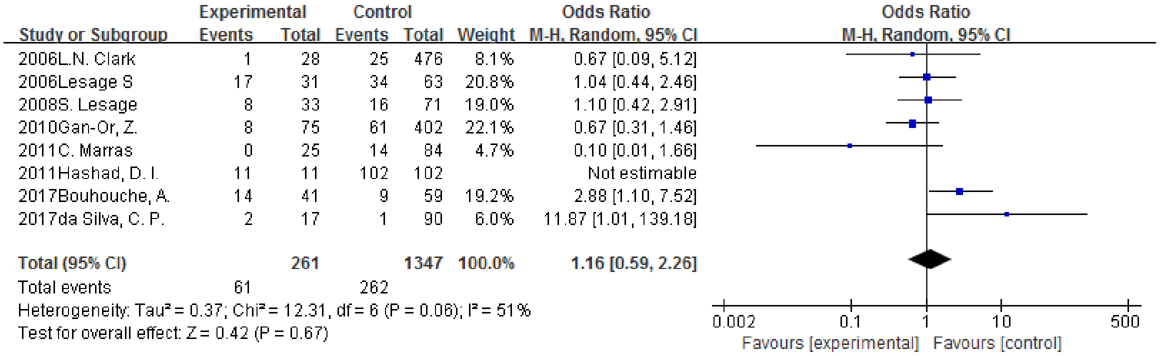

1.6 FS-bradykinesia of G2019S

Study or Subgroup	Experimental Events	Experimental Total	Control Events	Control Total	Weight	Odds Ratio M-H, Random, 95% CI
2006L.N. Clark	9	28	238	476	9.3%	0.47 [0.21, 1.07]
2006Lesage S	20	29	39	63	8.1%	1.37 [0.54, 3.49]
2008Mary M Hulihan	56	72	155	166	9.2%	0.25 [0.11, 0.57]
2008S. Lesage	31	33	62	73	4.2%	2.75 [0.57, 13.18]
2010Gan-Or, Z.	37	75	236	402	13.0%	0.68 [0.42, 1.12]
2011C. Marras	16	25	31	84	8.2%	3.04 [1.20, 7.70]
2011Hashad, D. I.	9	11	87	102	4.0%	0.78 [0.15, 3.95]
2013Roy N. Alcalay	42	97	214	391	13.6%	0.63 [0.40, 0.99]
2013Trinh, J.	158	220	265	350	14.4%	0.82 [0.56, 1.20]
2017Bouhouche, A.	11	41	31	59	8.9%	0.33 [0.14, 0.78]
2017da Silva, C. P.	9	17	66	90	7.1%	0.41 [0.14, 1.18]
**Total (95% CI)**		**648**		**2256**	**100.0%**	**0.70 [0.49, 1.02]**
Total events	398		1424			

Heterogeneity: Tau² = 0.21; Chi² = 26.19, df = 10 (P = 0.003); I² = 62%

Test for overall effect: Z = 1.86 (P = 0.06)

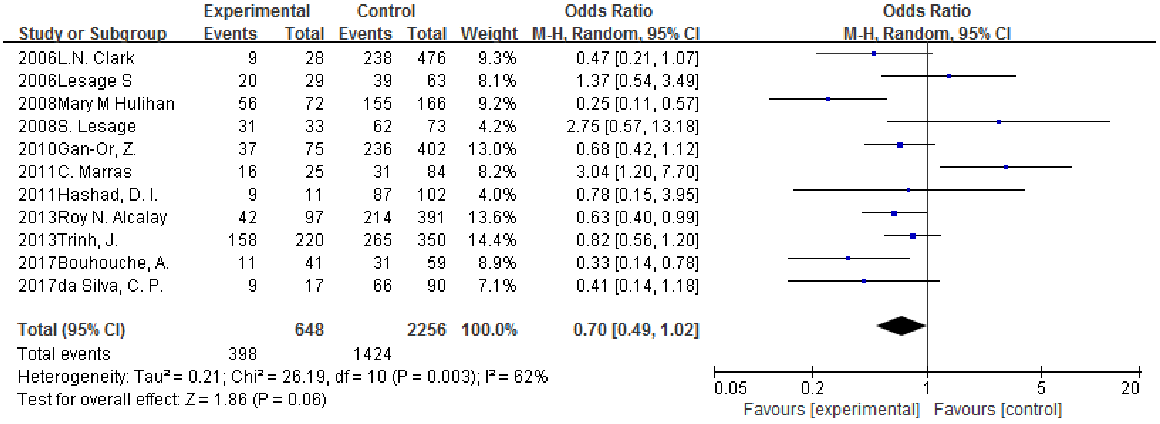

1.7 FS-resting tremor of G2019S

Study or Subgroup	Experimental Events	Experimental Total	Control Events	Control Total	Weight	Odds Ratio M-H, Fixed, 95% CI
2006L.N. Clark	3	28	40	476	10.3%	1.31 [0.38, 4.52]
2010Gan-Or, Z.	29	75	105	402	52.6%	1.78 [1.07, 2.99]
2011C. Marras	1	25	5	84	5.7%	0.66 [0.07, 5.91]
2011Hashad, D. I.	4	11	39	102	12.6%	0.92 [0.25, 3.36]
2013Trinh, J.	5	220	8	350	15.7%	0.99 [0.32, 3.08]
2017da Silva, C. P.	1	17	4	90	3.1%	1.34 [0.14, 12.82]
**Total (95% CI)**		**376**		**1504**	**100.0%**	**1.42 [0.96, 2.12]**
Total events	43		201			

Heterogeneity: Chi² = 2.05, df = 5 (P = 0.84); I² = 0%
Test for overall effect: Z = 1.74 (P = 0.08)

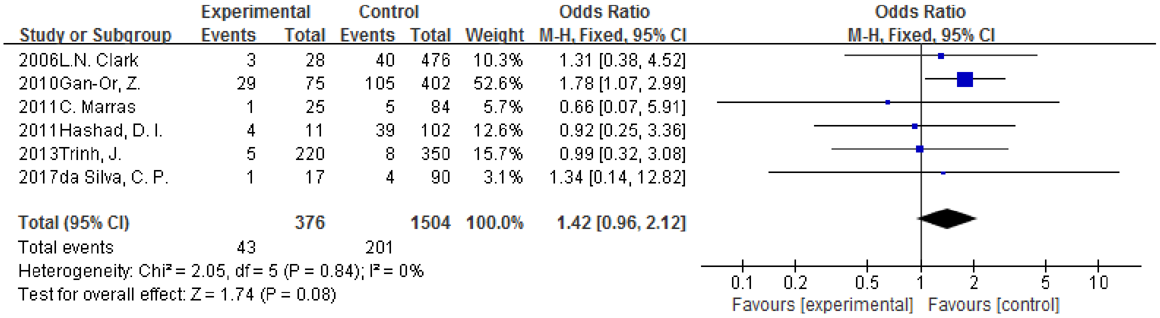

1.8 FS-rigidity of G2019S

Study or Subgroup	Experimental Events	Experimental Total	Control Events	Control Total	Weight	Odds Ratio M-H, Fixed, 95% CI
2006L.N. Clark	1	28	24	476	4.9%	0.70 [0.09, 5.35]
2008Mary M Hulihan	6	72	21	166	22.2%	0.63 [0.24, 1.63]
2010Gan-Or, Z.	14	75	62	402	30.2%	1.26 [0.66, 2.39]
2011C. Marras	1	25	11	84	9.2%	0.28 [0.03, 2.25]
2013Trinh, J.	27	220	26	350	33.5%	1.74 [0.99, 3.07]
**Total (95% CI)**		**420**		**1478**	**100.0%**	**1.16 [0.81, 1.67]**
Total events	49		144			

Heterogeneity: Chi² = 5.67, df = 4 (P = 0.23); I² = 29%
Test for overall effect: Z = 0.81 (P = 0.42)

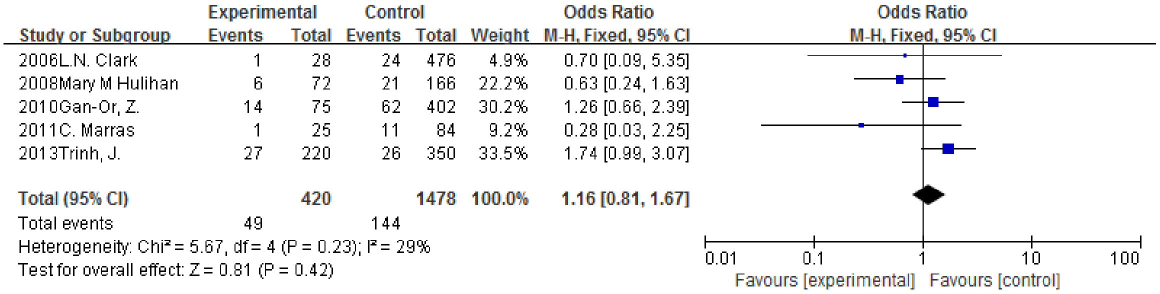

1.9 FS-Postural instability or Gait difficulty of G2019S

Study or Subgroup	Experimental Events	Experimental Total	Control Events	Control Total	Weight	Odds Ratio M-H, Random, 95% CI
2006Lesage S	2	28	7	62	21.7%	0.60 [0.12, 3.11]
2008Mary M Hulihan	1	72	1	166	7.5%	2.32 [0.14, 37.68]
2008S. Lesage	4	32	2	71	19.0%	4.93 [0.85, 28.45]
2013Trinh, J.	7	220	7	350	51.7%	1.61 [0.56, 4.66]
**Total (95% CI)**		**352**		**649**	**100.0%**	**1.65 [0.77, 3.55]**
Total events	14		17			

Heterogeneity: Tau² = 0.00; Chi² = 3.00, df = 3 (P = 0.39); I² = 0%
Test for overall effect: Z = 1.29 (P = 0.20)

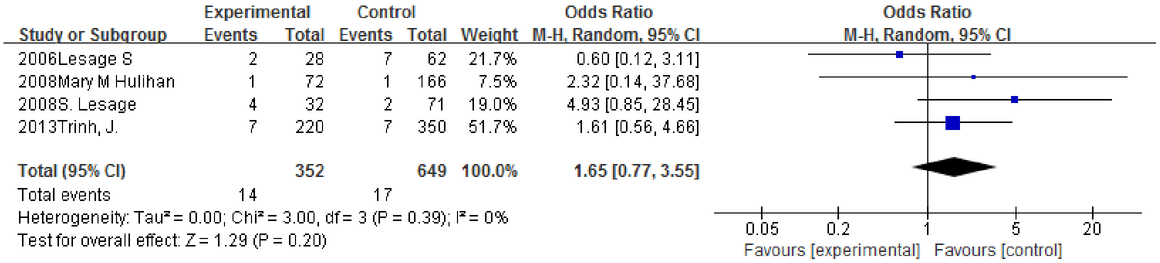

1.10 FS-Dystonia of G2019S

Study or Subgroup	Experimental Events	Experimental Total	Control Events	Control Total	Weight	Odds Ratio M-H, Random, 95% CI
2006Lesage S	4	30	13	62	43.8%	0.58 [0.17, 1.96]
2008S. Lesage	1	32	1	64	30.3%	2.03 [0.12, 33.59]
2011C. Marras	1	25	3	3	25.8%	0.01 [0.00, 0.26]
**Total (95% CI)**		**87**		**129**	**100.0%**	**0.29 [0.02, 3.53]**
Total events	6		17			

Heterogeneity: Tau² = 3.35; Chi² = 6.51, df = 2 (P = 0.04); I² = 69%
Test for overall effect: Z = 0.97 (P = 0.33)

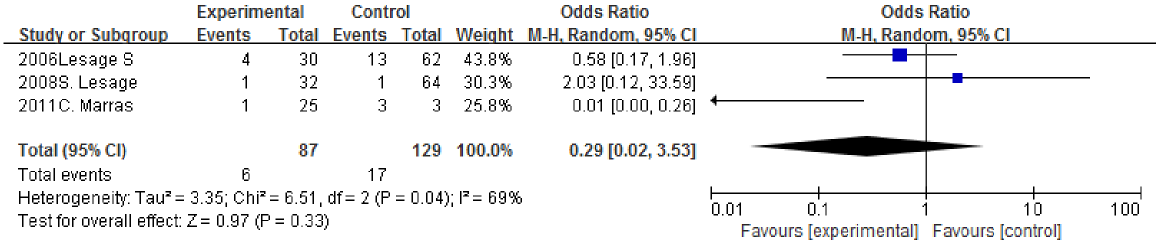

1.11 FS-Micrographia of G2019S

Study or Subgroup	Experimental Events	Experimental Total	Control Events	Control Total	Weight	Odds Ratio M-H, Fixed, 95% CI
2006L.N. Clark	21	28	379	476	36.7%	0.77 [0.32, 1.86]
2006Lesage S	31	31	56	61	2.1%	6.13 [0.33, 114.59]
2007A. Orr-Urtreger(1)	9	51	60	293	51.1%	0.83 [0.38, 1.80]
2007A. Orr-Urtreger(2)	1	7	17	121	5.6%	1.02 [0.12, 9.00]
2008S. Lesage	33	34	69	73	4.5%	1.91 [0.21, 17.80]
2014Carles Gaig	33	33	33	33		Not estimable
2017da Silva, C. P.	17	17	93	93		Not estimable
**Total (95% CI)**		**201**		**1150**	**100.0%**	**0.98 [0.58, 1.65]**
Total events	145		707			

Heterogeneity: Chi² = 2.32, df = 4 (P = 0.68); I² = 0%
Test for overall effect: Z = 0.08 (P = 0.94)

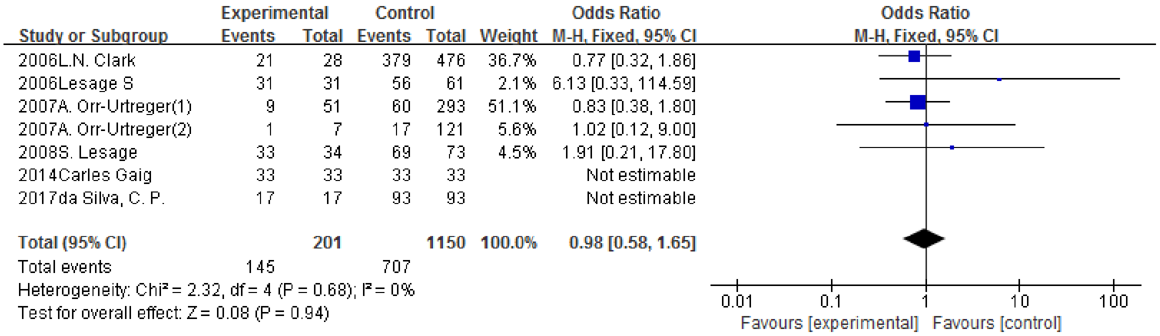

1.12 Bradykinesia of G2019S

Study or Subgroup	Experimental Events	Experimental Total	Control Events	Control Total	Weight	Odds Ratio M-H, Fixed, 95% CI
2006L.N. Clark	19	28	356	476	19.3%	0.71 [0.31, 1.62]
2006Lesage S	28	32	50	61	6.5%	1.54 [0.45, 5.29]
2007A. Orr-Urtreger(1)	32	51	164	293	27.5%	1.32 [0.72, 2.44]
2007A. Orr-Urtreger(2)	3	7	70	121	6.6%	0.55 [0.12, 2.55]
2008S. Lesage	31	33	62	69	3.7%	1.75 [0.34, 8.93]
2009RN Alcalay	11	26	271	665	17.9%	1.07 [0.48, 2.36]
2013Emilia Mabel Gatto	2	3	30	48	1.8%	1.20 [0.10, 14.19]
2014Carles Gaig	29	33	27	33	5.0%	1.61 [0.41, 6.34]
2016Dagan, E.	19	21	146	176	4.5%	1.95 [0.43, 8.83]
2017da Silva, C. P.	14	17	85	92	7.1%	0.38 [0.09, 1.66]
**Total (95% CI)**		**251**		**2034**	**100.0%**	**1.11 [0.80, 1.55]**
Total events	188		1261			

Heterogeneity: Chi² = 5.68, df = 9 (P = 0.77); I² = 0%
Test for overall effect: Z = 0.62 (P = 0.54)

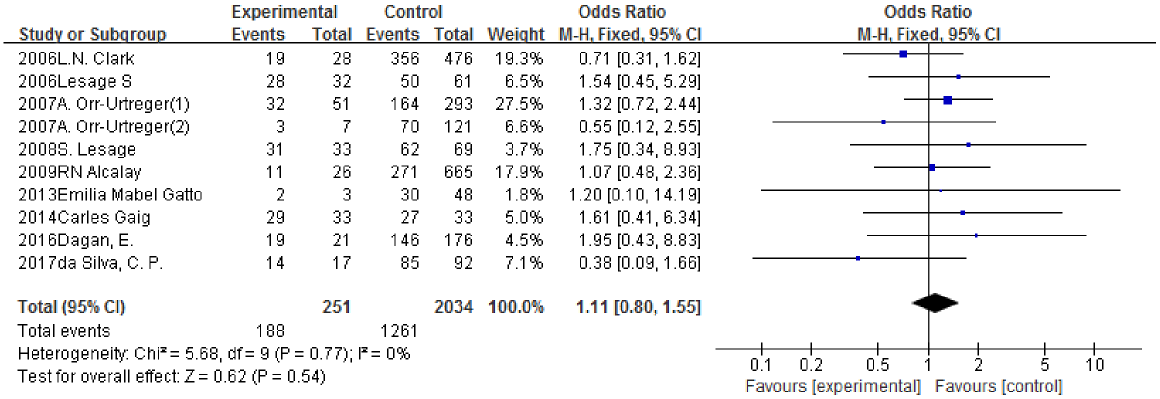

1.13 Resting tremor of G2019S

Study or Subgroup	Experimental Events	Experimental Total	Control Events	Control Total	Weight	Odds Ratio M-H, Fixed, 95% CI
2006L.N. Clark	27	28	463	476	5.7%	0.76 [0.10, 6.01]
2006Lesage S	31	31	55	62	1.8%	8.51 [0.47, 154.09]
2007A. Orr-Urtreger(1)	16	51	81	293	51.1%	1.20 [0.63, 2.28]
2007A. Orr-Urtreger(2)	4	7	38	121	5.5%	2.91 [0.62, 13.66]
2008S. Lesage	33	34	69	72	4.0%	1.43 [0.14, 14.32]
2016Dagan, E.	15	21	150	187	26.8%	0.62 [0.22, 1.70]
2017da Silva, C. P.	16	17	90	93	5.1%	0.53 [0.05, 5.45]
**Total (95% CI)**		**189**		**1304**	**100.0%**	**1.22 [0.77, 1.94]**
Total events	142		946			

Heterogeneity: Chi² = 5.40, df = 6 (P = 0.49); I² = 0%
Test for overall effect: Z = 0.83 (P = 0.40)

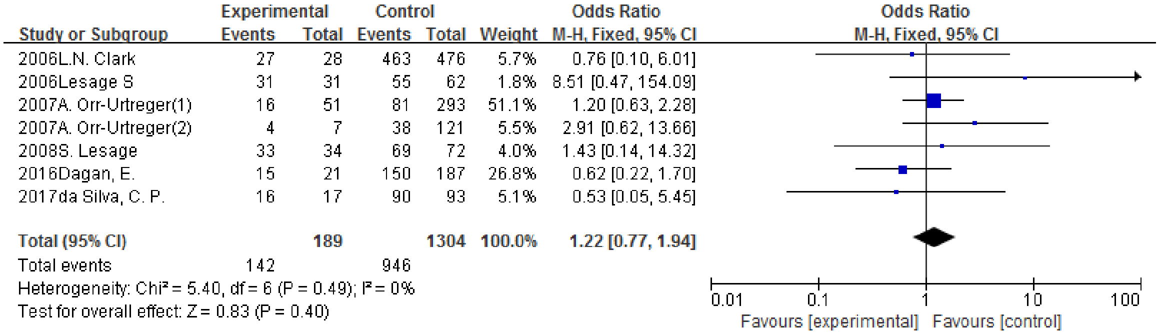

1.14 Rigidity of G2019S

Study or Subgroup	Experimental Events	Experimental Total	Control Events	Control Total	Weight	Odds Ratio M-H, Fixed, 95% CI
2006L.N. Clark	16	28	234	476	18.2%	1.38 [0.64, 2.98]
2007A. Orr-Urtreger(1)	15	51	80	293	27.4%	1.11 [0.58, 2.14]
2007A. Orr-Urtreger(2)	3	7	31	121	3.2%	2.18 [0.46, 10.28]
2014Carles Gaig	16	33	11	33	9.3%	1.88 [0.70, 5.09]
2016Dagan, E.	10	21	62	173	11.5%	1.63 [0.65, 4.05]
2017Bouhouche, A.	17	41	29	59	22.8%	0.73 [0.33, 1.64]
2017da Silva, C. P.	14	17	85	92	7.7%	0.38 [0.09, 1.66]
**Total (95% CI)**		**198**		**1247**	**100.0%**	**1.18 [0.84, 1.66]**
Total events	91		532			

Heterogeneity: Chi² = 5.71, df = 6 (P = 0.46); I² = 0%
Test for overall effect: Z = 0.97 (P = 0.33)

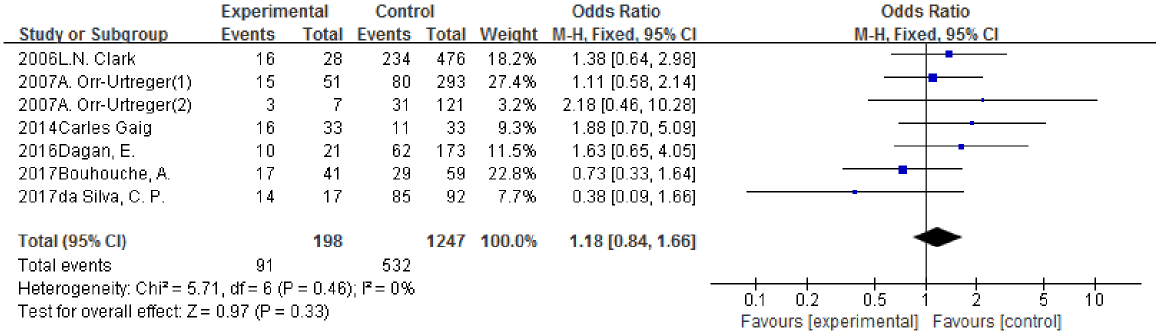

1.15 Postural instability or Gait difficulty of G2019S

Study or Subgroup	Experimental Events	Experimental Total	Control Events	Control Total	Weight	Odds Ratio M-H, Random, 95% CI
2007Ishihara, L.	7	73	25	128	8.0%	0.44 [0.18, 1.07]
2008Mary M Hulihan	10	72	27	166	9.2%	0.83 [0.38, 1.82]
2009RN Alcalay	24	26	392	665	4.2%	8.36 [1.96, 35.65]
2012Ben Sassi, S.	8	55	10	55	6.9%	0.77 [0.28, 2.11]
2013Roy N. Alcalay	51	97	164	391	13.9%	1.53 [0.98, 2.40]
2013Trinh, J.	37	220	68	350	14.0%	0.84 [0.54, 1.30]
2015Alcalay, R. N.	73	111	60	118	12.6%	1.86 [1.09, 3.16]
2015Nabli, F.	34	58	32	54	9.6%	0.97 [0.46, 2.07]
2017Bouhouche, A.	10	41	10	59	7.1%	1.58 [0.59, 4.23]
2018Saunders-Pullman, R.	97	144	218	401	14.6%	1.73 [1.16, 2.58]
**Total (95% CI)**		**897**		**2387**	**100.0%**	**1.23 [0.88, 1.73]**
Total events	351		1006			

Heterogeneity: Tau² = 0.16; Chi² = 23.11, df = 9 (P = 0.006); I² = 61%
Test for overall effect: Z = 1.22 (P = 0.22)

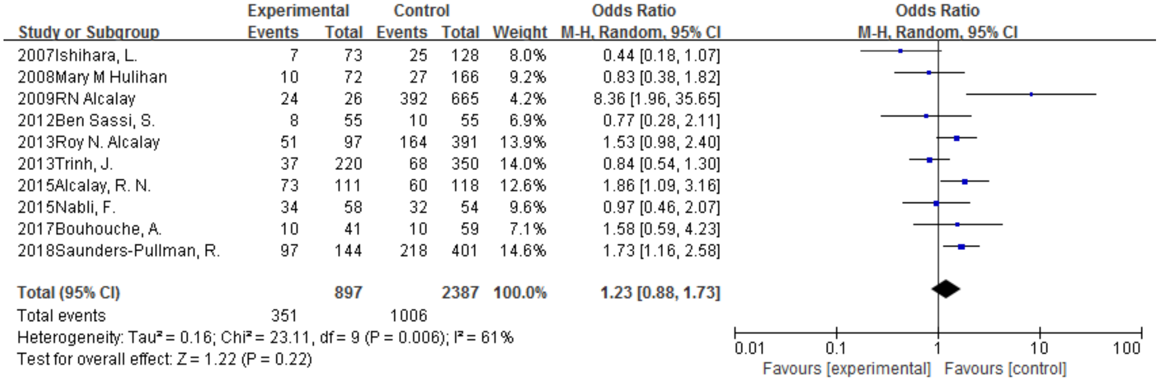

1.16 T-Akinetic-rigid/PIGD of G2019S

Study or Subgroup	Experimental Events	Experimental Total	Control Events	Control Total	Weight	Odds Ratio M-H, Random, 95% CI
2007Ishihara, L.	59	73	89	128	13.5%	1.85 [0.92, 3.70]
2008Mary M Hulihan	48	72	123	166	15.2%	0.70 [0.38, 1.27]
2012Ben Sassi, S.	40	55	36	55	11.5%	1.41 [0.62, 3.17]
2013Trinh, J.	123	220	192	350	20.6%	1.04 [0.74, 1.47]
2015Nabli, F.	14	58	11	54	10.4%	1.24 [0.51, 3.04]
2017Bouhouche, A.	24	41	22	59	11.5%	2.37 [1.05, 5.36]
2018Saunders-Pullman, R.	23	144	105	401	17.3%	0.54 [0.33, 0.88]
**Total (95% CI)**		**663**		**1213**	**100.0%**	**1.09 [0.75, 1.60]**
Total events	331		578			

Heterogeneity: Tau² = 0.15; Chi² = 15.67, df = 6 (P = 0.02); I² = 62%
Test for overall effect: Z = 0.47 (P = 0.64)

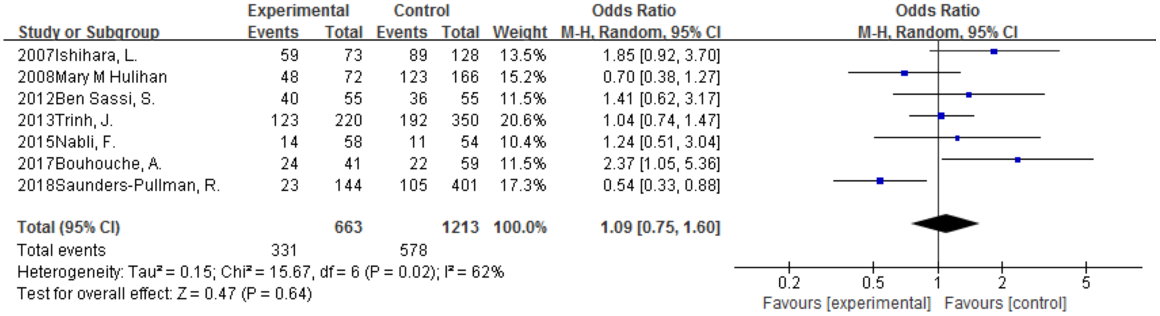

1.17 T-Mixed/Intermediate of G2019S

Study or Subgroup	Experimental Events	Experimental Total	Control Events	Control Total	Weight	Odds Ratio M-H, Random, 95% CI
2007Ishihara, L.	7	73	14	128	11.5%	0.86 [0.33, 2.25]
2008Mary M Hulihan	14	72	16	166	13.9%	2.26 [1.04, 4.93]
2009RN Alcalay	1	26	182	665	4.2%	0.11 [0.01, 0.79]
2012Ben Sassi, S.	7	55	9	55	10.2%	0.75 [0.26, 2.17]
2013Trinh, J.	37	220	62	350	19.1%	0.94 [0.60, 1.47]
2015Nabli, F.	10	58	11	54	11.6%	0.81 [0.31, 2.11]
2017Bouhouche, A.	7	41	27	59	11.5%	0.24 [0.09, 0.64]
2018Saunders-Pullman, R.	22	144	73	401	18.0%	0.81 [0.48, 1.36]
**Total (95% CI)**		**689**		**1878**	**100.0%**	**0.77 [0.49, 1.21]**
Total events	105		394			

Heterogeneity: Tau² = 0.23; Chi² = 17.26, df = 7 (P = 0.02); I² = 59%
Test for overall effect: Z = 1.13 (P = 0.26)

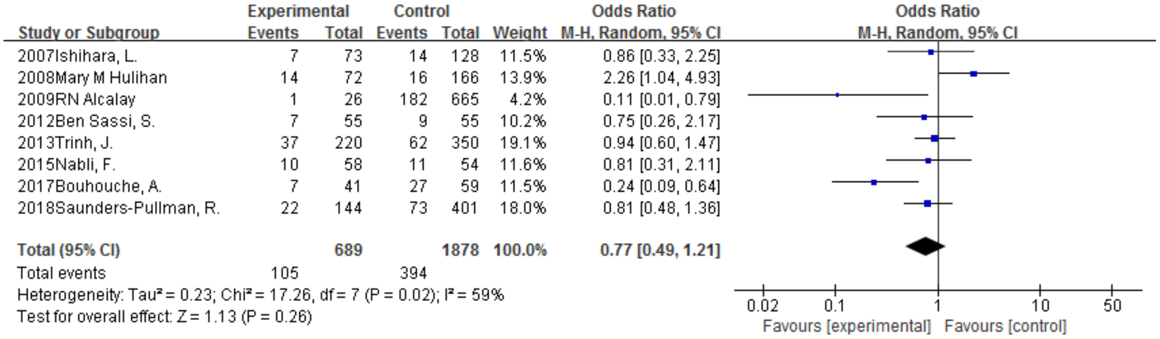

1.18 T-Tremor-dominant of G2019S

Study or Subgroup	Experimental Mean	Experimental SD	Experimental Total	Control Mean	Control SD	Control Total	Weight	Mean Difference IV, Random, 95% CI
2011Hashad, D. I.	6.8	1.9	11	7.6	2.3	102	22.0%	-0.80 [-2.01, 0.41]
2013Beatriz Tijero	2	0.89	6	2.08	1.76	13	22.2%	-0.08 [-1.27, 1.11]
2015Somme, J. H.	1.9	1.9	12	4.2	2.8	27	17.5%	-2.30 [-3.81, -0.79]
2018Saunders-Pullman, R.	1.69	1.55	144	1.8	1.7	401	38.3%	-0.11 [-0.41, 0.19]
**Total (95% CI)**			**173**			**543**	**100.0%**	**-0.64 [-1.48, 0.20]**

Heterogeneity: Tau² = 0.45; Chi² = 8.76, df = 3 (P = 0.03); I² = 66%
Test for overall effect: Z = 1.49 (P = 0.14)

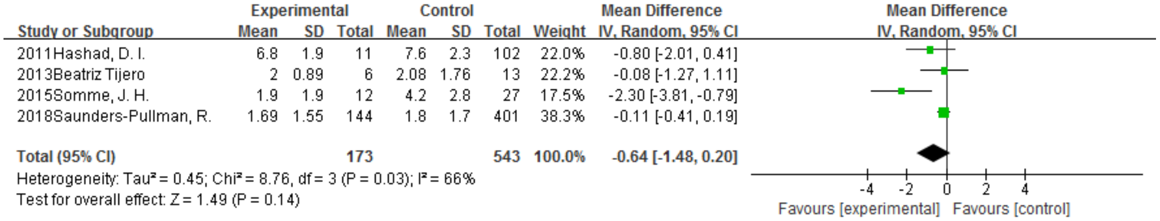

1.19 UPDRS Ⅰ of G2019S

Study or Subgroup	Experimental Mean	Experimental SD	Experimental Total	Control Mean	Control SD	Control Total	Weight	Mean Difference IV, Random, 95% CI
2010Vicki Shanker	6.4	5.8	21	8.1	4.1	21	5.0%	-1.70 [-4.74, 1.34]
2011Hashad, D. I.	24.3	6.7	11	24.2	5.8	102	3.0%	0.10 [-4.02, 4.22]
2013Beatriz Tijero	12.5	8.8	6	9.58	2.74	13	1.0%	2.92 [-4.28, 10.12]
2013Trinh, J.	1.41	0.77	220	1.31	0.72	350	29.3%	0.10 [-0.03, 0.23]
2014Carles Gaig	10.3	7.5	33	8.3	4.9	33	5.0%	2.00 [-1.06, 5.06]
2015Nabli, F.	11.17	6.71	58	12.02	5.4	54	8.0%	-0.85 [-3.10, 1.40]
2015Somme, J. H.	12.5	8.4	12	13.6	6.6	27	1.8%	-1.10 [-6.47, 4.27]
2016Marras, C.	10.18	0.75	449	11.09	0.19	684	29.5%	-0.91 [-0.98, -0.84]
2018Saunders-Pullman, R.	8.42	6.06	144	9.01	5.94	401	17.4%	-0.59 [-1.74, 0.56]
**Total (95% CI)**			**954**			**1685**	**100.0%**	**-0.38 [-1.13, 0.36]**

Heterogeneity: Tau² = 0.49; Chi² = 190.62, df = 8 (P < 0.00001); I² = 96%
Test for overall effect: Z = 1.01 (P = 0.31)

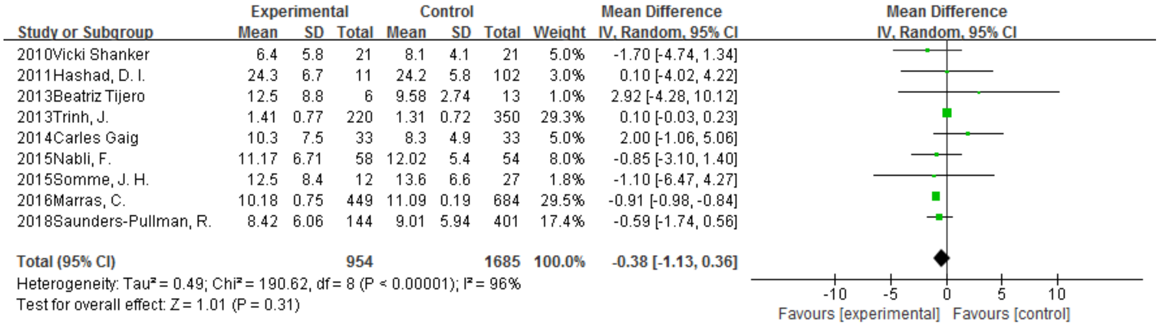

1.20 UPDRS Ⅱ of G2019S

Study or Subgroup	Experimental Mean	Experimental SD	Experimental Total	Control Mean	Control SD	Control Total	Weight	Mean Difference IV, Random, 95% CI
2006L.N. Clark	18.5	11.1	28	23	13.3	476	5.4%	-4.50 [-8.78, -0.22]
2006Lesage S	51	21	17	38	16	30	1.7%	13.00 [1.49, 24.51]
2007Ishihara, L.	48.5	22.7	42	43.8	23.3	65	2.6%	4.70 [-4.20, 13.60]
2008S. Lesage	47.2	20.3	11	30.5	24.1	14	0.9%	16.70 [-0.71, 34.11]
2009RN Alcalay	19.7	13.7	26	20.5	11.7	665	4.6%	-0.80 [-6.14, 4.54]
2010Vicki Shanker	9.5	5.6	21	11.2	5.8	21	6.1%	-1.70 [-5.15, 1.75]
2011Hashad, D. I.	33.1	4.3	11	29.1	5.9	102	6.7%	4.00 [1.21, 6.79]
2011R. Saunders-Pullman	12.3	10.9	31	13.1	9.4	30	4.7%	-0.80 [-5.90, 4.30]
2012Ben Sassi, S.	42.4	22.9	55	42.4	21.6	55	2.8%	0.00 [-8.32, 8.32]
2013Beatriz Tijero	31	18.07	6	26.54	7.48	13	1.1%	4.46 [-10.56, 19.48]
2013Maria Sierra	28.69	13.23	29	28	12.87	50	4.1%	0.69 [-5.30, 6.68]
2013Mirelman, A.	17.4	9.5	50	16.6	9.6	50	5.9%	0.80 [-2.94, 4.54]
2013Roy N. Alcalay	19.8	12.8	97	19.9	11.3	391	6.7%	-0.10 [-2.88, 2.68]
2014Carles Gaig	24	14	33	19.9	12.2	33	3.9%	4.10 [-2.24, 10.44]
2014Rachel Saunders-Pullman	22	12.4	126	19.2	11.3	126	6.6%	2.80 [-0.13, 5.73]
2015Alcalay, R. N.	21.4	12.2	116	19.5	11.1	120	6.5%	1.90 [-1.08, 4.88]
2015Nabli, F.	44.04	21.71	58	37.86	12.54	54	3.8%	6.18 [-0.33, 12.69]
2015Saunders-Pullman, R.	21.1	13.1	142	20.1	11.8	144	6.6%	1.00 [-1.89, 3.89]
2015Somme, J. H.	29.8	15.9	12	31.3	8.1	27	2.3%	-1.50 [-11.00, 8.00]
2016Marras, C.	24.55	0.45	424	27.68	1.12	652	8.2%	-3.13 [-3.23, -3.03]
2016Pal, G. D.	30.8	11.7	4	23.4	12.1	65	1.7%	7.40 [-4.44, 19.24]
2018Saunders-Pullman, R.	19.38	12.02	144	20.29	12.08	401	7.1%	-0.91 [-3.20, 1.38]
**Total (95% CI)**			**1483**			**3584**	**100.0%**	**0.99 [-0.72, 2.71]**

Heterogeneity: Tau² = 9.40; Chi² = 108.39, df = 21 (P < 0.00001); I² = 81%
Test for overall effect: Z = 1.13 (P = 0.26)

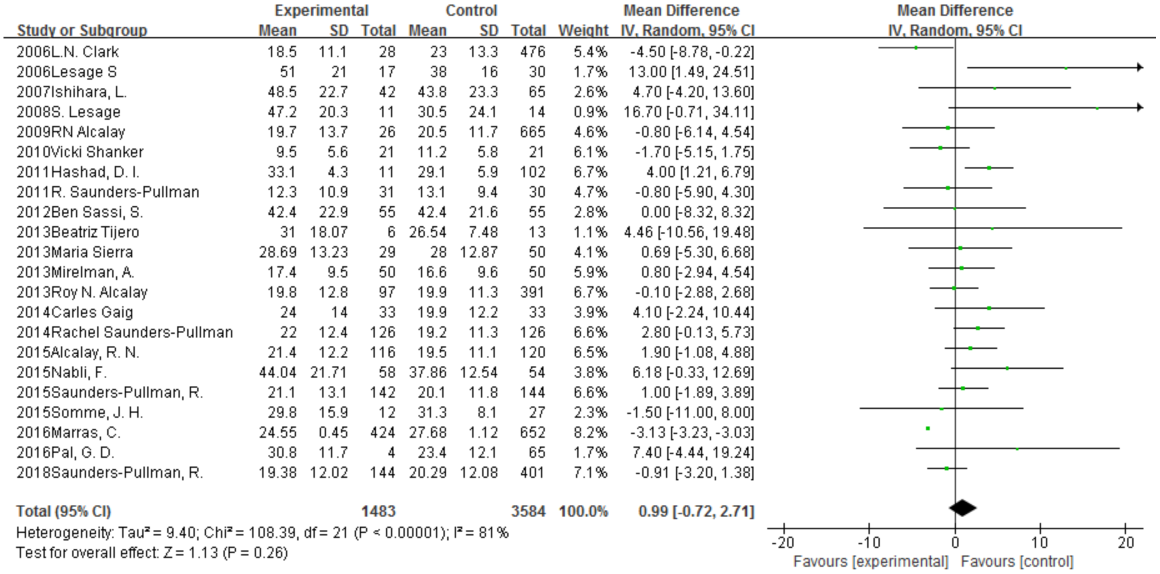

1.21 UPDRSⅢ of G2019S

Study or Subgroup	Experimental Mean	Experimental SD	Experimental Total	Control Mean	Control SD	Control Total	Weight	Mean Difference IV, Random, 95% CI
2006L.N. Clark	2.1	0.8	28	1.9	0.9	476	16.0%	0.20 [-0.11, 0.51]
2007Ishihara, L.	1.8	1	42	1.8	1	65	13.7%	0.00 [-0.39, 0.39]
2011Hashad, D. I.	2.95	0.65	11	2.8	0.54	102	13.4%	0.15 [-0.25, 0.55]
2012Ben Sassi, S.	2.3	0.9	55	2.7	1.9	55	9.8%	-0.40 [-0.96, 0.16]
2014Carles Gaig	2.1	1	33	1.8	0.8	33	12.4%	0.30 [-0.14, 0.74]
2015Nabli, F.	2.3	0.82	58	2.96	1.02	54	14.9%	-0.66 [-1.00, -0.32]
2018Saunders-Pullman, R.	2.07	0.88	144	2.11	0.84	401	19.9%	-0.04 [-0.21, 0.13]
**Total (95% CI)**			**371**			**1186**	**100.0%**	**-0.06 [-0.29, 0.18]**

Heterogeneity: Tau² = 0.06; Chi² = 19.64, df = 6 (P = 0.003); I² = 69%
Test for overall effect: Z = 0.47 (P = 0.64)

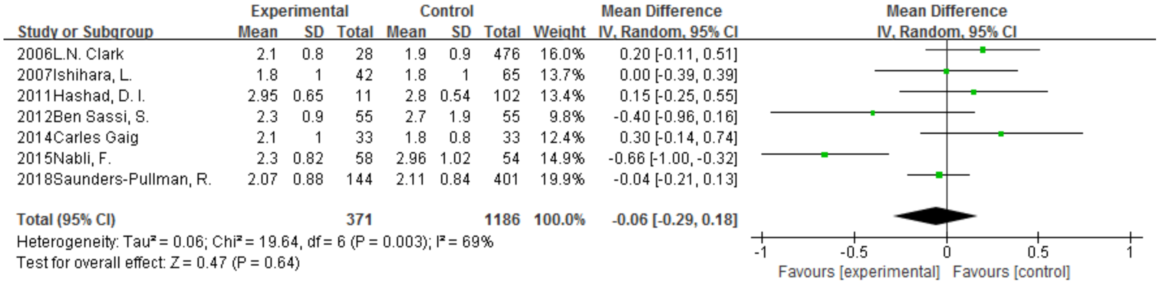

1.22 H-Y of G2019S

Study or Subgroup	Experimental Mean	Experimental SD	Experimental Total	Control Mean	Control SD	Control Total	Weight	Mean Difference IV, Fixed, 95% CI
2007Ishihara, L.	66	15.3	42	68.2	16.7	65	0.0%	-2.20 [-8.36, 3.96]
2012Ben Sassi, S.	67.8	23.8	55	66.2	23.2	55	0.0%	1.60 [-7.18, 10.38]
2014Carles Gaig	82.1	14.7	33	85.8	10.3	33	0.0%	-3.70 [-9.82, 2.42]
2016Marras, C.	86.2	0.81	432	84.71	0.13	698	99.9%	1.49 [1.41, 1.57]
2018Saunders-Pullman, R.	86.65	18.92	144	86.26	20.55	401	0.0%	0.39 [-3.30, 4.08]
**Total (95% CI)**			**706**			**1252**	**100.0%**	**1.49 [1.41, 1.57]**

Heterogeneity: Chi² = 4.48, df = 4 (P = 0.34); I² = 11%
Test for overall effect: Z = 37.90 (P < 0.00001)

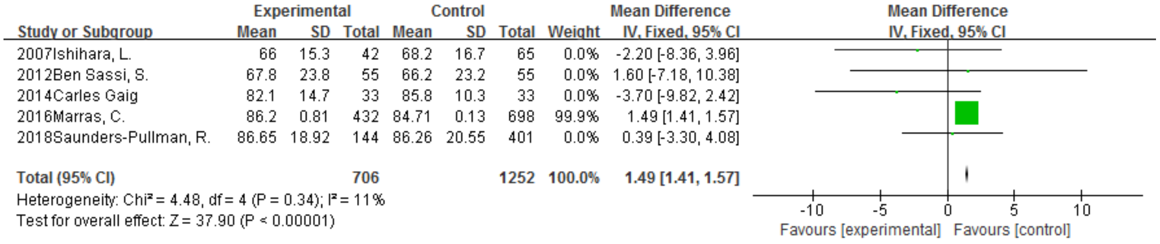

1.23 Schwab & England of G2019S

Study or Subgroup	Experimental Events	Experimental Total	Control Events	Control Total	Weight	Odds Ratio M-H, Fixed, 95% CI
2006Lesage S	19	28	27	49	4.9%	1.72 [0.65, 4.55]
2008S. Lesage	18	34	11	69	2.6%	5.93 [2.34, 15.07]
2012Gilad Yahalom	22	33	168	316	8.2%	1.76 [0.83, 3.76]
2013Mirelman, A.	15	50	14	50	7.6%	1.10 [0.46, 2.62]
2013Roy N. Alcalay	33	97	77	391	15.6%	2.10 [1.29, 3.43]
2014Carles Gaig	19	33	16	33	5.2%	1.44 [0.55, 3.81]
2015Nabli, F.	6	58	7	54	5.0%	0.77 [0.24, 2.47]
2016Marras, C.	86	242	73	419	26.5%	2.61 [1.81, 3.76]
2016Pal, G. D.	3	4	61	72	1.2%	0.54 [0.05, 5.69]
2017Bouhouche, A.	23	41	15	59	4.2%	3.75 [1.60, 8.78]
2017da Silva, C. P.	12	17	50	83	3.9%	1.58 [0.51, 4.91]
2017San Luciano, M.	41	530	26	759	15.2%	2.36 [1.43, 3.91]
**Total (95% CI)**		**1167**		**2354**	**100.0%**	**2.18 [1.79, 2.67]**
Total events	297		545			

Heterogeneity: Chi² = 15.39, df = 11 (P = 0.17); I² = 29%
Test for overall effect: Z = 7.70 (P < 0.00001)

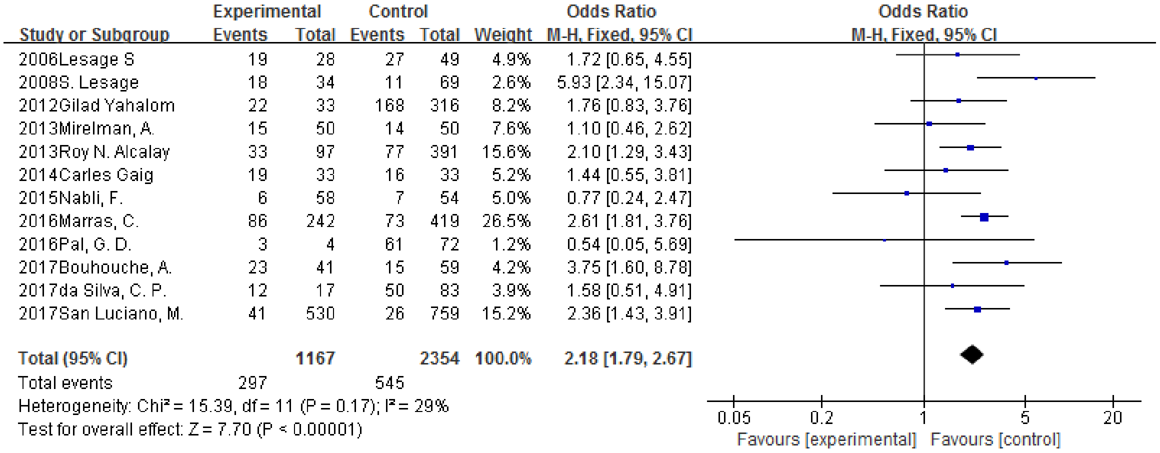

1.24 Dyskinesia of G2019S

Study or Subgroup	Experimental Events	Experimental Total	Control Events	Control Total	Weight	Odds Ratio M-H, Random, 95% CI
2008S. Lesage	18	34	19	69	16.4%	2.96 [1.26, 6.97]
2013Emilia Mabel Gatto	3	3	31	48	4.1%	3.89 [0.19, 79.71]
2014Carles Gaig	18	33	17	33	15.3%	1.13 [0.43, 2.97]
2015Nabli, F.	25	58	23	54	17.4%	1.02 [0.48, 2.16]
2016Marras, C.	86	135	95	396	20.4%	5.56 [3.65, 8.46]
2017Bouhouche, A.	25	41	29	59	16.8%	1.62 [0.72, 3.63]
2017da Silva, C. P.	15	17	51	59	9.6%	1.18 [0.23, 6.14]
**Total (95% CI)**		**321**		**718**	**100.0%**	**2.02 [1.03, 3.97]**
Total events	190		265			

Heterogeneity: Tau² = 0.54; Chi² = 23.52, df = 6 (P = 0.0006); I² = 74%
Test for overall effect: Z = 2.03 (P = 0.04)

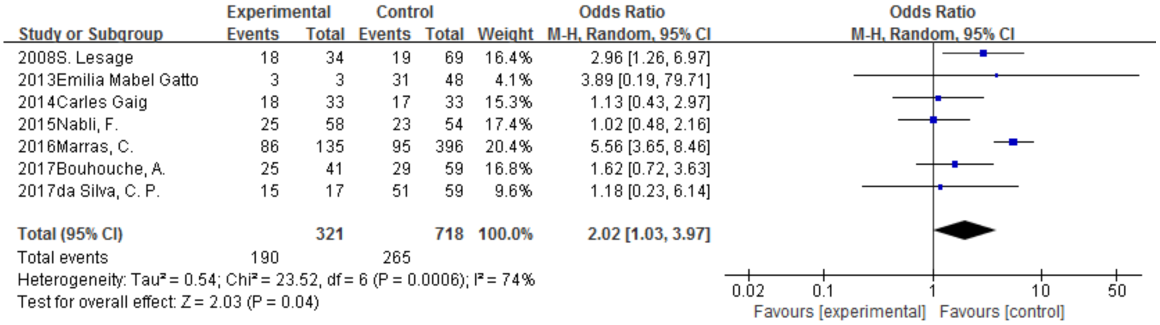

1.25 Motor fluctuations of G2019S

Study or Subgroup	Experimental Events	Experimental Total	Control Events	Control Total	Weight	Odds Ratio M-H, Fixed, 95% CI
2010Soreya Belarbi	16	23	27	48	38.5%	1.78 [0.62, 5.11]
2014Carles Gaig	8	33	9	33	49.3%	0.85 [0.28, 2.58]
2015Somme, J. H.	1	12	3	27	12.2%	0.73 [0.07, 7.80]
**Total (95% CI)**		**68**		**108**	**100.0%**	**1.19 [0.58, 2.44]**
Total events	25		39			

Heterogeneity: Chi² = 1.07, df = 2 (P = 0.59); I² = 0%
Test for overall effect: Z = 0.49 (P = 0.63)

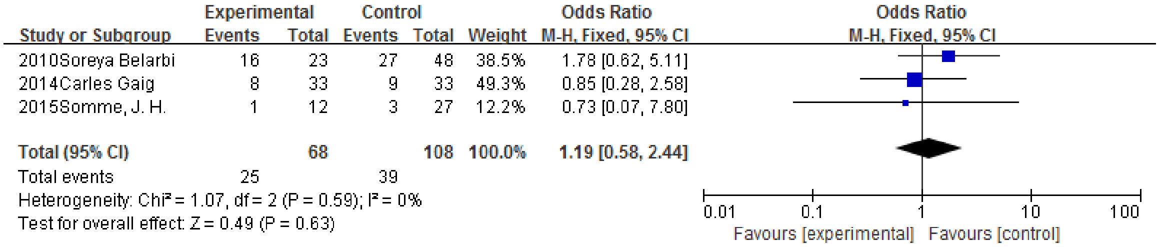

1.26 Anxiety of G2019S

Study or Subgroup	Experimental Events	Experimental Total	Control Events	Control Total	Weight	Odds Ratio M-H, Random, 95% CI
2008Pankratz, N.	1	34	34	806	8.9%	0.69 [0.09, 5.18]
2010Soreya Belarbi	15	23	19	48	19.3%	2.86 [1.02, 8.06]
2012Ben Sassi, S.	18	55	25	55	23.8%	0.58 [0.27, 1.27]
2014Carles Gaig	19	33	12	33	20.1%	2.38 [0.88, 6.39]
2015Somme, J. H.	2	12	8	27	11.0%	0.47 [0.08, 2.67]
2017da Silva, C. P.	13	17	51	90	16.9%	2.49 [0.75, 8.22]
**Total (95% CI)**		**174**		**1059**	**100.0%**	**1.33 [0.66, 2.69]**
Total events	68		149			

Heterogeneity: Tau² = 0.38; Chi² = 10.58, df = 5 (P = 0.06); I² = 53%
Test for overall effect: Z = 0.81 (P = 0.42)

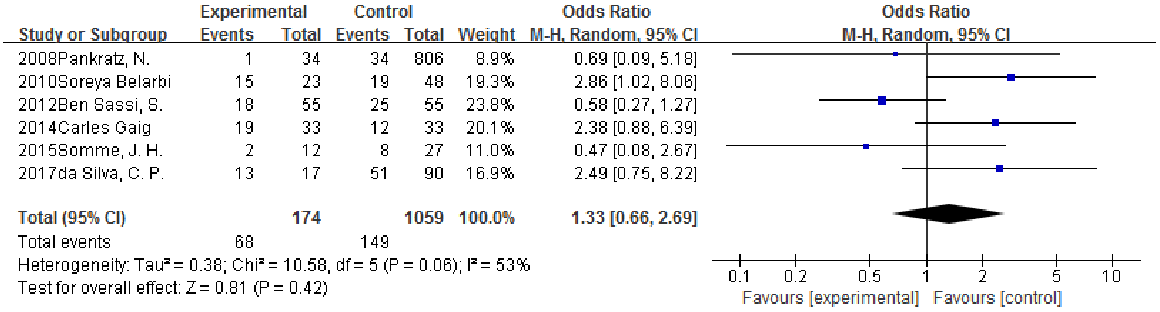

1.27 Depression of G2019S

Study or Subgroup	Experimental Mean	Experimental SD	Experimental Total	Control Mean	Control SD	Control Total	Weight	Mean Difference IV, Fixed, 95% CI
2012Ben Sassi, S.	16.8	5.9	55	20.2	10.1	55	1.2%	-3.40 [-6.49, -0.31]
2013Mirelman, A.	4.2	3.5	50	3.2	3.2	50	6.6%	1.00 [-0.31, 2.31]
2013Roy N. Alcalay	4.2	3.2	97	3.7	3.4	391	22.0%	0.50 [-0.22, 1.22]
2015Alcalay, R. N.	3.7	3.3	116	3.2	3.2	120	16.6%	0.50 [-0.33, 1.33]
2017San Luciano, M.	5.4	4.2	530	5	4.1	759	53.6%	0.40 [-0.06, 0.86]
**Total (95% CI)**			**848**			**1375**	**100.0%**	**0.43 [0.09, 0.77]**

Heterogeneity: Chi² = 6.70, df = 4 (P = 0.15); I² = 40%
Test for overall effect: Z = 2.51 (P = 0.01)

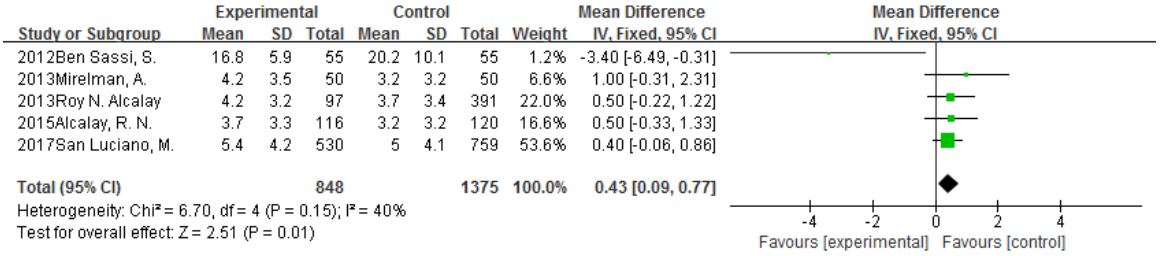

1.28 GDS15 of G2019S

Study or Subgroup	Experimental Events	Experimental Total	Control Events	Control Total	Weight	Odds Ratio M-H, Fixed, 95% CI
2009RN Alcalay	1	26	46	665	10.0%	0.54 [0.07, 4.06]
2010Soreya Belarbi	6	23	3	48	4.3%	5.29 [1.19, 23.58]
2013Emilia Mabel Gatto	3	3	12	48	0.7%	20.44 [0.99, 423.90]
2014Carles Gaig	3	33	5	33	13.7%	0.56 [0.12, 2.56]
2015Somme, J. H.	0	12	7	27	13.7%	0.11 [0.01, 2.08]
2017Bouhouche, A.	25	41	28	59	26.9%	1.73 [0.77, 3.89]
2017da Silva, C. P.	3	17	39	90	30.7%	0.28 [0.08, 1.04]
**Total (95% CI)**		**155**		**970**	**100.0%**	**1.07 [0.67, 1.71]**
Total events	41		140			

Heterogeneity: Chi² = 16.82, df = 6 (P = 0.010); I² = 64%
Test for overall effect: Z = 0.28 (P = 0.78)

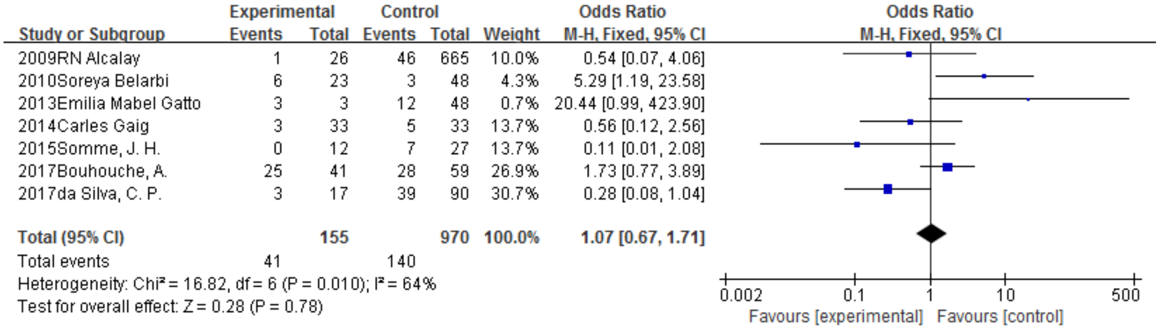

1.29 Hallucination of G2019S

Study or Subgroup	Experimental Mean	Experimental SD	Experimental Total	Control Mean	Control SD	Control Total	Weight	Mean Difference IV, Fixed, 95% CI
2013Beatriz Tijero	8.33	10.74	6	14.66	7.23	13	1.2%	-6.33 [-15.78, 3.12]
2014Carles Gaig	16.4	8.7	33	13.5	8.5	33	6.4%	2.90 [-1.25, 7.05]
2017San Luciano, M.	14.5	10.1	530	14.3	9.5	759	92.3%	0.20 [-0.89, 1.29]
**Total (95% CI)**			**569**			**805**	**100.0%**	**0.29 [-0.76, 1.34]**

Heterogeneity: Chi² = 3.43, df = 2 (P = 0.18); I² = 42%
Test for overall effect: Z = 0.55 (P = 0.59)

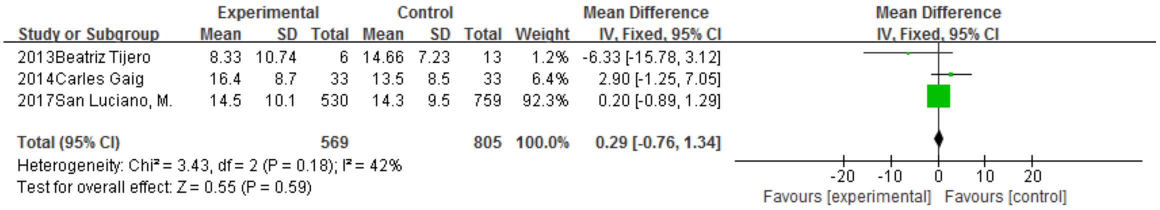

1.30 SCOPA-AUT of G2019S

Study or Subgroup	Experimental Events	Experimental Total	Control Events	Control Total	Weight	Odds Ratio M-H, Fixed, 95% CI
2005Jose Miguel Bras	2	11	13	117	5.0%	1.78 [0.35, 9.14]
2010Soreya Belarbi	10	23	10	48	10.0%	2.92 [0.99, 8.60]
2012Ben Sassi, S.	16	55	18	55	35.0%	0.84 [0.38, 1.90]
2016Dagan, E.	4	20	47	181	20.5%	0.71 [0.23, 2.24]
2017Bouhouche, A.	26	41	26	59	21.4%	2.20 [0.97, 4.98]
2017da Silva, C. P.	5	16	13	82	8.0%	2.41 [0.72, 8.11]
**Total (95% CI)**		**166**		**542**	**100.0%**	**1.49 [0.99, 2.24]**
Total events	63		127			

Heterogeneity: Chi² = 6.52, df = 5 (P = 0.26); I² = 23%
Test for overall effect: Z = 1.90 (P = 0.06)

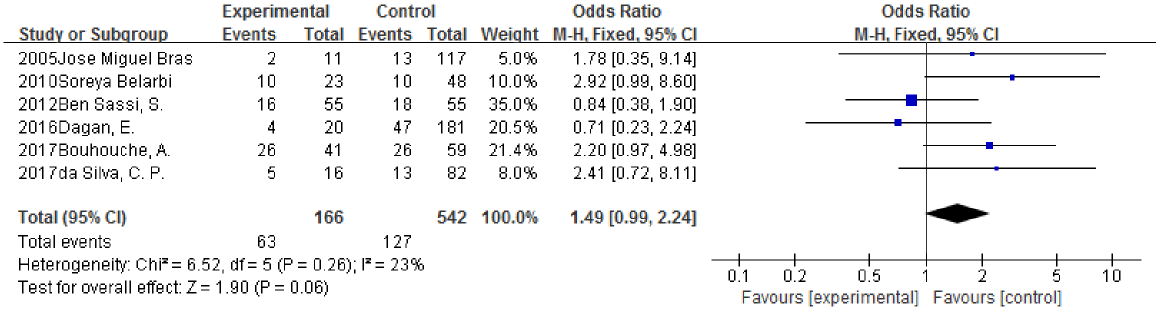

1.31 Cognitive impairments of G2019S

Study or Subgroup	Experimental Mean	Experimental SD	Experimental Total	Control Mean	Control SD	Control Total	Weight	Mean Difference IV, Fixed, 95% CI
2006Lesage S	26.3	4.6	14	28.4	2.6	45	3.0%	-2.10 [-4.63, 0.43]
2008S. Lesage	28.3	3.7	26	29.2	2.2	64	8.3%	-0.90 [-2.42, 0.62]
2009RN Alcalay	29.1	1.6	26	29.1	1.6	665	49.0%	0.00 [-0.63, 0.63]
2010Vicki Shanker	29.19	1.4	21	28.67	1.91	21	18.8%	0.52 [-0.49, 1.53]
2012Ben Sassi, S.	25.5	3.2	55	25.16	2.85	55	15.0%	0.34 [-0.79, 1.47]
2016Pal, G. D.	28.2	1.8	4	28.6	2	72	5.8%	-0.40 [-2.22, 1.42]
**Total (95% CI)**			**146**			**922**	**100.0%**	**-0.01 [-0.45, 0.43]**

Heterogeneity: Chi² = 5.54, df = 5 (P = 0.35); I² = 10%
Test for overall effect: Z = 0.06 (P = 0.95)

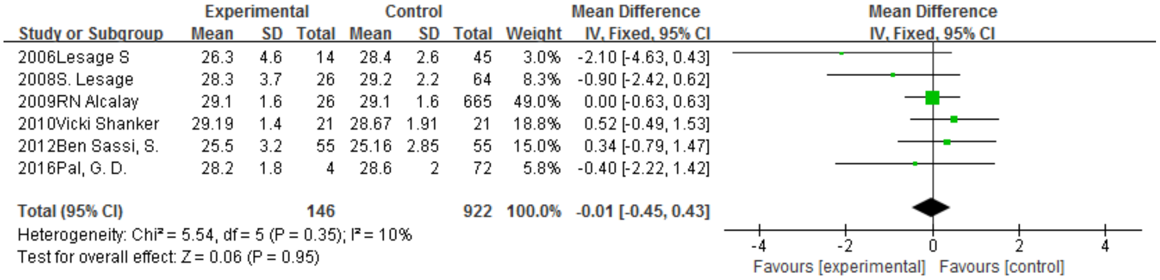

1.32 MMSE of G2019S

	Experimental			Control				Mean Difference
Study or Subgroup	Mean	SD	Total	Mean	SD	Total	Weight	IV, Fixed, 95% CI
2012Ben Sassi, S.	21.8	4.3	20	21.8	3.7	17	1.2%	0.00 [-2.58, 2.58]
2013Roy N. Alcalay	25.2	2.9	97	24.7	3.9	391	16.8%	0.50 [-0.19, 1.19]
2014Rachel Saunders-Pullman	25.4	3	126	25.2	3.8	126	11.4%	0.20 [-0.65, 1.05]
2015Alcalay, R. N.	25.4	2.7	116	25	3.4	120	13.3%	0.40 [-0.38, 1.18]
2017San Luciano, M.	24.1	4.3	530	24.5	4.4	759	35.0%	-0.40 [-0.88, 0.08]
2018Saunders-Pullman, R.	25.14	2.85	144	24.71	3.9	401	22.4%	0.43 [-0.17, 1.03]
**Total (95% CI)**			**1033**			**1814**	**100.0%**	**0.12 [-0.17, 0.40]**

Heterogeneity: $Chi^2 = 7.18$, df = 5 (P = 0.21); $I^2 = 30\%$
Test for overall effect: Z = 0.80 (P = 0.42)

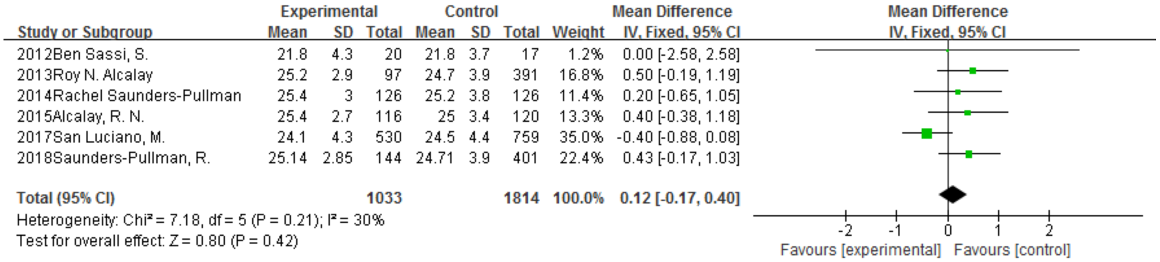

1.33 MoCA of G2019S

	Experimental		Control			Odds Ratio
Study or Subgroup	Events	Total	Events	Total	Weight	M-H, Random, 95% CI
2010Soreya Belarbi	15	23	19	48	16.5%	2.86 [1.02, 8.06]
2014Carles Gaig	22	33	19	33	16.9%	1.47 [0.54, 4.01]
2015Saunders-Pullman, R.	28	142	44	144	21.7%	0.56 [0.32, 0.96]
2015Somme, J. H.	6	12	11	27	13.2%	1.45 [0.37, 5.71]
2017Bouhouche, A.	22	41	19	59	18.8%	2.44 [1.07, 5.54]
2017da Silva, C. P.	13	17	76	82	12.9%	0.26 [0.06, 1.04]
**Total (95% CI)**		**268**		**393**	**100.0%**	**1.17 [0.56, 2.41]**
Total events	106		188			

Heterogeneity: $Tau^2 = 0.56$; $Chi^2 = 17.39$, df = 5 (P = 0.004); $I^2 = 71\%$
Test for overall effect: Z = 0.41 (P = 0.68)

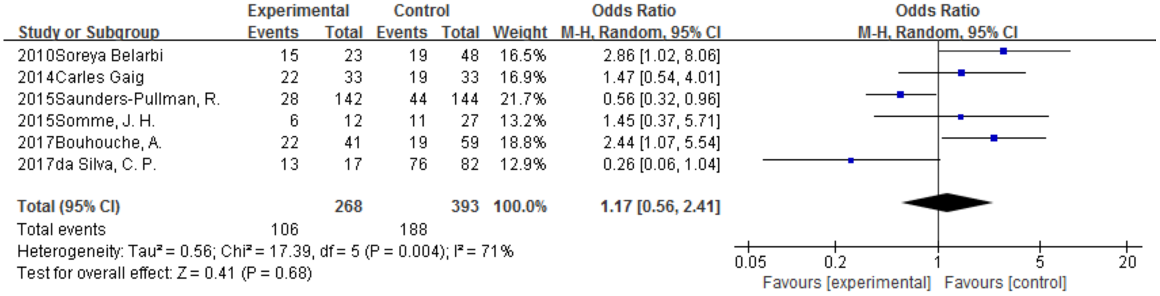

1.34 Sleep disturbances of G2019S

	Experimental		Control			Odds Ratio
Study or Subgroup	Events	Total	Events	Total	Weight	M-H, Random, 95% CI
2011R. Saunders-Pullman	18	31	25	30	15.7%	0.28 [0.08, 0.92]
2014Carles Gaig	13	33	25	33	16.4%	0.21 [0.07, 0.60]
2014Rachel Saunders-Pullman	69	126	101	126	18.6%	0.30 [0.17, 0.53]
2016Marras, C.	135	137	194	212	14.2%	6.26 [1.43, 27.44]
2017da Silva, C. P.	11	17	21	39	15.8%	1.57 [0.48, 5.10]
2017San Luciano, M.	160	530	131	759	19.3%	2.07 [1.59, 2.70]
**Total (95% CI)**		**874**		**1199**	**100.0%**	**0.81 [0.28, 2.36]**
Total events	406		497			

Heterogeneity: $Tau^2 = 1.52$; $Chi^2 = 60.67$, df = 5 (P < 0.00001); $I^2 = 92\%$
Test for overall effect: Z = 0.38 (P = 0.70)

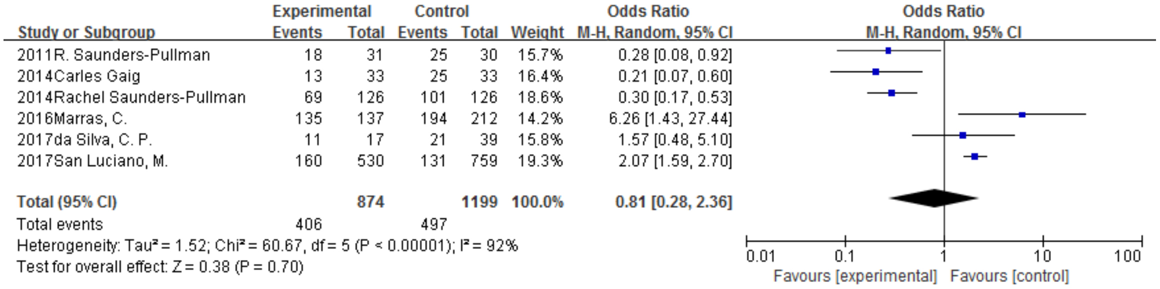

1.35 Olfactory disturbances of G2019S

	Experimental			Control				Mean Difference
Study or Subgroup	Mean	SD	Total	Mean	SD	Total	Weight	IV, Fixed, 95% CI
2011R. Saunders-Pullman	24.8	7.08	31	18.8	8.05	30	15.9%	6.00 [2.19, 9.81]
2014Carles Gaig	23.5	6.8	33	18.4	6	33	24.1%	5.10 [2.01, 8.19]
2014Rachel Saunders-Pullman	22.8	8.7	126	18.6	7.1	126	60.0%	4.20 [2.24, 6.16]
**Total (95% CI)**			**190**			**189**	**100.0%**	**4.70 [3.18, 6.22]**

Heterogeneity: $Chi^2 = 0.76$, df = 2 (P = 0.68); $I^2 = 0\%$
Test for overall effect: Z = 6.07 (P < 0.00001)

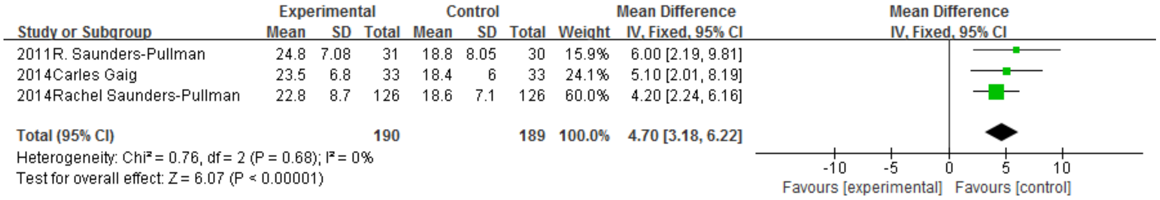

1.36 UPSIT scores of G2019S

	Experimental		Control			Odds Ratio
Study or Subgroup	Events	Total	Events	Total	Weight	M-H, Fixed, 95% CI
2006L.N. Clark	13	14	311	320	6.6%	0.38 [0.04, 3.19]
2006Lesage S	18	33	34	73	33.9%	1.38 [0.60, 3.14]
2011C. Marras	23	24	65	69	4.9%	1.42 [0.15, 13.33]
2013Roy N. Alcalay	35	97	58	391	51.9%	3.24 [1.97, 5.34]
2017da Silva, C. P.	16	16	83	91	2.7%	3.36 [0.18, 61.09]
**Total (95% CI)**		**184**		**944**	**100.0%**	**2.33 [1.54, 3.54]**
Total events	105		551			

Heterogeneity: $Chi^2 = 6.28$, df = 4 (P = 0.18); $I^2 = 36\%$
Test for overall effect: Z = 4.00 (P < 0.0001)

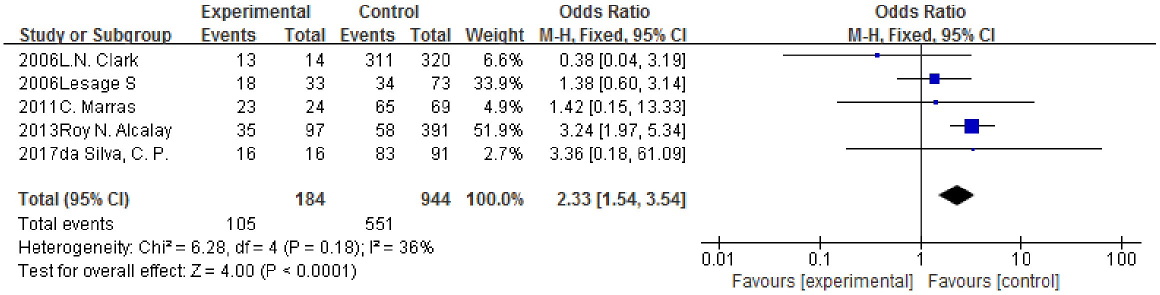

1.37 Good response to l-dopa of G2019S

Study or Subgroup	Experimental Mean	Experimental SD	Experimental Total	Control Mean	Control SD	Control Total	Weight	Mean Difference IV, Fixed, 95% CI
2006Lesage S	697	322	30	601	356	48	4.0%	96.00 [-57.03, 249.03]
2008S. Lesage	580	335	31	491	245	58	5.3%	89.00 [-44.72, 222.72]
2009RN Alcalay	556	624	26	471	492	665	1.6%	85.00 [-157.75, 327.75]
2011C. Marras	631	410	25	585	423	73	2.7%	46.00 [-141.74, 233.74]
2012Ben Sassi, S.	558.1	321.7	55	517.7	229.5	55	8.7%	40.40 [-64.04, 144.84]
2013Mirelman, A.	300.1	251.9	50	191.8	262.8	50	9.3%	108.30 [7.40, 209.20]
2013Roy N. Alcalay	443	444	97	348	373	391	10.3%	95.00 [-0.78, 190.78]
2014Carles Gaig	793.7	482.1	33	823	516.4	33	1.6%	-29.30 [-270.34, 211.74]
2015Alcalay, R. N.	389	402	116	220	288	120	11.8%	169.00 [79.52, 258.48]
2015Somme, J. H.	1,066.3	388	12	857	325	27	1.5%	209.30 [-42.14, 460.74]
2016Marras, C.	539.31	578.5	516	366.13	373	527	26.9%	173.18 [113.97, 232.39]
2018Saunders-Pullman, R.	407.39	410.23	144	337.1	367.58	401	16.3%	70.29 [-5.76, 146.34]
**Total (95% CI)**			**1135**			**2448**	**100.0%**	**115.20 [84.47, 145.93]**

Heterogeneity: Chi² = 11.28, df = 11 (P = 0.42); I² = 2%
Test for overall effect: Z = 7.35 (P < 0.00001)

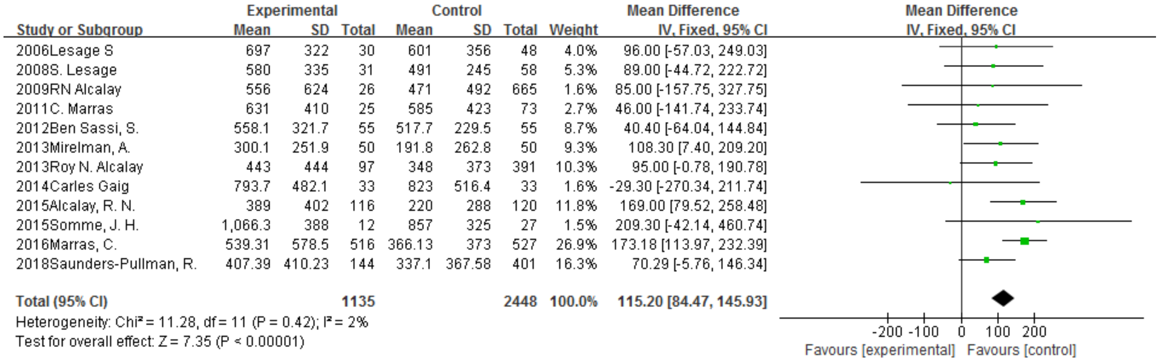

1.38 LEDD of G2019S

Study or Subgroup	Experimental Events	Experimental Total	Control Events	Control Total	Weight	Odds Ratio M-H, Fixed, 95% CI
2011R. Saunders-Pullman	11	31	11	29	6.6%	0.90 [0.31, 2.57]
2014Rachel Saunders-Pullman	65	126	44	126	19.1%	1.99 [1.20, 3.29]
2016Marras, C.	407	498	584	783	74.3%	1.52 [1.15, 2.01]
**Total (95% CI)**		**655**		**938**	**100.0%**	**1.57 [1.24, 1.99]**
Total events	483		639			

Heterogeneity: Chi² = 1.95, df = 2 (P = 0.38); I² = 0%
Test for overall effect: Z = 3.73 (P = 0.0002)

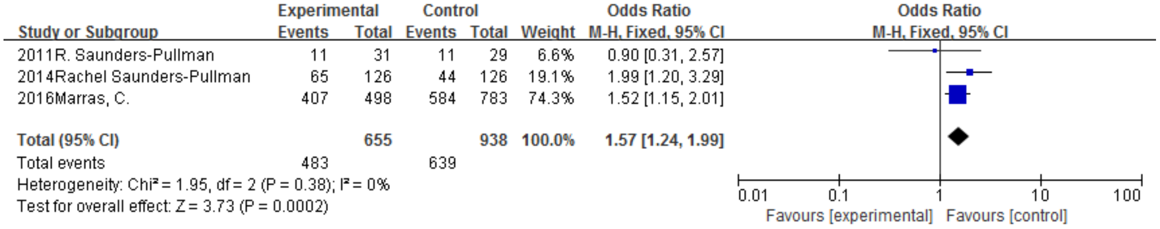

1.39 Smoke of G2019S

Study or Subgroup	Experimental Events	Experimental Total	Control Events	Control Total	Weight	Odds Ratio M-H, Random, 95% CI
2006Hon-Chung Fung	20	27	203	278	37.0%	1.06 [0.43, 2.60]
2008Daniel Kam Yin Chan	3	3	28	31	25.3%	0.86 [0.04, 20.35]
2016Sun, Qian	20	76	203	225	37.7%	0.04 [0.02, 0.08]
**Total (95% CI)**		**106**		**534**	**100.0%**	**0.29 [0.02, 4.33]**
Total events	43		434			

Heterogeneity: Tau² = 4.95; Chi² = 34.89, df = 2 (P < 0.00001); I² = 94%
Test for overall effect: Z = 0.90 (P = 0.37)

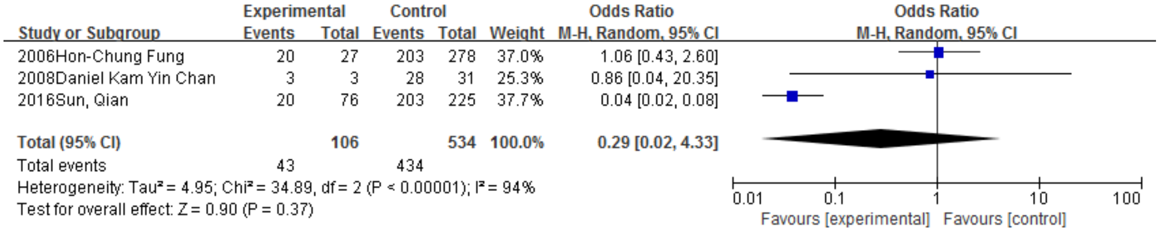

1.40 Asymmetrical onset of G2385R

Study or Subgroup	Experimental Mean	Experimental SD	Experimental Total	Control Mean	Control SD	Control Total	Weight	Mean Difference IV, Random, 95% CI
2006Hon-Chung Fung	61.5	11.3	27	61.9	10.5	278	6.1%	-0.40 [-4.84, 4.04]
2006Manabu Funayama	54	10.6	50	50.3	14.9	389	8.6%	3.70 [0.41, 6.99]
2007Cao Li	57	11	14	54.4	13.4	221	3.9%	2.60 [-3.43, 8.63]
2007Eng-King Tan	59	11	33	59	10	29	4.8%	0.00 [-5.23, 5.23]
2007Matthew J. Farrer	54.9	11.8	34	58	11.7	376	6.6%	-3.10 [-7.24, 1.04]
2008X.-K. An	52.75	9.8	71	56.32	11	529	11.0%	-3.57 [-6.03, -1.11]
2010Jong-Min Kim	55.3	11.5	82	54.7	10.8	841	10.6%	0.60 [-1.99, 3.19]
2013Chao Gao	60.5	10.4	36	58.2	8.8	139	7.6%	2.30 [-1.40, 6.00]
2013Jiangping Cai	56.8	10.7	49	58.6	11	461	8.9%	-1.80 [-4.96, 1.36]
2013Xiaoli Fu	55	11.7	37	55	11	409	7.1%	0.00 [-3.92, 3.92]
2016Marras, C.	55.99	11.1	197	58.69	12.5	790	13.3%	-2.70 [-4.48, -0.92]
2016Sun, Qian	55.57	8.78	76	57.05	8.92	225	11.6%	-1.48 [-3.77, 0.81]
**Total (95% CI)**			**706**			**4687**	**100.0%**	**-0.66 [-2.01, 0.70]**

Heterogeneity: Tau² = 2.76; Chi² = 23.25, df = 11 (P = 0.02); I² = 53%
Test for overall effect: Z = 0.95 (P = 0.34)

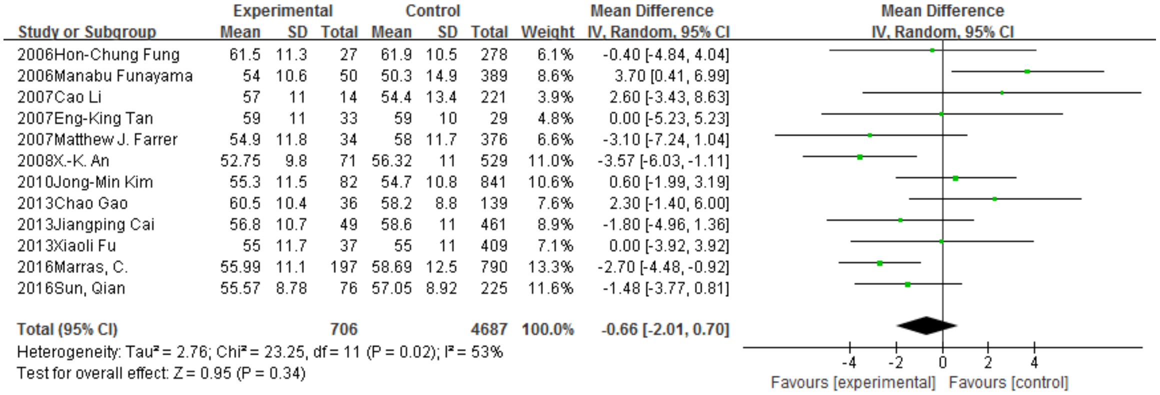

1.41 Age at onset of G2385R

	Experimental		Control			Odds Ratio
**Study or Subgroup**	**Events**	**Total**	**Events**	**Total**	**Weight**	**M-H, Fixed, 95% CI**
2006Di Fonzo, A.	23	61	192	547	21.3%	1.12 [0.65, 1.93]
2006Manabu Funayama	17	50	180	389	24.0%	0.60 [0.32, 1.11]
2007Cao Li	4	14	76	221	5.7%	0.76 [0.23, 2.51]
2008X.-K. An	26	71	124	529	16.5%	1.89 [1.12, 3.18]
2012HUIRU YAN	5	21	72	333	5.8%	1.13 [0.40, 3.20]
2013Chao Gao	5	36	5	139	1.6%	4.32 [1.18, 15.86]
2013Jiangping Cai	11	49	105	461	13.9%	0.98 [0.48, 1.99]
2013Xiaoli Fu	11	37	109	409	11.3%	1.16 [0.56, 2.44]
**Total (95% CI)**		**339**		**3028**	**100.0%**	**1.14 [0.89, 1.46]**
Total events	102		863			

Heterogeneity: $Chi^2 = 12.41, df = 7 (P = 0.09); I^2 = 44\%$
Test for overall effect: Z = 1.01 (P = 0.31)

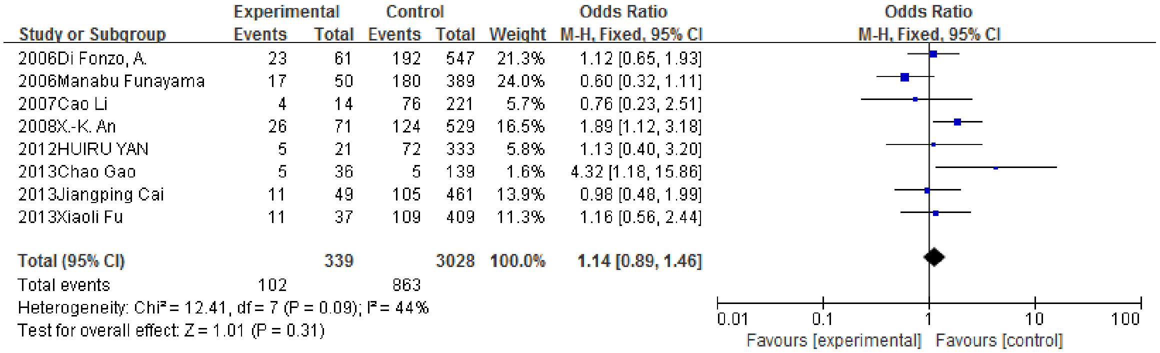

1.42 EOPD of G2385R

	Experimental		Control			Odds Ratio
**Study or Subgroup**	**Events**	**Total**	**Events**	**Total**	**Weight**	**M-H, Random, 95% CI**
2006Di Fonzo, A.	34	61	328	547	8.4%	0.84 [0.49, 1.43]
2007Cao Li	5	14	128	221	4.5%	0.40 [0.13, 1.24]
2007Eng-King Tan	16	33	17	29	5.1%	0.66 [0.24, 1.82]
2007Matthew J. Farrer	18	34	214	376	7.1%	0.85 [0.42, 1.72]
2008Daniel Kam Yin Chan	3	3	21	31	0.9%	3.42 [0.16, 72.45]
2008X.-K. An	40	71	314	529	8.7%	0.88 [0.54, 1.46]
2010Jong-Min Kim	35	82	373	841	9.0%	0.93 [0.59, 1.48]
2012HUIRU YAN	10	21	192	333	5.8%	0.67 [0.28, 1.62]
2013Chao Gao	21	36	15	139	6.0%	11.57 [4.94, 27.13]
2013Jiangping Cai	28	49	275	461	7.9%	0.90 [0.50, 1.64]
2013Xiaoli Fu	21	37	201	409	7.3%	1.36 [0.69, 2.68]
2016Cao, M.	16	25	28	43	5.0%	0.95 [0.34, 2.67]
2016Marras, C.	122	199	465	790	10.1%	1.11 [0.81, 1.52]
2016Sun, Qian	37	76	125	225	8.5%	0.76 [0.45, 1.28]
2017Hong, J. H.	7	23	136	276	5.6%	0.45 [0.18, 1.13]
**Total (95% CI)**		**764**		**5250**	**100.0%**	**0.98 [0.73, 1.34]**
Total events	413		2832			

Heterogeneity: $Tau^2 = 0.21; Chi^2 = 42.45, df = 14 (P = 0.0001); I^2 = 67\%$
Test for overall effect: Z = 0.10 (P = 0.92)

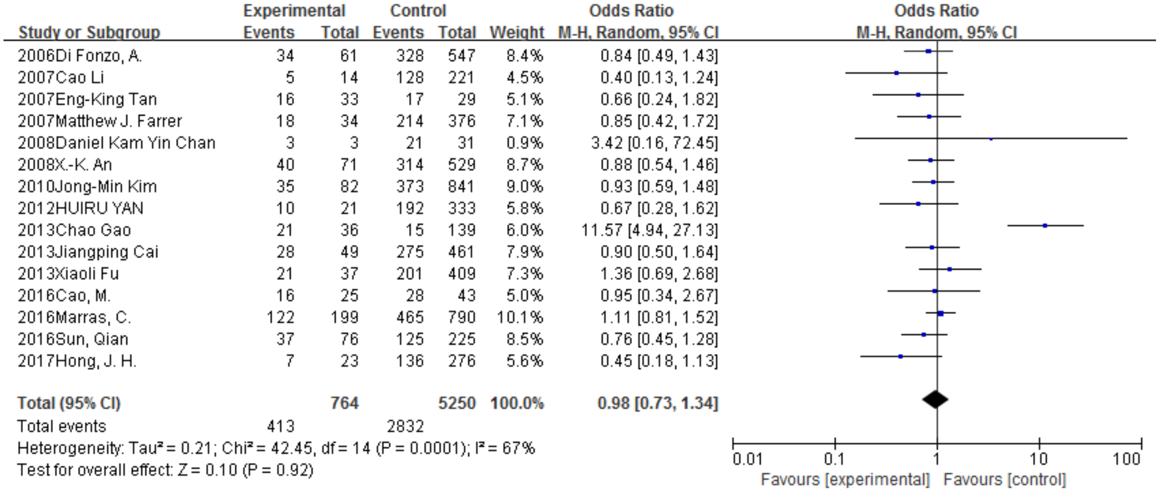

1.43 Gender-male of G2385R

	Experimental		Control			Odds Ratio
**Study or Subgroup**	**Events**	**Total**	**Events**	**Total**	**Weight**	**M-H, Fixed, 95% CI**
2007Cao Li	0	14	13	221	10.8%	0.53 [0.03, 9.42]
2007Eng-King Tan	5	33	0	29	2.9%	11.39 [0.60, 215.48]
2007Matthew J. Farrer	6	34	20	376	17.8%	3.81 [1.42, 10.27]
2013Chao Gao	2	36	10	139	25.3%	0.76 [0.16, 3.63]
2013Xiaoli Fu	8	37	51	409	43.2%	1.94 [0.84, 4.47]
**Total (95% CI)**		**154**		**1174**	**100.0%**	**2.10 [1.22, 3.59]**
Total events	21		94			

Heterogeneity: $Chi^2 = 5.21, df = 4 (P = 0.27); I^2 = 23\%$
Test for overall effect: Z = 2.69 (P = 0.007)

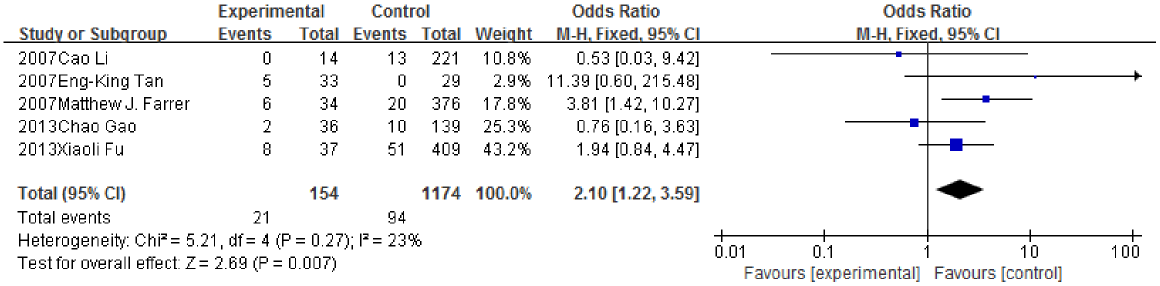

1.44 Family history of G2385R

	Experimental		Control			Odds Ratio
**Study or Subgroup**	**Events**	**Total**	**Events**	**Total**	**Weight**	**M-H, Random, 95% CI**
2007Cao Li	7	14	102	221	20.9%	1.17 [0.40, 3.44]
2007Matthew J. Farrer	30	34	365	376	18.7%	0.23 [0.07, 0.75]
2013Jiangping Cai	24	49	224	461	32.1%	1.02 [0.56, 1.83]
2013Xiaoli Fu	11	37	81	409	28.3%	1.71 [0.81, 3.61]
**Total (95% CI)**		**134**		**1467**	**100.0%**	**0.92 [0.45, 1.85]**
Total events	72		772			

Heterogeneity: $Tau^2 = 0.31; Chi^2 = 7.97, df = 3 (P = 0.05); I^2 = 62\%$
Test for overall effect: Z = 0.25 (P = 0.80)

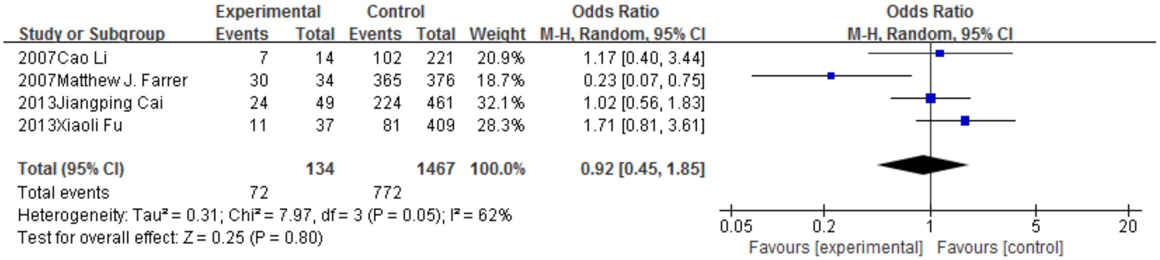

1.45 FS-Bradykinesia of G2385R

Study or Subgroup	Experimental Events	Experimental Total	Control Events	Control Total	Weight	Odds Ratio M-H, Fixed, 95% CI
2007Cao Li	8	14	134	221	9.8%	0.87 [0.29, 2.58]
2008X.-K. An	37	71	263	529	42.6%	1.10 [0.67, 1.81]
2013Jiangping Cai	34	49	299	461	25.1%	1.23 [0.65, 2.32]
2013Xiaoli Fu	21	37	220	409	22.5%	1.13 [0.57, 2.22]
**Total (95% CI)**		**171**		**1620**	**100.0%**	**1.12 [0.81, 1.54]**
Total events	100		916			

Heterogeneity: Chi² = 0.30, df = 3 (P = 0.96); I² = 0%
Test for overall effect: Z = 0.66 (P = 0.51)

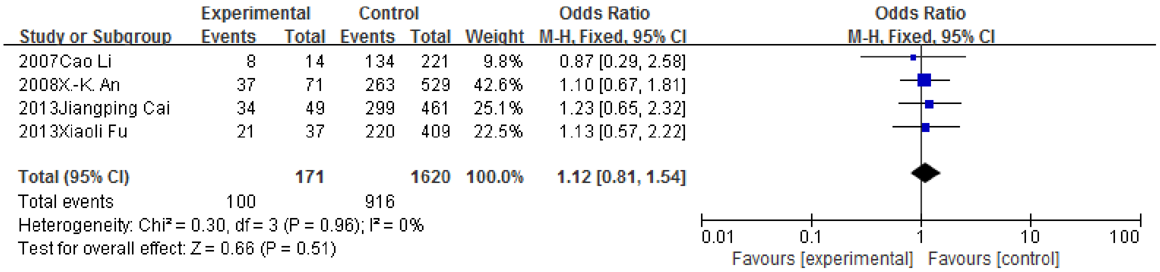

1.46 FS-Resting tremor of G2385R

Study or Subgroup	Experimental Events	Experimental Total	Control Events	Control Total	Weight	Odds Ratio M-H, Fixed, 95% CI
2007Cao Li	7	14	83	221	16.9%	1.66 [0.56, 4.91]
2013Jiangping Cai	18	49	168	461	69.6%	1.01 [0.55, 1.87]
2013Xiaoli Fu	3	37	26	409	13.5%	1.30 [0.37, 4.52]
**Total (95% CI)**		**100**		**1091**	**100.0%**	**1.16 [0.71, 1.89]**
Total events	28		277			

Heterogeneity: Chi² = 0.65, df = 2 (P = 0.72); I² = 0%
Test for overall effect: Z = 0.60 (P = 0.55)

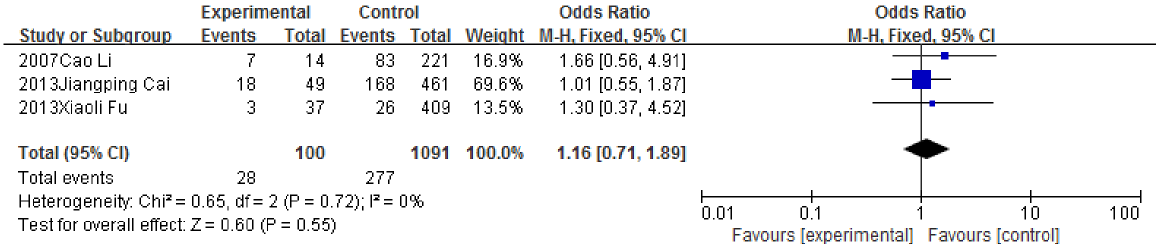

1.47 FS-Rigidity of G2385R

Study or Subgroup	Experimental Events	Experimental Total	Control Events	Control Total	Weight	Odds Ratio M-H, Fixed, 95% CI
2007Cao Li	5	14	64	221	52.6%	1.36 [0.44, 4.22]
2013Jiangping Cai	0	49	9	461	19.7%	0.48 [0.03, 8.39]
2013Xiaoli Fu	1	37	16	409	27.7%	0.68 [0.09, 5.29]
**Total (95% CI)**		**100**		**1091**	**100.0%**	**1.00 [0.40, 2.48]**
Total events	6		89			

Heterogeneity: Chi² = 0.67, df = 2 (P = 0.71); I² = 0%
Test for overall effect: Z = 0.00 (P = 1.00)

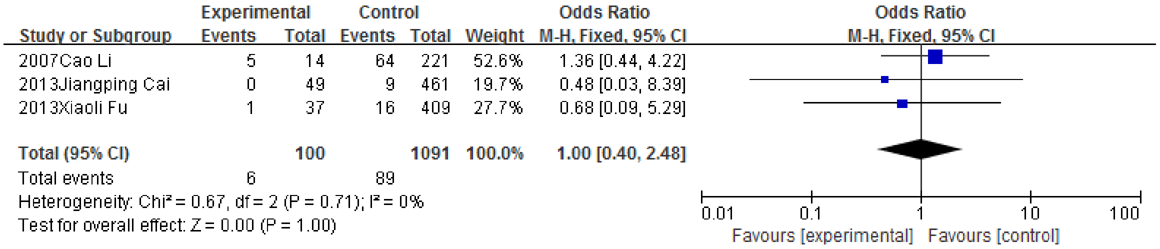

1.48 FS-Postural instability or Gait difficulty of G2385R

Study or Subgroup	Experimental Events	Experimental Total	Control Events	Control Total	Weight	Odds Ratio M-H, Fixed, 95% CI
2006Hon-Chung Fung	26	27	252	278	12.0%	2.68 [0.35, 20.58]
2007Matthew J. Farrer	25	34	258	376	81.9%	1.27 [0.58, 2.81]
2008Daniel Kam Yin Chan	3	3	30	31	6.1%	0.34 [0.01, 10.18]
**Total (95% CI)**		**64**		**685**	**100.0%**	**1.38 [0.68, 2.82]**
Total events	54		540			

Heterogeneity: Chi² = 1.10, df = 2 (P = 0.58); I² = 0%
Test for overall effect: Z = 0.89 (P = 0.37)

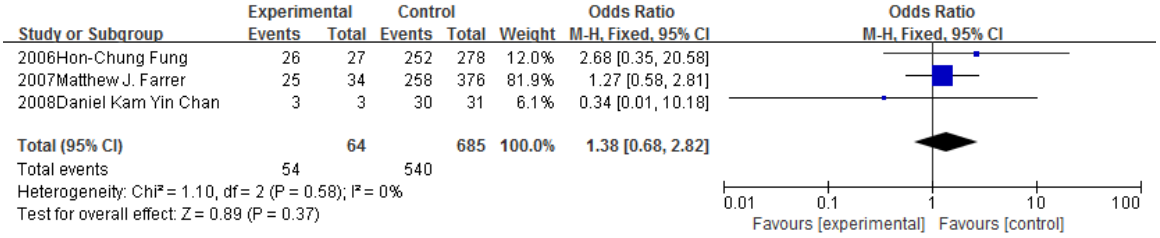

1.49 Resting tremor of G2385R

Study or Subgroup	Experimental Events	Experimental Total	Control Events	Control Total	Weight	Odds Ratio M-H, Fixed, 95% CI
2006Hon-Chung Fung	26	27	261	278	29.4%	1.69 [0.22, 13.24]
2007Matthew J. Farrer	32	34	343	376	57.5%	1.54 [0.35, 6.71]
2008Daniel Kam Yin Chan	3	3	27	31	13.1%	1.15 [0.05, 26.10]
**Total (95% CI)**		**64**		**685**	**100.0%**	**1.53 [0.50, 4.68]**
Total events	61		631			

Heterogeneity: Chi² = 0.04, df = 2 (P = 0.98); I² = 0%
Test for overall effect: Z = 0.75 (P = 0.45)

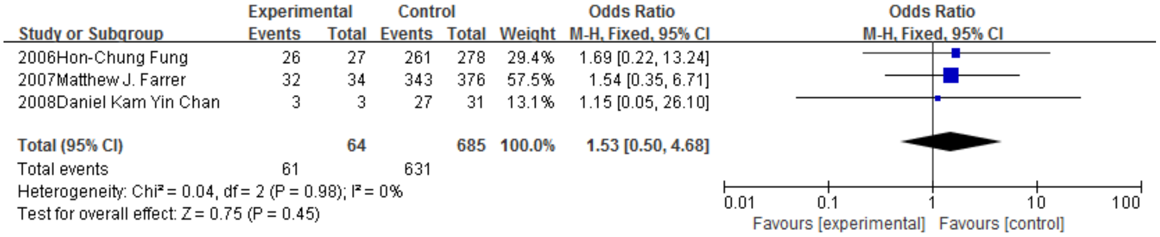

1.50 Rigidity of G2385R

Study or Subgroup	Experimental Mean	Experimental SD	Experimental Total	Control Mean	Control SD	Control Total	Weight	Mean Difference IV, Fixed, 95% CI
2007Cao Li	2.3	1.3	14	3.1	1.7	221	24.2%	-0.80 [-1.52, -0.08]
2013Jiangping Cai	2.57	2.45	49	2.96	2.26	461	24.2%	-0.39 [-1.11, 0.33]
2016Sun, Qian	1.92	1.98	76	1.67	1.58	225	51.6%	0.25 [-0.24, 0.74]
**Total (95% CI)**			**139**			**907**	**100.0%**	**-0.16 [-0.51, 0.19]**

Heterogeneity: $Chi^2 = 6.14, df = 2 (P = 0.05); I^2 = 67\%$
Test for overall effect: Z = 0.88 (P = 0.38)

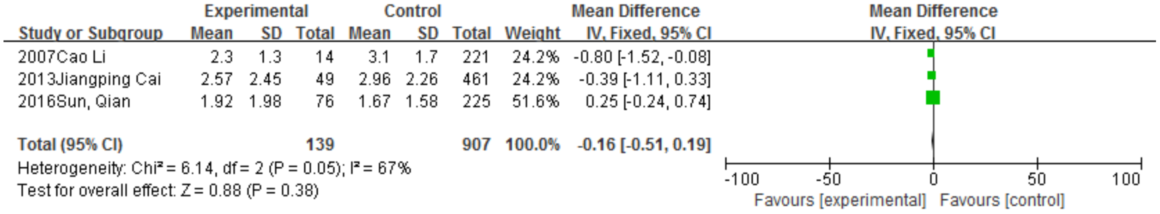

1.51 UPDRS Ⅰ of G2385R

Study or Subgroup	Experimental Mean	Experimental SD	Experimental Total	Control Mean	Control SD	Control Total	Weight	Mean Difference IV, Random, 95% CI
2007Cao Li	13.1	5.9	14	16.8	6.2	221	21.8%	-3.70 [-6.90, -0.50]
2013Jiangping Cai	16.8	8.8	49	17.2	8.26	461	23.7%	-0.40 [-2.98, 2.18]
2016Marras, C.	14.74	0.19	192	11.09	0.19	684	28.0%	3.65 [3.62, 3.68]
2016Sun, Qian	11.16	5.71	76	11.32	4.35	225	26.5%	-0.16 [-1.56, 1.24]
**Total (95% CI)**			**331**			**1591**	**100.0%**	**0.08 [-3.12, 3.27]**

Heterogeneity: $Tau^2 = 9.49; Chi^2 = 58.05, df = 3 (P < 0.00001); I^2 = 95\%$
Test for overall effect: Z = 0.05 (P = 0.96)

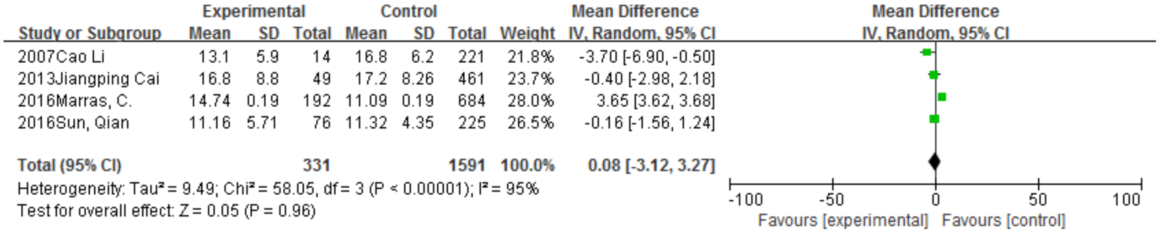

1.52 UPDRS Ⅱ of G2385R

Study or Subgroup	Experimental Mean	Experimental SD	Experimental Total	Control Mean	Control SD	Control Total	Weight	Mean Difference IV, Random, 95% CI
2007Cao Li	19.6	5.1	14	20.5	7.3	221	14.6%	-0.90 [-3.74, 1.94]
2007Eng-King Tan	26	11	33	27	11	29	12.9%	-1.00 [-6.49, 4.49]
2007Matthew J. Farrer	19.3	7.4	34	19.9	9.1	376	14.7%	-0.60 [-3.25, 2.05]
2013Jiangping Cai	21.4	8.78	49	22.1	8.35	461	14.7%	-0.70 [-3.27, 1.87]
2016Marras, C.	35.82	0.4	192	27.68	1.12	652	15.3%	8.14 [8.04, 8.24]
2016Sun, Qian	24.55	12.08	76	26.52	12.64	225	14.4%	-1.97 [-5.15, 1.21]
2017Hong, J. H.	19.8	11.2	23	23.6	8.7	276	13.5%	-3.80 [-8.49, 0.89]
**Total (95% CI)**			**421**			**2240**	**100.0%**	**-0.00 [-4.99, 4.99]**

Heterogeneity: $Tau^2 = 42.42; Chi^2 = 199.14, df = 6 (P < 0.00001); I^2 = 97\%$
Test for overall effect: Z = 0.00 (P = 1.00)

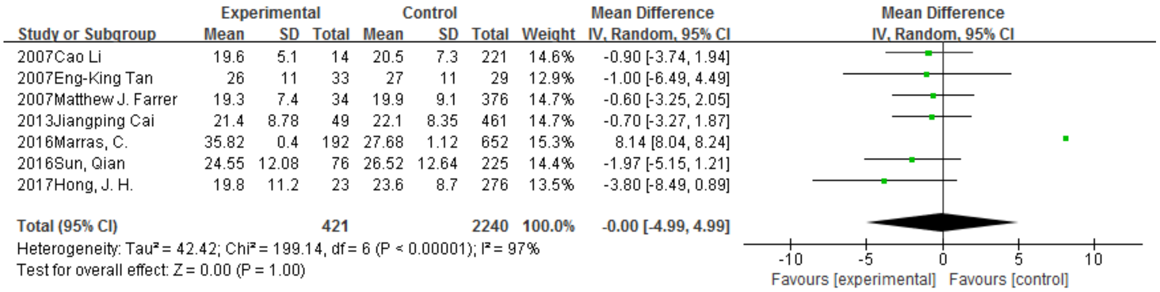

1.53 UPDRSⅢ of G2385R

Study or Subgroup	Experimental Mean	Experimental SD	Experimental Total	Control Mean	Control SD	Control Total	Weight	Mean Difference IV, Fixed, 95% CI
2007Cao Li	1.7	0.4	14	2	0.8	221	22.0%	-0.30 [-0.53, -0.07]
2007Matthew J. Farrer	2.5	1.2	34	2.6	0.9	376	7.1%	-0.10 [-0.51, 0.31]
2008X.-K. An	2.16	0.91	71	2.33	0.98	529	23.3%	-0.17 [-0.40, 0.06]
2013Jiangping Cai	2.47	0.89	49	2.57	0.93	461	17.4%	-0.10 [-0.36, 0.16]
2016Cao, M.	2.2	0.8	25	2.4	0.74	43	8.2%	-0.20 [-0.58, 0.18]
2016Sun, Qian	2.15	0.93	76	2.08	0.81	225	22.0%	0.07 [-0.16, 0.30]
**Total (95% CI)**			**269**			**1855**	**100.0%**	**-0.13 [-0.24, -0.02]**

Heterogeneity: $Chi^2 = 5.13, df = 5 (P = 0.40); I^2 = 3\%$
Test for overall effect: Z = 2.34 (P = 0.02)

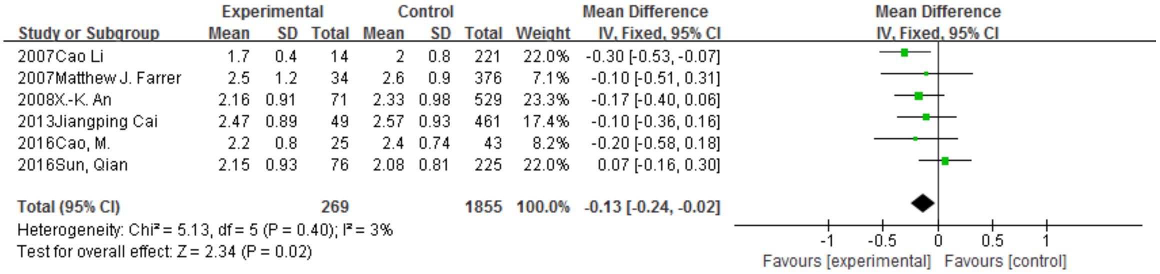

1.54 H-Y of G2385R

Study or Subgroup	Experimental Events	Experimental Total	Control Events	Control Total	Weight	Odds Ratio M-H, Fixed, 95% CI
2007Cao Li	3	14	57	221	8.3%	0.78 [0.21, 2.91]
2007Eng-King Tan	7	33	11	29	14.4%	0.44 [0.14, 1.35]
2007Matthew J. Farrer	13	34	117	376	18.7%	1.37 [0.66, 2.83]
2016Marras, C.	16	146	73	419	52.5%	0.58 [0.33, 1.04]
2016Sun, Qian	4	76	8	225	6.0%	1.51 [0.44, 5.15]
**Total (95% CI)**		**303**		**1270**	**100.0%**	**0.78 [0.54, 1.14]**
Total events	43		266			

Heterogeneity: $Chi^2 = 5.39, df = 4 (P = 0.25); I^2 = 26\%$
Test for overall effect: Z = 1.28 (P = 0.20)

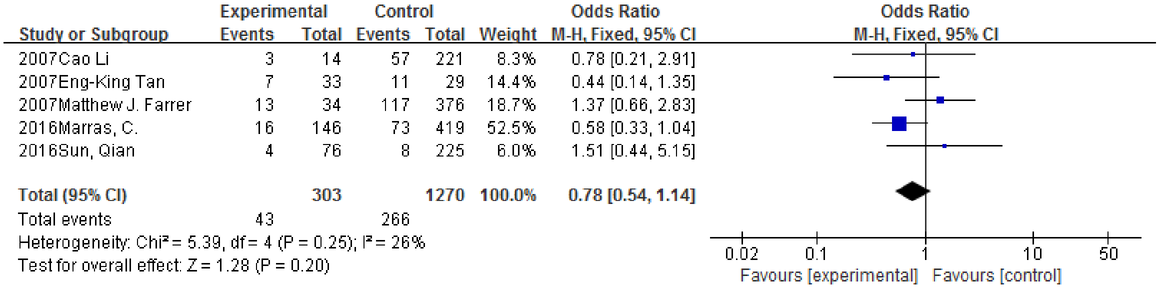

1.55 Dyskinesia of G2385R

Study or Subgroup	Experimental Events	Experimental Total	Control Events	Control Total	Weight	Odds Ratio M-H, Fixed, 95% CI
2007Matthew J. Farrer	12	34	122	376	33.7%	1.14 [0.54, 2.37]
2008Daniel Kam Yin Chan	0	3	3	31	1.7%	1.16 [0.05, 27.54]
2016Marras, C.	61	130	95	396	64.1%	2.80 [1.85, 4.24]
2016Sun, Qian	27	27	50	225	0.5%	191.14 [11.46, 3188.64]
**Total (95% CI)**		**194**		**1028**	**100.0%**	**3.17 [2.30, 4.38]**
Total events	100		270			

Heterogeneity: Chi² = 16.38, df = 3 (P = 0.0009); I² = 82%
Test for overall effect: Z = 7.05 (P < 0.00001)

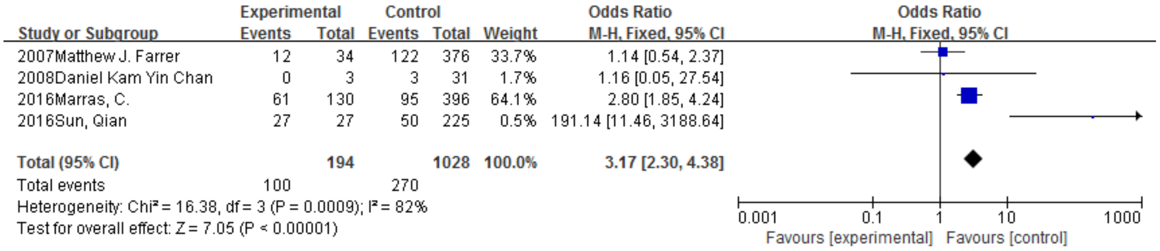

1.56 Motor fluctuations of G2385R

Study or Subgroup	Experimental Events	Experimental Total	Control Events	Control Total	Weight	Odds Ratio M-H, Fixed, 95% CI
2008Daniel Kam Yin Chan	1	3	12	31	5.2%	0.79 [0.06, 9.71]
2016Sun, Qian	27	76	58	225	69.8%	1.59 [0.91, 2.77]
2017Hong, J. H.	5	23	56	276	24.9%	1.09 [0.39, 3.07]
**Total (95% CI)**		**102**		**532**	**100.0%**	**1.42 [0.88, 2.29]**
Total events	33		126			

Heterogeneity: Chi² = 0.61, df = 2 (P = 0.74); I² = 0%
Test for overall effect: Z = 1.44 (P = 0.15)

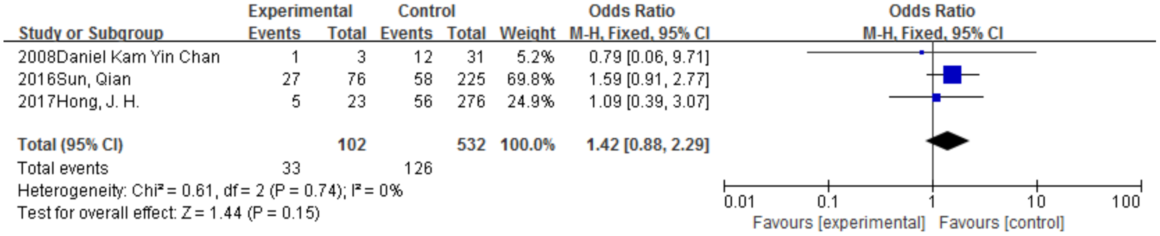

1.57 Depression of G2385R

Study or Subgroup	Experimental Mean	Experimental SD	Experimental Total	Control Mean	Control SD	Control Total	Weight	Mean Difference IV, Fixed, 95% CI
2013Chao Gao	27.2	3.4	36	25.3	4	139	20.9%	1.90 [0.61, 3.19]
2016Sun, Qian	27.78	2.68	76	26.96	2.92	225	68.9%	0.82 [0.11, 1.53]
2017Hong, J. H.	25.6	4.4	23	25	4	276	10.1%	0.60 [-1.26, 2.46]
**Total (95% CI)**			**135**			**640**	**100.0%**	**1.02 [0.43, 1.62]**

Heterogeneity: Chi² = 2.27, df = 2 (P = 0.32); I² = 12%
Test for overall effect: Z = 3.39 (P = 0.0007)

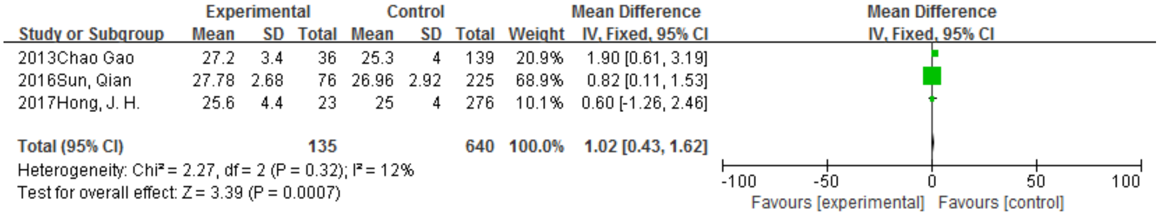

1.58 MMSE of G2385R

Study or Subgroup	Experimental Mean	Experimental SD	Experimental Total	Control Mean	Control SD	Control Total	Weight	Mean Difference IV, Fixed, 95% CI
2007Eng-King Tan	467	275	33	537	287	29	8.8%	-70.00 [-210.41, 70.41]
2007Matthew J. Farrer	457.6	246.6	34	444.6	270.9	376	22.7%	13.00 [-74.30, 100.30]
2016Marras, C.	446.24	351.6	161	366.13	373	527	43.6%	80.11 [17.15, 143.07]
2016Sun, Qian	553.67	329.84	76	467.71	286.34	225	25.0%	85.96 [2.90, 169.02]
**Total (95% CI)**			**304**			**1157**	**100.0%**	**53.22 [11.67, 94.77]**

Heterogeneity: Chi² = 5.07, df = 3 (P = 0.17); I² = 41%
Test for overall effect: Z = 2.51 (P = 0.01)

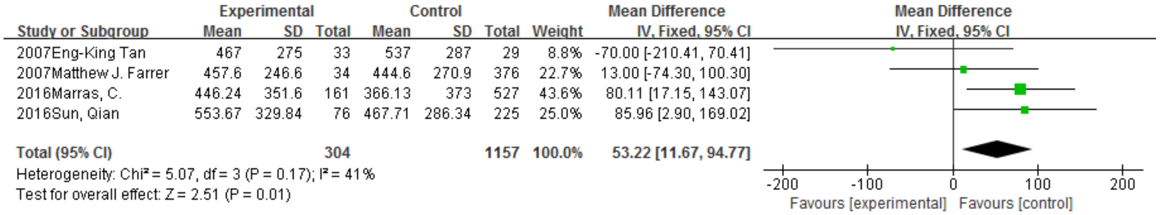

1.59 LEDD of G2385R

Study or Subgroup	Experimental Mean	Experimental SD	Experimental Total	Control Mean	Control SD	Control Total	Weight	Mean Difference IV, Random, 95% CI
2009Lihua Yu	53.53	10.35	328	52.6	11.55	311	19.4%	0.93 [-0.77, 2.63]
2009Zijuan Zhang	57.33	9.13	600	55.79	11.3	557	20.7%	1.54 [0.35, 2.73]
2013Jiangping Cai	55.6	10.7	510	58.6	10.9	481	20.3%	-3.00 [-4.35, -1.65]
2013Xiaoli Fu	54	11.7	446	55	11	399	19.9%	-1.00 [-2.53, 0.53]
2014Pulkes, T.	56	13	485	60.1	12.2	431	19.6%	-4.10 [-5.73, -2.47]
**Total (95% CI)**			**2369**			**2179**	**100.0%**	**-1.11 [-3.30, 1.07]**

Heterogeneity: Tau² = 5.62; Chi² = 44.73, df = 4 (P < 0.00001); I² = 91%
Test for overall effect: Z = 1.00 (P = 0.32)

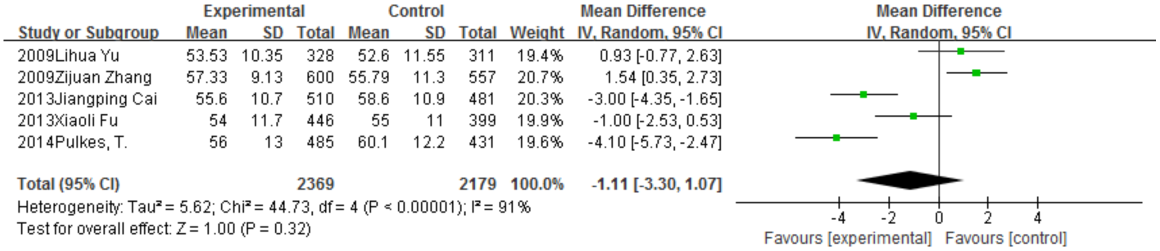

1.60 Age at onset of R1628P

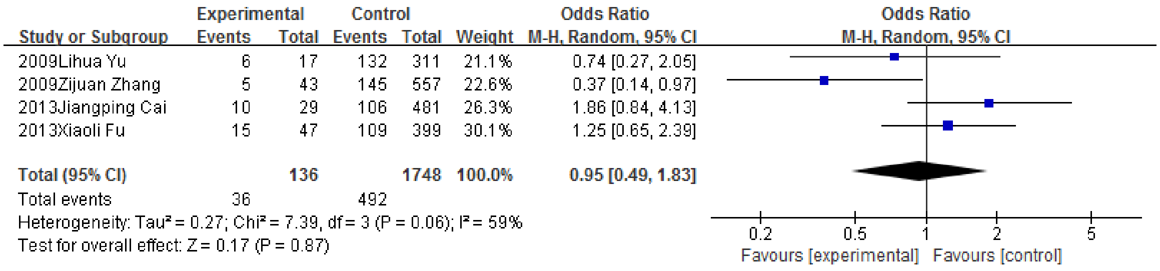

1.61 EOPD of R1628P

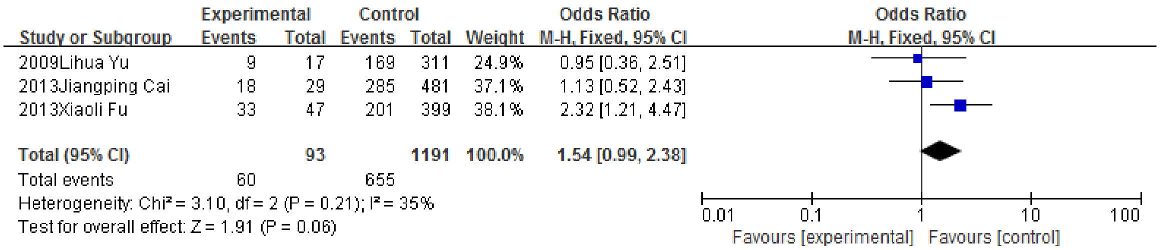

1.62 Gender-male of R1628P

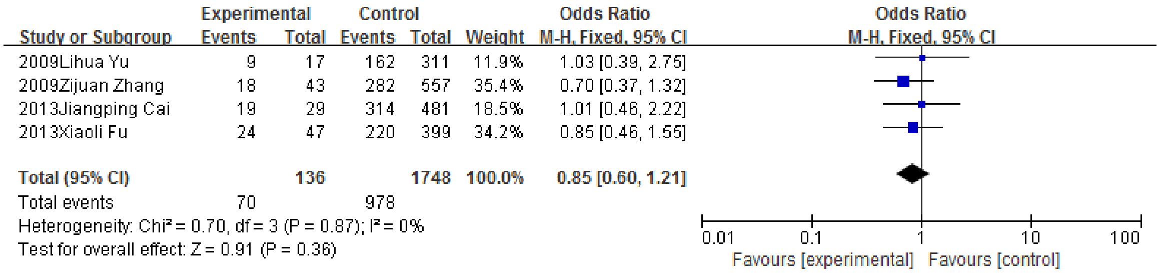

1.63 FS-Resting tremor of R1628P

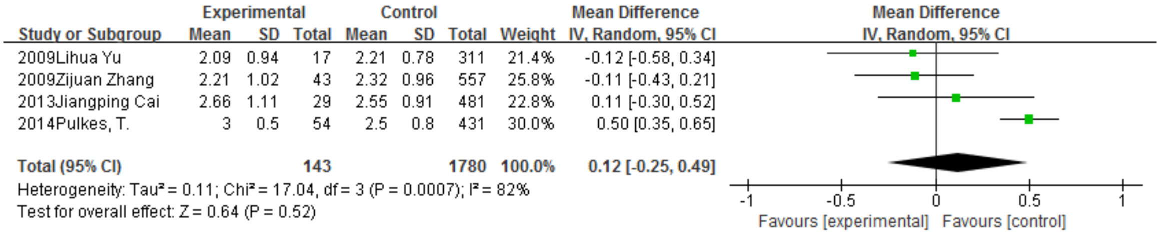

1.64 H-Y of R1628P

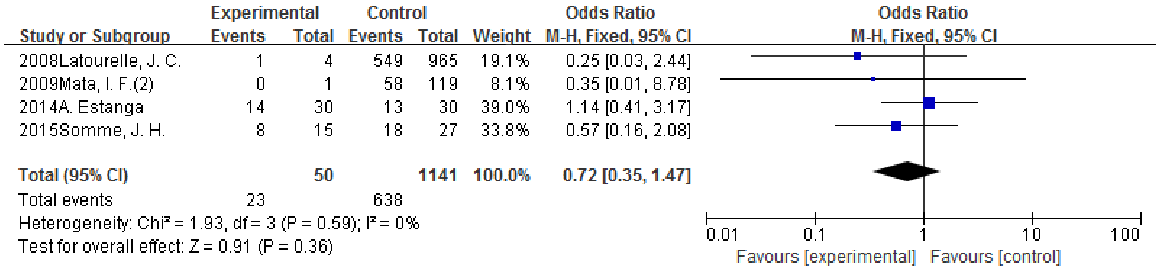

1.65 Gender-male of R1441G

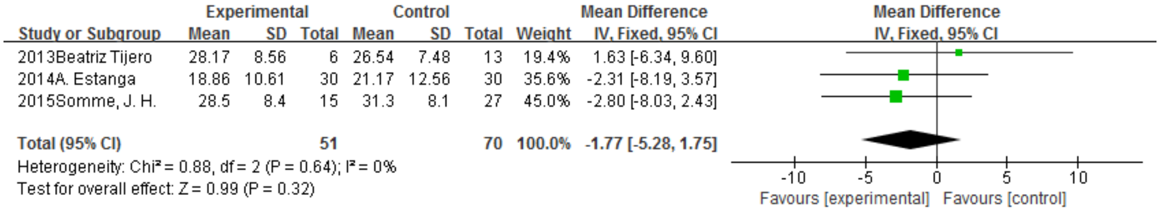

1.66 UPDRSⅢ of R1441G

Supplementary Figure 1. Forest plots of the association between specific variants in *LRRK2* and PD clinical features. 1.1-1.39 reflected the pooled results of G2019S-related clinical features.1.40-1.59 reflected the pooled results of G2385R-related clinical features. 1.60-1.64 represented the pooled results of R1628P-related clinical features. 1.65-1.66 represented the pooled results of R1441G-related clinical features.
