# Supplementary material for: Clinical Heterogeneity Among LRRK2 Variants in Parkinson's Disease: A Meta-Analysis
Source: Front Aging Neurosci. 2018 Sep 19;10:283. doi: 10.3389/fnagi.2018.00283 (PMC6156433; doi:10.3389/fnagi.2018.00283)
Supplement: Supplementary file 7 [file Image_3.pdf]

# *Supplementary Material*

## Clinical heterogeneity among *LRRK2* variants in Parkinson's disease: a meta-analysis

**Li Shu[2] †, Yuan Zhang[2] †, Hongxu Pan[2], Qian Xu[2, 3,4], Jifeng Guo[2,3,4,6,7,8], Beisha Tang[1, 2,3,4,5,6,7,8], Qiying Sun[1,3,4*]**

† These authors have contributed equally to this work and are co-first authors.

* **Correspondence**: Qiying Sun sunqiying2015@163.com

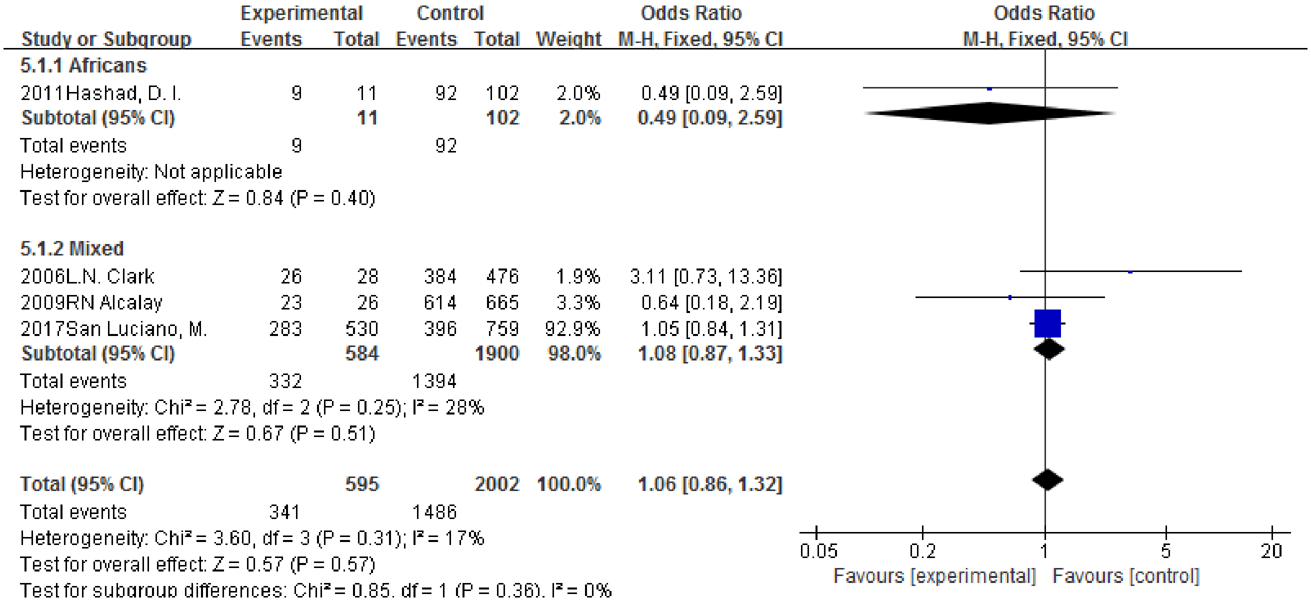


1.1 Asymmetrical onset of G2019S by ethnicity

| Study or Subgroup | Experimental Mean | Experimental SD | Experimental Total | Control Mean | Control SD | Control Total | Weight | Mean Difference IV, Random, 95% CI |
|---|---|---|---|---|---|---|---|---|
| **5.2.1 Africans** | | | | | | | | |
| 2006Lesage S | 45.2 | 9.4 | 33 | 43.2 | 15.3 | 73 | 2.8% | 2.00 [-2.75, 6.75] |
| 2007Ishihara, L. | 58.9 | 13 | 73 | 48.6 | 18.3 | 128 | 3.0% | 10.30 [5.95, 14.65] |
| 2008S. Lesage | 54.6 | 12.1 | 35 | 54.7 | 12.6 | 81 | 2.7% | -0.10 [-4.96, 4.76] |
| 2010Soreya Belarbi | 58.7 | 9.7 | 21 | 57.8 | 8.7 | 21 | 2.4% | 0.90 [-4.67, 6.47] |
| 2011Hashad, D. I. | 55.3 | 5.4 | 11 | 56.4 | 6.8 | 102 | 3.6% | -1.10 [-4.55, 2.35] |
| 2012Ben Sassi, S. | 54.4 | 10.3 | 55 | 55.3 | 8.6 | 55 | 3.5% | -0.90 [-4.45, 2.65] |
| 2013Trinh, J. | 57.1 | 11.6 | 220 | 55.3 | 14.4 | 350 | 4.4% | 1.80 [-0.35, 3.95] |
| 2017Bouhouche, A. | 52.15 | 11.28 | 41 | 55.12 | 11.65 | 59 | 2.9% | -2.97 [-7.53, 1.59] |
| **Subtotal (95% CI)** | | | **489** | | | **869** | **25.4%** | **1.22 [-1.32, 3.76]** |
| Heterogeneity: Tau² = 9.04; Chi² = 23.75, df = 7 (P = 0.001); I² = 71% | | | | | | | | |
| Test for overall effect: Z = 0.94 (P = 0.35) | | | | | | | | |
| | | | | | | | | |
| **5.2.2 European/West Asians** | | | | | | | | |
| 2007A. Orr-Urtreger(1) | 57.7 | 11.6 | 51 | 59.2 | 11.6 | 293 | 3.6% | -1.50 [-4.95, 1.95] |
| 2008Gan-Or, Z. | 56.9 | 11.4 | 56 | 60.7 | 11.5 | 283 | 3.7% | -3.80 [-7.07, -0.53] |
| 2012Gilad Yahalom | 58.1 | 11.2 | 33 | 60.9 | 13.4 | 316 | 3.2% | -2.80 [-6.90, 1.30] |
| 2013Greenbaum, L. | 49.5 | 6.8 | 13 | 49.15 | 6.6 | 26 | 3.0% | 0.35 [-4.13, 4.83] |
| 2013Maria Sierra | 62.5 | 11.05 | 29 | 62.98 | 9.67 | 50 | 2.8% | -0.48 [-5.31, 4.35] |
| 2014Gilad Yahalom | 58.2 | 10.1 | 60 | 61.9 | 12 | 345 | 4.0% | -3.70 [-6.55, -0.85] |
| **Subtotal (95% CI)** | | | **242** | | | **1313** | **20.1%** | **-2.44 [-3.93, -0.96]** |
| Heterogeneity: Tau² = 0.00; Chi² = 3.85, df = 5 (P = 0.57); I² = 0% | | | | | | | | |
| Test for overall effect: Z = 3.22 (P = 0.001) | | | | | | | | |
| | | | | | | | | |
| **5.2.3 Hispanics** | | | | | | | | |
| 2009Mata, I. F.(1) | 68 | 0 | 1 | 59.1 | 11.6 | 239 | | Not estimable |
| 2009Mata, I. F.(2) | 50.2 | 9.3 | 5 | 55.4 | 14 | 119 | 1.4% | -5.20 [-13.73, 3.33] |
| 2013Emilia Mabel Gatto | 66.67 | 4.04 | 3 | 58.78 | 12.45 | 52 | 2.3% | 7.89 [2.20, 13.58] |
| 2017da Silva, C. P. | 46.76 | 9.14 | 17 | 55.02 | 12.36 | 93 | 2.7% | -8.26 [-13.28, -3.24] |
| **Subtotal (95% CI)** | | | **26** | | | **503** | **6.4%** | **-1.78 [-12.61, 9.05]** |
| Heterogeneity: Tau² = 80.56; Chi² = 18.12, df = 2 (P = 0.0001); I² = 89% | | | | | | | | |
| Test for overall effect: Z = 0.32 (P = 0.75) | | | | | | | | |
| | | | | | | | | |
| **5.2.4 Mixed** | | | | | | | | |
| 2006L.N. Clark | 52.1 | 11.4 | 28 | 53.9 | 13.7 | 476 | 3.0% | -1.80 [-6.20, 2.60] |
| 2009RN Alcalay | 42.9 | 5 | 26 | 41.7 | 6.7 | 665 | 4.5% | 1.20 [-0.79, 3.19] |
| 2011C. Marras | 59 | 11 | 25 | 54 | 14 | 78 | 2.5% | 5.00 [-0.31, 10.31] |
| 2011R. Saunders-Pullman | 64.7 | 9.8 | 31 | 63.4 | 7.8 | 30 | 3.0% | 1.30 [-3.14, 5.74] |
| 2013Roy N. Alcalay | 60 | 10 | 97 | 62 | 10.5 | 391 | 4.3% | -2.00 [-4.25, 0.25] |
| 2015Alcalay, R. N. | 57.2 | 10.8 | 116 | 58.2 | 10.8 | 120 | 4.0% | -1.00 [-3.76, 1.76] |
| 2015Marder, K. | 58.4 | 11 | 129 | 61.6 | 10.4 | 345 | 4.4% | -3.20 [-5.39, -1.01] |
| 2015Saunders-Pullman, R. | 57.4 | 11 | 142 | 58 | 10.9 | 144 | 4.2% | -0.60 [-3.14, 1.94] |
| 2016Marras, C. | 55.65 | 11.6 | 506 | 58.69 | 12.5 | 773 | 4.8% | -3.04 [-4.38, -1.70] |
| 2016Pal, G. D. | 47.5 | 2.4 | 4 | 41.2 | 5.3 | 72 | 4.1% | 6.30 [3.65, 8.95] |
| 2017San Luciano, M. | 55.7 | 11.3 | 530 | 58.7 | 12.5 | 759 | 4.8% | -3.00 [-4.31, -1.69] |
| 2018Saunders-Pullman, R. | 60 | 9.3 | 144 | 61.5 | 10.7 | 401 | 4.6% | -1.50 [-3.35, 0.35] |
| **Subtotal (95% CI)** | | | **1778** | | | **4254** | **48.1%** | **-0.52 [-2.08, 1.03]** |
| Heterogeneity: Tau² = 5.63; Chi² = 60.79, df = 11 (P < 0.00001); I² = 82% | | | | | | | | |
| Test for overall effect: Z = 0.66 (P = 0.51) | | | | | | | | |
| | | | | | | | | |
| **Total (95% CI)** | | | **2535** | | | **6939** | **100.0%** | **-0.46 [-1.64, 0.72]** |
| Heterogeneity: Tau² = 7.07; Chi² = 123.49, df = 28 (P < 0.00001); I² = 77% | | | | | | | | |
| Test for overall effect: Z = 0.76 (P = 0.44) | | | | | | | | |
| Test for subgroup differences: Chi² = 6.88, df = 3 (P = 0.08), I² = 56.4% | | | | | | | | |

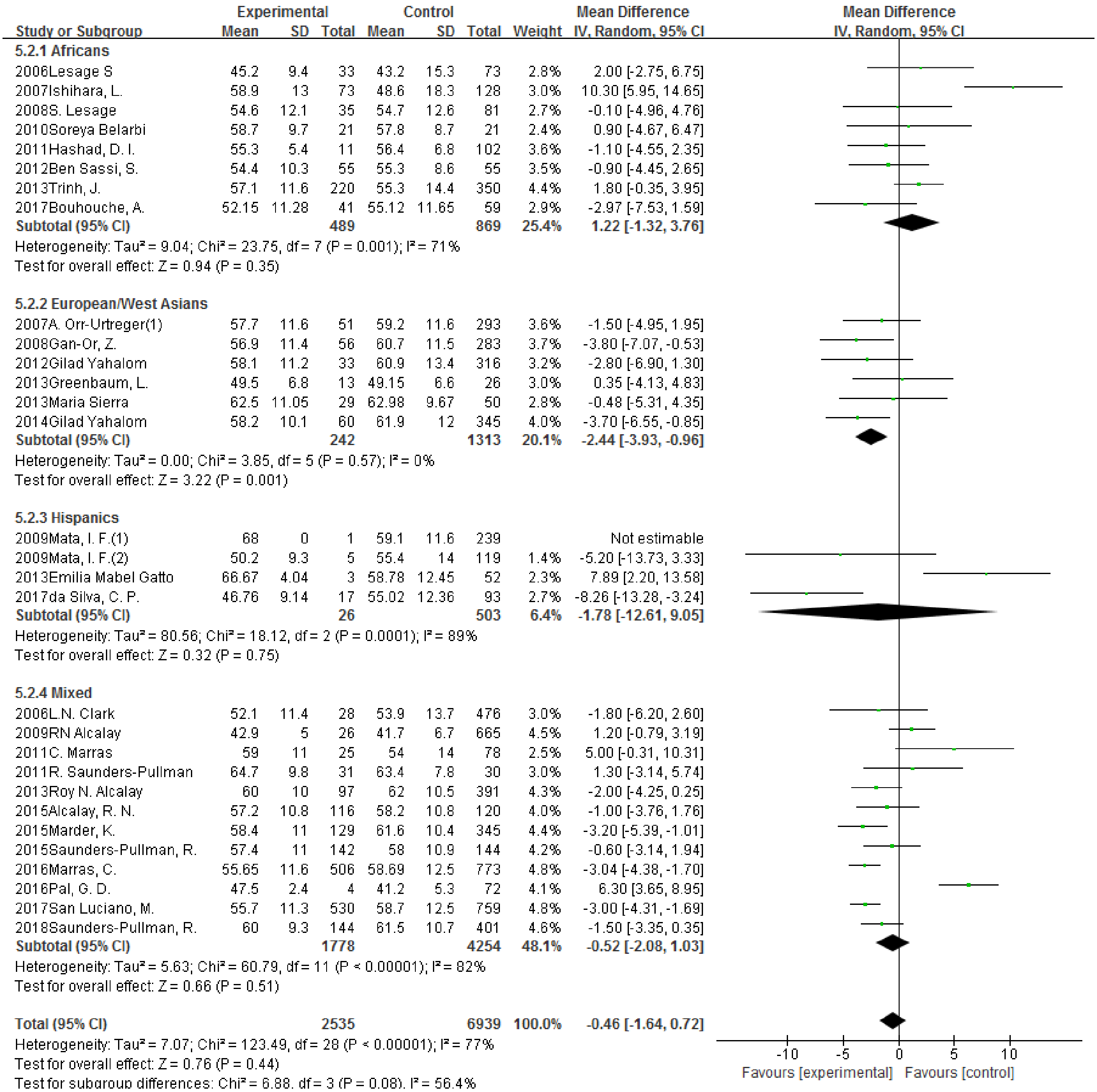


## 1.2 Age at onset of G2019S by ethnicity

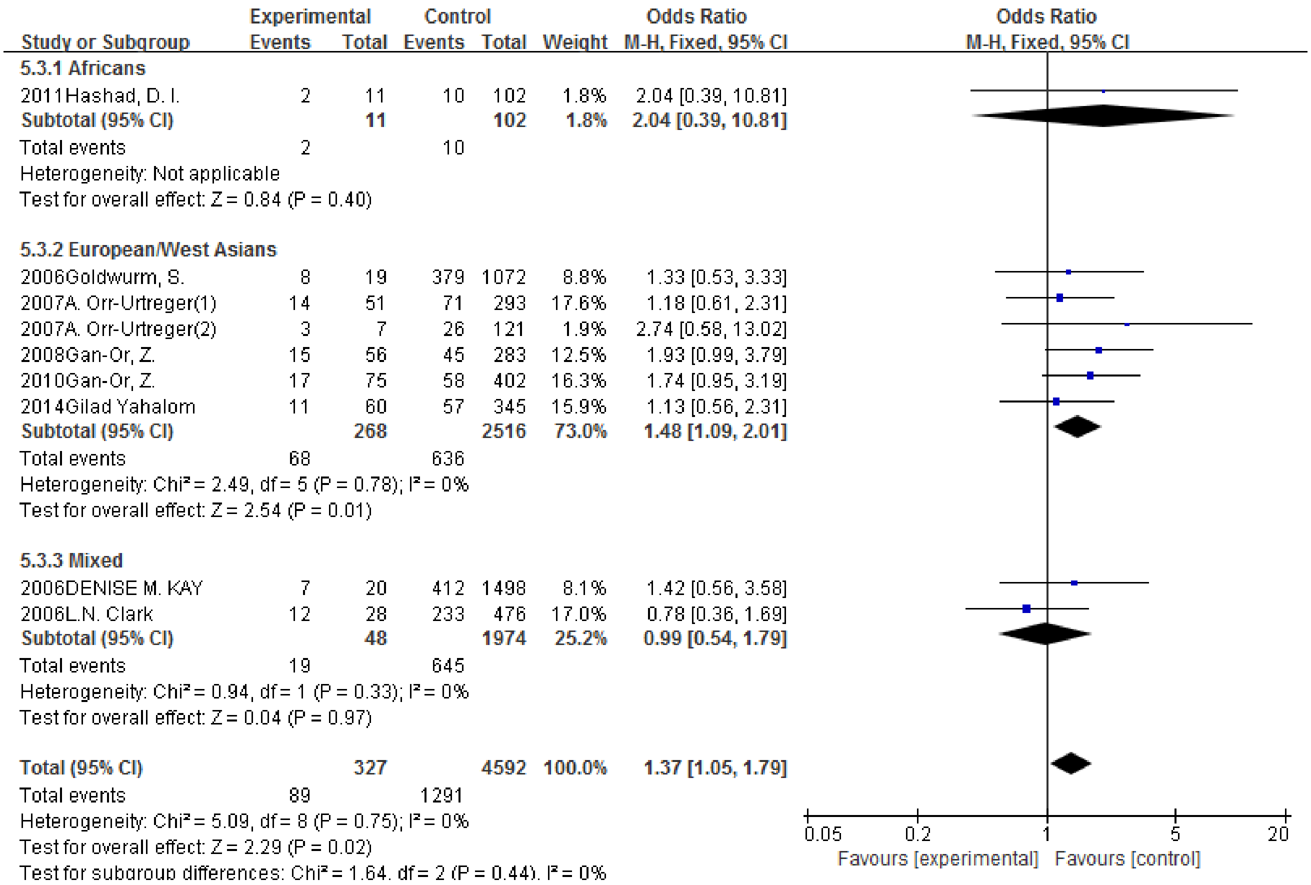


1.3 EOPD of G2019S by ethnicity

| Study or Subgroup | Experimental Events | Experimental Total | Control Events | Control Total | Weight | Odds Ratio M-H, Fixed, 95% CI |
|---|---|---|---|---|---|---|
| **5.4.1 Africans** | | | | | | |
| 2006Lesage S | 13 | 33 | 41 | 73 | 1.4% | 0.51 [0.22, 1.17] |
| 2008Mary M Hulihan | 39 | 72 | 77 | 166 | 1.9% | 1.37 [0.78, 2.38] |
| 2008S. Lesage | 21 | 47 | 53 | 89 | 1.8% | 0.55 [0.27, 1.12] |
| 2010Soreya Belarbi | 11 | 23 | 34 | 48 | 1.0% | 0.38 [0.14, 1.06] |
| 2012Ben Sassi, S. | 34 | 55 | 30 | 55 | 1.0% | 1.35 [0.63, 2.89] |
| 2013Trinh, J. | 124 | 220 | 187 | 350 | 5.7% | 1.13 [0.80, 1.58] |
| 2015Nabli, F. | 32 | 58 | 29 | 54 | 1.2% | 1.06 [0.50, 2.23] |
| 2017Bouhouche, A. | 19 | 41 | 37 | 59 | 1.5% | 0.51 [0.23, 1.15] |
| **Subtotal (95% CI)** | | **549** | | **894** | **15.8%** | **0.93 [0.75, 1.16]** |
| Total events | 293 | | 488 | | | |
| Heterogeneity: Chi² = 13.24, df = 7 (P = 0.07); I² = 47% | | | | | | |
| Test for overall effect: Z = 0.62 (P = 0.54) | | | | | | |
| **5.4.2 European/West Asians** | | | | | | |
| 2007A. Orr-Urtreger(1) | 25 | 51 | 191 | 293 | 2.6% | 0.51 [0.28, 0.94] |
| 2007A. Orr-Urtreger(2) | 5 | 7 | 82 | 121 | 0.2% | 1.19 [0.22, 6.40] |
| 2008Gan-Or, Z. | 28 | 56 | 187 | 283 | 2.8% | 0.51 [0.29, 0.92] |
| 2010Gan-Or, Z. | 37 | 75 | 262 | 402 | 3.8% | 0.52 [0.32, 0.86] |
| 2010Vicki Shanker | 10 | 21 | 10 | 21 | 0.5% | 1.00 [0.30, 3.36] |
| 2013Greenbaum, L. | 8 | 13 | 17 | 26 | 0.4% | 0.85 [0.21, 3.36] |
| 2013Maria Sierra | 16 | 29 | 29 | 50 | 0.9% | 0.89 [0.35, 2.24] |
| 2013Mirelman, A. | 27 | 50 | 24 | 50 | 1.0% | 1.27 [0.58, 2.79] |
| 2014Carles Gaig | 15 | 33 | 15 | 33 | 0.7% | 1.00 [0.38, 2.64] |
| 2014Gilad Yahalom | 30 | 60 | 211 | 345 | 2.9% | 0.64 [0.37, 1.10] |
| 2015Somme, J. H. | 6 | 12 | 18 | 27 | 0.5% | 0.50 [0.13, 2.00] |
| 2015Vilas, D. | 12 | 26 | 21 | 31 | 0.9% | 0.41 [0.14, 1.20] |
| 2016Dagan, E. | 9 | 22 | 114 | 189 | 1.3% | 0.46 [0.19, 1.12] |
| **Subtotal (95% CI)** | | **455** | | **1871** | **18.6%** | **0.63 [0.51, 0.78]** |
| Total events | 228 | | 1181 | | | |
| Heterogeneity: Chi² = 8.50, df = 12 (P = 0.74); I² = 0% | | | | | | |
| Test for overall effect: Z = 4.18 (P < 0.0001) | | | | | | |
| **5.4.3 Hispanics** | | | | | | |
| 2009Mata, I. F.(1) | 1 | 1 | 121 | 239 | 0.0% | 2.93 [0.12, 72.54] |
| 2009Mata, I. F.(2) | 3 | 5 | 58 | 119 | 0.2% | 1.58 [0.25, 9.78] |
| 2013Emilia Mabel Gatto | 2 | 3 | 23 | 52 | 0.1% | 2.52 [0.22, 29.58] |
| 2017da Silva, C. P. | 7 | 17 | 57 | 93 | 0.9% | 0.44 [0.15, 1.27] |
| **Subtotal (95% CI)** | | **26** | | **503** | **1.2%** | **0.82 [0.37, 1.80]** |
| Total events | 13 | | 259 | | | |
| Heterogeneity: Chi² = 3.22, df = 3 (P = 0.36); I² = 7% | | | | | | |
| Test for overall effect: Z = 0.50 (P = 0.62) | | | | | | |
| **5.4.4 Mixed** | | | | | | |
| 2006DENISE M. KAY | 13 | 20 | 1010 | 1498 | 0.9% | 0.90 [0.36, 2.26] |
| 2006L.N. Clark | 11 | 28 | 293 | 476 | 1.8% | 0.40 [0.19, 0.88] |
| 2008Latourelle, J. C. | 32 | 60 | 549 | 965 | 2.7% | 0.87 [0.51, 1.46] |
| 2009RN Alcalay | 14 | 26 | 411 | 665 | 1.3% | 0.72 [0.33, 1.58] |
| 2011C. Marras | 12 | 25 | 49 | 84 | 1.1% | 0.66 [0.27, 1.62] |
| 2011R. Saunders-Pullman | 15 | 31 | 17 | 30 | 0.8% | 0.72 [0.26, 1.97] |
| 2013Roy N. Alcalay | 47 | 97 | 243 | 391 | 4.5% | 0.57 [0.37, 0.90] |
| 2014Rachel Saunders-Pullman | 70 | 126 | 78 | 126 | 3.2% | 0.77 [0.47, 1.27] |
| 2015Alcalay, R. N. | 64 | 116 | 76 | 120 | 3.1% | 0.71 [0.42, 1.20] |
| 2015Marder, K. | 64 | 129 | 215 | 345 | 5.4% | 0.60 [0.40, 0.90] |
| 2015Saunders-Pullman, R. | 77 | 142 | 93 | 144 | 3.9% | 0.65 [0.40, 1.04] |
| 2016Marras, C. | 266 | 516 | 465 | 790 | 16.2% | 0.74 [0.59, 0.93] |
| 2017San Luciano, M. | 276 | 530 | 448 | 759 | 16.1% | 0.75 [0.60, 0.94] |
| 2018Saunders-Pullman, R. | 72 | 144 | 144 | 401 | 3.5% | 1.78 [1.21, 2.62] |
| **Subtotal (95% CI)** | | **1990** | | **6794** | **64.4%** | **0.77 [0.69, 0.86]** |
| Total events | 1033 | | 4091 | | | |
| Heterogeneity: Chi² = 25.29, df = 13 (P = 0.02); I² = 49% | | | | | | |
| Test for overall effect: Z = 4.66 (P < 0.00001) | | | | | | |
| **Total (95% CI)** | | **3020** | | **10062** | **100.0%** | **0.77 [0.70, 0.84]** |
| Total events | 1567 | | 6019 | | | |
| Heterogeneity: Chi² = 56.77, df = 38 (P = 0.03); I² = 33% | | | | | | |
| Test for overall effect: Z = 5.77 (P < 0.00001) | | | | | | |
| Test for subgroup differences: Chi² = 6.45, df = 3 (P = 0.09), I² = 53.5% | | | | | | |

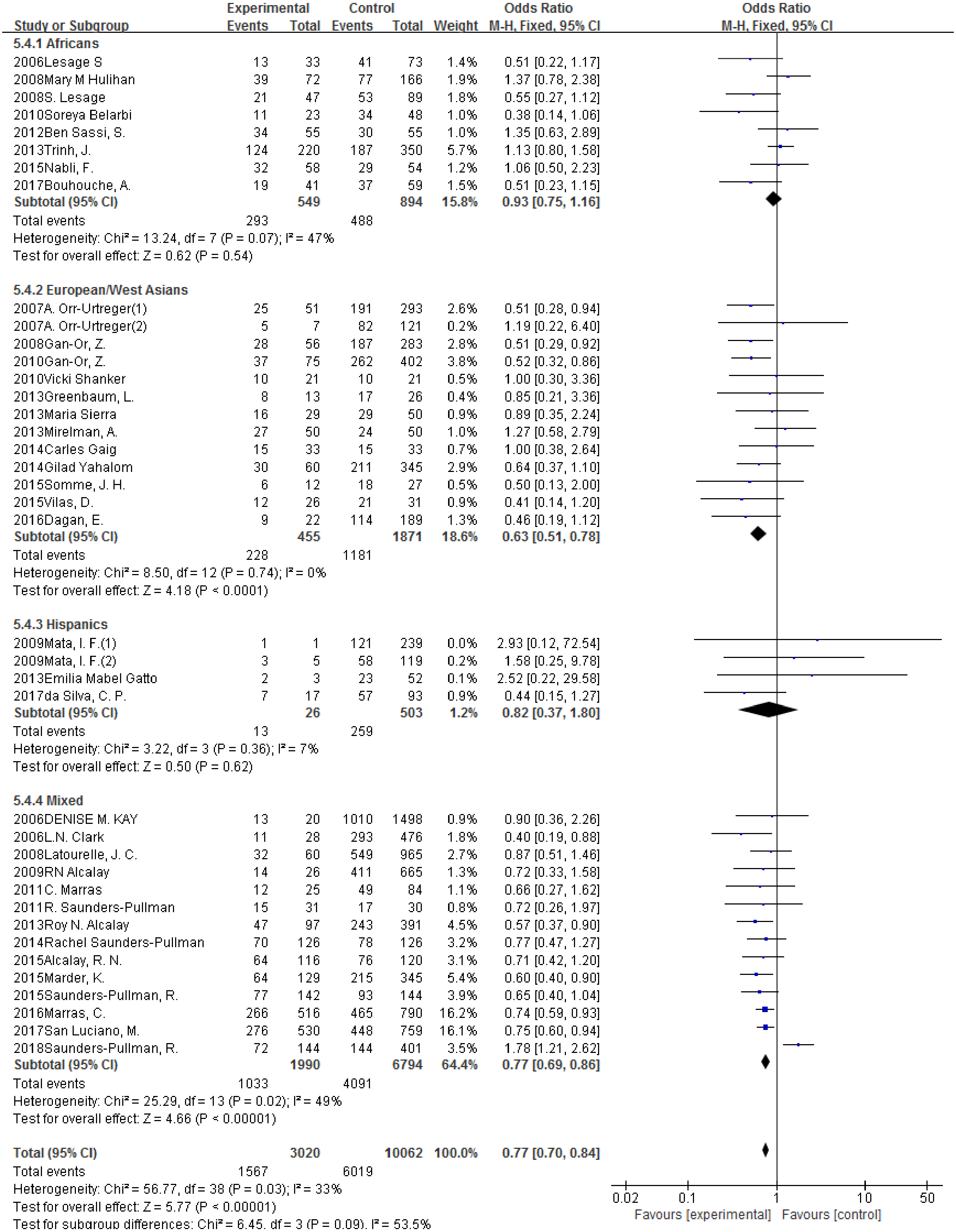

## 1.4 Gender-male of G2019S by ethnicity

| Study or Subgroup | Experimental Events | Experimental Total | Control Events | Control Total | Weight | Odds Ratio M-H, Fixed, 95% CI |
|---|---|---|---|---|---|---|
| **5.5.1 Africans** | | | | | | |
| 2017Bouhouche, A. | 22 | 41 | 11 | 59 | 2.2% | 5.05 [2.06, 12.40] |
| **Subtotal (95% CI)** | | **41** | | **59** | **2.2%** | **5.05 [2.06, 12.40]** |
| Total events | 22 | | 11 | | | |
| Heterogeneity: Not applicable | | | | | | |
| Test for overall effect: Z = 3.54 (P = 0.0004) | | | | | | |
| **5.5.2 European/West Asians** | | | | | | |
| 2005Jose Miguel Bras | 6 | 11 | 20 | 117 | 0.8% | 5.82 [1.62, 20.95] |
| 2006Gaig, C. | 6 | 13 | 85 | 286 | 2.1% | 2.03 [0.66, 6.21] |
| 2006Goldwurm, S. | 10 | 19 | 226 | 1073 | 2.0% | 4.16 [1.67, 10.37] |
| 2007A. Orr-Urtreger(1) | 25 | 51 | 71 | 293 | 5.6% | 3.01 [1.63, 5.54] |
| 2007A. Orr-Urtreger(2) | 3 | 7 | 32 | 121 | 1.0% | 2.09 [0.44, 9.83] |
| 2008Gan-Or, Z. | 24 | 56 | 64 | 283 | 6.3% | 2.57 [1.41, 4.67] |
| 2010Gan-Or, Z. | 34 | 75 | 85 | 402 | 7.7% | 3.09 [1.85, 5.17] |
| 2012Gilad Yahalom | 16 | 33 | 70 | 316 | 3.6% | 3.31 [1.59, 6.88] |
| 2014Gilad Yahalom | 26 | 60 | 73 | 345 | 6.4% | 2.85 [1.61, 5.05] |
| 2016Dagan, E. | 8 | 21 | 32 | 177 | 2.2% | 2.79 [1.07, 7.28] |
| **Subtotal (95% CI)** | | **346** | | **3413** | **37.7%** | **2.98 [2.36, 3.76]** |
| Total events | 158 | | 758 | | | |
| Heterogeneity: Chi² = 2.61, df = 9 (P = 0.98); I² = 0% | | | | | | |
| Test for overall effect: Z = 9.17 (P < 0.00001) | | | | | | |
| **5.5.3 Hispanics** | | | | | | |
| 2009Mata, I. F.(1) | 0 | 1 | 4 | 239 | 0.0% | 17.44 [0.62, 489.20] |
| 2009Mata, I. F.(2) | 1 | 5 | 28 | 119 | 0.9% | 0.81 [0.09, 7.57] |
| 2013Emilia Mabel Gatto | 3 | 3 | 13 | 50 | 0.1% | 19.44 [0.94, 401.55] |
| 2017da Silva, C. P. | 9 | 17 | 15 | 93 | 1.1% | 5.85 [1.95, 17.59] |
| **Subtotal (95% CI)** | | **26** | | **501** | **2.2%** | **4.66 [2.02, 10.73]** |
| Total events | 13 | | 60 | | | |
| Heterogeneity: Chi² = 3.97, df = 3 (P = 0.26); I² = 25% | | | | | | |
| Test for overall effect: Z = 3.61 (P = 0.0003) | | | | | | |
| **5.5.4 Mixed** | | | | | | |
| 2006DENISE M. KAY | 11 | 20 | 344 | 1498 | 2.1% | 4.10 [1.69, 9.98] |
| 2006L.N. Clark | 7 | 26 | 54 | 453 | 2.2% | 2.72 [1.09, 6.78] |
| 2008Latourelle, J. C. | 54 | 60 | 845 | 965 | 5.2% | 1.28 [0.54, 3.04] |
| 2009RN Alcalay | 5 | 26 | 99 | 665 | 3.2% | 1.36 [0.50, 3.69] |
| 2013Roy N. Alcalay | 34 | 97 | 69 | 391 | 9.3% | 2.52 [1.54, 4.12] |
| 2017San Luciano, M. | 151 | 530 | 116 | 759 | 35.8% | 2.21 [1.68, 2.90] |
| **Subtotal (95% CI)** | | **759** | | **4731** | **57.8%** | **2.22 [1.79, 2.75]** |
| Total events | 262 | | 1527 | | | |
| Heterogeneity: Chi² = 4.76, df = 5 (P = 0.45); I² = 0% | | | | | | |
| Test for overall effect: Z = 7.32 (P < 0.00001) | | | | | | |
| **Total (95% CI)** | | **1172** | | **8704** | **100.0%** | **2.62 [2.25, 3.06]** |
| Total events | 455 | | 2356 | | | |
| Heterogeneity: Chi² = 18.74, df = 20 (P = 0.54); I² = 0% | | | | | | |
| Test for overall effect: Z = 12.38 (P < 0.00001) | | | | | | |
| Test for subgroup differences: Chi² = 7.37, df = 3 (P = 0.06), I² = 59.3% | | | | | | |

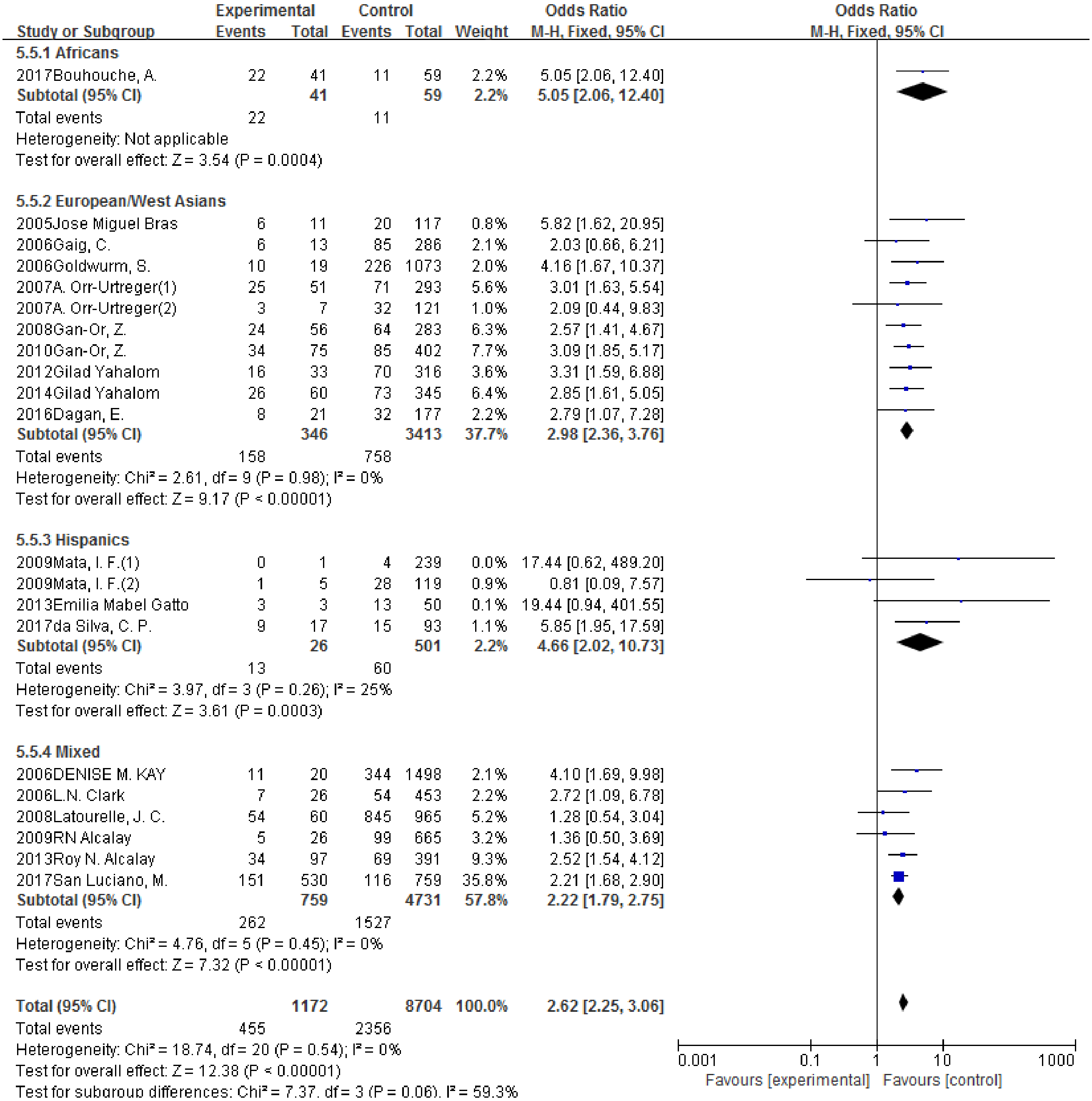


## 1.5 Family history of G2019S by ethnicity

| Study or Subgroup | Experimental Events | Experimental Total | Control Events | Control Total | Weight | Odds Ratio M-H, Random, 95% CI |
|---|---|---|---|---|---|---|
| **5.6.1 Africans** | | | | | | |
| 2006Lesage S | 17 | 31 | 34 | 63 | 20.8% | 1.04 [0.44, 2.46] |
| 2008S. Lesage | 8 | 33 | 16 | 71 | 19.0% | 1.10 [0.42, 2.91] |
| 2011Hashad, D. I. | 11 | 11 | 102 | 102 | | Not estimable |
| 2017Bouhouche, A. | 14 | 41 | 9 | 59 | 19.2% | 2.88 [1.10, 7.52] |
| **Subtotal (95% CI)** | | **116** | | **295** | **59.0%** | **1.46 [0.77, 2.78]** |
| Total events | 50 | | 161 | | | |
| Heterogeneity: Tau² = 0.10; Chi² = 2.86, df = 2 (P = 0.24); I² = 30% | | | | | | |
| Test for overall effect: Z = 1.16 (P = 0.25) | | | | | | |
| **5.6.2 European/West Asians** | | | | | | |
| 2010Gan-Or, Z. | 8 | 75 | 61 | 402 | 22.1% | 0.67 [0.31, 1.46] |
| **Subtotal (95% CI)** | | **75** | | **402** | **22.1%** | **0.67 [0.31, 1.46]** |
| Total events | 8 | | 61 | | | |
| Heterogeneity: Not applicable | | | | | | |
| Test for overall effect: Z = 1.01 (P = 0.31) | | | | | | |
| **5.6.3 Hispanics** | | | | | | |
| 2017da Silva, C. P. | 2 | 17 | 1 | 90 | 6.0% | 11.87 [1.01, 139.18] |
| **Subtotal (95% CI)** | | **17** | | **90** | **6.0%** | **11.87 [1.01, 139.18]** |
| Total events | 2 | | 1 | | | |
| Heterogeneity: Not applicable | | | | | | |
| Test for overall effect: Z = 1.97 (P = 0.05) | | | | | | |
| **5.6.4 Mixed** | | | | | | |
| 2006L.N. Clark | 1 | 28 | 25 | 476 | 8.1% | 0.67 [0.09, 5.12] |
| 2011C. Marras | 0 | 25 | 14 | 84 | 4.7% | 0.10 [0.01, 1.66] |
| **Subtotal (95% CI)** | | **53** | | **560** | **12.8%** | **0.32 [0.05, 2.23]** |
| Total events | 1 | | 39 | | | |
| Heterogeneity: Tau² = 0.48; Chi² = 1.30, df = 1 (P = 0.25); I² = 23% | | | | | | |
| Test for overall effect: Z = 1.15 (P = 0.25) | | | | | | |
| **Total (95% CI)** | | **261** | | **1347** | **100.0%** | **1.16 [0.59, 2.26]** |
| Total events | 61 | | 262 | | | |
| Heterogeneity: Tau² = 0.37; Chi² = 12.31, df = 6 (P = 0.06); I² = 51% | | | | | | |
| Test for overall effect: Z = 0.42 (P = 0.67) | | | | | | |
| Test for subgroup differences: Chi² = 7.45, df = 3 (P = 0.06), I² = 59.7% | | | | | | |

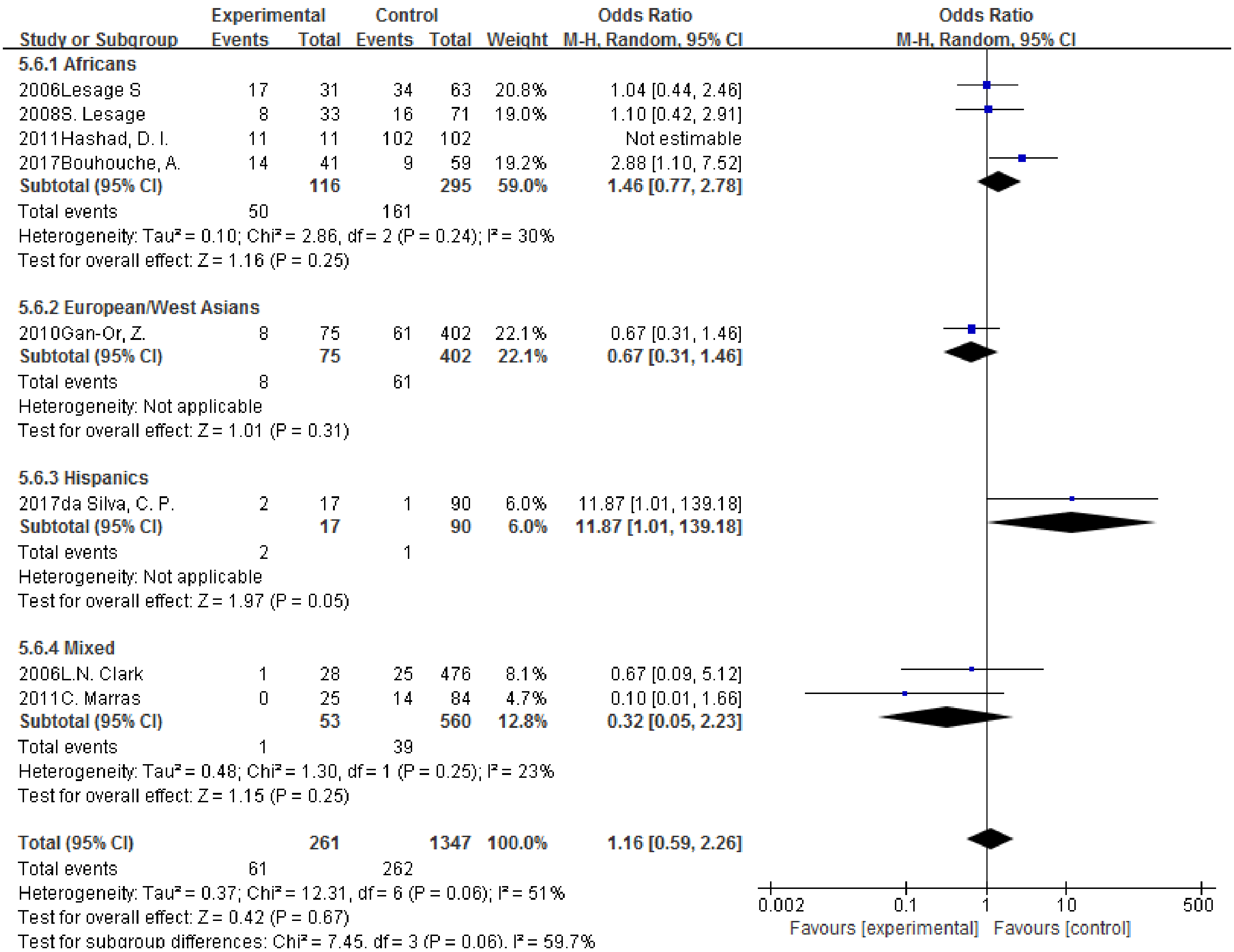


1.6 FS-bradykinesia of G2019S by ethnicity

| Study or Subgroup | Experimental Events | Experimental Total | Control Events | Control Total | Weight | Odds Ratio M-H, Random, 95% CI |
|---|---|---|---|---|---|---|
| **5.7.1 African** | | | | | | |
| 2006Lesage S | 20 | 29 | 39 | 63 | 8.1% | 1.37 [0.54, 3.49] |
| 2008Mary M Hulihan | 56 | 72 | 155 | 166 | 9.2% | 0.25 [0.11, 0.57] |
| 2008S. Lesage | 31 | 33 | 62 | 73 | 4.2% | 2.75 [0.57, 13.18] |
| 2011Hashad, D. I. | 9 | 11 | 87 | 102 | 4.0% | 0.78 [0.15, 3.95] |
| 2013Trinh, J. | 158 | 220 | 265 | 350 | 14.4% | 0.82 [0.56, 1.20] |
| 2017Bouhouche, A. | 11 | 41 | 31 | 59 | 8.9% | 0.33 [0.14, 0.78] |
| **Subtotal (95% CI)** | | **406** | | **813** | **48.7%** | **0.68 [0.37, 1.26]** |
| Total events | 285 | | 639 | | | |
| Heterogeneity: Tau² = 0.35; Chi² = 14.50, df = 5 (P = 0.01); I² = 66% | | | | | | |
| Test for overall effect: Z = 1.22 (P = 0.22) | | | | | | |
| **5.7.2 European/West Asians** | | | | | | |
| 2010Gan-Or, Z. | 37 | 75 | 236 | 402 | 13.0% | 0.68 [0.42, 1.12] |
| **Subtotal (95% CI)** | | **75** | | **402** | **13.0%** | **0.68 [0.42, 1.12]** |
| Total events | 37 | | 236 | | | |
| Heterogeneity: Not applicable | | | | | | |
| Test for overall effect: Z = 1.50 (P = 0.13) | | | | | | |
| **5.7.3 Hispanics** | | | | | | |
| 2017da Silva, C. P. | 9 | 17 | 66 | 90 | 7.1% | 0.41 [0.14, 1.18] |
| **Subtotal (95% CI)** | | **17** | | **90** | **7.1%** | **0.41 [0.14, 1.18]** |
| Total events | 9 | | 66 | | | |
| Heterogeneity: Not applicable | | | | | | |
| Test for overall effect: Z = 1.65 (P = 0.10) | | | | | | |
| **5.7.4 Mixed** | | | | | | |
| 2006L.N. Clark | 9 | 28 | 238 | 476 | 9.3% | 0.47 [0.21, 1.07] |
| 2011C. Marras | 16 | 25 | 31 | 84 | 8.2% | 3.04 [1.20, 7.70] |
| 2013Roy N. Alcalay | 42 | 97 | 214 | 391 | 13.6% | 0.63 [0.40, 0.99] |
| **Subtotal (95% CI)** | | **150** | | **951** | **31.1%** | **0.92 [0.36, 2.38]** |
| Total events | 67 | | 483 | | | |
| Heterogeneity: Tau² = 0.56; Chi² = 10.50, df = 2 (P = 0.005); I² = 81% | | | | | | |
| Test for overall effect: Z = 0.17 (P = 0.86) | | | | | | |
| **Total (95% CI)** | | **648** | | **2256** | **100.0%** | **0.70 [0.49, 1.02]** |
| Total events | 398 | | 1424 | | | |
| Heterogeneity: Tau² = 0.21; Chi² = 26.19, df = 10 (P = 0.003); I² = 62% | | | | | | |
| Test for overall effect: Z = 1.86 (P = 0.06) | | | | | | |
| Test for subgroup differences: Chi² = 1.27, df = 3 (P = 0.74), I² = 0% | | | | | | |

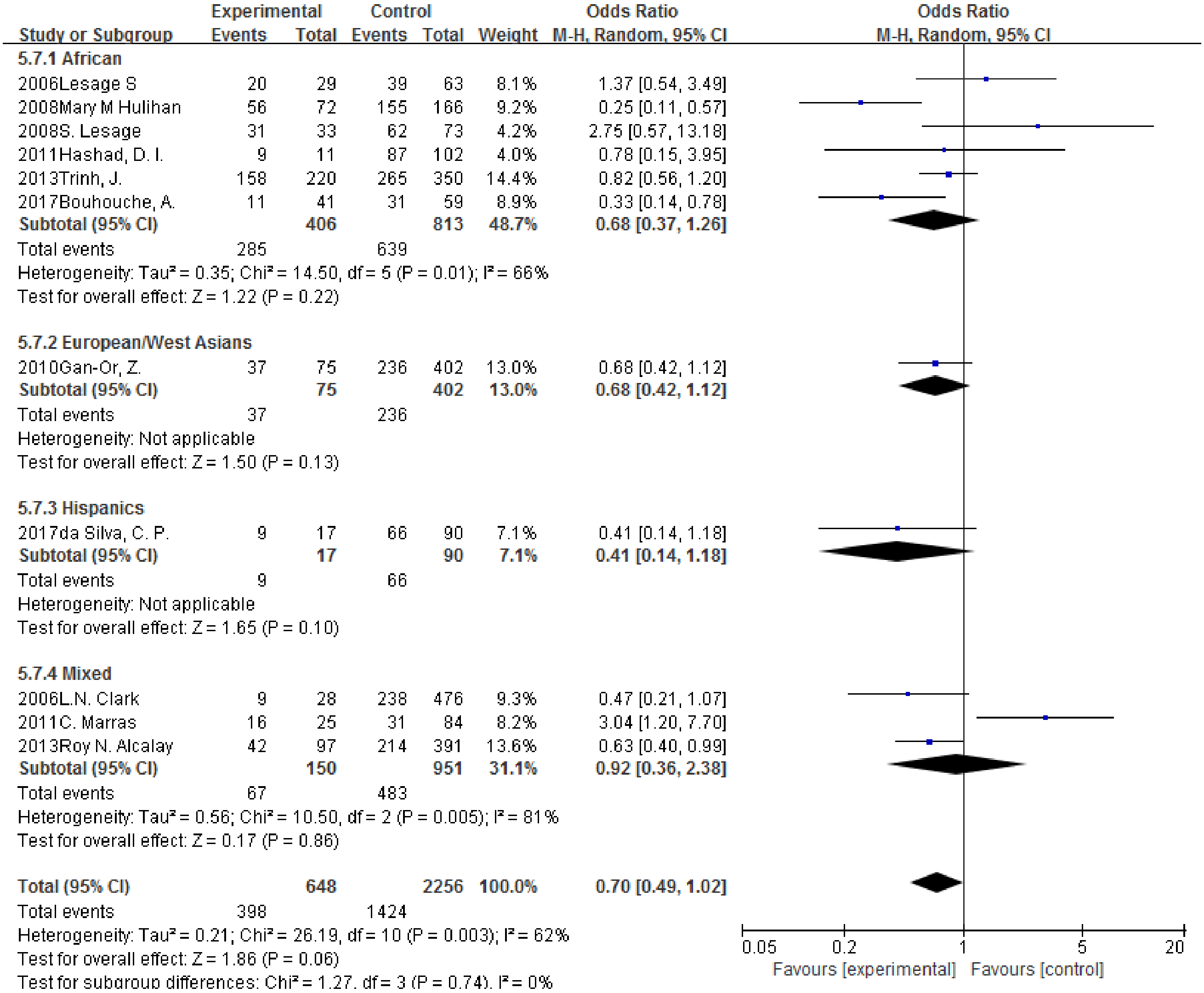


1.7 FS-resting tremor of G2019S by ethnicity

| Study or Subgroup | Experimental Events | Experimental Total | Control Events | Control Total | Weight | Odds Ratio M-H, Fixed, 95% CI |
|---|---|---|---|---|---|---|
| **5.8.1 Africans** | | | | | | |
| 2011Hashad, D. I. | 4 | 11 | 39 | 102 | 12.6% | 0.92 [0.25, 3.36] |
| 2013Trinh, J. | 5 | 220 | 8 | 350 | 15.7% | 0.99 [0.32, 3.08] |
| **Subtotal (95% CI)** | | **231** | | **452** | **28.2%** | **0.96 [0.41, 2.25]** |
| Total events | 9 | | 47 | | | |
| Heterogeneity: Chi² = 0.01, df = 1 (P = 0.93); I² = 0% | | | | | | |
| Test for overall effect: Z = 0.09 (P = 0.93) | | | | | | |
| **5.8.2 European/West Asians** | | | | | | |
| 2010Gan-Or, Z. | 29 | 75 | 105 | 402 | 52.6% | 1.78 [1.07, 2.99] |
| **Subtotal (95% CI)** | | **75** | | **402** | **52.6%** | **1.78 [1.07, 2.99]** |
| Total events | 29 | | 105 | | | |
| Heterogeneity: Not applicable | | | | | | |
| Test for overall effect: Z = 2.20 (P = 0.03) | | | | | | |
| **5.8.3 Hispanics** | | | | | | |
| 2017da Silva, C. P. | 1 | 17 | 4 | 90 | 3.1% | 1.34 [0.14, 12.82] |
| **Subtotal (95% CI)** | | **17** | | **90** | **3.1%** | **1.34 [0.14, 12.82]** |
| Total events | 1 | | 4 | | | |
| Heterogeneity: Not applicable | | | | | | |
| Test for overall effect: Z = 0.26 (P = 0.80) | | | | | | |
| **5.8.4 Mixed** | | | | | | |
| 2006L.N. Clark | 3 | 28 | 40 | 476 | 10.3% | 1.31 [0.38, 4.52] |
| 2011C. Marras | 1 | 25 | 5 | 84 | 5.7% | 0.66 [0.07, 5.91] |
| **Subtotal (95% CI)** | | **53** | | **560** | **16.0%** | **1.08 [0.36, 3.18]** |
| Total events | 4 | | 45 | | | |
| Heterogeneity: Chi² = 0.29, df = 1 (P = 0.59); I² = 0% | | | | | | |
| Test for overall effect: Z = 0.13 (P = 0.89) | | | | | | |
| **Total (95% CI)** | | **376** | | **1504** | **100.0%** | **1.42 [0.96, 2.12]** |
| Total events | 43 | | 201 | | | |
| Heterogeneity: Chi² = 2.05, df = 5 (P = 0.84); I² = 0% | | | | | | |
| Test for overall effect: Z = 1.74 (P = 0.08) | | | | | | |
| Test for subgroup differences: Chi² = 1.80, df = 3 (P = 0.61), I² = 0% | | | | | | |

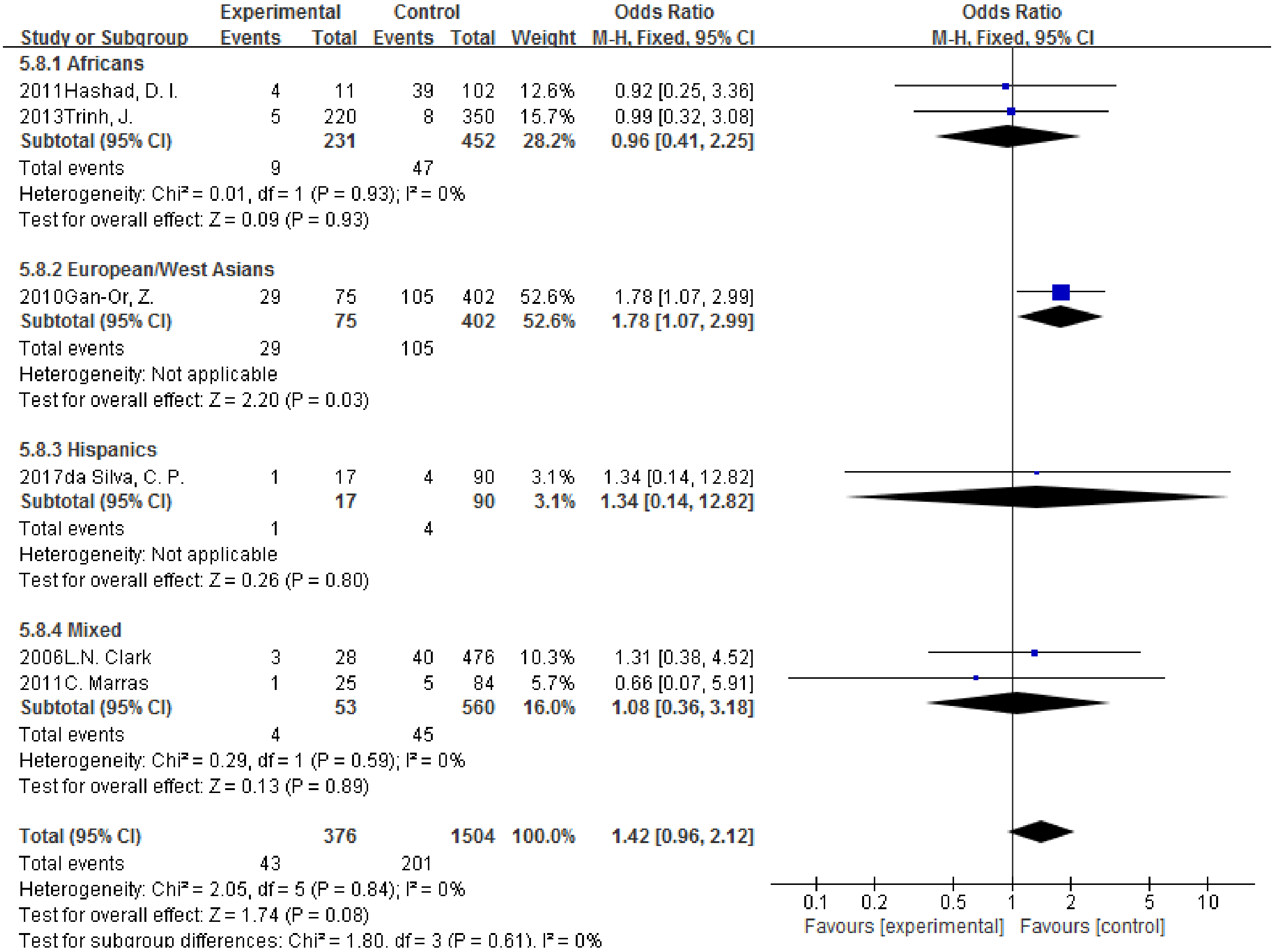


1.8 FS-rigidity of G2019S by ethnicity

| Study or Subgroup | Experimental Events | Experimental Total | Control Events | Control Total | Weight | Odds Ratio M-H, Fixed, 95% CI |
|---|---|---|---|---|---|---|
| **5.9.1 Africans** | | | | | | |
| 2008Mary M Hulihan | 6 | 72 | 21 | 166 | 22.2% | 0.63 [0.24, 1.63] |
| 2013Trinh, J. | 27 | 220 | 26 | 350 | 33.5% | 1.74 [0.99, 3.07] |
| **Subtotal (95% CI)** | | **292** | | **516** | **55.7%** | **1.30 [0.81, 2.09]** |
| Total events | 33 | | 47 | | | |
| Heterogeneity: Chi² = 3.27, df = 1 (P = 0.07); I² = 69% | | | | | | |
| Test for overall effect: Z = 1.08 (P = 0.28) | | | | | | |
| **5.9.2 European/West Asians** | | | | | | |
| 2010Gan-Or, Z. | 14 | 75 | 62 | 402 | 30.2% | 1.26 [0.66, 2.39] |
| **Subtotal (95% CI)** | | **75** | | **402** | **30.2%** | **1.26 [0.66, 2.39]** |
| Total events | 14 | | 62 | | | |
| Heterogeneity: Not applicable | | | | | | |
| Test for overall effect: Z = 0.70 (P = 0.48) | | | | | | |
| **5.9.3 Mixed** | | | | | | |
| 2006L.N. Clark | 1 | 28 | 24 | 476 | 4.9% | 0.70 [0.09, 5.35] |
| 2011C. Marras | 1 | 25 | 11 | 84 | 9.2% | 0.28 [0.03, 2.25] |
| **Subtotal (95% CI)** | | **53** | | **560** | **14.1%** | **0.42 [0.10, 1.82]** |
| Total events | 2 | | 35 | | | |
| Heterogeneity: Chi² = 0.39, df = 1 (P = 0.53); I² = 0% | | | | | | |
| Test for overall effect: Z = 1.15 (P = 0.25) | | | | | | |
| **Total (95% CI)** | | **420** | | **1478** | **100.0%** | **1.16 [0.81, 1.67]** |
| Total events | 49 | | 144 | | | |
| Heterogeneity: Chi² = 5.67, df = 4 (P = 0.23); I² = 29% | | | | | | |
| Test for overall effect: Z = 0.81 (P = 0.42) | | | | | | |
| Test for subgroup differences: Chi² = 2.08, df = 2 (P = 0.35), I² = 4.0% | | | | | | |

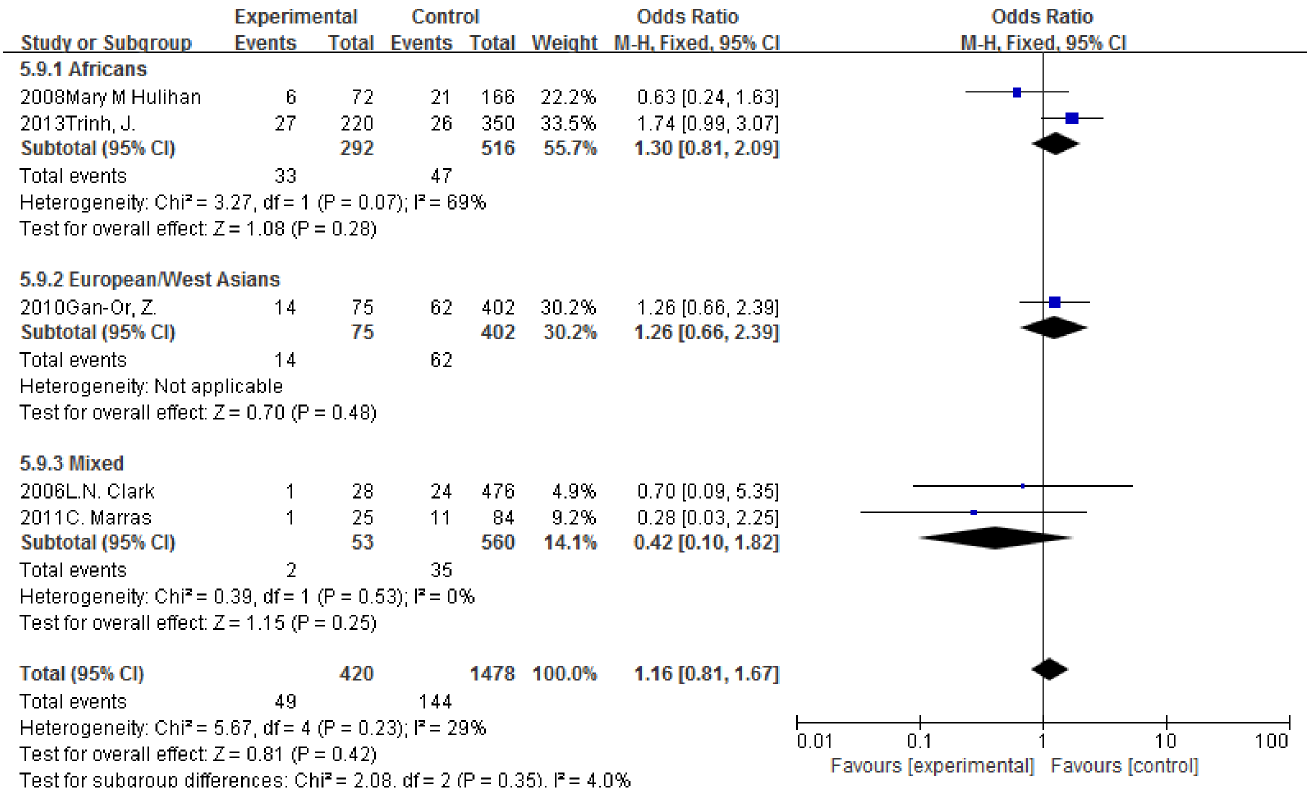


## 1.9 FS-Postural instability or Gait difficulty of G2019S by ethnicity

| Study or Subgroup | Experimental Events | Experimental Total | Control Events | Control Total | Weight | Odds Ratio M-H, Random, 95% CI |
|---|---|---|---|---|---|---|
| **5.10.1 Africans** | | | | | | |
| 2006Lesage S | 2 | 28 | 7 | 62 | 21.7% | 0.60 [0.12, 3.11] |
| 2008Mary M Hulihan | 1 | 72 | 1 | 166 | 7.5% | 2.32 [0.14, 37.68] |
| 2008S. Lesage | 4 | 32 | 2 | 71 | 19.0% | 4.93 [0.85, 28.45] |
| 2013Trinh, J. | 7 | 220 | 7 | 350 | 51.7% | 1.61 [0.56, 4.66] |
| **Subtotal (95% CI)** | | **352** | | **649** | **100.0%** | **1.65 [0.77, 3.55]** |
| Total events | 14 | | 17 | | | |
| Heterogeneity: Tau² = 0.00; Chi² = 3.00, df = 3 (P = 0.39); I² = 0% | | | | | | |
| Test for overall effect: Z = 1.29 (P = 0.20) | | | | | | |
| **Total (95% CI)** | | **352** | | **649** | **100.0%** | **1.65 [0.77, 3.55]** |
| Total events | 14 | | 17 | | | |
| Heterogeneity: Tau² = 0.00; Chi² = 3.00, df = 3 (P = 0.39); I² = 0% | | | | | | |
| Test for overall effect: Z = 1.29 (P = 0.20) | | | | | | |
| Test for subgroup differences: Not applicable | | | | | | |

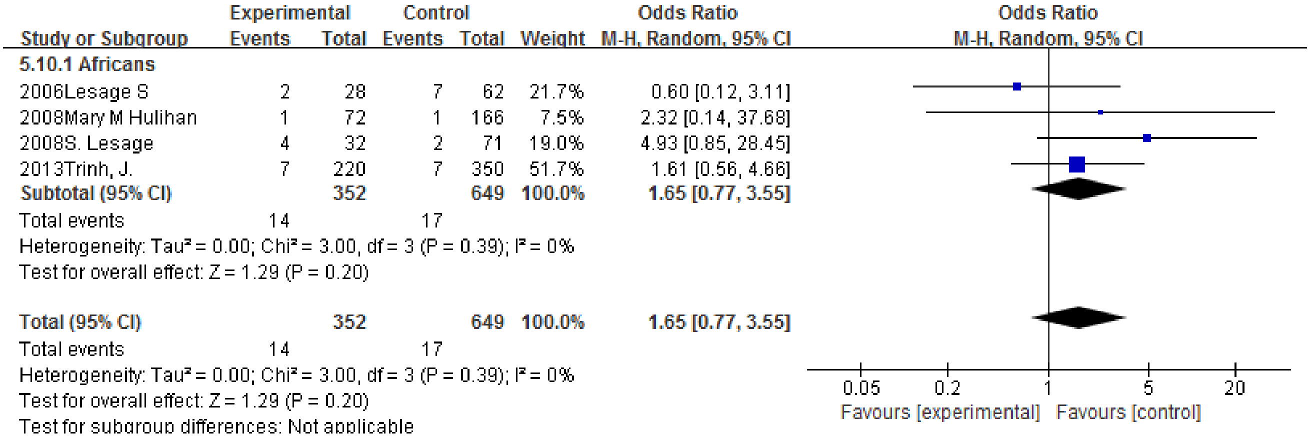


## 1.10 FS-Dystonia of G2019S by ethnicity

| Study or Subgroup | Experimental Events | Experimental Total | Control Events | Control Total | Weight | Odds Ratio M-H, Random, 95% CI |
|---|---|---|---|---|---|---|
| **5.37.1 Africans** | | | | | | |
| 2006Lesage S | 4 | 30 | 13 | 62 | 43.8% | 0.58 [0.17, 1.96] |
| 2008S. Lesage | 1 | 32 | 1 | 64 | 30.3% | 2.03 [0.12, 33.59] |
| **Subtotal (95% CI)** | | **62** | | **126** | **74.2%** | **0.71 [0.23, 2.16]** |
| Total events | 5 | | 14 | | | |
| Heterogeneity: Tau² = 0.00; Chi² = 0.65, df = 1 (P = 0.42); I² = 0% | | | | | | |
| Test for overall effect: Z = 0.61 (P = 0.54) | | | | | | |
| **5.37.2 Mixed** | | | | | | |
| 2011C. Marras | 1 | 25 | 3 | 3 | 25.8% | 0.01 [0.00, 0.26] |
| **Subtotal (95% CI)** | | **25** | | **3** | **25.8%** | **0.01 [0.00, 0.26]** |
| Total events | 1 | | 3 | | | |
| Heterogeneity: Not applicable | | | | | | |
| Test for overall effect: Z = 2.74 (P = 0.006) | | | | | | |
| **Total (95% CI)** | | **87** | | **129** | **100.0%** | **0.29 [0.02, 3.53]** |
| Total events | 6 | | 17 | | | |
| Heterogeneity: Tau² = 3.35; Chi² = 6.51, df = 2 (P = 0.04); I² = 69% | | | | | | |
| Test for overall effect: Z = 0.97 (P = 0.33) | | | | | | |
| Test for subgroup differences: Chi² = 5.82, df = 1 (P = 0.02), I² = 82.8% | | | | | | |

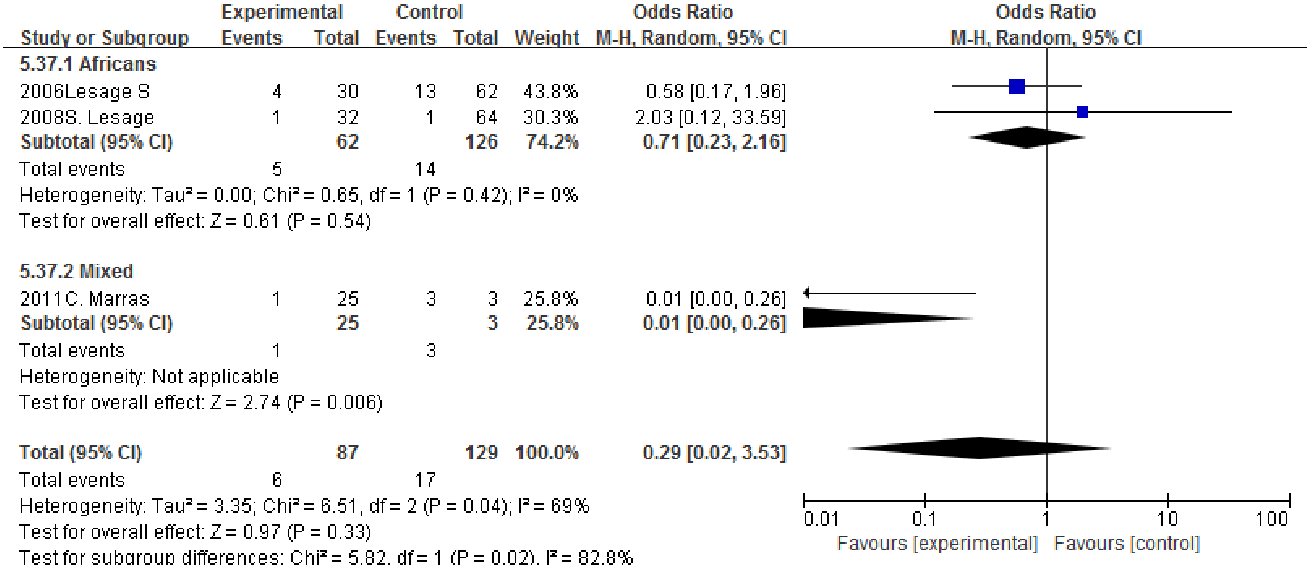


## 1.11 FS-Micrographia of G2019S by ethnicity

| Study or Subgroup | Experimental Events | Experimental Total | Control Events | Control Total | Weight | Odds Ratio M-H, Fixed, 95% CI |
|---|---|---|---|---|---|---|
| **5.11.1 Africans** | | | | | | |
| 2006Lesage S | 31 | 31 | 56 | 61 | 2.1% | 6.13 [0.33, 114.59] |
| 2008S. Lesage | 33 | 34 | 69 | 73 | 4.5% | 1.91 [0.21, 17.80] |
| **Subtotal (95% CI)** | | **65** | | **134** | **6.6%** | **3.25 [0.57, 18.55]** |
| Total events | 64 | | 125 | | | |
| Heterogeneity: Chi² = 0.40, df = 1 (P = 0.53); I² = 0% | | | | | | |
| Test for overall effect: Z = 1.33 (P = 0.18) | | | | | | |
| **5.11.2 European/West Asians** | | | | | | |
| 2007A. Orr-Urtreger(1) | 9 | 51 | 60 | 293 | 51.1% | 0.83 [0.38, 1.80] |
| 2007A. Orr-Urtreger(2) | 1 | 7 | 17 | 121 | 5.6% | 1.02 [0.12, 9.00] |
| 2014Carles Gaig | 33 | 33 | 33 | 33 | | Not estimable |
| **Subtotal (95% CI)** | | **91** | | **447** | **56.7%** | **0.85 [0.41, 1.76]** |
| Total events | 43 | | 110 | | | |
| Heterogeneity: Chi² = 0.03, df = 1 (P = 0.86); I² = 0% | | | | | | |
| Test for overall effect: Z = 0.43 (P = 0.66) | | | | | | |
| **5.11.3 Hispanics** | | | | | | |
| 2017da Silva, C. P. | 17 | 17 | 93 | 93 | | Not estimable |
| **Subtotal (95% CI)** | | **17** | | **93** | | **Not estimable** |
| Total events | 17 | | 93 | | | |
| Heterogeneity: Not applicable | | | | | | |
| Test for overall effect: Not applicable | | | | | | |
| **5.11.4 Mixed** | | | | | | |
| 2006L.N. Clark | 21 | 28 | 379 | 476 | 36.7% | 0.77 [0.32, 1.86] |
| **Subtotal (95% CI)** | | **28** | | **476** | **36.7%** | **0.77 [0.32, 1.86]** |
| Total events | 21 | | 379 | | | |
| Heterogeneity: Not applicable | | | | | | |
| Test for overall effect: Z = 0.59 (P = 0.56) | | | | | | |
| **Total (95% CI)** | | **201** | | **1150** | **100.0%** | **0.98 [0.58, 1.65]** |
| Total events | 145 | | 707 | | | |
| Heterogeneity: Chi² = 2.32, df = 4 (P = 0.68); I² = 0% | | | | | | |
| Test for overall effect: Z = 0.08 (P = 0.94) | | | | | | |
| Test for subgroup differences: Chi² = 2.23, df = 2 (P = 0.33), I² = 10.2% | | | | | | |

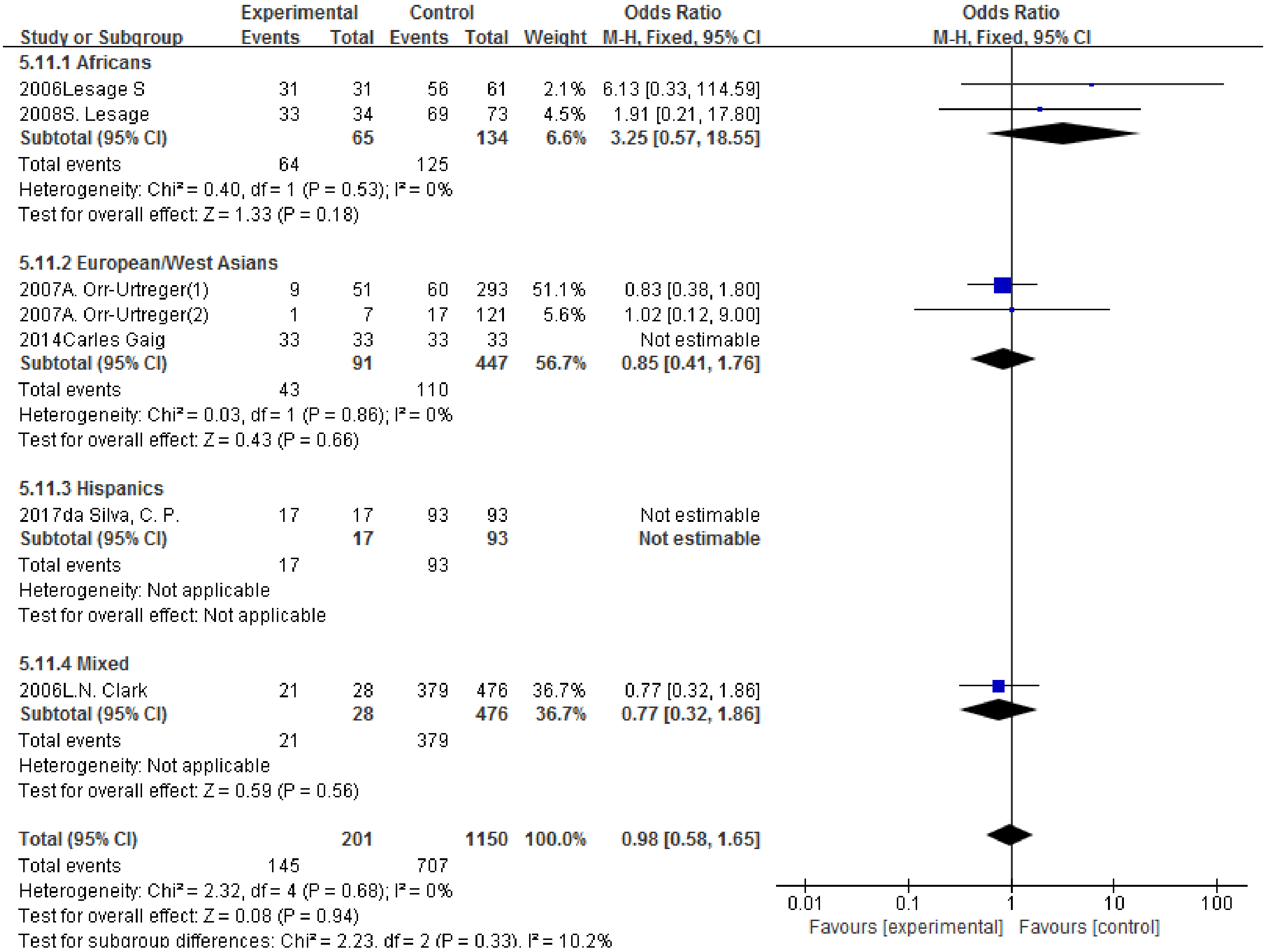


## 1.12 Bradykinesia of G2019S by ethnicity

| Study or Subgroup | Experimental Events | Experimental Total | Control Events | Control Total | Weight | Odds Ratio M-H, Fixed, 95% CI |
|---|---|---|---|---|---|---|
| **5.12.1 Africans** | | | | | | |
| 2006Lesage S | 28 | 32 | 50 | 61 | 6.5% | 1.54 [0.45, 5.29] |
| 2008S. Lesage | 31 | 33 | 62 | 69 | 3.7% | 1.75 [0.34, 8.93] |
| **Subtotal (95% CI)** | | **65** | | **130** | **10.2%** | **1.62 [0.60, 4.32]** |
| Total events | 59 | | 112 | | | |
| Heterogeneity: Chi² = 0.02, df = 1 (P = 0.90); I² = 0% | | | | | | |
| Test for overall effect: Z = 0.96 (P = 0.34) | | | | | | |
| **5.12.2 European/West Asians** | | | | | | |
| 2007A. Orr-Urtreger(1) | 32 | 51 | 164 | 293 | 27.5% | 1.32 [0.72, 2.44] |
| 2007A. Orr-Urtreger(2) | 3 | 7 | 70 | 121 | 6.6% | 0.55 [0.12, 2.55] |
| 2014Carles Gaig | 29 | 33 | 27 | 33 | 5.0% | 1.61 [0.41, 6.34] |
| 2016Dagan, E. | 19 | 21 | 146 | 176 | 4.5% | 1.95 [0.43, 8.83] |
| **Subtotal (95% CI)** | | **112** | | **623** | **43.7%** | **1.30 [0.80, 2.13]** |
| Total events | 83 | | 407 | | | |
| Heterogeneity: Chi² = 1.59, df = 3 (P = 0.66); I² = 0% | | | | | | |
| Test for overall effect: Z = 1.06 (P = 0.29) | | | | | | |
| **5.12.3 Hispanics** | | | | | | |
| 2013Emilia Mabel Gatto | 2 | 3 | 30 | 48 | 1.8% | 1.20 [0.10, 14.19] |
| 2017da Silva, C. P. | 14 | 17 | 85 | 92 | 7.1% | 0.38 [0.09, 1.66] |
| **Subtotal (95% CI)** | | **20** | | **140** | **8.9%** | **0.55 [0.16, 1.93]** |
| Total events | 16 | | 115 | | | |
| Heterogeneity: Chi² = 0.61, df = 1 (P = 0.43); I² = 0% | | | | | | |
| Test for overall effect: Z = 0.93 (P = 0.35) | | | | | | |
| **5.12.4 Mixed** | | | | | | |
| 2006L.N. Clark | 19 | 28 | 356 | 476 | 19.3% | 0.71 [0.31, 1.62] |
| 2009RN Alcalay | 11 | 26 | 271 | 665 | 17.9% | 1.07 [0.48, 2.36] |
| **Subtotal (95% CI)** | | **54** | | **1141** | **37.2%** | **0.88 [0.50, 1.57]** |
| Total events | 30 | | 627 | | | |
| Heterogeneity: Chi² = 0.48, df = 1 (P = 0.49); I² = 0% | | | | | | |
| Test for overall effect: Z = 0.43 (P = 0.67) | | | | | | |
| **Total (95% CI)** | | **251** | | **2034** | **100.0%** | **1.11 [0.80, 1.55]** |
| Total events | 188 | | 1261 | | | |
| Heterogeneity: Chi² = 5.68, df = 9 (P = 0.77); I² = 0% | | | | | | |
| Test for overall effect: Z = 0.62 (P = 0.54) | | | | | | |
| Test for subgroup differences: Chi² = 2.79, df = 3 (P = 0.43), I² = 0% | | | | | | |

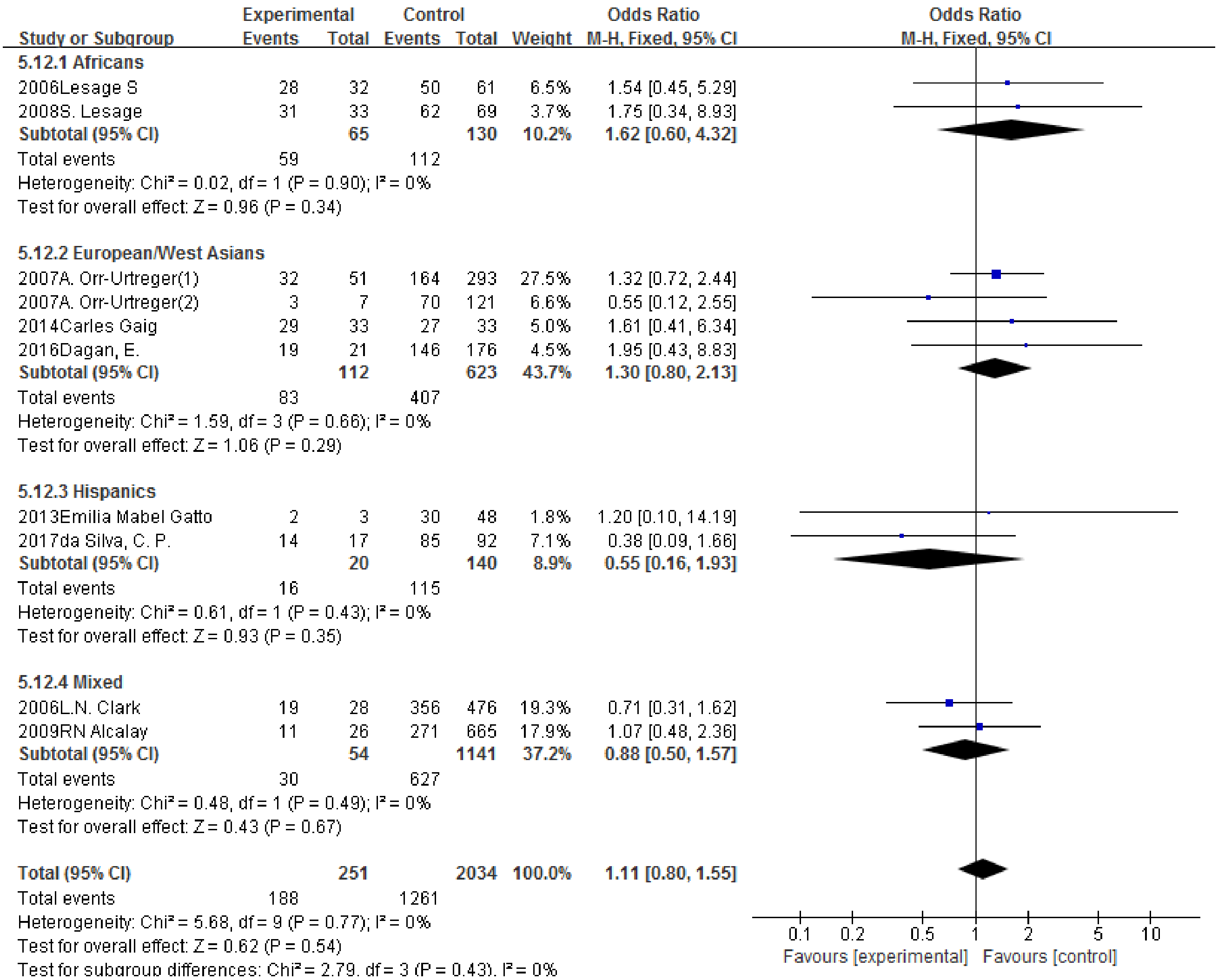


## 1.13 Resting tremor of G2019S by ethnicity

| Study or Subgroup | Experimental Events | Experimental Total | Control Events | Control Total | Weight | Odds Ratio M-H, Fixed, 95% CI |
|---|---|---|---|---|---|---|
| **5.13.1 Africans** | | | | | | |
| 2006Lesage S | 31 | 31 | 55 | 62 | 1.8% | 8.51 [0.47, 154.09] |
| 2008S. Lesage | 33 | 34 | 69 | 72 | 4.0% | 1.43 [0.14, 14.32] |
| **Subtotal (95% CI)** | | **65** | | **134** | **5.8%** | **3.63 [0.65, 20.28]** |
| Total events | 64 | | 124 | | | |
| Heterogeneity: Chi² = 0.96, df = 1 (P = 0.33); I² = 0% | | | | | | |
| Test for overall effect: Z = 1.47 (P = 0.14) | | | | | | |
| **5.13.2 European/West Asians** | | | | | | |
| 2007A. Orr-Urtreger(1) | 16 | 51 | 81 | 293 | 51.1% | 1.20 [0.63, 2.28] |
| 2007A. Orr-Urtreger(2) | 4 | 7 | 38 | 121 | 5.5% | 2.91 [0.62, 13.66] |
| 2016Dagan, E. | 15 | 21 | 150 | 187 | 26.8% | 0.62 [0.22, 1.70] |
| **Subtotal (95% CI)** | | **79** | | **601** | **83.4%** | **1.12 [0.67, 1.89]** |
| Total events | 35 | | 269 | | | |
| Heterogeneity: Chi² = 2.84, df = 2 (P = 0.24); I² = 30% | | | | | | |
| Test for overall effect: Z = 0.44 (P = 0.66) | | | | | | |
| **5.13.3 Hispanics** | | | | | | |
| 2017da Silva, C. P. | 16 | 17 | 90 | 93 | 5.1% | 0.53 [0.05, 5.45] |
| **Subtotal (95% CI)** | | **17** | | **93** | **5.1%** | **0.53 [0.05, 5.45]** |
| Total events | 16 | | 90 | | | |
| Heterogeneity: Not applicable | | | | | | |
| Test for overall effect: Z = 0.53 (P = 0.60) | | | | | | |
| **5.13.4 Mixed** | | | | | | |
| 2006L.N. Clark | 27 | 28 | 463 | 476 | 5.7% | 0.76 [0.10, 6.01] |
| **Subtotal (95% CI)** | | **28** | | **476** | **5.7%** | **0.76 [0.10, 6.01]** |
| Total events | 27 | | 463 | | | |
| Heterogeneity: Not applicable | | | | | | |
| Test for overall effect: Z = 0.26 (P = 0.79) | | | | | | |
| **Total (95% CI)** | | **189** | | **1304** | **100.0%** | **1.22 [0.77, 1.94]** |
| Total events | 142 | | 946 | | | |
| Heterogeneity: Chi² = 5.40, df = 6 (P = 0.49); I² = 0% | | | | | | |
| Test for overall effect: Z = 0.83 (P = 0.40) | | | | | | |
| Test for subgroup differences: Chi² = 2.29, df = 3 (P = 0.51), I² = 0% | | | | | | |

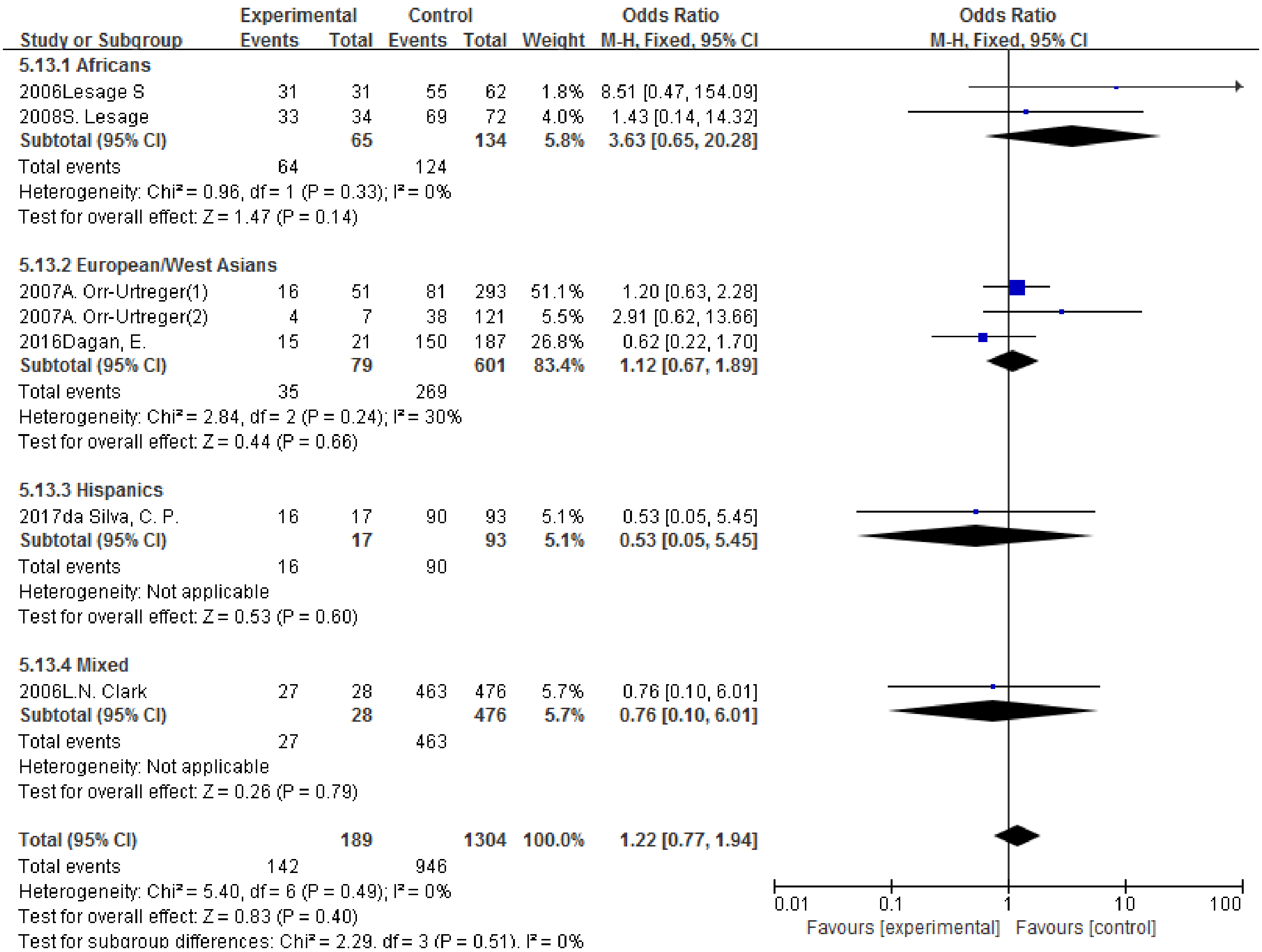


## 1.14 Rigidity of G2019S by ethnicity

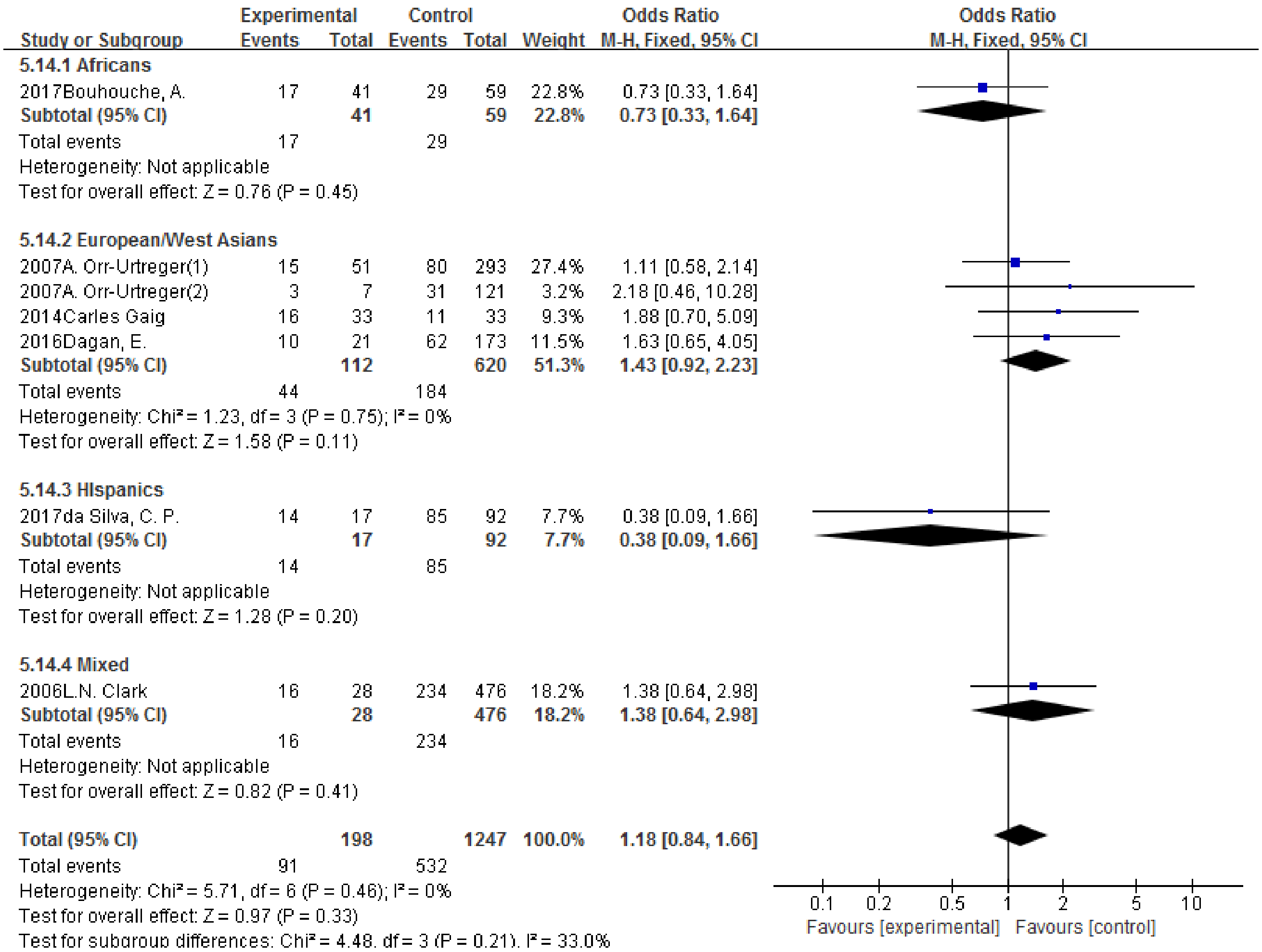


## 1.15 Postural instability or Gait difficulty of G2019S by ethnicity

| Study or Subgroup | Experimental Events | Experimental Total | Control Events | Control Total | Weight | Odds Ratio M-H, Random, 95% CI |
|---|---|---|---|---|---|---|
| **5.15.1 Africans** | | | | | | |
| 2007Ishihara, L. | 7 | 73 | 25 | 128 | 8.0% | 0.44 [0.18, 1.07] |
| 2008Mary M Hulihan | 10 | 72 | 27 | 166 | 9.2% | 0.83 [0.38, 1.82] |
| 2012Ben Sassi, S. | 8 | 55 | 10 | 55 | 6.9% | 0.77 [0.28, 2.11] |
| 2013Trinh, J. | 37 | 220 | 68 | 350 | 14.0% | 0.84 [0.54, 1.30] |
| 2015Nabli, F. | 34 | 58 | 32 | 54 | 9.6% | 0.97 [0.46, 2.07] |
| 2017Bouhouche, A. | 10 | 41 | 10 | 59 | 7.1% | 1.58 [0.59, 4.23] |
| **Subtotal (95% CI)** | | **519** | | **812** | **54.8%** | **0.84 [0.63, 1.12]** |
| Total events | 106 | | 172 | | | |
| Heterogeneity: Tau² = 0.00; Chi² = 3.82, df = 5 (P = 0.58); I² = 0% | | | | | | |
| Test for overall effect: Z = 1.19 (P = 0.24) | | | | | | |
| **5.15.2 Mixed** | | | | | | |
| 2009RN Alcalay | 24 | 26 | 392 | 665 | 4.2% | 8.36 [1.96, 35.65] |
| 2013Roy N. Alcalay | 51 | 97 | 164 | 391 | 13.9% | 1.53 [0.98, 2.40] |
| 2015Alcalay, R. N. | 73 | 111 | 60 | 118 | 12.6% | 1.86 [1.09, 3.16] |
| 2018Saunders-Pullman, R. | 97 | 144 | 218 | 401 | 14.6% | 1.73 [1.16, 2.58] |
| **Subtotal (95% CI)** | | **378** | | **1575** | **45.2%** | **1.85 [1.30, 2.64]** |
| Total events | 245 | | 834 | | | |
| Heterogeneity: Tau² = 0.05; Chi² = 4.95, df = 3 (P = 0.18); I² = 39% | | | | | | |
| Test for overall effect: Z = 3.40 (P = 0.0007) | | | | | | |
| **Total (95% CI)** | | **897** | | **2387** | **100.0%** | **1.23 [0.88, 1.73]** |
| Total events | 351 | | 1006 | | | |
| Heterogeneity: Tau² = 0.16; Chi² = 23.11, df = 9 (P = 0.006); I² = 61% | | | | | | |
| Test for overall effect: Z = 1.22 (P = 0.22) | | | | | | |
| Test for subgroup differences: Chi² = 11.43, df = 1 (P = 0.0007), I² = 91.2% | | | | | | |

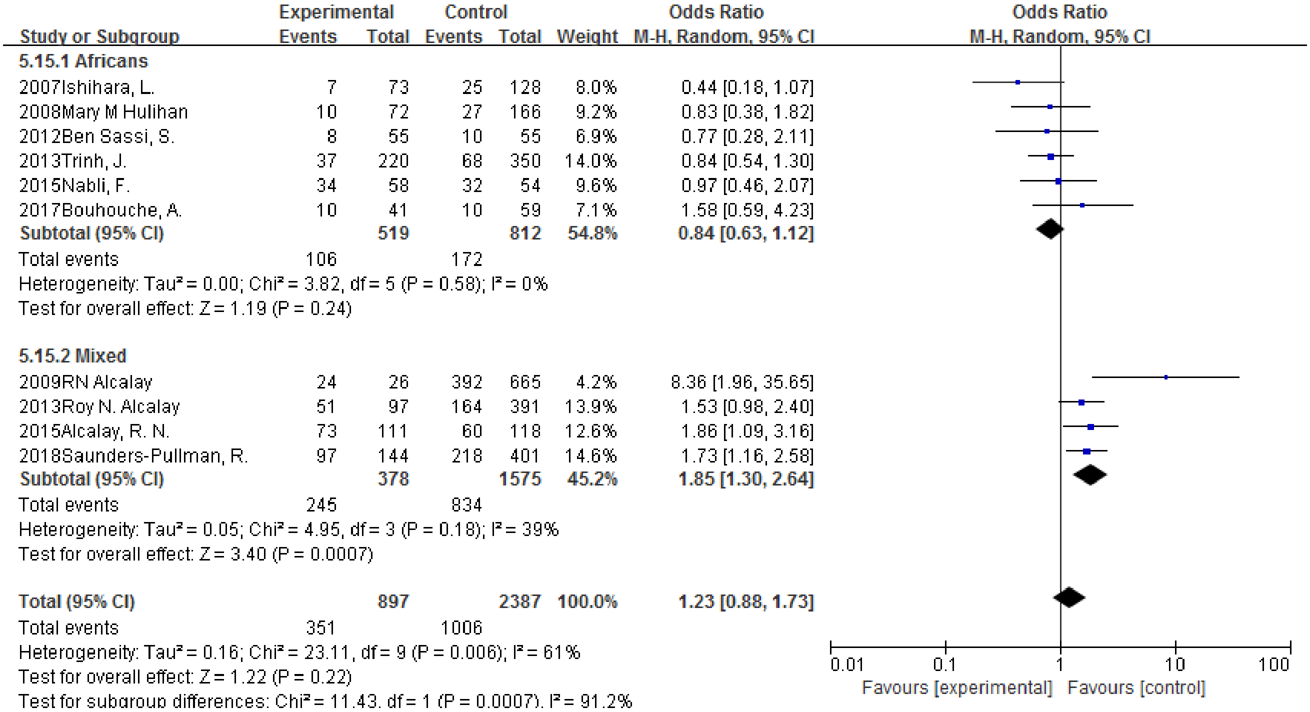


## 1.16 T-Akinetic-rigid/PIGD of G2019S by ethnicity

| Study or Subgroup | Experimental Events | Experimental Total | Control Events | Control Total | Weight | Odds Ratio M-H, Random, 95% CI |
|---|---|---|---|---|---|---|
| **5.16.1 Africans** | | | | | | |
| 2007Ishihara, L. | 59 | 73 | 89 | 128 | 13.5% | 1.85 [0.92, 3.70] |
| 2008Mary M Hulihan | 48 | 72 | 123 | 166 | 15.2% | 0.70 [0.38, 1.27] |
| 2012Ben Sassi, S. | 40 | 55 | 36 | 55 | 11.5% | 1.41 [0.62, 3.17] |
| 2013Trinh, J. | 123 | 220 | 192 | 350 | 20.6% | 1.04 [0.74, 1.47] |
| 2015Nabli, F. | 14 | 58 | 11 | 54 | 10.4% | 1.24 [0.51, 3.04] |
| 2017Bouhouche, A. | 24 | 41 | 22 | 59 | 11.5% | 2.37 [1.05, 5.36] |
| **Subtotal (95% CI)** | | **519** | | **812** | **82.7%** | **1.24 [0.89, 1.73]** |
| Total events | 308 | | 473 | | | |
| Heterogeneity: Tau² = 0.06; Chi² = 8.04, df = 5 (P = 0.15); I² = 38% | | | | | | |
| Test for overall effect: Z = 1.25 (P = 0.21) | | | | | | |
| **5.16.2 Mixed** | | | | | | |
| 2018Saunders-Pullman, R. | 23 | 144 | 105 | 401 | 17.3% | 0.54 [0.33, 0.88] |
| **Subtotal (95% CI)** | | **144** | | **401** | **17.3%** | **0.54 [0.33, 0.88]** |
| Total events | 23 | | 105 | | | |
| Heterogeneity: Not applicable | | | | | | |
| Test for overall effect: Z = 2.45 (P = 0.01) | | | | | | |
| **Total (95% CI)** | | **663** | | **1213** | **100.0%** | **1.09 [0.75, 1.60]** |
| Total events | 331 | | 578 | | | |
| Heterogeneity: Tau² = 0.15; Chi² = 15.67, df = 6 (P = 0.02); I² = 62% | | | | | | |
| Test for overall effect: Z = 0.47 (P = 0.64) | | | | | | |
| Test for subgroup differences: Chi² = 7.48, df = 1 (P = 0.006), I² = 86.6% | | | | | | |

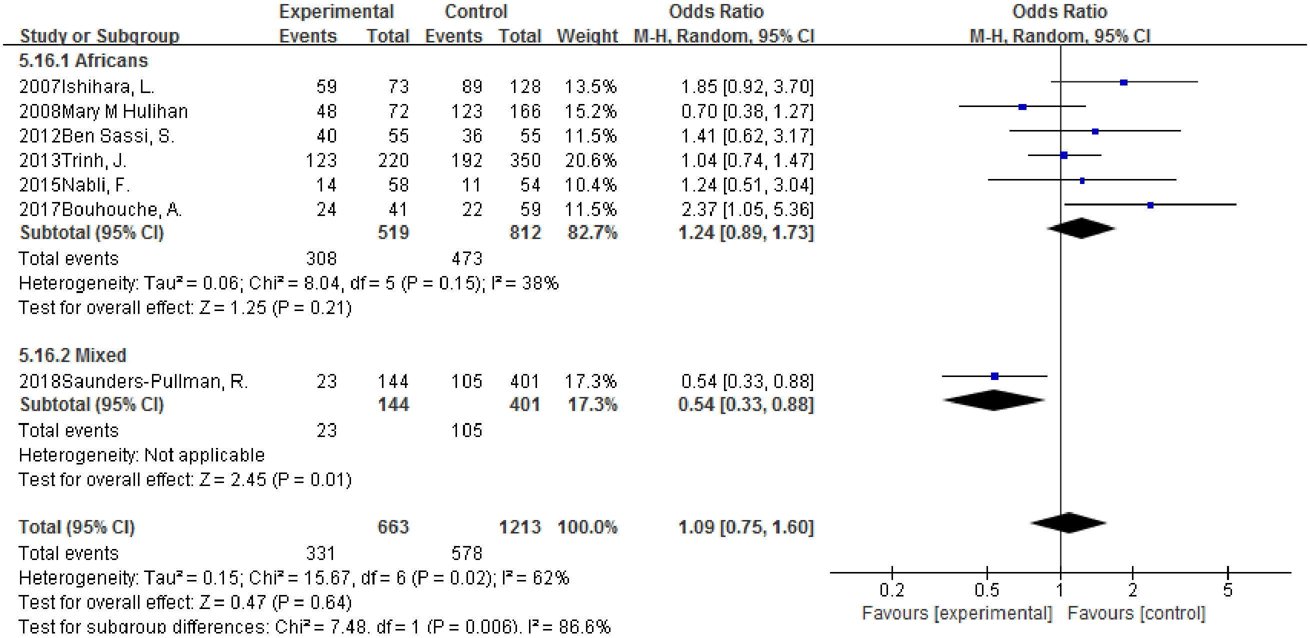


## 1.17 T-Mixed/Intermediate of G2019S by ethnicity

| Study or Subgroup | Experimental Events | Experimental Total | Control Events | Control Total | Weight | Odds Ratio M-H, Random, 95% CI |
|---|---|---|---|---|---|---|
| **5.17.1 Africans** | | | | | | |
| 2007Ishihara, L. | 7 | 73 | 14 | 128 | 11.5% | 0.86 [0.33, 2.25] |
| 2008Mary M Hulihan | 14 | 72 | 16 | 166 | 13.9% | 2.26 [1.04, 4.93] |
| 2012Ben Sassi, S. | 7 | 55 | 9 | 55 | 10.2% | 0.75 [0.26, 2.17] |
| 2013Trinh, J. | 37 | 220 | 62 | 350 | 19.1% | 0.94 [0.60, 1.47] |
| 2015Nabli, F. | 10 | 58 | 11 | 54 | 11.6% | 0.81 [0.31, 2.11] |
| 2017Bouhouche, A. | 7 | 41 | 27 | 59 | 11.5% | 0.24 [0.09, 0.64] |
| **Subtotal (95% CI)** | | **519** | | **812** | **77.8%** | **0.84 [0.49, 1.44]** |
| Total events | 82 | | 139 | | | |
| Heterogeneity: Tau² = 0.26; Chi² = 12.70, df = 5 (P = 0.03); I² = 61% | | | | | | |
| Test for overall effect: Z = 0.63 (P = 0.53) | | | | | | |
| **5.17.2 Mixed** | | | | | | |
| 2009RN Alcalay | 1 | 26 | 182 | 665 | 4.2% | 0.11 [0.01, 0.79] |
| 2018Saunders-Pullman, R. | 22 | 144 | 73 | 401 | 18.0% | 0.81 [0.48, 1.36] |
| **Subtotal (95% CI)** | | **170** | | **1066** | **22.2%** | **0.36 [0.05, 2.82]** |
| Total events | 23 | | 255 | | | |
| Heterogeneity: Tau² = 1.72; Chi² = 4.08, df = 1 (P = 0.04); I² = 75% | | | | | | |
| Test for overall effect: Z = 0.97 (P = 0.33) | | | | | | |
| **Total (95% CI)** | | **689** | | **1878** | **100.0%** | **0.77 [0.49, 1.21]** |
| Total events | 105 | | 394 | | | |
| Heterogeneity: Tau² = 0.23; Chi² = 17.26, df = 7 (P = 0.02); I² = 59% | | | | | | |
| Test for overall effect: Z = 1.13 (P = 0.26) | | | | | | |
| Test for subgroup differences: Chi² = 0.60, df = 1 (P = 0.44), I² = 0% | | | | | | |

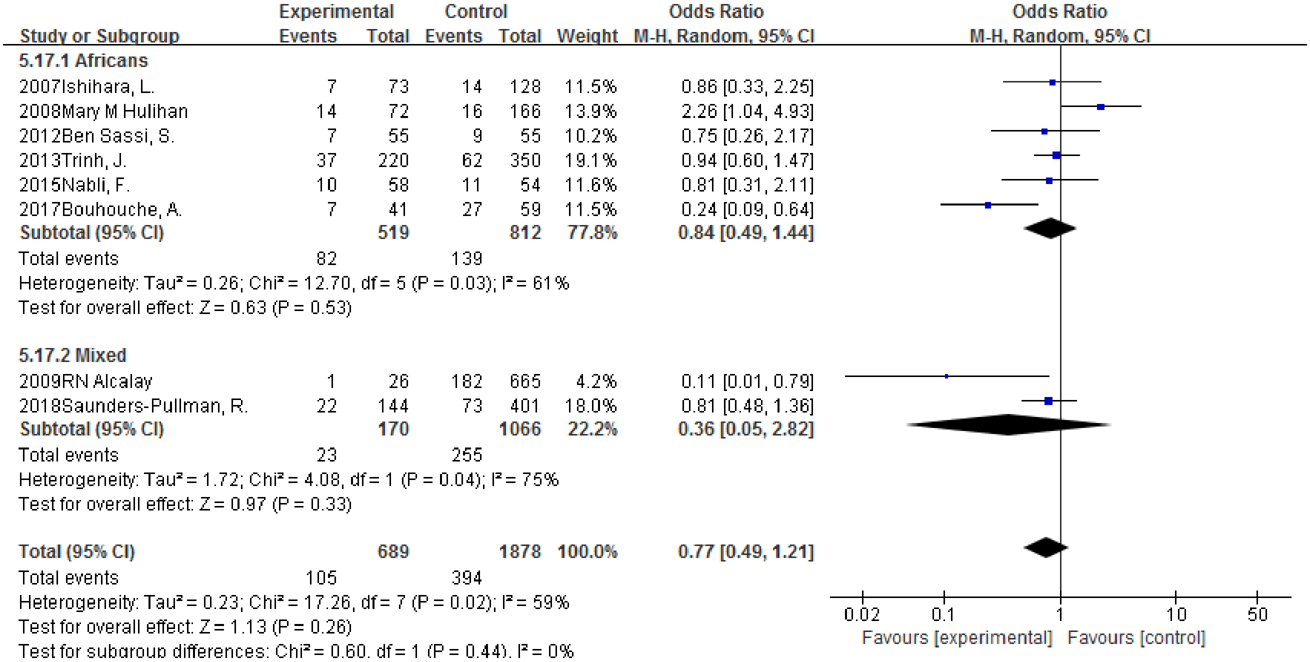


## 1.18 T-Tremor-dominant of G2019S by ethnicity

| Study or Subgroup | Experimental Mean | Experimental SD | Experimental Total | Control Mean | Control SD | Control Total | Weight | Mean Difference IV, Random, 95% CI |
|---|---|---|---|---|---|---|---|---|
| **5.18.1 Africans** | | | | | | | | |
| 2011Hashad, D. I. | 6.8 | 1.9 | 11 | 7.6 | 2.3 | 102 | 22.0% | -0.80 [-2.01, 0.41] |
| **Subtotal (95% CI)** | | | **11** | | | **102** | **22.0%** | **-0.80 [-2.01, 0.41]** |
| Heterogeneity: Not applicable | | | | | | | | |
| Test for overall effect: Z = 1.30 (P = 0.19) | | | | | | | | |
| **5.18.2 European/West Asians** | | | | | | | | |
| 2013Beatriz Tijero | 2 | 0.89 | 6 | 2.08 | 1.76 | 13 | 22.2% | -0.08 [-1.27, 1.11] |
| 2015Somme, J. H. | 1.9 | 1.9 | 12 | 4.2 | 2.8 | 27 | 17.5% | -2.30 [-3.81, -0.79] |
| **Subtotal (95% CI)** | | | **18** | | | **40** | **39.7%** | **-1.14 [-3.31, 1.03]** |
| Heterogeneity: Tau² = 1.98; Chi² = 5.13, df = 1 (P = 0.02); I² = 80% | | | | | | | | |
| Test for overall effect: Z = 1.03 (P = 0.30) | | | | | | | | |
| **5.18.3 Mixed** | | | | | | | | |
| 2018Saunders-Pullman, R. | 1.69 | 1.55 | 144 | 1.8 | 1.7 | 401 | 38.3% | -0.11 [-0.41, 0.19] |
| **Subtotal (95% CI)** | | | **144** | | | **401** | **38.3%** | **-0.11 [-0.41, 0.19]** |
| Heterogeneity: Not applicable | | | | | | | | |
| Test for overall effect: Z = 0.71 (P = 0.48) | | | | | | | | |
| **Total (95% CI)** | | | **173** | | | **543** | **100.0%** | **-0.64 [-1.48, 0.20]** |
| Heterogeneity: Tau² = 0.45; Chi² = 8.76, df = 3 (P = 0.03); I² = 66% | | | | | | | | |
| Test for overall effect: Z = 1.49 (P = 0.14) | | | | | | | | |
| Test for subgroup differences: Chi² = 1.96, df = 2 (P = 0.38), I² = 0% | | | | | | | | |

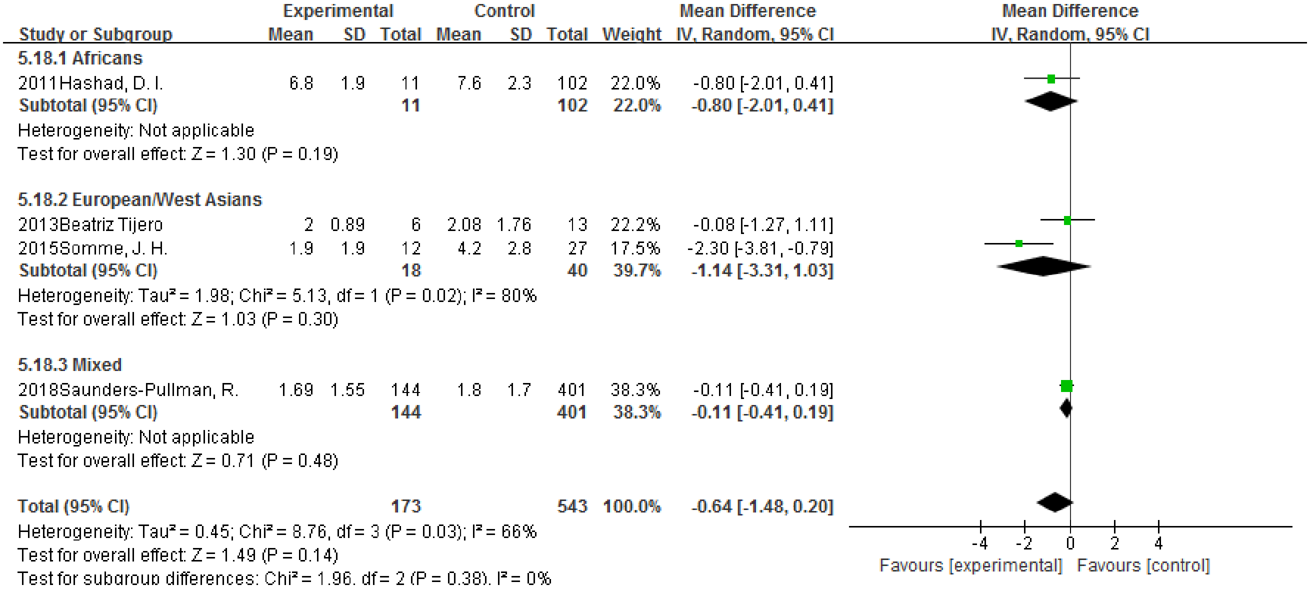


## 1.19 UPDRS Ⅰ of G2019S by ethnicity

| Study or Subgroup | Experimental Mean | Experimental SD | Experimental Total | Control Mean | Control SD | Control Total | Weight | Mean Difference IV, Random, 95% CI |
|---|---|---|---|---|---|---|---|---|
| **5.19.1 Africans** | | | | | | | | |
| 2011Hashad, D. I. | 24.3 | 6.7 | 11 | 24.2 | 5.8 | 102 | 3.0% | 0.10 [-4.02, 4.22] |
| 2013Trinh, J. | 1.41 | 0.77 | 220 | 1.31 | 0.72 | 350 | 29.3% | 0.10 [-0.03, 0.23] |
| 2015Nabli, F. | 11.17 | 6.71 | 58 | 12.02 | 5.4 | 54 | 8.0% | -0.85 [-3.10, 1.40] |
| **Subtotal (95% CI)** | | | **289** | | | **506** | **40.3%** | **0.10 [-0.03, 0.22]** |
| Heterogeneity: Tau² = 0.00; Chi² = 0.68, df = 2 (P = 0.71); I² = 0% | | | | | | | | |
| Test for overall effect: Z = 1.50 (P = 0.13) | | | | | | | | |
| **5.19.2 Europeans/West Asians** | | | | | | | | |
| 2010Vicki Shanker | 6.4 | 5.8 | 21 | 8.1 | 4.1 | 21 | 5.0% | -1.70 [-4.74, 1.34] |
| 2013Beatriz Tijero | 12.5 | 8.8 | 6 | 9.58 | 2.74 | 13 | 1.0% | 2.92 [-4.28, 10.12] |
| 2014Carles Gaig | 10.3 | 7.5 | 33 | 8.3 | 4.9 | 33 | 5.0% | 2.00 [-1.06, 5.06] |
| 2015Somme, J. H. | 12.5 | 8.4 | 12 | 13.6 | 6.6 | 27 | 1.8% | -1.10 [-6.47, 4.27] |
| **Subtotal (95% CI)** | | | **72** | | | **94** | **12.8%** | **0.20 [-2.00, 2.40]** |
| Heterogeneity: Tau² = 0.89; Chi² = 3.61, df = 3 (P = 0.31); I² = 17% | | | | | | | | |
| Test for overall effect: Z = 0.18 (P = 0.86) | | | | | | | | |
| **5.19.3 Mixed** | | | | | | | | |
| 2016Marras, C. | 10.18 | 0.75 | 449 | 11.09 | 0.19 | 684 | 29.5% | -0.91 [-0.98, -0.84] |
| 2018Saunders-Pullman, R. | 8.42 | 6.06 | 144 | 9.01 | 5.94 | 401 | 17.4% | -0.59 [-1.74, 0.56] |
| **Subtotal (95% CI)** | | | **593** | | | **1085** | **46.9%** | **-0.91 [-0.98, -0.84]** |
| Heterogeneity: Tau² = 0.00; Chi² = 0.30, df = 1 (P = 0.59); I² = 0% | | | | | | | | |
| Test for overall effect: Z = 25.20 (P < 0.00001) | | | | | | | | |
| **Total (95% CI)** | | | **954** | | | **1685** | **100.0%** | **-0.38 [-1.13, 0.36]** |
| Heterogeneity: Tau² = 0.49; Chi² = 190.62, df = 8 (P < 0.00001); I² = 96% | | | | | | | | |
| Test for overall effect: Z = 1.01 (P = 0.31) | | | | | | | | |
| Test for subgroup differences: Chi² = 185.89, df = 2 (P < 0.00001), I² = 98.9% | | | | | | | | |

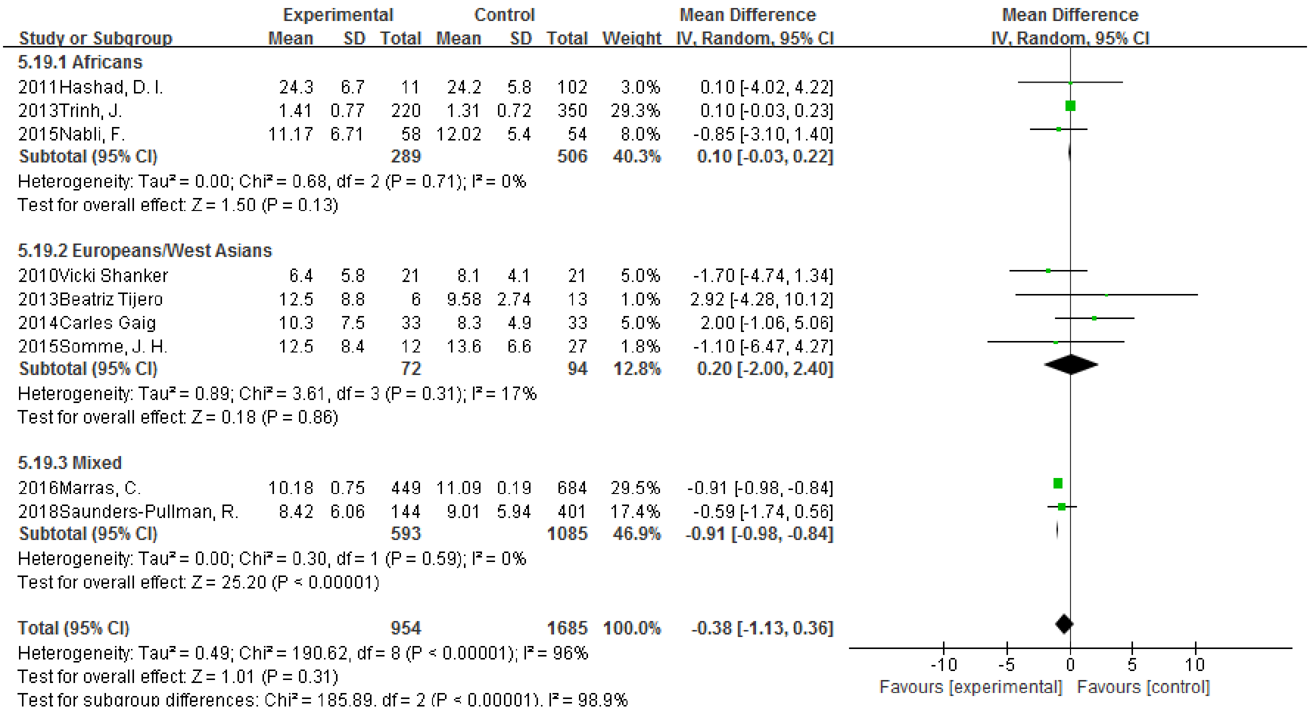


## 1.20 UPDRS II of G2019S by ethnicity

| Study or Subgroup | Experimental Mean | Experimental SD | Experimental Total | Control Mean | Control SD | Control Total | Weight | Mean Difference IV, Random, 95% CI |
|---|---|---|---|---|---|---|---|---|
| **5.21.1 Africans** | | | | | | | | |
| 2006Lesage S | 51 | 21 | 17 | 38 | 16 | 30 | 1.7% | 13.00 [1.49, 24.51] |
| 2007Ishihara, L. | 48.5 | 22.7 | 42 | 43.8 | 23.3 | 65 | 2.6% | 4.70 [-4.20, 13.60] |
| 2008S. Lesage | 47.2 | 20.3 | 11 | 30.5 | 24.1 | 14 | 0.9% | 16.70 [-0.71, 34.11] |
| 2011Hashad, D. I. | 33.1 | 4.3 | 11 | 29.1 | 5.9 | 102 | 6.7% | 4.00 [1.21, 6.79] |
| 2012Ben Sassi, S. | 42.4 | 22.9 | 55 | 42.4 | 21.6 | 55 | 2.8% | 0.00 [-8.32, 8.32] |
| 2015Nabli, F. | 44.04 | 21.71 | 58 | 37.86 | 12.54 | 54 | 3.8% | 6.18 [-0.33, 12.69] |
| **Subtotal (95% CI)** | | | **194** | | | **320** | **18.4%** | **4.79 [2.08, 7.50]** |
| Heterogeneity: Tau² = 1.28; Chi² = 5.48, df = 5 (P = 0.36); I² = 9% | | | | | | | | |
| Test for overall effect: Z = 3.46 (P = 0.0005) | | | | | | | | |
| **5.21.2 European/West Asians** | | | | | | | | |
| 2010Vicki Shanker | 9.5 | 5.6 | 21 | 11.2 | 5.8 | 21 | 6.1% | -1.70 [-5.15, 1.75] |
| 2013Beatriz Tijero | 31 | 18.07 | 6 | 26.54 | 7.48 | 13 | 1.1% | 4.46 [-10.56, 19.48] |
| 2013Maria Sierra | 28.69 | 13.23 | 29 | 28 | 12.87 | 50 | 4.1% | 0.69 [-5.30, 6.68] |
| 2013Mirelman, A. | 17.4 | 9.5 | 50 | 16.6 | 9.6 | 50 | 5.9% | 0.80 [-2.94, 4.54] |
| 2014Carles Gaig | 24 | 14 | 33 | 19.9 | 12.2 | 33 | 3.9% | 4.10 [-2.24, 10.44] |
| 2015Somme, J. H. | 29.8 | 15.9 | 12 | 31.3 | 8.1 | 27 | 2.3% | -1.50 [-11.00, 8.00] |
| **Subtotal (95% CI)** | | | **151** | | | **194** | **23.4%** | **0.17 [-1.94, 2.29]** |
| Heterogeneity: Tau² = 0.00; Chi² = 3.18, df = 5 (P = 0.67); I² = 0% | | | | | | | | |
| Test for overall effect: Z = 0.16 (P = 0.87) | | | | | | | | |
| **5.21.3 Mixed** | | | | | | | | |
| 2006L.N. Clark | 18.5 | 11.1 | 28 | 23 | 13.3 | 476 | 5.4% | -4.50 [-8.78, -0.22] |
| 2009RN Alcalay | 19.7 | 13.7 | 26 | 20.5 | 11.7 | 665 | 4.6% | -0.80 [-6.14, 4.54] |
| 2011R. Saunders-Pullman | 12.3 | 10.9 | 31 | 13.1 | 9.4 | 30 | 4.7% | -0.80 [-5.90, 4.30] |
| 2013Roy N. Alcalay | 19.8 | 12.8 | 97 | 19.9 | 11.3 | 391 | 6.7% | -0.10 [-2.88, 2.68] |
| 2014Rachel Saunders-Pullman | 22 | 12.4 | 126 | 19.2 | 11.3 | 126 | 6.6% | 2.80 [-0.13, 5.73] |
| 2015Alcalay, R. N. | 21.4 | 12.2 | 116 | 19.5 | 11.1 | 120 | 6.5% | 1.90 [-1.08, 4.88] |
| 2015Saunders-Pullman, R. | 21.1 | 13.1 | 142 | 20.1 | 11.8 | 144 | 6.6% | 1.00 [-1.89, 3.89] |
| 2016Marras, C. | 24.55 | 0.45 | 424 | 27.68 | 1.12 | 652 | 8.2% | -3.13 [-3.23, -3.03] |
| 2016Pal, G. D. | 30.8 | 11.7 | 4 | 23.4 | 12.1 | 65 | 1.7% | 7.40 [-4.44, 19.24] |
| 2018Saunders-Pullman, R. | 19.38 | 12.02 | 144 | 20.29 | 12.08 | 401 | 7.1% | -0.91 [-3.20, 1.38] |
| **Subtotal (95% CI)** | | | **1138** | | | **3070** | **58.1%** | **-0.31 [-2.24, 1.62]** |
| Heterogeneity: Tau² = 6.25; Chi² = 47.40, df = 9 (P < 0.00001); I² = 81% | | | | | | | | |
| Test for overall effect: Z = 0.31 (P = 0.76) | | | | | | | | |
| **Total (95% CI)** | | | **1483** | | | **3584** | **100.0%** | **0.99 [-0.72, 2.71]** |
| Heterogeneity: Tau² = 9.40; Chi² = 108.39, df = 21 (P < 0.00001); I² = 81% | | | | | | | | |
| Test for overall effect: Z = 1.13 (P = 0.26) | | | | | | | | |
| Test for subgroup differences: Chi² = 9.84, df = 2 (P = 0.007), I² = 79.7% | | | | | | | | |

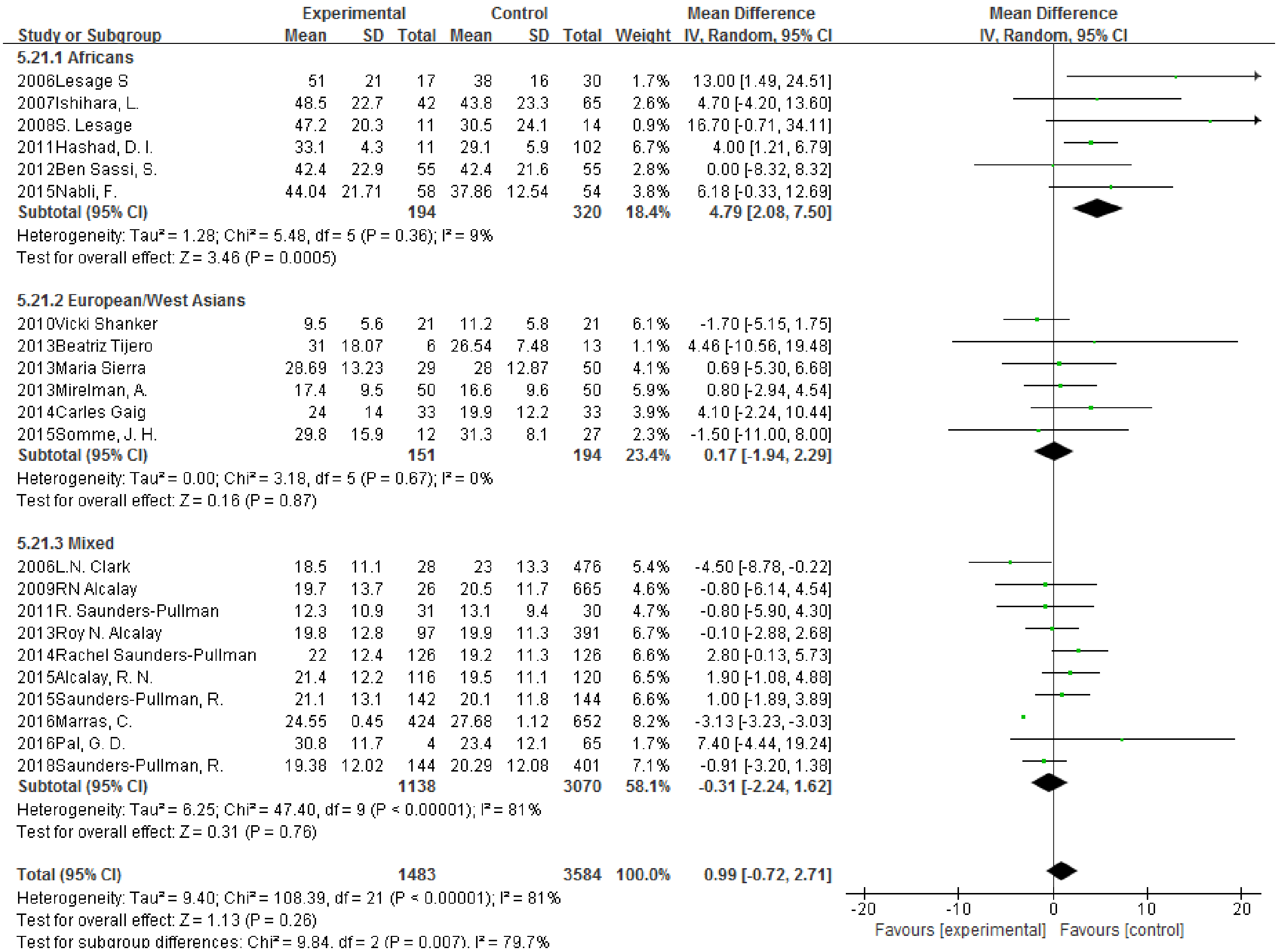


## 1.21 UPDRSIII of G2019S by ethnicity

| Study or Subgroup | Experimental Mean | Experimental SD | Experimental Total | Control Mean | Control SD | Control Total | Weight | Mean Difference IV, Random, 95% CI |
|---|---|---|---|---|---|---|---|---|
| **5.23.1 Africans** | | | | | | | | |
| 2007Ishihara, L. | 1.8 | 1 | 42 | 1.8 | 1 | 65 | 13.7% | 0.00 [-0.39, 0.39] |
| 2011Hashad, D. I. | 2.95 | 0.65 | 11 | 2.8 | 0.54 | 102 | 13.4% | 0.15 [-0.25, 0.55] |
| 2012Ben Sassi, S. | 2.3 | 0.9 | 55 | 2.7 | 1.9 | 55 | 9.8% | -0.40 [-0.96, 0.16] |
| 2015Nabli, F. | 2.3 | 0.82 | 58 | 2.96 | 1.02 | 54 | 14.9% | -0.66 [-1.00, -0.32] |
| **Subtotal (95% CI)** | | | **166** | | | **276** | **51.8%** | **-0.23 [-0.62, 0.17]** |
| Heterogeneity: Tau² = 0.12; Chi² = 11.19, df = 3 (P = 0.01); I² = 73% | | | | | | | | |
| Test for overall effect: Z = 1.11 (P = 0.27) | | | | | | | | |
| **5.23.2 European/West Asians** | | | | | | | | |
| 2014Carles Gaig | 2.1 | 1 | 33 | 1.8 | 0.8 | 33 | 12.4% | 0.30 [-0.14, 0.74] |
| **Subtotal (95% CI)** | | | **33** | | | **33** | **12.4%** | **0.30 [-0.14, 0.74]** |
| Heterogeneity: Not applicable | | | | | | | | |
| Test for overall effect: Z = 1.35 (P = 0.18) | | | | | | | | |
| **5.23.3 Mixed** | | | | | | | | |
| 2006L.N. Clark | 2.1 | 0.8 | 28 | 1.9 | 0.9 | 476 | 16.0% | 0.20 [-0.11, 0.51] |
| 2018Saunders-Pullman, R. | 2.07 | 0.88 | 144 | 2.11 | 0.84 | 401 | 19.9% | -0.04 [-0.21, 0.13] |
| **Subtotal (95% CI)** | | | **172** | | | **877** | **35.8%** | **0.04 [-0.18, 0.27]** |
| Heterogeneity: Tau² = 0.01; Chi² = 1.82, df = 1 (P = 0.18); I² = 45% | | | | | | | | |
| Test for overall effect: Z = 0.38 (P = 0.70) | | | | | | | | |
| **Total (95% CI)** | | | **371** | | | **1186** | **100.0%** | **-0.06 [-0.29, 0.18]** |
| Heterogeneity: Tau² = 0.06; Chi² = 19.64, df = 6 (P = 0.003); I² = 69% | | | | | | | | |
| Test for overall effect: Z = 0.47 (P = 0.64) | | | | | | | | |
| Test for subgroup differences: Chi² = 3.07, df = 2 (P = 0.22), I² = 34.8% | | | | | | | | |

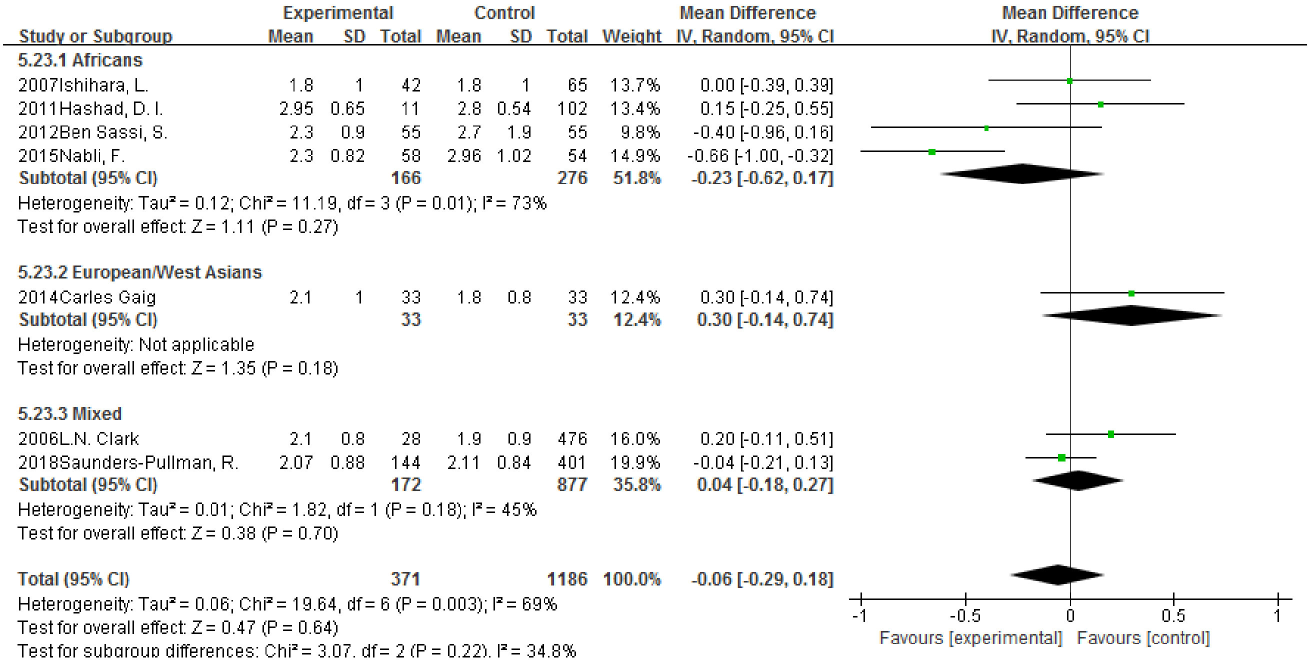

## 1.22 H-Y of G2019S by ethnicity

| Study or Subgroup | Experimental Mean | Experimental SD | Experimental Total | Control Mean | Control SD | Control Total | Weight | Mean Difference IV, Fixed, 95% CI |
|---|---|---|---|---|---|---|---|---|
| **5.24.1 Africans** | | | | | | | | |
| 2007Ishihara, L. | 66 | 15.3 | 42 | 68.2 | 16.7 | 65 | 0.0% | -2.20 [-8.36, 3.96] |
| 2012Ben Sassi, S. | 67.8 | 23.8 | 55 | 66.2 | 23.2 | 55 | 0.0% | 1.60 [-7.18, 10.38] |
| **Subtotal (95% CI)** | | | **97** | | | **120** | **0.0%** | **-0.95 [-5.99, 4.09]** |
| Heterogeneity: Chi² = 0.48, df = 1 (P = 0.49); I² = 0% | | | | | | | | |
| Test for overall effect: Z = 0.37 (P = 0.71) | | | | | | | | |
| **5.24.2 European/West Asians** | | | | | | | | |
| 2014Carles Gaig | 82.1 | 14.7 | 33 | 85.8 | 10.3 | 33 | 0.0% | -3.70 [-9.82, 2.42] |
| **Subtotal (95% CI)** | | | **33** | | | **33** | **0.0%** | **-3.70 [-9.82, 2.42]** |
| Heterogeneity: Not applicable | | | | | | | | |
| Test for overall effect: Z = 1.18 (P = 0.24) | | | | | | | | |
| **5.24.3 Mixed** | | | | | | | | |
| 2016Marras, C. | 86.2 | 0.81 | 432 | 84.71 | 0.13 | 698 | 99.9% | 1.49 [1.41, 1.57] |
| 2018Saunders-Pullman, R. | 86.65 | 18.92 | 144 | 86.26 | 20.55 | 401 | 0.0% | 0.39 [-3.30, 4.08] |
| **Subtotal (95% CI)** | | | **576** | | | **1099** | **100.0%** | **1.49 [1.41, 1.57]** |
| Heterogeneity: Chi² = 0.34, df = 1 (P = 0.56); I² = 0% | | | | | | | | |
| Test for overall effect: Z = 37.93 (P < 0.00001) | | | | | | | | |
| **Total (95% CI)** | | | **706** | | | **1252** | **100.0%** | **1.49 [1.41, 1.57]** |
| Heterogeneity: Chi² = 4.48, df = 4 (P = 0.34); I² = 11% | | | | | | | | |
| Test for overall effect: Z = 37.90 (P < 0.00001) | | | | | | | | |
| Test for subgroup differences: Chi² = 3.66, df = 2 (P = 0.16), I² = 45.3% | | | | | | | | |

Mean Difference IV, Fixed, 95% CI

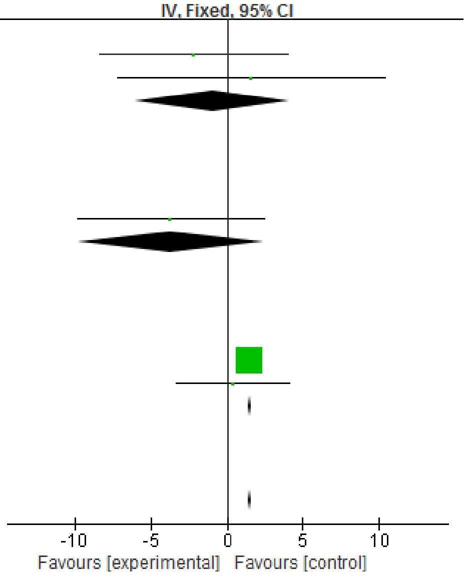


## 1.23 Schwab & England of G2019S by ethnicity

| Study or Subgroup | Experimental Events | Experimental Total | Control Events | Control Total | Weight | Odds Ratio M-H, Fixed, 95% CI |
|---|---|---|---|---|---|---|
| **5.25.1 Africans** | | | | | | |
| 2006Lesage S | 19 | 28 | 27 | 49 | 4.9% | 1.72 [0.65, 4.55] |
| 2008S. Lesage | 18 | 34 | 11 | 69 | 2.6% | 5.93 [2.34, 15.07] |
| 2015Nabli, F. | 6 | 58 | 7 | 54 | 5.0% | 0.77 [0.24, 2.47] |
| 2017Bouhouche, A. | 23 | 41 | 15 | 59 | 4.2% | 3.75 [1.60, 8.78] |
| **Subtotal (95% CI)** | | **161** | | **231** | **16.7%** | **2.61 [1.63, 4.17]** |
| Total events | 66 | | 60 | | | |
| Heterogeneity: Chi² = 8.60, df = 3 (P = 0.04); I² = 65% | | | | | | |
| Test for overall effect: Z = 4.00 (P < 0.0001) | | | | | | |
| **5.25.2 European/West Asians** | | | | | | |
| 2012Gilad Yahalom | 22 | 33 | 168 | 316 | 8.2% | 1.76 [0.83, 3.76] |
| 2013Mirelman, A. | 15 | 50 | 14 | 50 | 7.6% | 1.10 [0.46, 2.62] |
| 2014Carles Gaig | 19 | 33 | 16 | 33 | 5.2% | 1.44 [0.55, 3.81] |
| **Subtotal (95% CI)** | | **116** | | **399** | **20.9%** | **1.44 [0.89, 2.35]** |
| Total events | 56 | | 198 | | | |
| Heterogeneity: Chi² = 0.64, df = 2 (P = 0.73); I² = 0% | | | | | | |
| Test for overall effect: Z = 1.47 (P = 0.14) | | | | | | |
| **5.25.3 Hispanics** | | | | | | |
| 2017da Silva, C. P. | 12 | 17 | 50 | 83 | 3.9% | 1.58 [0.51, 4.91] |
| **Subtotal (95% CI)** | | **17** | | **83** | **3.9%** | **1.58 [0.51, 4.91]** |
| Total events | 12 | | 50 | | | |
| Heterogeneity: Not applicable | | | | | | |
| Test for overall effect: Z = 0.80 (P = 0.43) | | | | | | |
| **5.25.4 Mixed** | | | | | | |
| 2013Roy N. Alcalay | 33 | 97 | 77 | 391 | 15.6% | 2.10 [1.29, 3.43] |
| 2016Marras, C. | 86 | 242 | 73 | 419 | 26.5% | 2.61 [1.81, 3.76] |
| 2016Pal, G. D. | 3 | 4 | 61 | 72 | 1.2% | 0.54 [0.05, 5.69] |
| 2017San Luciano, M. | 41 | 530 | 26 | 759 | 15.2% | 2.36 [1.43, 3.91] |
| **Subtotal (95% CI)** | | **873** | | **1641** | **58.5%** | **2.37 [1.84, 3.05]** |
| Total events | 163 | | 237 | | | |
| Heterogeneity: Chi² = 2.02, df = 3 (P = 0.57); I² = 0% | | | | | | |
| Test for overall effect: Z = 6.72 (P < 0.00001) | | | | | | |
| **Total (95% CI)** | | **1167** | | **2354** | **100.0%** | **2.18 [1.79, 2.67]** |
| Total events | 297 | | 545 | | | |
| Heterogeneity: Chi² = 15.39, df = 11 (P = 0.17); I² = 29% | | | | | | |
| Test for overall effect: Z = 7.70 (P < 0.00001) | | | | | | |
| Test for subgroup differences: Chi² = 4.01, df = 3 (P = 0.26), I² = 25.3% | | | | | | |

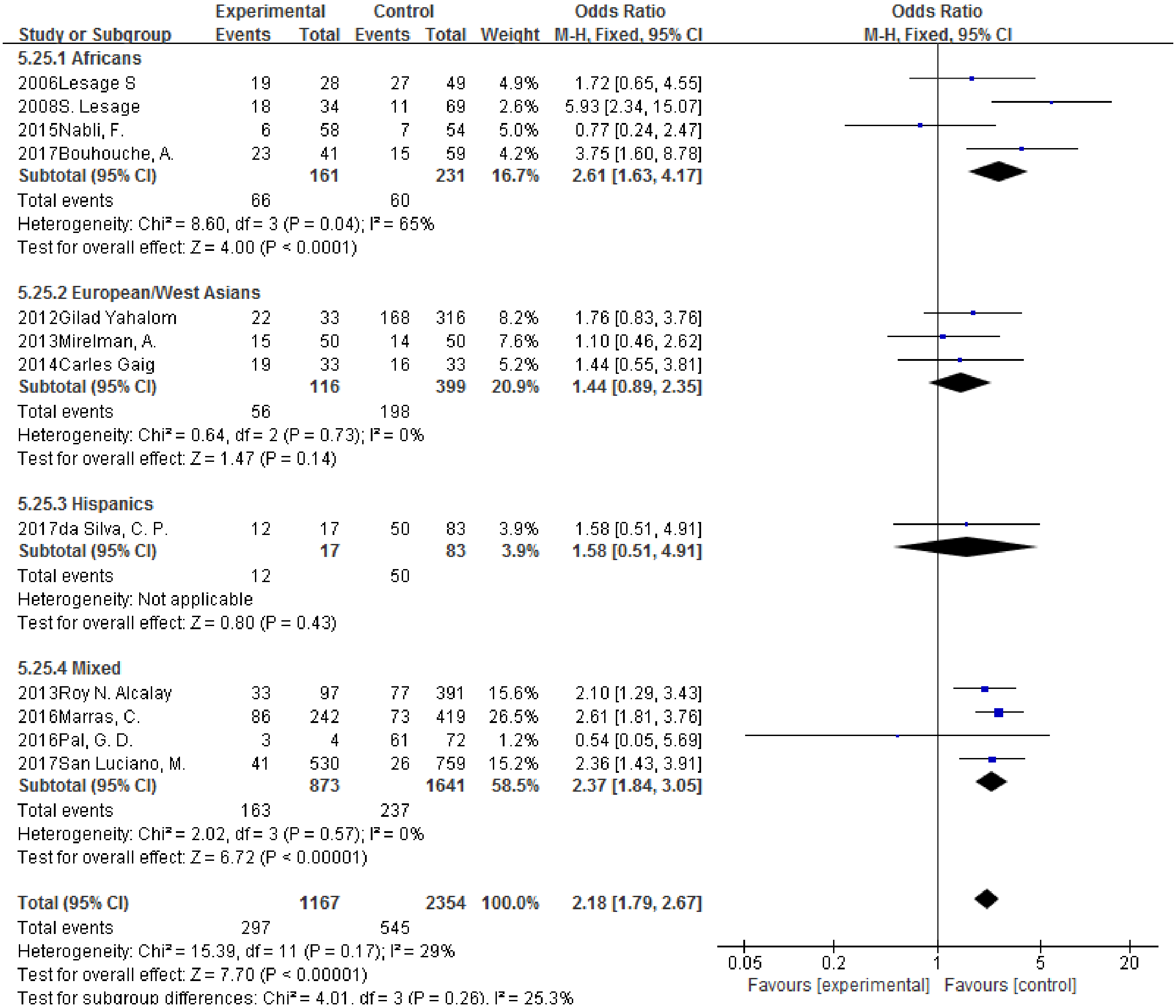


## 1.24 Dyskinesia of G2019S by ethnicity

| Study or Subgroup | Experimental Events | Experimental Total | Control Events | Control Total | Weight | Odds Ratio M-H, Random, 95% CI |
|---|---|---|---|---|---|---|
| **5.26.1 Africans** | | | | | | |
| 2008S. Lesage | 18 | 34 | 19 | 69 | 16.4% | 2.96 [1.26, 6.97] |
| 2015Nabli, F. | 25 | 58 | 23 | 54 | 17.4% | 1.02 [0.48, 2.16] |
| 2017Bouhouche, A. | 25 | 41 | 29 | 59 | 16.8% | 1.62 [0.72, 3.63] |
| **Subtotal (95% CI)** | | **133** | | **182** | **50.7%** | **1.65 [0.90, 3.01]** |
| Total events | 68 | | 71 | | | |
| Heterogeneity: Tau² = 0.11; Chi² = 3.37, df = 2 (P = 0.19); I² = 41% | | | | | | |
| Test for overall effect: Z = 1.63 (P = 0.10) | | | | | | |
| **5.26.2 European/West Asians** | | | | | | |
| 2014Carles Gaig | 18 | 33 | 17 | 33 | 15.3% | 1.13 [0.43, 2.97] |
| **Subtotal (95% CI)** | | **33** | | **33** | **15.3%** | **1.13 [0.43, 2.97]** |
| Total events | 18 | | 17 | | | |
| Heterogeneity: Not applicable | | | | | | |
| Test for overall effect: Z = 0.25 (P = 0.81) | | | | | | |
| **5.26.3 Hispanics** | | | | | | |
| 2013Emilia Mabel Gatto | 3 | 3 | 31 | 48 | 4.1% | 3.89 [0.19, 79.71] |
| 2017da Silva, C. P. | 15 | 17 | 51 | 59 | 9.6% | 1.18 [0.23, 6.14] |
| **Subtotal (95% CI)** | | **20** | | **107** | **13.7%** | **1.55 [0.36, 6.61]** |
| Total events | 18 | | 82 | | | |
| Heterogeneity: Tau² = 0.00; Chi² = 0.47, df = 1 (P = 0.49); I² = 0% | | | | | | |
| Test for overall effect: Z = 0.59 (P = 0.55) | | | | | | |
| **5.26.4 Mixed** | | | | | | |
| 2016Marras, C. | 86 | 135 | 95 | 396 | 20.4% | 5.56 [3.65, 8.46] |
| **Subtotal (95% CI)** | | **135** | | **396** | **20.4%** | **5.56 [3.65, 8.46]** |
| Total events | 86 | | 95 | | | |
| Heterogeneity: Not applicable | | | | | | |
| Test for overall effect: Z = 8.01 (P < 0.00001) | | | | | | |
| **Total (95% CI)** | | **321** | | **718** | **100.0%** | **2.02 [1.03, 3.97]** |
| Total events | 190 | | 265 | | | |
| Heterogeneity: Tau² = 0.54; Chi² = 23.52, df = 6 (P = 0.0006); I² = 74% | | | | | | |
| Test for overall effect: Z = 2.03 (P = 0.04) | | | | | | |
| Test for subgroup differences: Chi² = 16.72, df = 3 (P = 0.0008), I² = 82.1% | | | | | | |

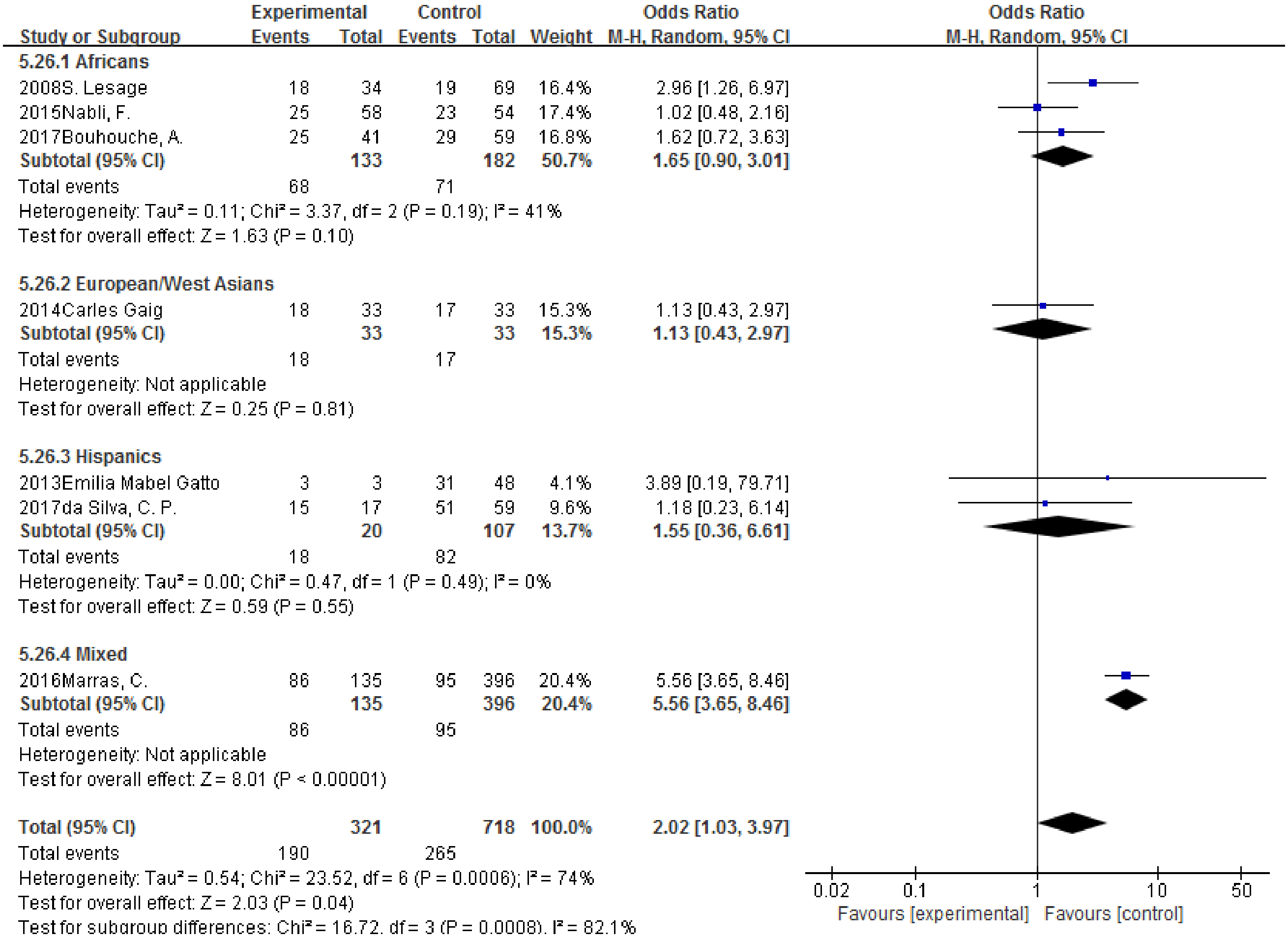


## 1.25 Motor fluctuations of G2019S by ethnicity

| Study or Subgroup | Experimental Events | Experimental Total | Control Events | Control Total | Weight | Odds Ratio M-H, Fixed, 95% CI |
|---|---|---|---|---|---|---|
| **5.39.1 Africans** | | | | | | |
| 2010Soreya Belarbi | 16 | 23 | 27 | 48 | 38.5% | 1.78 [0.62, 5.11] |
| **Subtotal (95% CI)** | | **23** | | **48** | **38.5%** | **1.78 [0.62, 5.11]** |
| Total events | 16 | | 27 | | | |
| Heterogeneity: Not applicable | | | | | | |
| Test for overall effect: Z = 1.07 (P = 0.29) | | | | | | |
| **5.39.2 European/West Asians** | | | | | | |
| 2014Carles Gaig | 8 | 33 | 9 | 33 | 49.3% | 0.85 [0.28, 2.58] |
| 2015Somme, J. H. | 1 | 12 | 3 | 27 | 12.2% | 0.73 [0.07, 7.80] |
| **Subtotal (95% CI)** | | **45** | | **60** | **61.5%** | **0.83 [0.30, 2.25]** |
| Total events | 9 | | 12 | | | |
| Heterogeneity: Chi² = 0.01, df = 1 (P = 0.90); I² = 0% | | | | | | |
| Test for overall effect: Z = 0.37 (P = 0.71) | | | | | | |
| **Total (95% CI)** | | **68** | | **108** | **100.0%** | **1.19 [0.58, 2.44]** |
| Total events | 25 | | 39 | | | |
| Heterogeneity: Chi² = 1.07, df = 2 (P = 0.59); I² = 0% | | | | | | |
| Test for overall effect: Z = 0.49 (P = 0.63) | | | | | | |
| Test for subgroup differences: Chi² = 1.06, df = 1 (P = 0.30), I² = 5.7% | | | | | | |

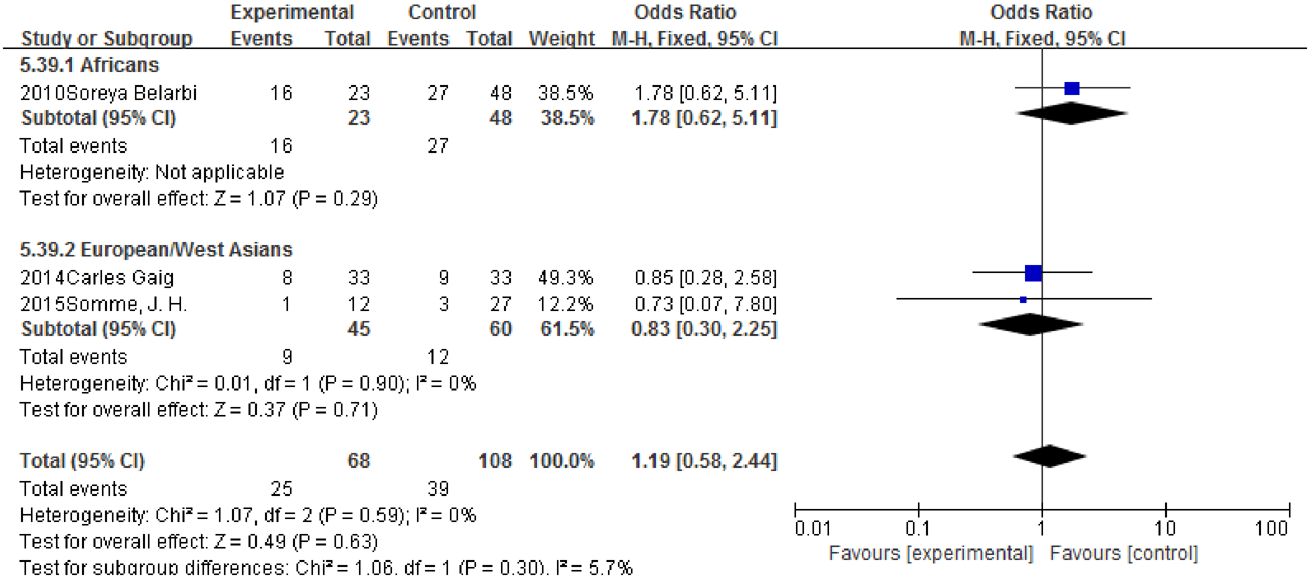


## 1.26 Anxiety of G2019S by ethnicity

| Study or Subgroup | Experimental Events | Experimental Total | Control Events | Control Total | Weight | Odds Ratio M-H, Random, 95% CI |
|---|---|---|---|---|---|---|
| **5.27.1 Africans** | | | | | | |
| 2010Soreya Belarbi | 15 | 23 | 19 | 48 | 19.3% | 2.86 [1.02, 8.06] |
| 2012Ben Sassi, S. | 18 | 55 | 25 | 55 | 23.8% | 0.58 [0.27, 1.27] |
| **Subtotal (95% CI)** | | **78** | | **103** | **43.1%** | **1.24 [0.26, 5.90]** |
| Total events | 33 | | 44 | | | |
| Heterogeneity: Tau² = 1.05; Chi² = 5.82, df = 1 (P = 0.02); I² = 83% | | | | | | |
| Test for overall effect: Z = 0.27 (P = 0.78) | | | | | | |
| **5.27.2 European/West Asians** | | | | | | |
| 2014Carles Gaig | 19 | 33 | 12 | 33 | 20.1% | 2.38 [0.88, 6.39] |
| 2015Somme, J. H. | 2 | 12 | 8 | 27 | 11.0% | 0.47 [0.08, 2.67] |
| **Subtotal (95% CI)** | | **45** | | **60** | **31.1%** | **1.25 [0.27, 5.87]** |
| Total events | 21 | | 20 | | | |
| Heterogeneity: Tau² = 0.78; Chi² = 2.52, df = 1 (P = 0.11); I² = 60% | | | | | | |
| Test for overall effect: Z = 0.28 (P = 0.78) | | | | | | |
| **5.27.3 Hispanics** | | | | | | |
| 2017da Silva, C. P. | 13 | 17 | 51 | 90 | 16.9% | 2.49 [0.75, 8.22] |
| **Subtotal (95% CI)** | | **17** | | **90** | **16.9%** | **2.49 [0.75, 8.22]** |
| Total events | 13 | | 51 | | | |
| Heterogeneity: Not applicable | | | | | | |
| Test for overall effect: Z = 1.49 (P = 0.14) | | | | | | |
| **5.27.4 Mixed** | | | | | | |
| 2008Pankratz, N. | 1 | 34 | 34 | 806 | 8.9% | 0.69 [0.09, 5.18] |
| **Subtotal (95% CI)** | | **34** | | **806** | **8.9%** | **0.69 [0.09, 5.18]** |
| Total events | 1 | | 34 | | | |
| Heterogeneity: Not applicable | | | | | | |
| Test for overall effect: Z = 0.36 (P = 0.72) | | | | | | |
| **Total (95% CI)** | | **174** | | **1059** | **100.0%** | **1.33 [0.66, 2.69]** |
| Total events | 68 | | 149 | | | |
| Heterogeneity: Tau² = 0.38; Chi² = 10.58, df = 5 (P = 0.06); I² = 53% | | | | | | |
| Test for overall effect: Z = 0.81 (P = 0.42) | | | | | | |
| Test for subgroup differences: Chi² = 1.37, df = 3 (P = 0.71), I² = 0% | | | | | | |

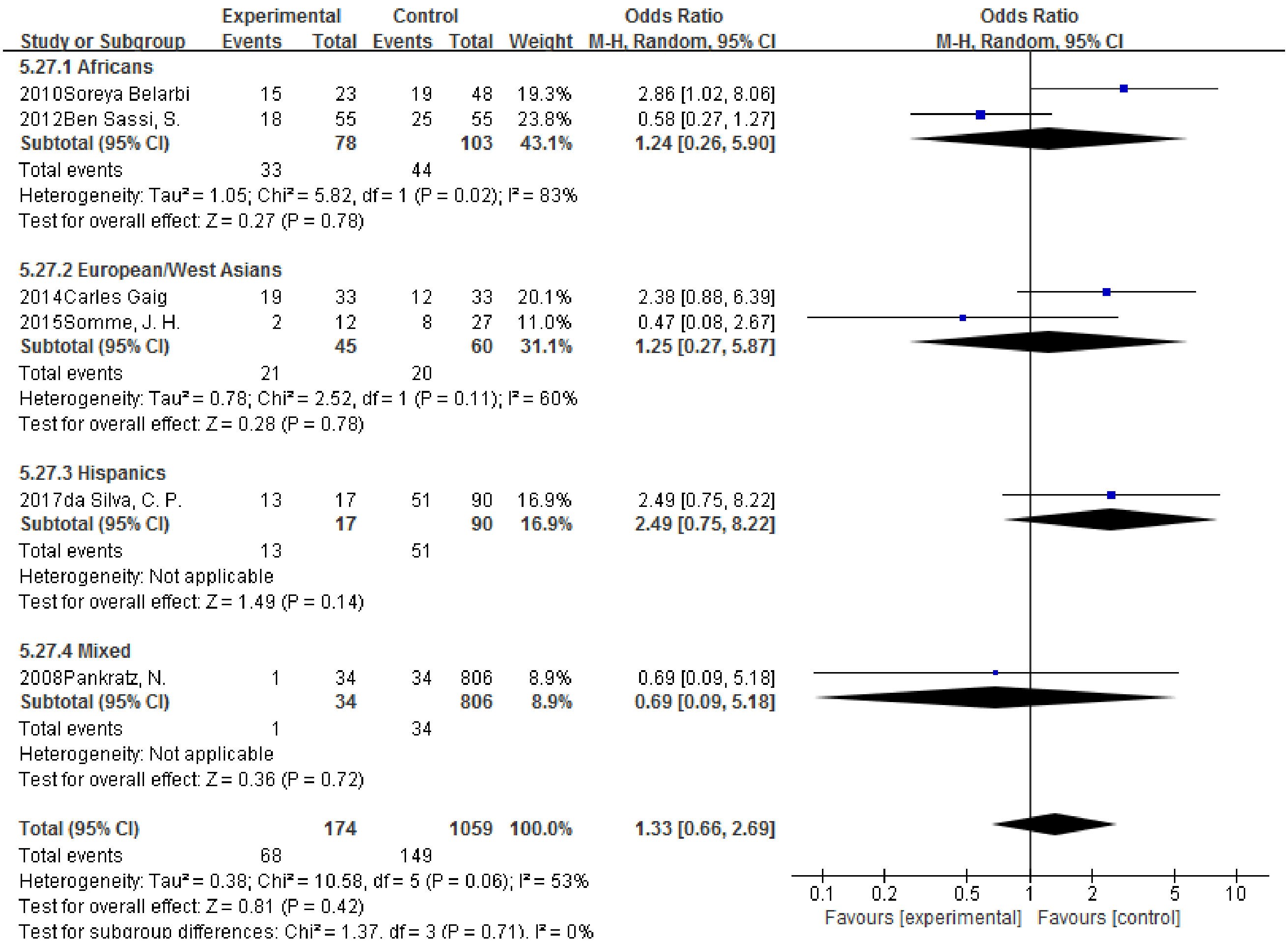


## 1.27 Depression of G2019S by ethnicity

| Study or Subgroup | Experimental Mean | Experimental SD | Experimental Total | Control Mean | Control SD | Control Total | Weight | Mean Difference IV, Fixed, 95% CI |
|---|---|---|---|---|---|---|---|---|
| **5.28.1 Africans** | | | | | | | | |
| 2012Ben Sassi, S. | 16.8 | 5.9 | 55 | 20.2 | 10.1 | 55 | 1.2% | -3.40 [-6.49, -0.31] |
| **Subtotal (95% CI)** | | | **55** | | | **55** | **1.2%** | **-3.40 [-6.49, -0.31]** |
| Heterogeneity: Not applicable | | | | | | | | |
| Test for overall effect: Z = 2.16 (P = 0.03) | | | | | | | | |
| **5.28.2 European/West Asians** | | | | | | | | |
| 2013Mirelman, A. | 4.2 | 3.5 | 50 | 3.2 | 3.2 | 50 | 6.6% | 1.00 [-0.31, 2.31] |
| **Subtotal (95% CI)** | | | **50** | | | **50** | **6.6%** | **1.00 [-0.31, 2.31]** |
| Heterogeneity: Not applicable | | | | | | | | |
| Test for overall effect: Z = 1.49 (P = 0.14) | | | | | | | | |
| **5.28.3 Mixed** | | | | | | | | |
| 2013Roy N. Alcalay | 4.2 | 3.2 | 97 | 3.7 | 3.4 | 391 | 22.0% | 0.50 [-0.22, 1.22] |
| 2015Alcalay, R. N. | 3.7 | 3.3 | 116 | 3.2 | 3.2 | 120 | 16.6% | 0.50 [-0.33, 1.33] |
| 2017San Luciano, M. | 5.4 | 4.2 | 530 | 5 | 4.1 | 759 | 53.6% | 0.40 [-0.06, 0.86] |
| **Subtotal (95% CI)** | | | **743** | | | **1270** | **92.2%** | **0.44 [0.09, 0.79]** |
| Heterogeneity: Chi² = 0.08, df = 2 (P = 0.96); I² = 0% | | | | | | | | |
| Test for overall effect: Z = 2.46 (P = 0.01) | | | | | | | | |
| **Total (95% CI)** | | | **848** | | | **1375** | **100.0%** | **0.43 [0.09, 0.77]** |
| Heterogeneity: Chi² = 6.70, df = 4 (P = 0.15); I² = 40% | | | | | | | | |
| Test for overall effect: Z = 2.51 (P = 0.01) | | | | | | | | |
| Test for subgroup differences: Chi² = 6.62, df = 2 (P = 0.04), I² = 69.8% | | | | | | | | |

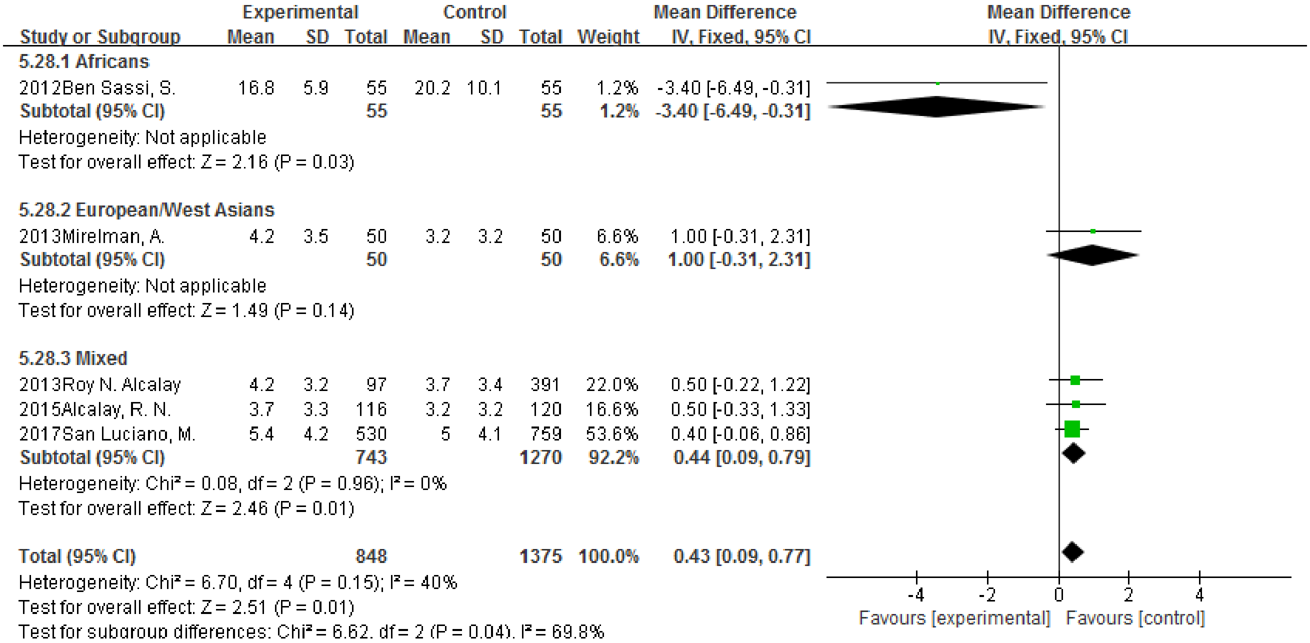

## 1.28 GDS15 of G2019S by ethnicity

| Study or Subgroup | Experimental Events | Experimental Total | Control Events | Control Total | Weight | Odds Ratio M-H, Fixed, 95% CI |
|---|---|---|---|---|---|---|
| **5.29.1 Africans** | | | | | | |
| 2010Soreya Belarbi | 6 | 23 | 3 | 48 | 4.3% | 5.29 [1.19, 23.58] |
| 2017Bouhouche, A. | 25 | 41 | 28 | 59 | 26.9% | 1.73 [0.77, 3.89] |
| **Subtotal (95% CI)** | | **64** | | **107** | **31.2%** | **2.22 [1.10, 4.50]** |
| Total events | 31 | | 31 | | | |
| Heterogeneity: Chi² = 1.66, df = 1 (P = 0.20); I² = 40% | | | | | | |
| Test for overall effect: Z = 2.22 (P = 0.03) | | | | | | |
| **5.29.2 European/West Asians** | | | | | | |
| 2014Carles Gaig | 3 | 33 | 5 | 33 | 13.7% | 0.56 [0.12, 2.56] |
| 2015Somme, J. H. | 0 | 12 | 7 | 27 | 13.7% | 0.11 [0.01, 2.08] |
| **Subtotal (95% CI)** | | **45** | | **60** | **27.4%** | **0.33 [0.09, 1.21]** |
| Total events | 3 | | 12 | | | |
| Heterogeneity: Chi² = 0.99, df = 1 (P = 0.32); I² = 0% | | | | | | |
| Test for overall effect: Z = 1.67 (P = 0.09) | | | | | | |
| **5.29.3 Hispanics** | | | | | | |
| 2013Emilia Mabel Gatto | 3 | 3 | 12 | 48 | 0.7% | 20.44 [0.99, 423.90] |
| 2017da Silva, C. P. | 3 | 17 | 39 | 90 | 30.7% | 0.28 [0.08, 1.04] |
| **Subtotal (95% CI)** | | **20** | | **138** | **31.4%** | **0.74 [0.29, 1.89]** |
| Total events | 6 | | 51 | | | |
| Heterogeneity: Chi² = 6.69, df = 1 (P = 0.010); I² = 85% | | | | | | |
| Test for overall effect: Z = 0.64 (P = 0.52) | | | | | | |
| **5.29.4 Mixed** | | | | | | |
| 2009RN Alcalay | 1 | 26 | 46 | 665 | 10.0% | 0.54 [0.07, 4.06] |
| **Subtotal (95% CI)** | | **26** | | **665** | **10.0%** | **0.54 [0.07, 4.06]** |
| Total events | 1 | | 46 | | | |
| Heterogeneity: Not applicable | | | | | | |
| Test for overall effect: Z = 0.60 (P = 0.55) | | | | | | |
| **Total (95% CI)** | | **155** | | **970** | **100.0%** | **1.07 [0.67, 1.71]** |
| Total events | 41 | | 140 | | | |
| Heterogeneity: Chi² = 16.82, df = 6 (P = 0.010); I² = 64% | | | | | | |
| Test for overall effect: Z = 0.28 (P = 0.78) | | | | | | |
| Test for subgroup differences: Chi² = 8.30, df = 3 (P = 0.04), I² = 63.9% | | | | | | |

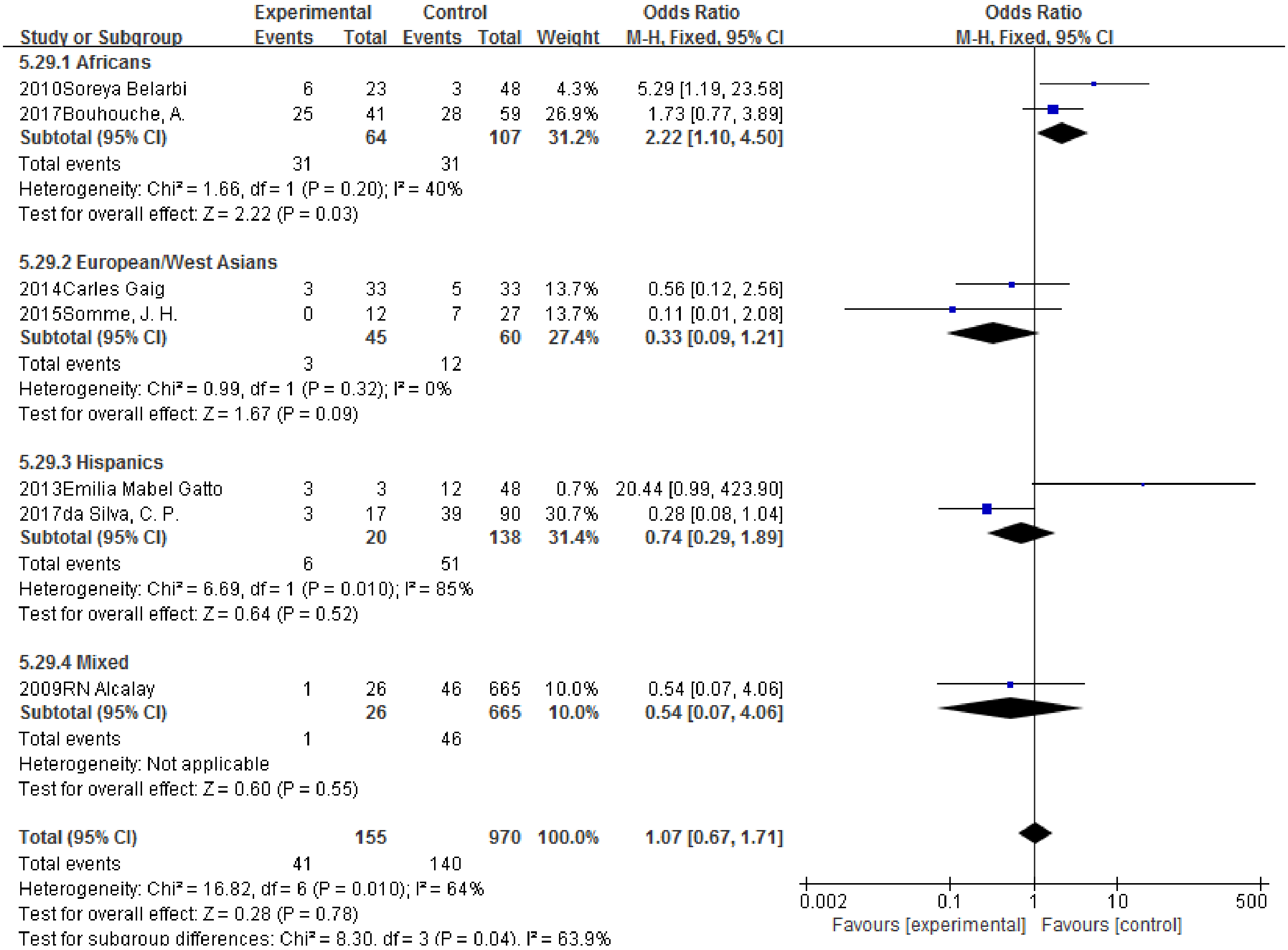


## 1.29 Hallucination of G2019S by ethnicity

| Study or Subgroup | Experimental Mean | Experimental SD | Experimental Total | Control Mean | Control SD | Control Total | Weight | Mean Difference IV, Fixed, 95% CI |
|---|---|---|---|---|---|---|---|---|
| **5.40.1 European/West Asians** | | | | | | | | |
| 2013Beatriz Tijero | 8.33 | 10.74 | 6 | 14.66 | 7.23 | 13 | 1.2% | -6.33 [-15.78, 3.12] |
| 2014Carles Gaig | 16.4 | 8.7 | 33 | 13.5 | 8.5 | 33 | 6.4% | 2.90 [-1.25, 7.05] |
| **Subtotal (95% CI)** | | | **39** | | | **46** | **7.7%** | **1.41 [-2.39, 5.21]** |
| Heterogeneity: Chi² = 3.07, df = 1 (P = 0.08); I² = 67% | | | | | | | | |
| Test for overall effect: Z = 0.73 (P = 0.47) | | | | | | | | |
| **5.40.2 Mixed** | | | | | | | | |
| 2017San Luciano, M. | 14.5 | 10.1 | 530 | 14.3 | 9.5 | 759 | 92.3% | 0.20 [-0.89, 1.29] |
| **Subtotal (95% CI)** | | | **530** | | | **759** | **92.3%** | **0.20 [-0.89, 1.29]** |
| Heterogeneity: Not applicable | | | | | | | | |
| Test for overall effect: Z = 0.36 (P = 0.72) | | | | | | | | |
| **Total (95% CI)** | | | **569** | | | **805** | **100.0%** | **0.29 [-0.76, 1.34]** |
| Heterogeneity: Chi² = 3.43, df = 2 (P = 0.18); I² = 42% | | | | | | | | |
| Test for overall effect: Z = 0.55 (P = 0.59) | | | | | | | | |
| Test for subgroup differences: Chi² = 0.36, df = 1 (P = 0.55), I² = 0% | | | | | | | | |

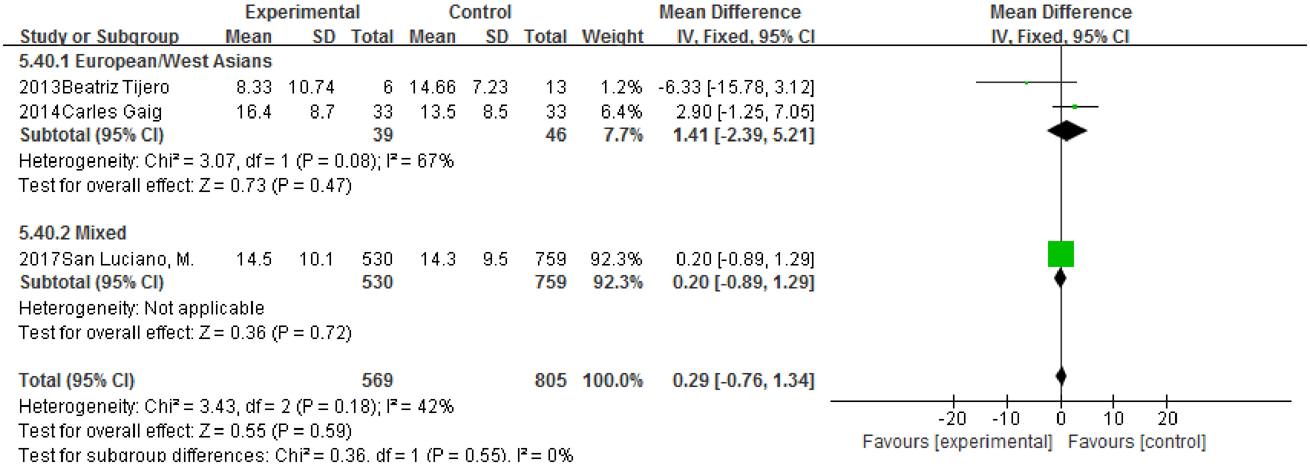


## 1.30 SCOPA-AUT of G2019S by ethnicity

| Study or Subgroup | Experimental Events | Experimental Total | Control Events | Control Total | Weight | Odds Ratio M-H, Fixed, 95% CI |
|---|---|---|---|---|---|---|
| **5.30.1 African** | | | | | | |
| 2010Soreya Belarbi | 10 | 23 | 10 | 48 | 10.0% | 2.92 [0.99, 8.60] |
| 2012Ben Sassi, S. | 16 | 55 | 18 | 55 | 35.0% | 0.84 [0.38, 1.90] |
| 2017Bouhouche, A. | 26 | 41 | 26 | 59 | 21.4% | 2.20 [0.97, 4.98] |
| **Subtotal (95% CI)** | | **119** | | **162** | **66.5%** | **1.59 [0.97, 2.63]** |
| Total events | 52 | | 54 | | | |
| Heterogeneity: Chi² = 4.18, df = 2 (P = 0.12); I² = 52% | | | | | | |
| Test for overall effect: Z = 1.83 (P = 0.07) | | | | | | |
| **5.30.2 European/West Asians** | | | | | | |
| 2005Jose Miguel Bras | 2 | 11 | 13 | 117 | 5.0% | 1.78 [0.35, 9.14] |
| 2016Dagan, E. | 4 | 20 | 47 | 181 | 20.5% | 0.71 [0.23, 2.24] |
| **Subtotal (95% CI)** | | **31** | | **298** | **25.5%** | **0.92 [0.36, 2.35]** |
| Total events | 6 | | 60 | | | |
| Heterogeneity: Chi² = 0.81, df = 1 (P = 0.37); I² = 0% | | | | | | |
| Test for overall effect: Z = 0.17 (P = 0.86) | | | | | | |
| **5.30.3 Hispanics** | | | | | | |
| 2017da Silva, C. P. | 5 | 16 | 13 | 82 | 8.0% | 2.41 [0.72, 8.11] |
| **Subtotal (95% CI)** | | **16** | | **82** | **8.0%** | **2.41 [0.72, 8.11]** |
| Total events | 5 | | 13 | | | |
| Heterogeneity: Not applicable | | | | | | |
| Test for overall effect: Z = 1.42 (P = 0.15) | | | | | | |
| **Total (95% CI)** | | **166** | | **542** | **100.0%** | **1.49 [0.99, 2.24]** |
| Total events | 63 | | 127 | | | |
| Heterogeneity: Chi² = 6.52, df = 5 (P = 0.26); I² = 23% | | | | | | |
| Test for overall effect: Z = 1.90 (P = 0.06) | | | | | | |
| Test for subgroup differences: Chi² = 1.69, df = 2 (P = 0.43), I² = 0% | | | | | | |

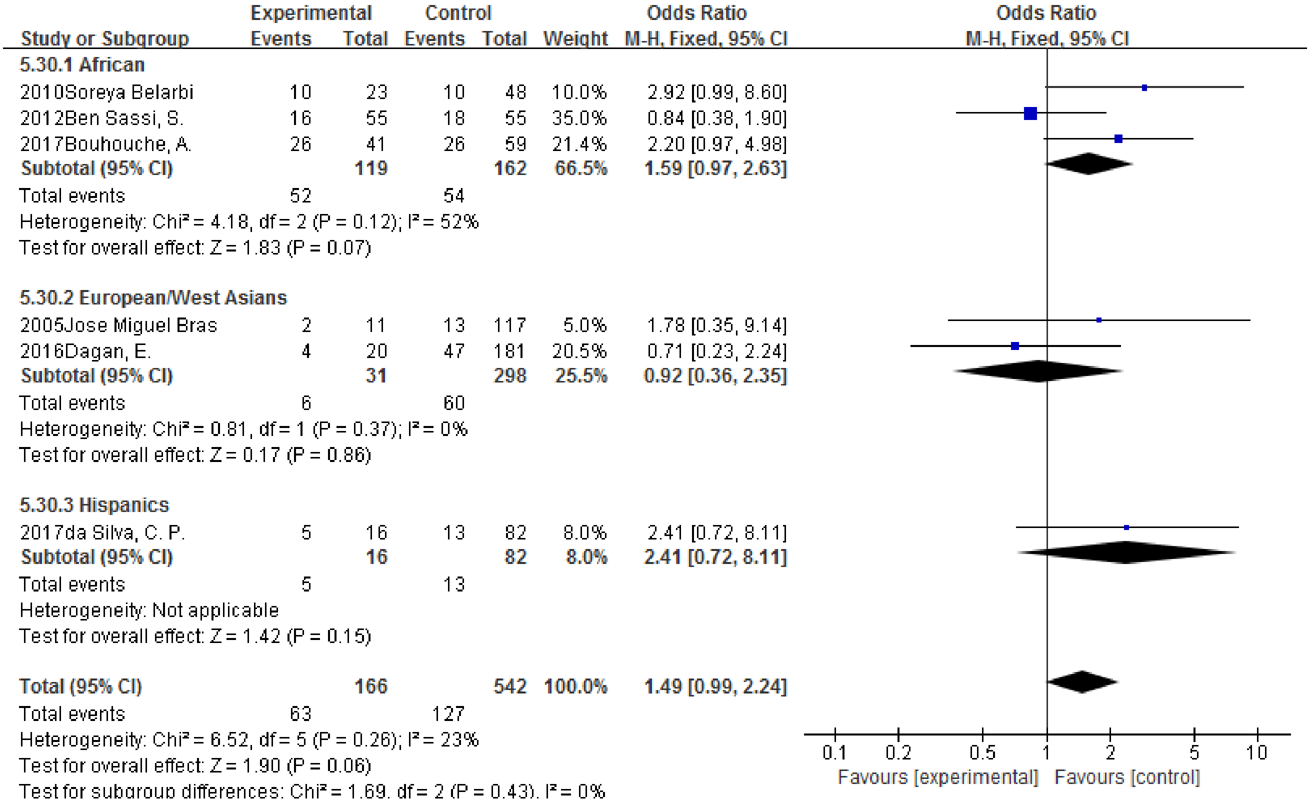


## 1.31 Cognitive impairments of G2019S by ethnicity

| Study or Subgroup | Experimental Mean | Experimental SD | Experimental Total | Control Mean | Control SD | Control Total | Weight | Mean Difference IV, Fixed, 95% CI |
|---|---|---|---|---|---|---|---|---|
| **5.31.1 Africans** | | | | | | | | |
| 2006Lesage S | 26.3 | 4.6 | 14 | 28.4 | 2.6 | 45 | 3.0% | -2.10 [-4.63, 0.43] |
| 2008S. Lesage | 28.3 | 3.7 | 26 | 29.2 | 2.2 | 64 | 8.3% | -0.90 [-2.42, 0.62] |
| 2012Ben Sassi, S. | 25.5 | 3.2 | 55 | 25.16 | 2.85 | 55 | 15.0% | 0.34 [-0.79, 1.47] |
| **Subtotal (95% CI)** | | | **95** | | | **164** | **26.4%** | **-0.33 [-1.19, 0.52]** |
| Heterogeneity: Chi² = 3.77, df = 2 (P = 0.15); I² = 47% | | | | | | | | |
| Test for overall effect: Z = 0.76 (P = 0.45) | | | | | | | | |
| **5.31.2 European/West Asians** | | | | | | | | |
| 2010Vicki Shanker | 29.19 | 1.4 | 21 | 28.67 | 1.91 | 21 | 18.8% | 0.52 [-0.49, 1.53] |
| **Subtotal (95% CI)** | | | **21** | | | **21** | **18.8%** | **0.52 [-0.49, 1.53]** |
| Heterogeneity: Not applicable | | | | | | | | |
| Test for overall effect: Z = 1.01 (P = 0.31) | | | | | | | | |
| **5.31.3 Mixed** | | | | | | | | |
| 2009RN Alcalay | 29.1 | 1.6 | 26 | 29.1 | 1.6 | 665 | 49.0% | 0.00 [-0.63, 0.63] |
| 2016Pal, G. D. | 28.2 | 1.8 | 4 | 28.6 | 2 | 72 | 5.8% | -0.40 [-2.22, 1.42] |
| **Subtotal (95% CI)** | | | **30** | | | **737** | **54.8%** | **-0.04 [-0.64, 0.55]** |
| Heterogeneity: Chi² = 0.17, df = 1 (P = 0.68); I² = 0% | | | | | | | | |
| Test for overall effect: Z = 0.14 (P = 0.89) | | | | | | | | |
| **Total (95% CI)** | | | **146** | | | **922** | **100.0%** | **-0.01 [-0.45, 0.43]** |
| Heterogeneity: Chi² = 5.54, df = 5 (P = 0.35); I² = 10% | | | | | | | | |
| Test for overall effect: Z = 0.06 (P = 0.95) | | | | | | | | |
| Test for subgroup differences: Chi² = 1.60, df = 2 (P = 0.45), I² = 0% | | | | | | | | |

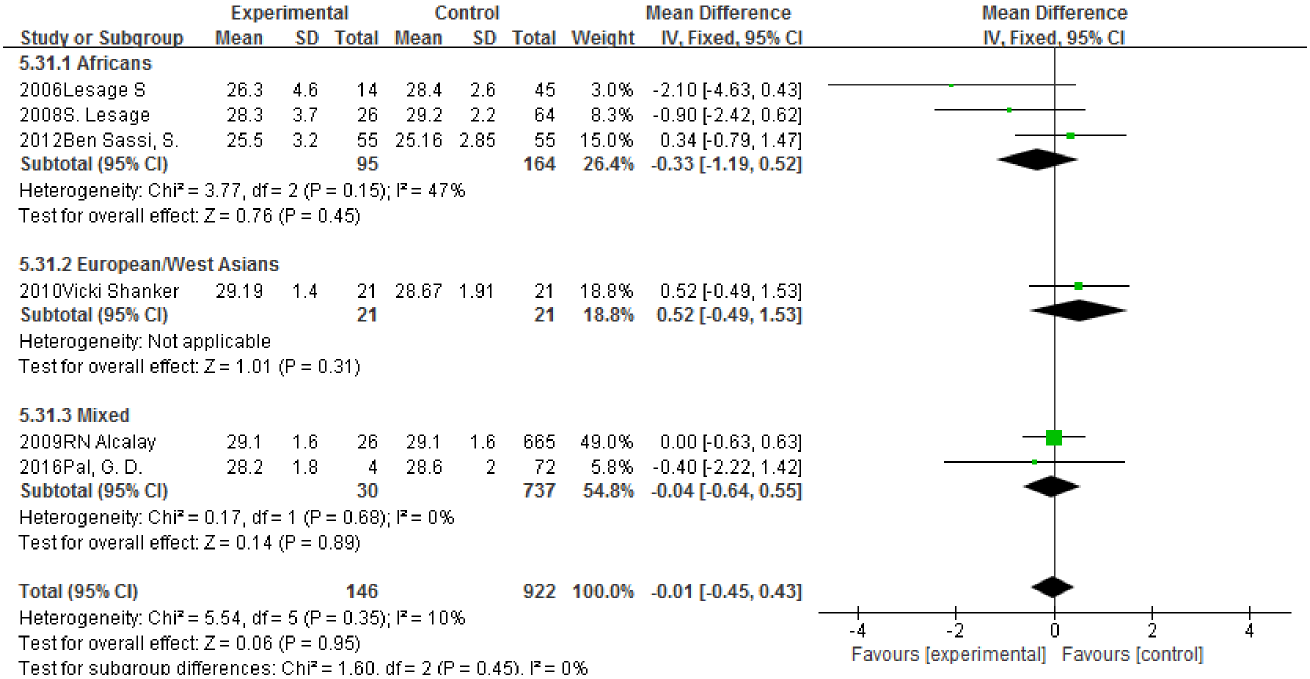


## 1.32 MMSE of G2019S by ethnicity

| Study or Subgroup | Experimental Mean | Experimental SD | Experimental Total | Control Mean | Control SD | Control Total | Weight | Mean Difference IV, Fixed, 95% CI |
|---|---|---|---|---|---|---|---|---|
| **5.32.1 Africans** | | | | | | | | |
| 2012Ben Sassi, S. | 21.8 | 4.3 | 20 | 21.8 | 3.7 | 17 | 1.2% | 0.00 [-2.58, 2.58] |
| **Subtotal (95% CI)** | | | **20** | | | **17** | **1.2%** | **0.00 [-2.58, 2.58]** |
| Heterogeneity: Not applicable | | | | | | | | |
| Test for overall effect: Z = 0.00 (P = 1.00) | | | | | | | | |
| **5.32.2 Mixed** | | | | | | | | |
| 2013Roy N. Alcalay | 25.2 | 2.9 | 97 | 24.7 | 3.9 | 391 | 16.8% | 0.50 [-0.19, 1.19] |
| 2014Rachel Saunders-Pullman | 25.4 | 3 | 126 | 25.2 | 3.8 | 126 | 11.4% | 0.20 [-0.65, 1.05] |
| 2015Alcalay, R. N. | 25.4 | 2.7 | 116 | 25 | 3.4 | 120 | 13.3% | 0.40 [-0.38, 1.18] |
| 2017San Luciano, M. | 24.1 | 4.3 | 530 | 24.5 | 4.4 | 759 | 35.0% | -0.40 [-0.88, 0.08] |
| 2018Saunders-Pullman, R. | 25.14 | 2.85 | 144 | 24.71 | 3.9 | 401 | 22.4% | 0.43 [-0.17, 1.03] |
| **Subtotal (95% CI)** | | | **1013** | | | **1797** | **98.8%** | **0.12 [-0.17, 0.40]** |
| Heterogeneity: Chi² = 7.17, df = 4 (P = 0.13); I² = 44% | | | | | | | | |
| Test for overall effect: Z = 0.80 (P = 0.42) | | | | | | | | |
| **Total (95% CI)** | | | **1033** | | | **1814** | **100.0%** | **0.12 [-0.17, 0.40]** |
| Heterogeneity: Chi² = 7.18, df = 5 (P = 0.21); I² = 30% | | | | | | | | |
| Test for overall effect: Z = 0.80 (P = 0.42) | | | | | | | | |
| Test for subgroup differences: Chi² = 0.01, df = 1 (P = 0.93), I² = 0% | | | | | | | | |

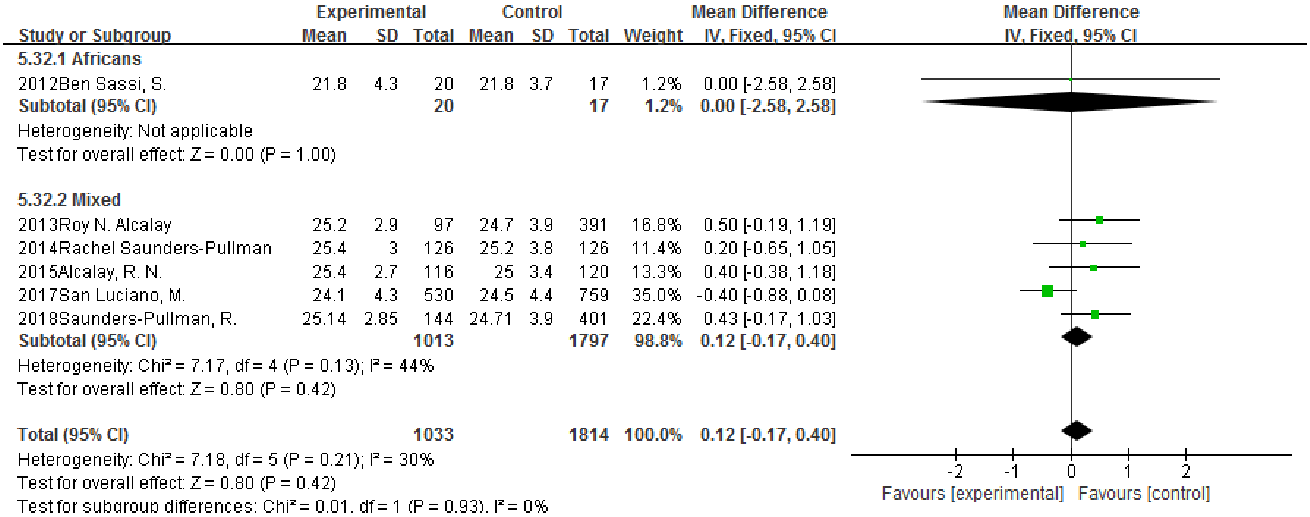


## 1.33 MoCA of G2019S by ethnicity

| Study or Subgroup | Experimental Events | Experimental Total | Control Events | Control Total | Weight | Odds Ratio M-H, Random, 95% CI |
|---|---|---|---|---|---|---|
| **5.33.1 Africans** | | | | | | |
| 2010Soreya Belarbi | 15 | 23 | 19 | 48 | 16.5% | 2.86 [1.02, 8.06] |
| 2017Bouhouche, A. | 22 | 41 | 19 | 59 | 18.8% | 2.44 [1.07, 5.54] |
| **Subtotal (95% CI)** | | **64** | | **107** | **35.3%** | **2.59 [1.36, 4.94]** |
| Total events | 37 | | 38 | | | |
| Heterogeneity: Tau² = 0.00; Chi² = 0.06, df = 1 (P = 0.81); I² = 0% | | | | | | |
| Test for overall effect: Z = 2.90 (P = 0.004) | | | | | | |
| **5.33.2 European/West Asians** | | | | | | |
| 2014Carles Gaig | 22 | 33 | 19 | 33 | 16.9% | 1.47 [0.54, 4.01] |
| 2015Somme, J. H. | 6 | 12 | 11 | 27 | 13.2% | 1.45 [0.37, 5.71] |
| **Subtotal (95% CI)** | | **45** | | **60** | **30.0%** | **1.47 [0.65, 3.29]** |
| Total events | 28 | | 30 | | | |
| Heterogeneity: Tau² = 0.00; Chi² = 0.00, df = 1 (P = 0.99); I² = 0% | | | | | | |
| Test for overall effect: Z = 0.93 (P = 0.35) | | | | | | |
| **5.33.3 Hispanics** | | | | | | |
| 2017da Silva, C. P. | 13 | 17 | 76 | 82 | 12.9% | 0.26 [0.06, 1.04] |
| **Subtotal (95% CI)** | | **17** | | **82** | **12.9%** | **0.26 [0.06, 1.04]** |
| Total events | 13 | | 76 | | | |
| Heterogeneity: Not applicable | | | | | | |
| Test for overall effect: Z = 1.91 (P = 0.06) | | | | | | |
| **5.33.4 Mixed** | | | | | | |
| 2015Saunders-Pullman, R. | 28 | 142 | 44 | 144 | 21.7% | 0.56 [0.32, 0.96] |
| **Subtotal (95% CI)** | | **142** | | **144** | **21.7%** | **0.56 [0.32, 0.96]** |
| Total events | 28 | | 44 | | | |
| Heterogeneity: Not applicable | | | | | | |
| Test for overall effect: Z = 2.10 (P = 0.04) | | | | | | |
| **Total (95% CI)** | | **268** | | **393** | **100.0%** | **1.17 [0.56, 2.41]** |
| Total events | 106 | | 188 | | | |
| Heterogeneity: Tau² = 0.56; Chi² = 17.39, df = 5 (P = 0.004); I² = 71% | | | | | | |
| Test for overall effect: Z = 0.41 (P = 0.68) | | | | | | |
| Test for subgroup differences: Chi² = 17.32, df = 3 (P = 0.0006), I² = 82.7% | | | | | | |

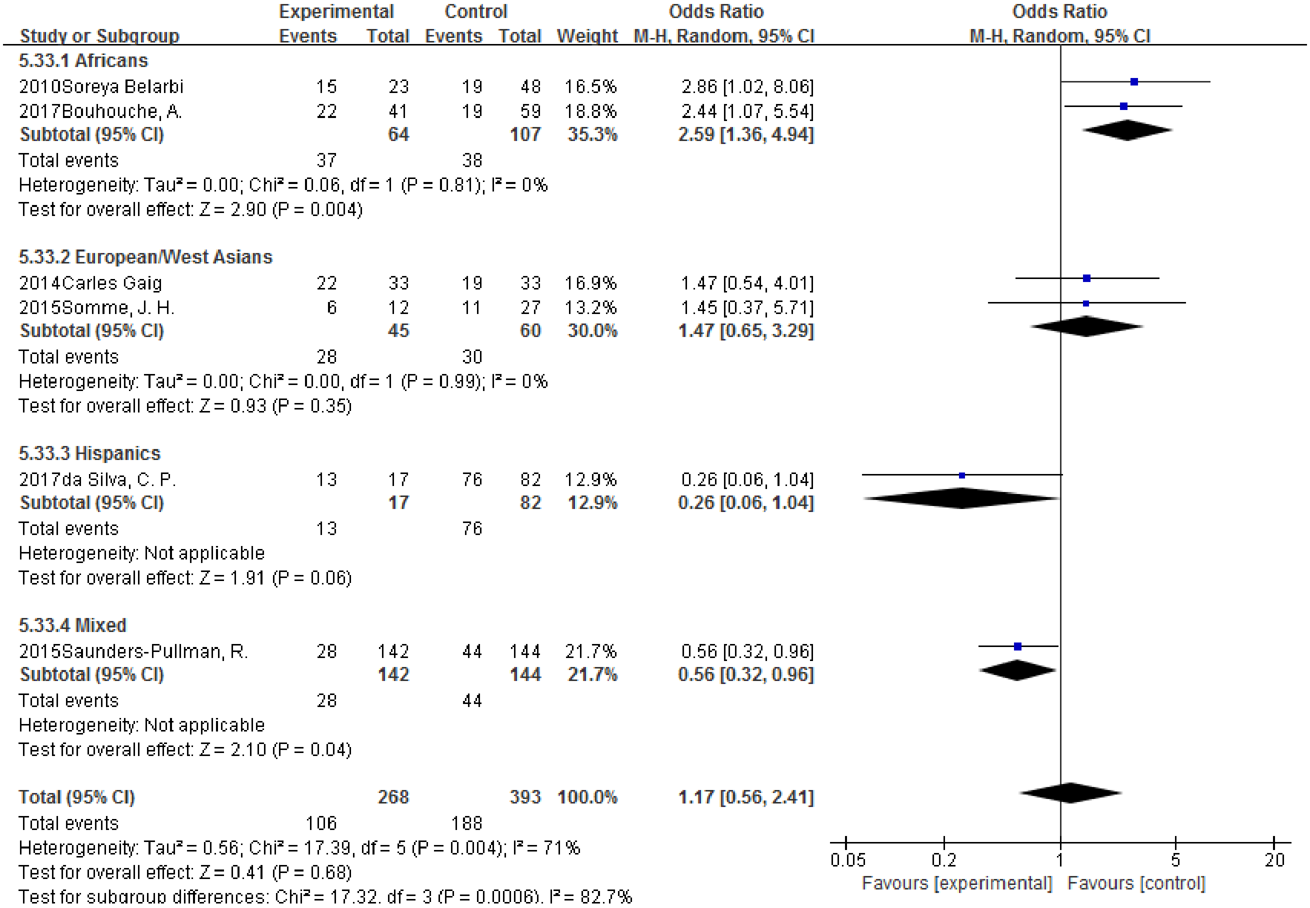


## 1.34 Sleep disturbances of G2019S by ethnicity

| Study or Subgroup | Experimental Events | Experimental Total | Control Events | Control Total | Weight | Odds Ratio M-H, Random, 95% CI |
|---|---|---|---|---|---|---|
| **5.34.1 European/West Asians** | | | | | | |
| 2014Carles Gaig | 13 | 33 | 25 | 33 | 16.4% | 0.21 [0.07, 0.60] |
| **Subtotal (95% CI)** | | **33** | | **33** | **16.4%** | **0.21 [0.07, 0.60]** |
| Total events | 13 | | 25 | | | |
| Heterogeneity: Not applicable | | | | | | |
| Test for overall effect: Z = 2.91 (P = 0.004) | | | | | | |
| **5.34.2 Hispanics** | | | | | | |
| 2017da Silva, C. P. | 11 | 17 | 21 | 39 | 15.8% | 1.57 [0.48, 5.10] |
| **Subtotal (95% CI)** | | **17** | | **39** | **15.8%** | **1.57 [0.48, 5.10]** |
| Total events | 11 | | 21 | | | |
| Heterogeneity: Not applicable | | | | | | |
| Test for overall effect: Z = 0.75 (P = 0.45) | | | | | | |
| **5.34.3 Mixed** | | | | | | |
| 2011R. Saunders-Pullman | 18 | 31 | 25 | 30 | 15.7% | 0.28 [0.08, 0.92] |
| 2014Rachel Saunders-Pullman | 69 | 126 | 101 | 126 | 18.6% | 0.30 [0.17, 0.53] |
| 2016Marras, C. | 135 | 137 | 194 | 212 | 14.2% | 6.26 [1.43, 27.44] |
| 2017San Luciano, M. | 160 | 530 | 131 | 759 | 19.3% | 2.07 [1.59, 2.70] |
| **Subtotal (95% CI)** | | **824** | | **1127** | **67.8%** | **0.97 [0.25, 3.75]** |
| Total events | 382 | | 451 | | | |
| Heterogeneity: Tau² = 1.67; Chi² = 48.49, df = 3 (P < 0.00001); I² = 94% | | | | | | |
| Test for overall effect: Z = 0.04 (P = 0.96) | | | | | | |
| **Total (95% CI)** | | **874** | | **1199** | **100.0%** | **0.81 [0.28, 2.36]** |
| Total events | 406 | | 497 | | | |
| Heterogeneity: Tau² = 1.52; Chi² = 60.67, df = 5 (P < 0.00001); I² = 92% | | | | | | |
| Test for overall effect: Z = 0.38 (P = 0.70) | | | | | | |
| Test for subgroup differences: Chi² = 6.90, df = 2 (P = 0.03), I² = 71.0% | | | | | | |

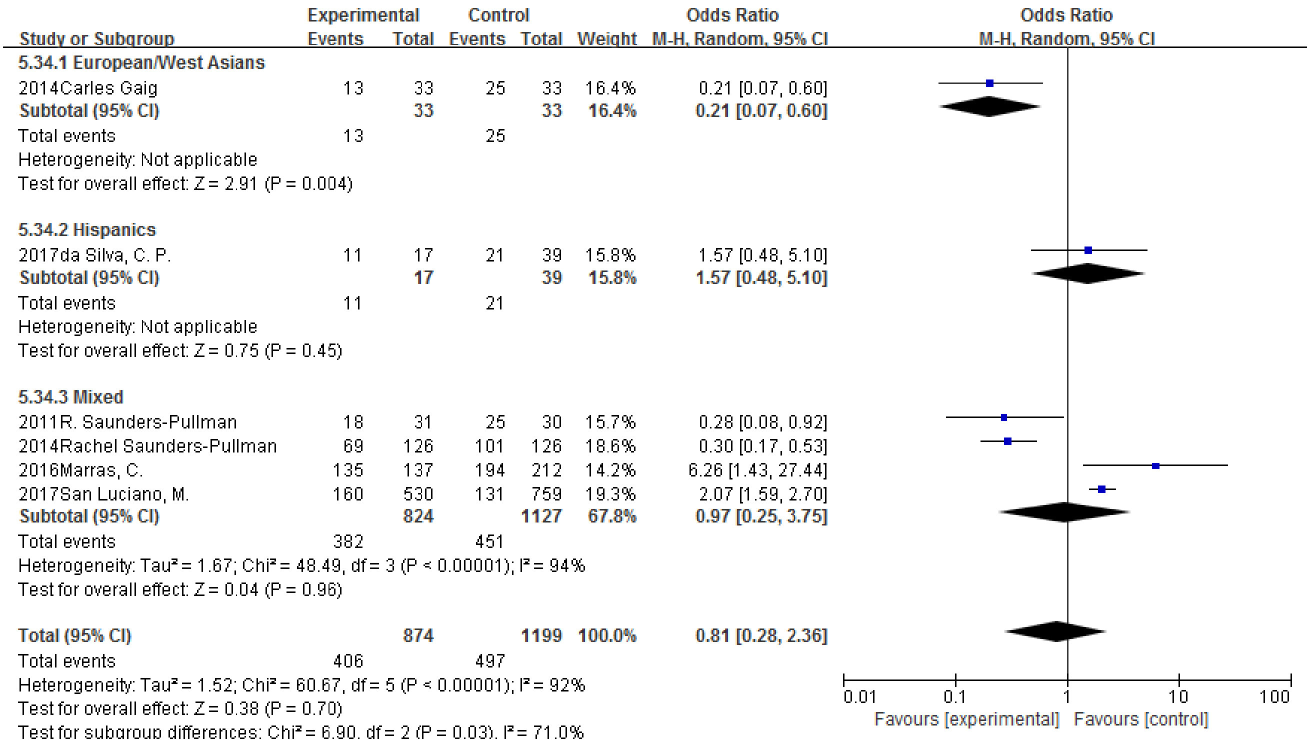


## 1.35 Olfactory disturbances of G2019S by ethnicity

| Study or Subgroup | Experimental Mean | Experimental SD | Experimental Total | Control Mean | Control SD | Control Total | Weight | Mean Difference IV, Fixed, 95% CI |
|---|---|---|---|---|---|---|---|---|
| **5.41.1 European/West Asians** | | | | | | | | |
| 2014Carles Gaig | 23.5 | 6.8 | 33 | 18.4 | 6 | 33 | 24.1% | 5.10 [2.01, 8.19] |
| **Subtotal (95% CI)** | | | **33** | | | **33** | **24.1%** | **5.10 [2.01, 8.19]** |
| Heterogeneity: Not applicable | | | | | | | | |
| Test for overall effect: Z = 3.23 (P = 0.001) | | | | | | | | |
| **5.41.2 Mixed** | | | | | | | | |
| 2011R. Saunders-Pullman | 24.8 | 7.08 | 31 | 18.8 | 8.05 | 30 | 15.9% | 6.00 [2.19, 9.81] |
| 2014Rachel Saunders-Pullman | 22.8 | 8.7 | 126 | 18.6 | 7.1 | 126 | 60.0% | 4.20 [2.24, 6.16] |
| **Subtotal (95% CI)** | | | **157** | | | **156** | **75.9%** | **4.58 [2.83, 6.32]** |
| Heterogeneity: Chi² = 0.68, df = 1 (P = 0.41); I² = 0% | | | | | | | | |
| Test for overall effect: Z = 5.15 (P < 0.00001) | | | | | | | | |
| **Total (95% CI)** | | | **190** | | | **189** | **100.0%** | **4.70 [3.18, 6.22]** |
| Heterogeneity: Chi² = 0.76, df = 2 (P = 0.68); I² = 0% | | | | | | | | |
| Test for overall effect: Z = 6.07 (P < 0.00001) | | | | | | | | |
| Test for subgroup differences: Chi² = 0.08, df = 1 (P = 0.77), I² = 0% | | | | | | | | |

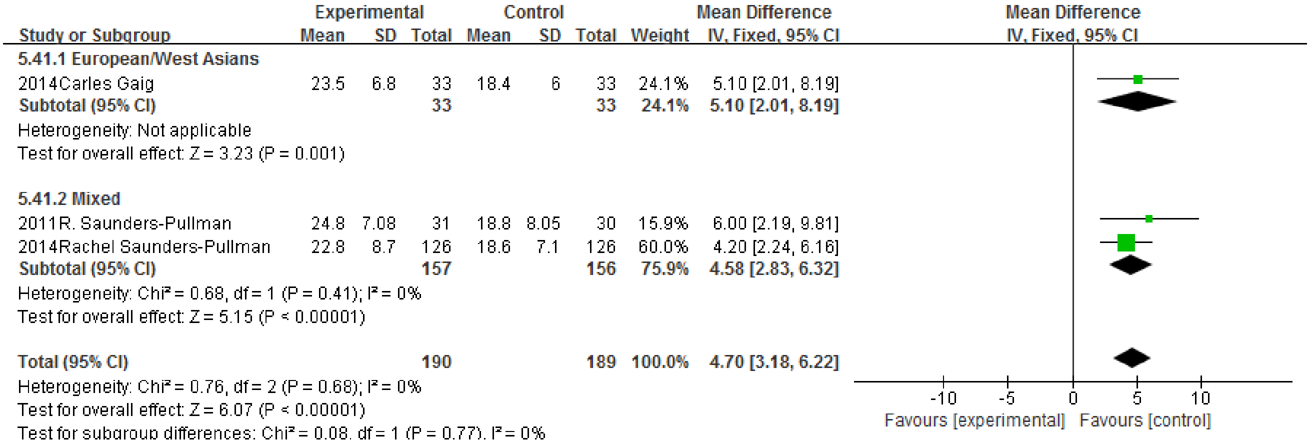


## 1.36 UPSIT scores of G2019S by ethnicity

| Study or Subgroup | Experimental Events | Experimental Total | Control Events | Control Total | Weight | Odds Ratio M-H, Fixed, 95% CI |
|---|---|---|---|---|---|---|
| **5.35.1 Africans** | | | | | | |
| 2006Lesage S | 18 | 33 | 34 | 73 | 33.9% | 1.38 [0.60, 3.14] |
| **Subtotal (95% CI)** | | **33** | | **73** | **33.9%** | **1.38 [0.60, 3.14]** |
| Total events | 18 | | 34 | | | |
| Heterogeneity: Not applicable | | | | | | |
| Test for overall effect: Z = 0.76 (P = 0.45) | | | | | | |
| **5.35.2 Hispanics** | | | | | | |
| 2017da Silva, C. P. | 16 | 16 | 83 | 91 | 2.7% | 3.36 [0.18, 61.09] |
| **Subtotal (95% CI)** | | **16** | | **91** | **2.7%** | **3.36 [0.18, 61.09]** |
| Total events | 16 | | 83 | | | |
| Heterogeneity: Not applicable | | | | | | |
| Test for overall effect: Z = 0.82 (P = 0.41) | | | | | | |
| **5.35.3 Mixed** | | | | | | |
| 2006L.N. Clark | 13 | 14 | 311 | 320 | 6.6% | 0.38 [0.04, 3.19] |
| 2011C. Marras | 23 | 24 | 65 | 69 | 4.9% | 1.42 [0.15, 13.33] |
| 2013Roy N. Alcalay | 35 | 97 | 58 | 391 | 51.9% | 3.24 [1.97, 5.34] |
| **Subtotal (95% CI)** | | **135** | | **780** | **63.4%** | **2.80 [1.72, 4.57]** |
| Total events | 71 | | 434 | | | |
| Heterogeneity: Chi² = 4.07, df = 2 (P = 0.13); I² = 51% | | | | | | |
| Test for overall effect: Z = 4.14 (P < 0.0001) | | | | | | |
| **Total (95% CI)** | | **184** | | **944** | **100.0%** | **2.33 [1.54, 3.54]** |
| Total events | 105 | | 551 | | | |
| Heterogeneity: Chi² = 6.28, df = 4 (P = 0.18); I² = 36% | | | | | | |
| Test for overall effect: Z = 4.00 (P < 0.0001) | | | | | | |
| Test for subgroup differences: Chi² = 2.17, df = 2 (P = 0.34), I² = 8.0% | | | | | | |

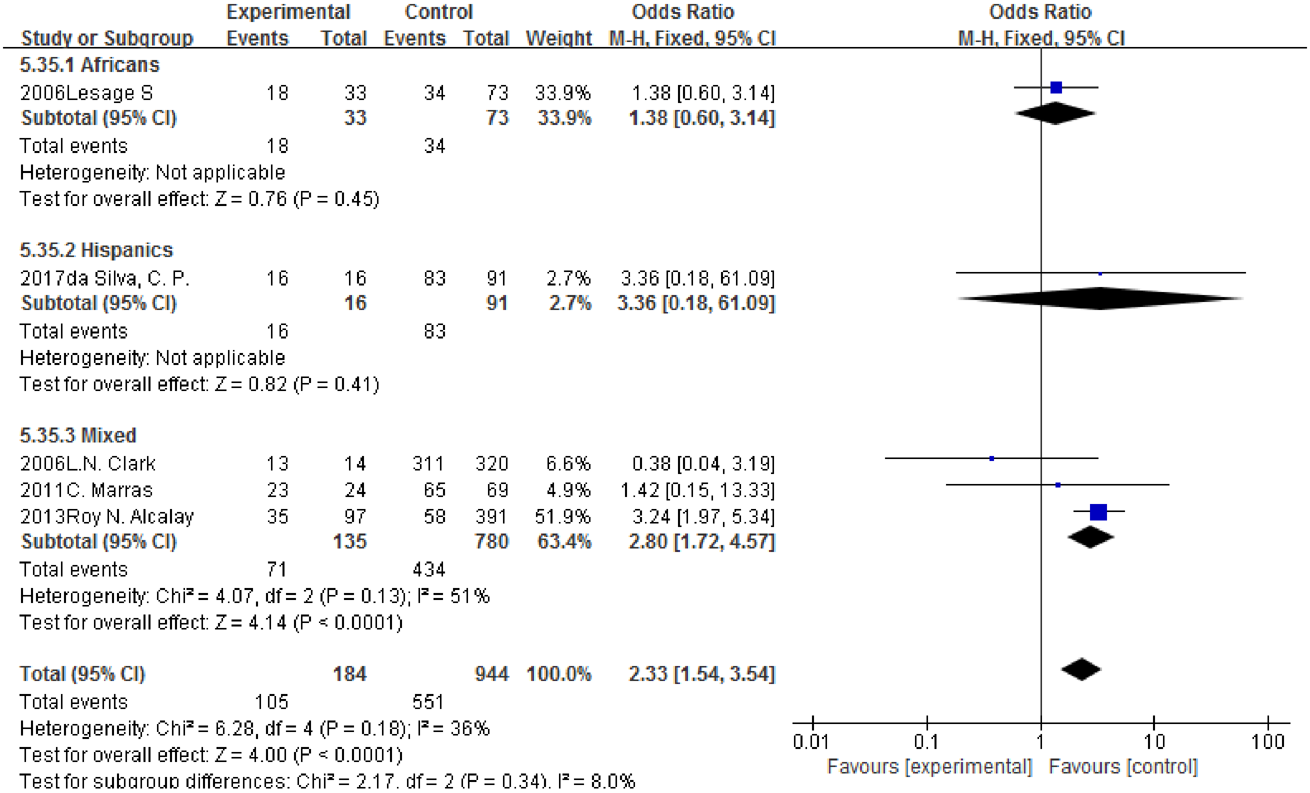


## 1.37 Good response to l-dopa of G2019S by ethnicity

| Study or Subgroup | Experimental Mean | Experimental SD | Experimental Total | Control Mean | Control SD | Control Total | Weight | Mean Difference IV, Fixed, 95% CI |
|---|---|---|---|---|---|---|---|---|
| **5.36.1 Africans** | | | | | | | | |
| 2006Lesage S | 697 | 322 | 30 | 601 | 356 | 48 | 4.0% | 96.00 [-57.03, 249.03] |
| 2008S. Lesage | 580 | 335 | 31 | 491 | 245 | 58 | 5.3% | 89.00 [-44.72, 222.72] |
| 2012Ben Sassi, S. | 558.1 | 321.7 | 55 | 517.7 | 229.5 | 55 | 8.7% | 40.40 [-64.04, 144.84] |
| **Subtotal (95% CI)** | | | **116** | | | **161** | **18.0%** | **67.16 [-5.33, 139.65]** |
| Heterogeneity: Chi² = 0.49, df = 2 (P = 0.78); I² = 0% | | | | | | | | |
| Test for overall effect: Z = 1.82 (P = 0.07) | | | | | | | | |
| **5.36.2 European/West Asians** | | | | | | | | |
| 2013Mirelman, A. | 300.1 | 251.9 | 50 | 191.8 | 262.8 | 50 | 9.3% | 108.30 [7.40, 209.20] |
| 2014Carles Gaig | 793.7 | 482.1 | 33 | 823 | 516.4 | 33 | 1.6% | -29.30 [-270.34, 211.74] |
| 2015Somme, J. H. | 1,066.3 | 388 | 12 | 857 | 325 | 27 | 1.5% | 209.30 [-42.14, 460.74] |
| **Subtotal (95% CI)** | | | **95** | | | **110** | **12.4%** | **102.43 [15.14, 189.71]** |
| Heterogeneity: Chi² = 1.85, df = 2 (P = 0.40); I² = 0% | | | | | | | | |
| Test for overall effect: Z = 2.30 (P = 0.02) | | | | | | | | |
| **5.36.3 Mixed** | | | | | | | | |
| 2009RN Alcalay | 556 | 624 | 26 | 471 | 492 | 665 | 1.6% | 85.00 [-157.75, 327.75] |
| 2011C. Marras | 631 | 410 | 25 | 585 | 423 | 73 | 2.7% | 46.00 [-141.74, 233.74] |
| 2013Roy N. Alcalay | 443 | 444 | 97 | 348 | 373 | 391 | 10.3% | 95.00 [-0.78, 190.78] |
| 2015Alcalay, R. N. | 389 | 402 | 116 | 220 | 288 | 120 | 11.8% | 169.00 [79.52, 258.48] |
| 2016Marras, C. | 539.31 | 578.5 | 516 | 366.13 | 373 | 527 | 26.9% | 173.18 [113.97, 232.39] |
| 2018Saunders-Pullman, R. | 407.39 | 410.23 | 144 | 337.1 | 367.58 | 401 | 16.3% | 70.29 [-5.76, 146.34] |
| **Subtotal (95% CI)** | | | **924** | | | **2177** | **69.6%** | **129.87 [93.04, 166.69]** |
| Heterogeneity: Chi² = 6.55, df = 5 (P = 0.26); I² = 24% | | | | | | | | |
| Test for overall effect: Z = 6.91 (P < 0.00001) | | | | | | | | |
| **Total (95% CI)** | | | **1135** | | | **2448** | **100.0%** | **115.20 [84.47, 145.93]** |
| Heterogeneity: Chi² = 11.28, df = 11 (P = 0.42); I² = 2% | | | | | | | | |
| Test for overall effect: Z = 7.35 (P < 0.00001) | | | | | | | | |
| Test for subgroup differences: Chi² = 2.38, df = 2 (P = 0.30), I² = 15.9% | | | | | | | | |

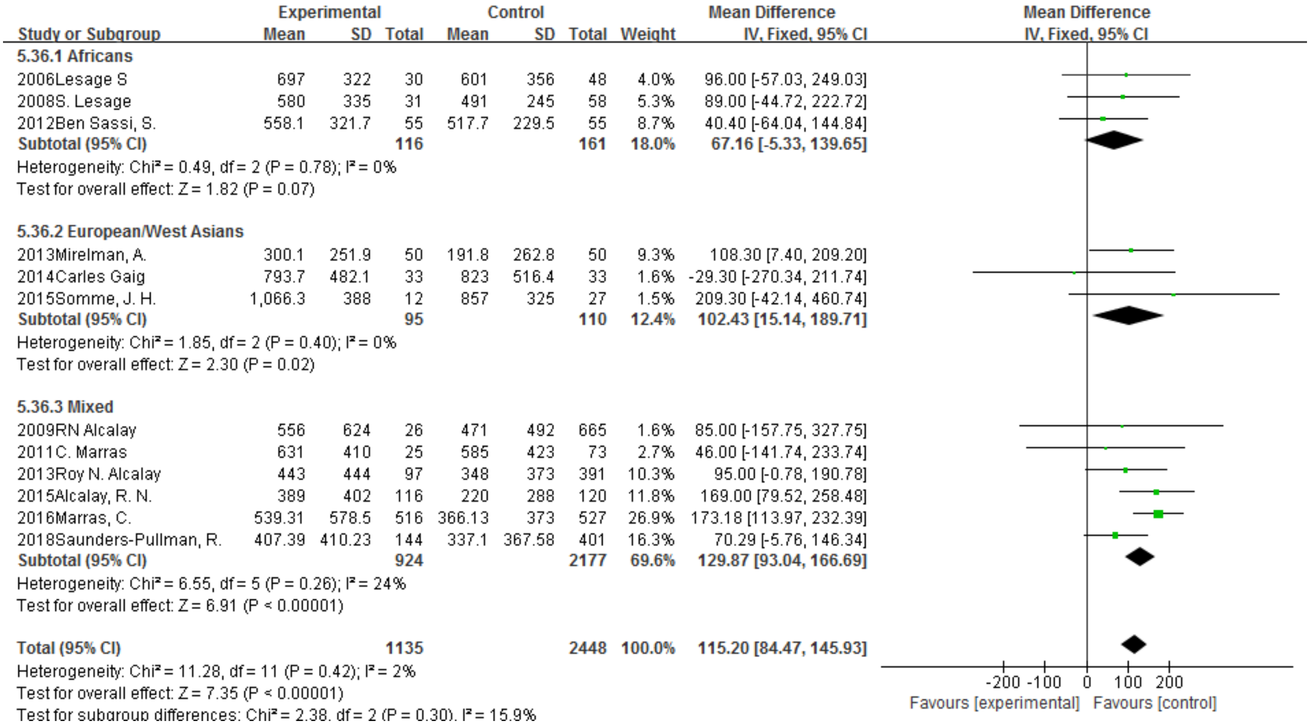


## 1.38 LEDD of G2019S by ethnicity

| Study or Subgroup | Experimental Events | Experimental Total | Control Events | Control Total | Weight | Odds Ratio M-H, Fixed, 95% CI |
|---|---|---|---|---|---|---|
| **5.42.1 Mixed** | | | | | | |
| 2011R. Saunders-Pullman | 11 | 31 | 11 | 29 | 6.6% | 0.90 [0.31, 2.57] |
| 2014Rachel Saunders-Pullman | 65 | 126 | 44 | 126 | 19.1% | 1.99 [1.20, 3.29] |
| 2016Marras, C. | 407 | 498 | 584 | 783 | 74.3% | 1.52 [1.15, 2.01] |
| **Subtotal (95% CI)** | | **655** | | **938** | **100.0%** | **1.57 [1.24, 1.99]** |
| Total events | 483 | | 639 | | | |
| Heterogeneity: Chi² = 1.95, df = 2 (P = 0.38); I² = 0% | | | | | | |
| Test for overall effect: Z = 3.73 (P = 0.0002) | | | | | | |
| | | | | | | |
| **Total (95% CI)** | | **655** | | **938** | **100.0%** | **1.57 [1.24, 1.99]** |
| Total events | 483 | | 639 | | | |
| Heterogeneity: Chi² = 1.95, df = 2 (P = 0.38); I² = 0% | | | | | | |
| Test for overall effect: Z = 3.73 (P = 0.0002) | | | | | | |
| Test for subgroup differences: Not applicable | | | | | | |

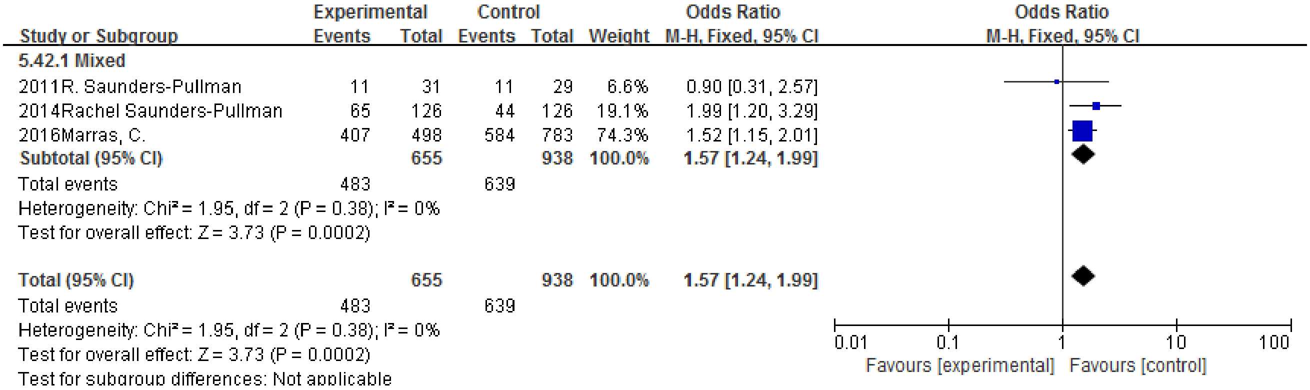


## 1.39 Smoke of G2019S by ethnicity

| Study or Subgroup | Experimental Events | Experimental Total | Control Events | Control Total | Weight | Odds Ratio M-H, Random, 95% CI |
|---|---|---|---|---|---|---|
| **7.17.1 East Asians** | | | | | | |
| 2006Hon-Chung Fung | 20 | 27 | 203 | 278 | 37.0% | 1.06 [0.43, 2.60] |
| 2008Daniel Kam Yin Chan | 3 | 3 | 28 | 31 | 25.3% | 0.86 [0.04, 20.35] |
| 2016Sun, Qian | 20 | 76 | 203 | 225 | 37.7% | 0.04 [0.02, 0.08] |
| **Subtotal (95% CI)** | | **106** | | **534** | **100.0%** | **0.29 [0.02, 4.33]** |
| Total events | 43 | | 434 | | | |
| Heterogeneity: Tau² = 4.95; Chi² = 34.89, df = 2 (P < 0.00001); I² = 94% | | | | | | |
| Test for overall effect: Z = 0.90 (P = 0.37) | | | | | | |
| | | | | | | |
| **Total (95% CI)** | | **106** | | **534** | **100.0%** | **0.29 [0.02, 4.33]** |
| Total events | 43 | | 434 | | | |
| Heterogeneity: Tau² = 4.95; Chi² = 34.89, df = 2 (P < 0.00001); I² = 94% | | | | | | |
| Test for overall effect: Z = 0.90 (P = 0.37) | | | | | | |
| Test for subgroup differences: Not applicable | | | | | | |

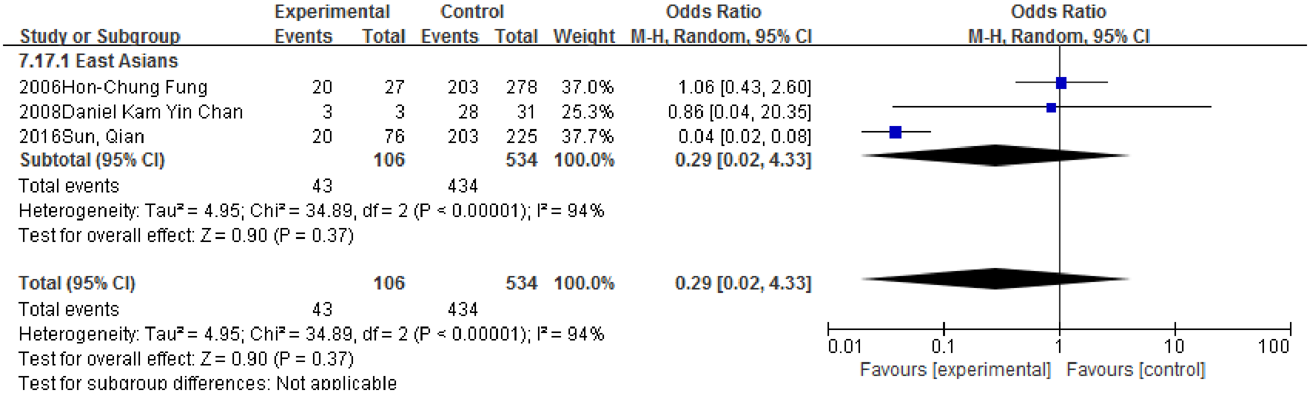


## 1.40 Asymmetrical onset of G2385R by ethnicity

| Study or Subgroup | Experimental Mean | Experimental SD | Experimental Total | Control Mean | Control SD | Control Total | Weight | Mean Difference IV, Random, 95% CI |
|---|---|---|---|---|---|---|---|---|
| **7.1.1 East Asians** | | | | | | | | |
| 2006Hon-Chung Fung | 61.5 | 11.3 | 27 | 61.9 | 10.5 | 278 | 6.1% | -0.40 [-4.84, 4.04] |
| 2006Manabu Funayama | 54 | 10.6 | 50 | 50.3 | 14.9 | 389 | 8.6% | 3.70 [0.41, 6.99] |
| 2007Cao Li | 57 | 11 | 14 | 54.4 | 13.4 | 221 | 3.9% | 2.60 [-3.43, 8.63] |
| 2007Eng-King Tan | 59 | 11 | 33 | 59 | 10 | 29 | 4.8% | 0.00 [-5.23, 5.23] |
| 2007Matthew J. Farrer | 54.9 | 11.8 | 34 | 58 | 11.7 | 376 | 6.6% | -3.10 [-7.24, 1.04] |
| 2008X.-K. An | 52.75 | 9.8 | 71 | 56.32 | 11 | 529 | 11.0% | -3.57 [-6.03, -1.11] |
| 2010Jong-Min Kim | 55.3 | 11.5 | 82 | 54.7 | 10.8 | 841 | 10.6% | 0.60 [-1.99, 3.19] |
| 2013Chao Gao | 60.5 | 10.4 | 36 | 58.2 | 8.8 | 139 | 7.6% | 2.30 [-1.40, 6.00] |
| 2013Jiangping Cai | 56.8 | 10.7 | 49 | 58.6 | 11 | 461 | 8.9% | -1.80 [-4.96, 1.36] |
| 2013Xiaoli Fu | 55 | 11.7 | 37 | 55 | 11 | 409 | 7.1% | 0.00 [-3.92, 3.92] |
| 2016Sun, Qian | 55.57 | 8.78 | 76 | 57.05 | 8.92 | 225 | 11.6% | -1.48 [-3.77, 0.81] |
| **Subtotal (95% CI)** | | | **509** | | | **3897** | **86.7%** | **-0.35 [-1.79, 1.09]** |
| Heterogeneity: Tau² = 2.66; Chi² = 19.07, df = 10 (P = 0.04); I² = 48% | | | | | | | | |
| Test for overall effect: Z = 0.47 (P = 0.64) | | | | | | | | |
| | | | | | | | | |
| **7.1.2 Mixed** | | | | | | | | |
| 2016Marras, C. | 55.99 | 11.1 | 197 | 58.69 | 12.5 | 790 | 13.3% | -2.70 [-4.48, -0.92] |
| **Subtotal (95% CI)** | | | **197** | | | **790** | **13.3%** | **-2.70 [-4.48, -0.92]** |
| Heterogeneity: Not applicable | | | | | | | | |
| Test for overall effect: Z = 2.98 (P = 0.003) | | | | | | | | |
| | | | | | | | | |
| **Total (95% CI)** | | | **706** | | | **4687** | **100.0%** | **-0.66 [-2.01, 0.70]** |
| Heterogeneity: Tau² = 2.76; Chi² = 23.25, df = 11 (P = 0.02); I² = 53% | | | | | | | | |
| Test for overall effect: Z = 0.95 (P = 0.34) | | | | | | | | |
| Test for subgroup differences: Chi² = 4.06, df = 1 (P = 0.04), I² = 75.3% | | | | | | | | |

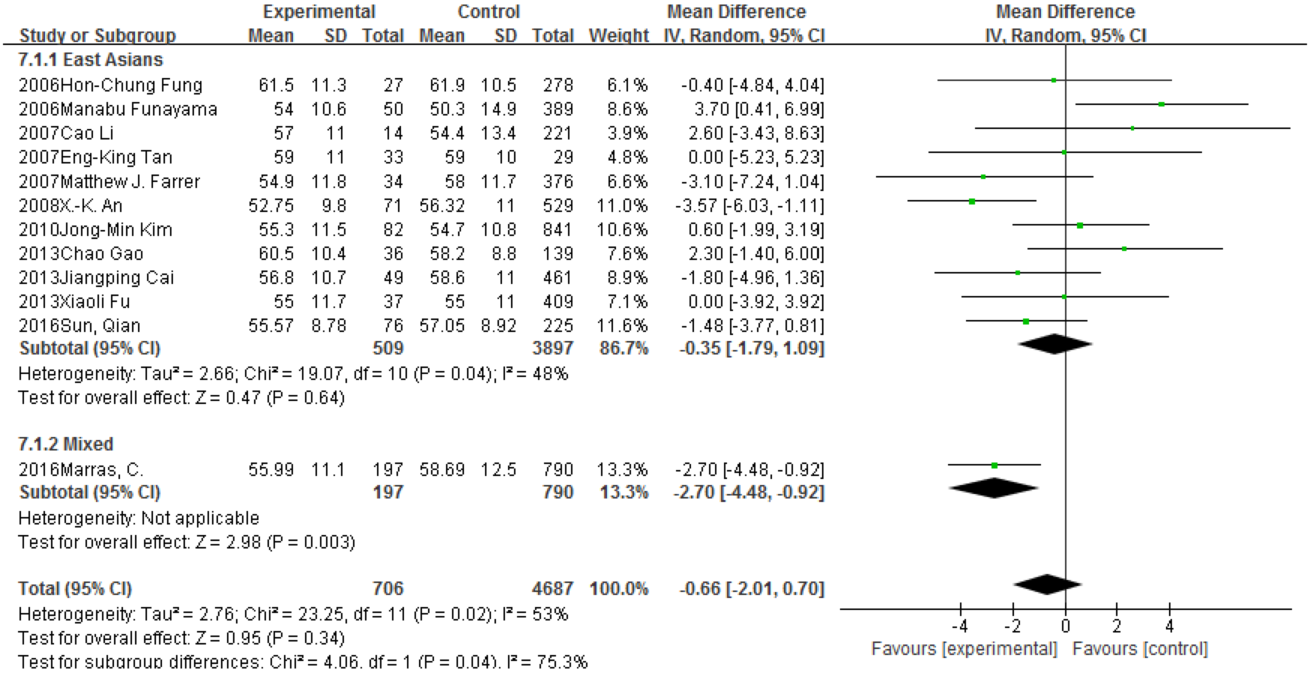


## 1.41 Age at onset of G2385R by ethnicity

| Study or Subgroup | Experimental Events | Experimental Total | Control Events | Control Total | Weight | Odds Ratio M-H, Fixed, 95% CI |
|---|---|---|---|---|---|---|
| **7.2.1 East Asians** | | | | | | |
| 2006Di Fonzo, A. | 23 | 61 | 192 | 547 | 21.3% | 1.12 [0.65, 1.93] |
| 2006Manabu Funayama | 17 | 50 | 180 | 389 | 24.0% | 0.60 [0.32, 1.11] |
| 2007Cao Li | 4 | 14 | 76 | 221 | 5.7% | 0.76 [0.23, 2.51] |
| 2008X.-K. An | 26 | 71 | 124 | 529 | 16.5% | 1.89 [1.12, 3.18] |
| 2012HUIRU YAN | 5 | 21 | 72 | 333 | 5.8% | 1.13 [0.40, 3.20] |
| 2013Chao Gao | 5 | 36 | 5 | 139 | 1.6% | 4.32 [1.18, 15.86] |
| 2013Jiangping Cai | 11 | 49 | 105 | 461 | 13.9% | 0.98 [0.48, 1.99] |
| 2013Xiaoli Fu | 11 | 37 | 109 | 409 | 11.3% | 1.16 [0.56, 2.44] |
| **Subtotal (95% CI)** | | **339** | | **3028** | **100.0%** | **1.14 [0.89, 1.46]** |
| Total events | 102 | | 863 | | | |
| Heterogeneity: Chi² = 12.41, df = 7 (P = 0.09); I² = 44% | | | | | | |
| Test for overall effect: Z = 1.01 (P = 0.31) | | | | | | |
| **Total (95% CI)** | | **339** | | **3028** | **100.0%** | **1.14 [0.89, 1.46]** |
| Total events | 102 | | 863 | | | |
| Heterogeneity: Chi² = 12.41, df = 7 (P = 0.09); I² = 44% | | | | | | |
| Test for overall effect: Z = 1.01 (P = 0.31) | | | | | | |
| Test for subgroup differences: Not applicable | | | | | | |

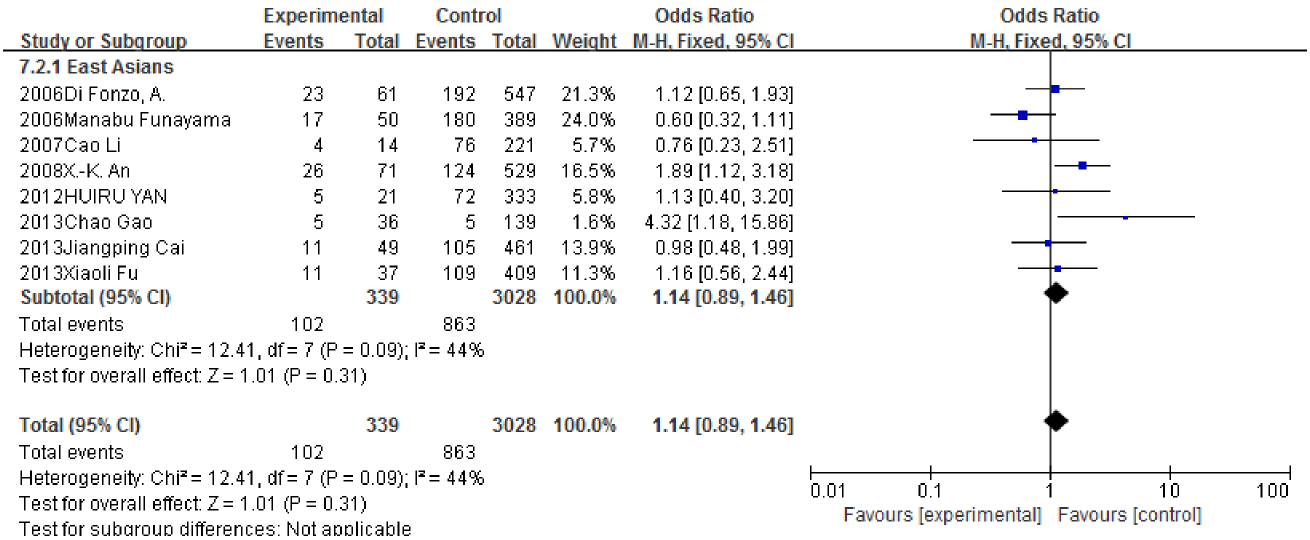


## 1.42 EOPD of G2385R by ethnicity

| Study or Subgroup | Experimental Events | Experimental Total | Control Events | Control Total | Weight | Odds Ratio M-H, Random, 95% CI |
|---|---|---|---|---|---|---|
| **7.3.1 East Asians** | | | | | | |
| 2006Di Fonzo, A. | 34 | 61 | 328 | 547 | 8.4% | 0.84 [0.49, 1.43] |
| 2007Cao Li | 5 | 14 | 128 | 221 | 4.5% | 0.40 [0.13, 1.24] |
| 2007Eng-King Tan | 16 | 33 | 17 | 29 | 5.1% | 0.66 [0.24, 1.82] |
| 2007Matthew J. Farrer | 18 | 34 | 214 | 376 | 7.1% | 0.85 [0.42, 1.72] |
| 2008Daniel Kam Yin Chan | 3 | 3 | 21 | 31 | 0.9% | 3.42 [0.16, 72.45] |
| 2008X.-K. An | 40 | 71 | 314 | 529 | 8.7% | 0.88 [0.54, 1.46] |
| 2010Jong-Min Kim | 35 | 82 | 373 | 841 | 9.0% | 0.93 [0.59, 1.48] |
| 2012HUIRU YAN | 10 | 21 | 192 | 333 | 5.8% | 0.67 [0.28, 1.62] |
| 2013Chao Gao | 21 | 36 | 15 | 139 | 6.0% | 11.57 [4.94, 27.13] |
| 2013Jiangping Cai | 28 | 49 | 275 | 461 | 7.9% | 0.90 [0.50, 1.64] |
| 2013Xiaoli Fu | 21 | 37 | 201 | 409 | 7.3% | 1.36 [0.69, 2.68] |
| 2016Cao, M. | 16 | 25 | 28 | 43 | 5.0% | 0.95 [0.34, 2.67] |
| 2016Sun, Qian | 37 | 76 | 125 | 225 | 8.5% | 0.76 [0.45, 1.28] |
| 2017Hong, J. H. | 7 | 23 | 136 | 276 | 5.6% | 0.45 [0.18, 1.13] |
| **Subtotal (95% CI)** | | **565** | | **4460** | **89.9%** | **0.97 [0.68, 1.38]** |
| Total events | 291 | | 2367 | | | |
| Heterogeneity: Tau² = 0.28; Chi² = 41.79, df = 13 (P < 0.0001); I² = 69% | | | | | | |
| Test for overall effect: Z = 0.15 (P = 0.88) | | | | | | |
| **7.3.2 Mixed** | | | | | | |
| 2016Marras, C. | 122 | 199 | 465 | 790 | 10.1% | 1.11 [0.81, 1.52] |
| **Subtotal (95% CI)** | | **199** | | **790** | **10.1%** | **1.11 [0.81, 1.52]** |
| Total events | 122 | | 465 | | | |
| Heterogeneity: Not applicable | | | | | | |
| Test for overall effect: Z = 0.63 (P = 0.53) | | | | | | |
| **Total (95% CI)** | | **764** | | **5250** | **100.0%** | **0.98 [0.73, 1.34]** |
| Total events | 413 | | 2832 | | | |
| Heterogeneity: Tau² = 0.21; Chi² = 42.45, df = 14 (P = 0.0001); I² = 67% | | | | | | |
| Test for overall effect: Z = 0.10 (P = 0.92) | | | | | | |
| Test for subgroup differences: Chi² = 0.28, df = 1 (P = 0.60), I² = 0% | | | | | | |

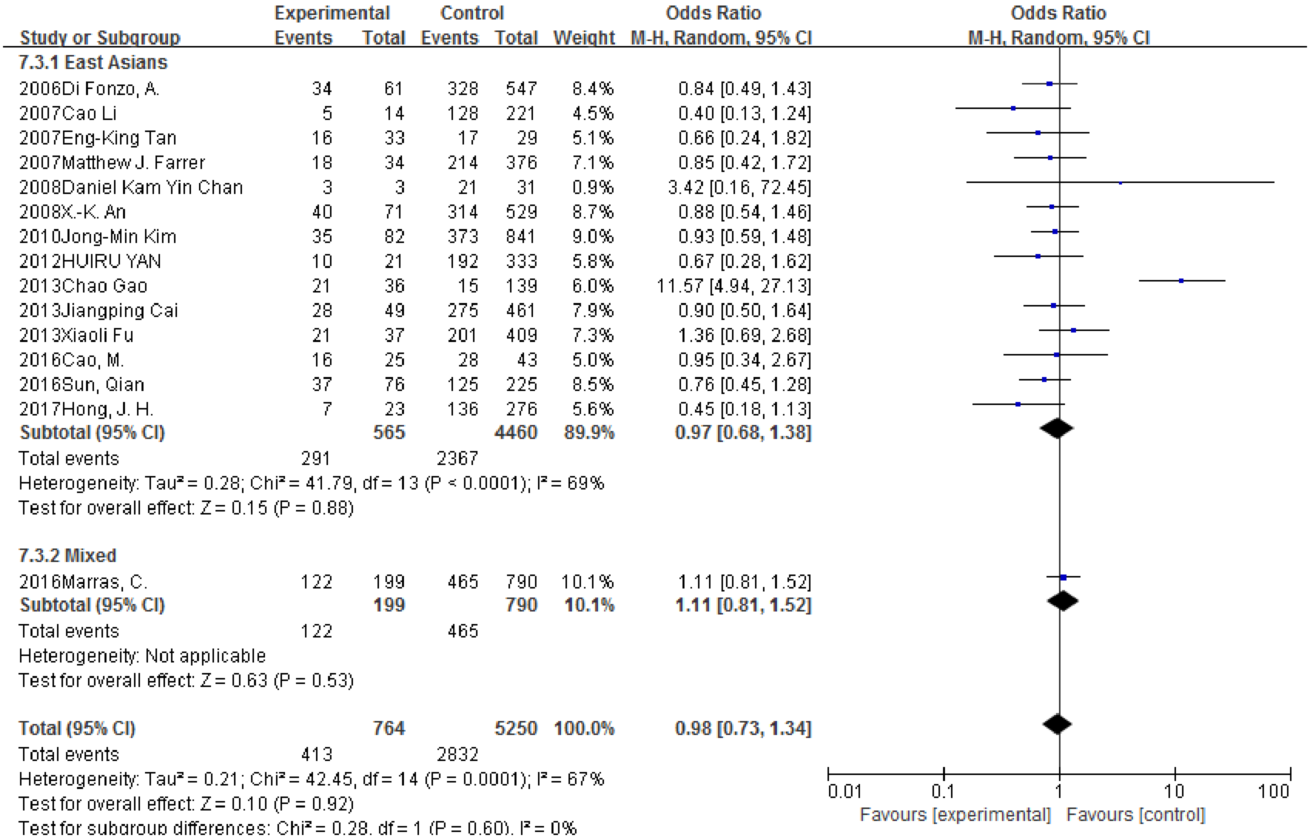


## 1.43 Gender-male of G2385R by ethnicity

| Study or Subgroup | Experimental Events | Experimental Total | Control Events | Control Total | Weight | Odds Ratio M-H, Fixed, 95% CI |
|---|---|---|---|---|---|---|
| **7.4.1 East Asians** | | | | | | |
| 2007Cao Li | 0 | 14 | 13 | 221 | 10.8% | 0.53 [0.03, 9.42] |
| 2007Eng-King Tan | 5 | 33 | 0 | 29 | 2.9% | 11.39 [0.60, 215.48] |
| 2007Matthew J. Farrer | 6 | 34 | 20 | 376 | 17.8% | 3.81 [1.42, 10.27] |
| 2013Chao Gao | 2 | 36 | 10 | 139 | 25.3% | 0.76 [0.16, 3.63] |
| 2013Xiaoli Fu | 8 | 37 | 51 | 409 | 43.2% | 1.94 [0.84, 4.47] |
| **Subtotal (95% CI)** | | **154** | | **1174** | **100.0%** | **2.10 [1.22, 3.59]** |
| Total events | 21 | | 94 | | | |
| Heterogeneity: Chi² = 5.21, df = 4 (P = 0.27); I² = 23% | | | | | | |
| Test for overall effect: Z = 2.69 (P = 0.007) | | | | | | |
| **Total (95% CI)** | | **154** | | **1174** | **100.0%** | **2.10 [1.22, 3.59]** |
| Total events | 21 | | 94 | | | |
| Heterogeneity: Chi² = 5.21, df = 4 (P = 0.27); I² = 23% | | | | | | |
| Test for overall effect: Z = 2.69 (P = 0.007) | | | | | | |
| Test for subgroup differences: Not applicable | | | | | | |

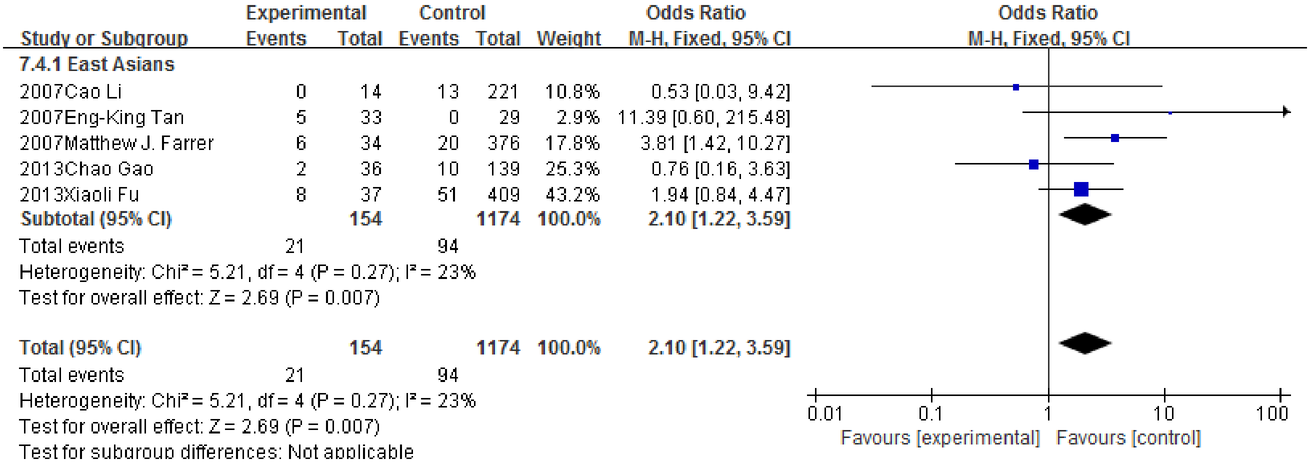


## 1.44 Family history of G2385R by ethnicity

| Study or Subgroup | Experimental Events | Experimental Total | Control Events | Control Total | Weight | Odds Ratio M-H, Random, 95% CI |
|---|---|---|---|---|---|---|
| **7.5.1 East Asians** | | | | | | |
| 2007Cao Li | 7 | 14 | 102 | 221 | 20.9% | 1.17 [0.40, 3.44] |
| 2007Matthew J. Farrer | 30 | 34 | 365 | 376 | 18.7% | 0.23 [0.07, 0.75] |
| 2013Jiangping Cai | 24 | 49 | 224 | 461 | 32.1% | 1.02 [0.56, 1.83] |
| 2013Xiaoli Fu | 11 | 37 | 81 | 409 | 28.3% | 1.71 [0.81, 3.61] |
| **Subtotal (95% CI)** | | **134** | | **1467** | **100.0%** | **0.92 [0.45, 1.85]** |
| Total events | 72 | | 772 | | | |
| Heterogeneity: Tau² = 0.31; Chi² = 7.97, df = 3 (P = 0.05); I² = 62% | | | | | | |
| Test for overall effect: Z = 0.25 (P = 0.80) | | | | | | |
| **Total (95% CI)** | | **134** | | **1467** | **100.0%** | **0.92 [0.45, 1.85]** |
| Total events | 72 | | 772 | | | |
| Heterogeneity: Tau² = 0.31; Chi² = 7.97, df = 3 (P = 0.05); I² = 62% | | | | | | |
| Test for overall effect: Z = 0.25 (P = 0.80) | | | | | | |
| Test for subgroup differences: Not applicable | | | | | | |

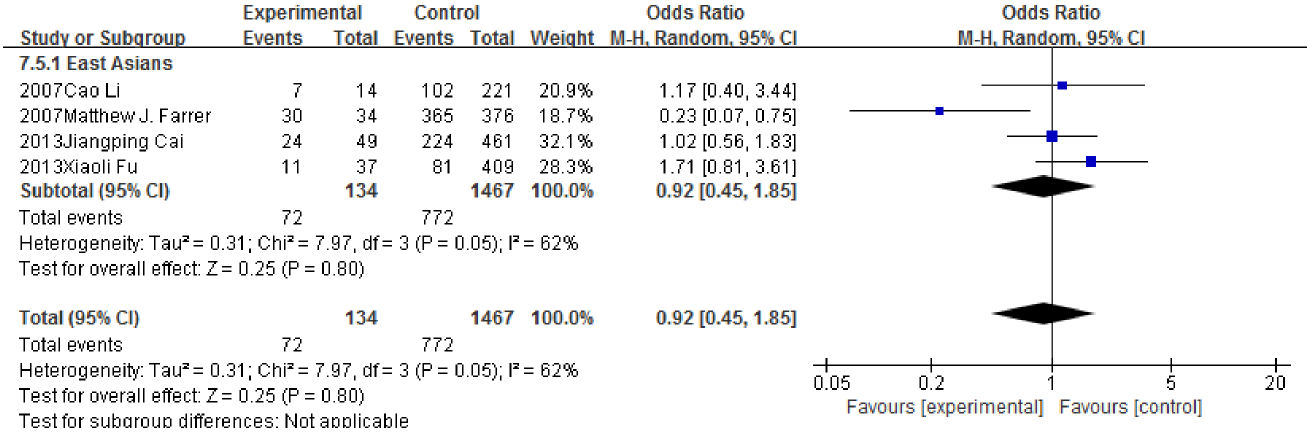


## 1.45 FS-Bradykinesia of G2385R by ethnicity

| Study or Subgroup | Experimental Events | Experimental Total | Control Events | Control Total | Weight | Odds Ratio M-H, Fixed, 95% CI |
|---|---|---|---|---|---|---|
| **7.6.1 East Asians** | | | | | | |
| 2007Cao Li | 8 | 14 | 134 | 221 | 9.8% | 0.87 [0.29, 2.58] |
| 2008X.-K. An | 37 | 71 | 263 | 529 | 42.6% | 1.10 [0.67, 1.81] |
| 2013Jiangping Cai | 34 | 49 | 299 | 461 | 25.1% | 1.23 [0.65, 2.32] |
| 2013Xiaoli Fu | 21 | 37 | 220 | 409 | 22.5% | 1.13 [0.57, 2.22] |
| **Subtotal (95% CI)** | | **171** | | **1620** | **100.0%** | **1.12 [0.81, 1.54]** |
| Total events | 100 | | 916 | | | |
| Heterogeneity: Chi² = 0.30, df = 3 (P = 0.96); I² = 0% | | | | | | |
| Test for overall effect: Z = 0.66 (P = 0.51) | | | | | | |
| **Total (95% CI)** | | **171** | | **1620** | **100.0%** | **1.12 [0.81, 1.54]** |
| Total events | 100 | | 916 | | | |
| Heterogeneity: Chi² = 0.30, df = 3 (P = 0.96); I² = 0% | | | | | | |
| Test for overall effect: Z = 0.66 (P = 0.51) | | | | | | |
| Test for subgroup differences: Not applicable | | | | | | |

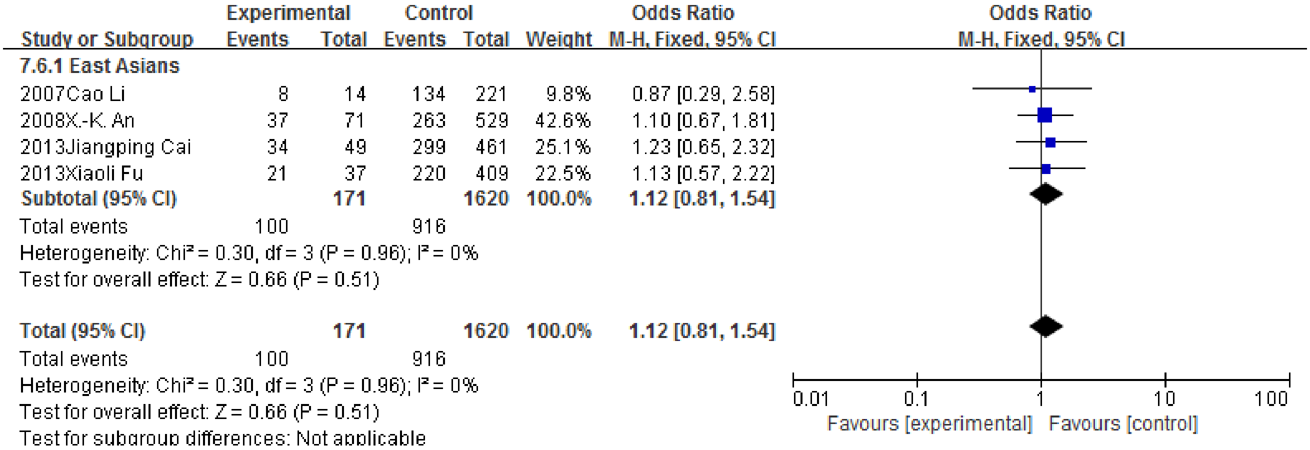


## 1.46 FS-Resting tremor of G2385R by ethnicity

| Study or Subgroup | Experimental Events | Experimental Total | Control Events | Control Total | Weight | Odds Ratio M-H, Fixed, 95% CI |
|---|---|---|---|---|---|---|
| **7.18.1 East Asians** | | | | | | |
| 2007Cao Li | 7 | 14 | 83 | 221 | 16.9% | 1.66 [0.56, 4.91] |
| 2013Jiangping Cai | 18 | 49 | 168 | 461 | 69.6% | 1.01 [0.55, 1.87] |
| 2013Xiaoli Fu | 3 | 37 | 26 | 409 | 13.5% | 1.30 [0.37, 4.52] |
| **Subtotal (95% CI)** | | **100** | | **1091** | **100.0%** | **1.16 [0.71, 1.89]** |
| Total events | 28 | | 277 | | | |
| Heterogeneity: Chi² = 0.65, df = 2 (P = 0.72); I² = 0% | | | | | | |
| Test for overall effect: Z = 0.60 (P = 0.55) | | | | | | |
| **Total (95% CI)** | | **100** | | **1091** | **100.0%** | **1.16 [0.71, 1.89]** |
| Total events | 28 | | 277 | | | |
| Heterogeneity: Chi² = 0.65, df = 2 (P = 0.72); I² = 0% | | | | | | |
| Test for overall effect: Z = 0.60 (P = 0.55) | | | | | | |
| Test for subgroup differences: Not applicable | | | | | | |

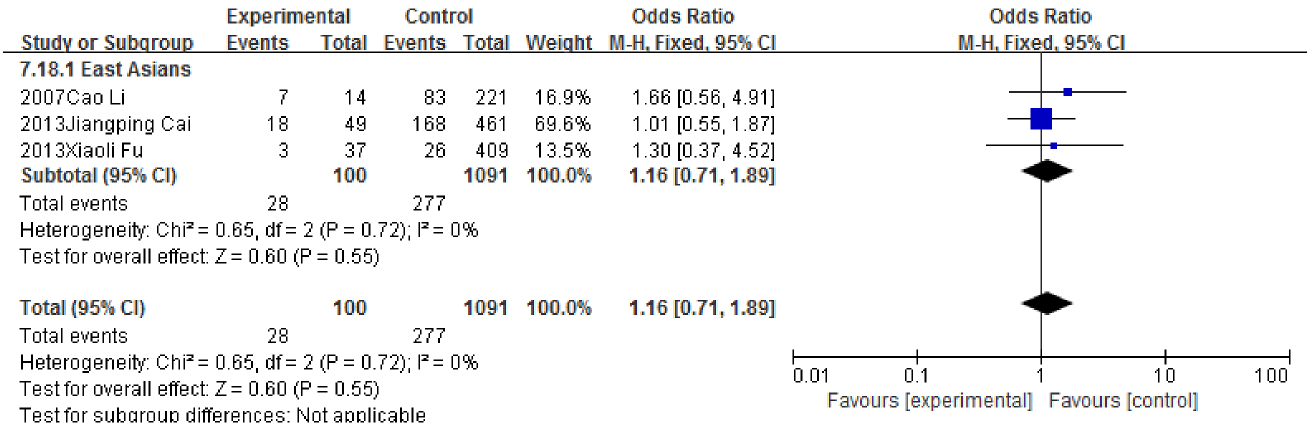


## 1.47 FS-Rigidity of G2385R by ethnicity

| Study or Subgroup | Experimental Events | Experimental Total | Control Events | Control Total | Weight | Odds Ratio M-H, Fixed, 95% CI |
|---|---|---|---|---|---|---|
| **7.19.1 East Asians** | | | | | | |
| 2007Cao Li | 5 | 14 | 64 | 221 | 52.6% | 1.36 [0.44, 4.22] |
| 2013Jiangping Cai | 0 | 49 | 9 | 461 | 19.7% | 0.48 [0.03, 8.39] |
| 2013Xiaoli Fu | 1 | 37 | 16 | 409 | 27.7% | 0.68 [0.09, 5.29] |
| **Subtotal (95% CI)** | | **100** | | **1091** | **100.0%** | **1.00 [0.40, 2.48]** |
| Total events | 6 | | 89 | | | |
| Heterogeneity: Chi² = 0.67, df = 2 (P = 0.71); I² = 0% | | | | | | |
| Test for overall effect: Z = 0.00 (P = 1.00) | | | | | | |
| **Total (95% CI)** | | **100** | | **1091** | **100.0%** | **1.00 [0.40, 2.48]** |
| Total events | 6 | | 89 | | | |
| Heterogeneity: Chi² = 0.67, df = 2 (P = 0.71); I² = 0% | | | | | | |
| Test for overall effect: Z = 0.00 (P = 1.00) | | | | | | |
| Test for subgroup differences: Not applicable | | | | | | |

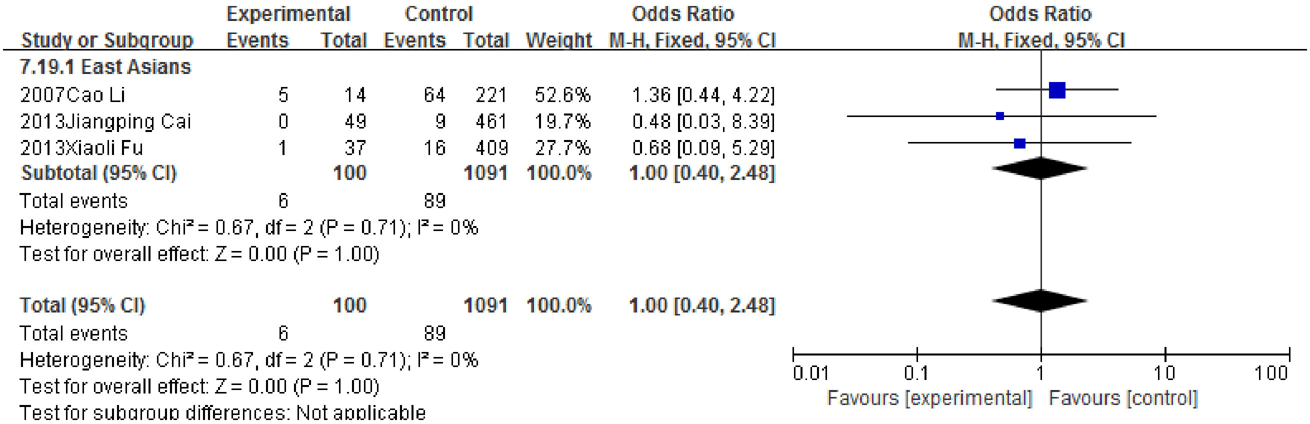


## 1.48 FS-Postural instability or Gait difficulty of G2385R by ethnicity

| Study or Subgroup | Experimental Events | Experimental Total | Control Events | Control Total | Weight | Odds Ratio M-H, Fixed, 95% CI |
|---|---|---|---|---|---|---|
| **7.7.1 East Asians** | | | | | | |
| 2006Hon-Chung Fung | 26 | 27 | 252 | 278 | 12.0% | 2.68 [0.35, 20.58] |
| 2007Matthew J. Farrer | 25 | 34 | 258 | 376 | 81.9% | 1.27 [0.58, 2.81] |
| 2008Daniel Kam Yin Chan | 3 | 3 | 30 | 31 | 6.1% | 0.34 [0.01, 10.18] |
| **Subtotal (95% CI)** | | **64** | | **685** | **100.0%** | **1.38 [0.68, 2.82]** |
| Total events | 54 | | 540 | | | |
| Heterogeneity: Chi² = 1.10, df = 2 (P = 0.58); I² = 0% | | | | | | |
| Test for overall effect: Z = 0.89 (P = 0.37) | | | | | | |
| **Total (95% CI)** | | **64** | | **685** | **100.0%** | **1.38 [0.68, 2.82]** |
| Total events | 54 | | 540 | | | |
| Heterogeneity: Chi² = 1.10, df = 2 (P = 0.58); I² = 0% | | | | | | |
| Test for overall effect: Z = 0.89 (P = 0.37) | | | | | | |
| Test for subgroup differences: Not applicable | | | | | | |

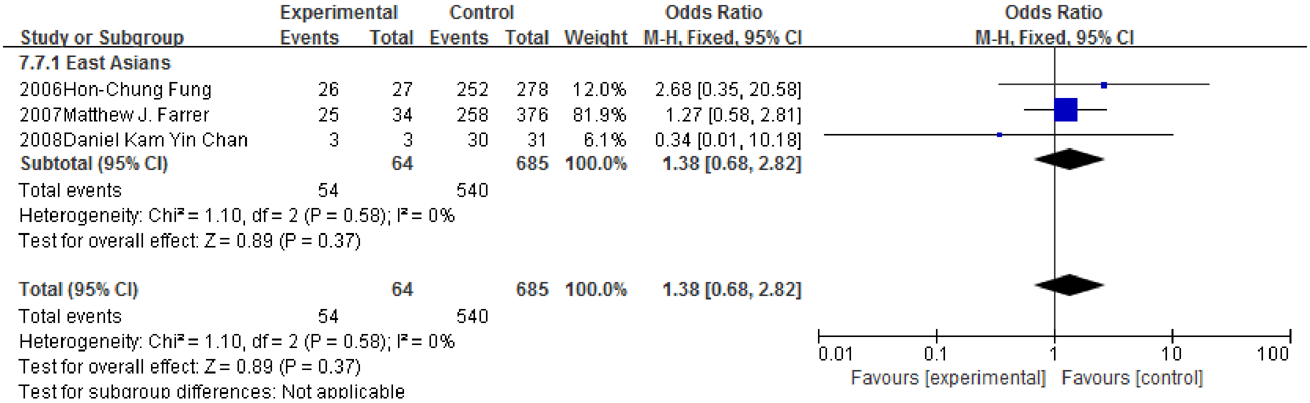


## 1.49 Resting tremor of G2385R by ethnicity

| Study or Subgroup | Experimental Events | Experimental Total | Control Events | Control Total | Weight | Odds Ratio M-H, Fixed, 95% CI |
|---|---|---|---|---|---|---|
| **7.8.1 East Asians** | | | | | | |
| 2006Hon-Chung Fung | 26 | 27 | 261 | 278 | 29.4% | 1.69 [0.22, 13.24] |
| 2007Matthew J. Farrer | 32 | 34 | 343 | 376 | 57.5% | 1.54 [0.35, 6.71] |
| 2008Daniel Kam Yin Chan | 3 | 3 | 27 | 31 | 13.1% | 1.15 [0.05, 26.10] |
| **Subtotal (95% CI)** | | **64** | | **685** | **100.0%** | **1.53 [0.50, 4.68]** |
| Total events | 61 | | 631 | | | |
| Heterogeneity: Chi² = 0.04, df = 2 (P = 0.98); I² = 0% | | | | | | |
| Test for overall effect: Z = 0.75 (P = 0.45) | | | | | | |
| **Total (95% CI)** | | **64** | | **685** | **100.0%** | **1.53 [0.50, 4.68]** |
| Total events | 61 | | 631 | | | |
| Heterogeneity: Chi² = 0.04, df = 2 (P = 0.98); I² = 0% | | | | | | |
| Test for overall effect: Z = 0.75 (P = 0.45) | | | | | | |
| Test for subgroup differences: Not applicable | | | | | | |

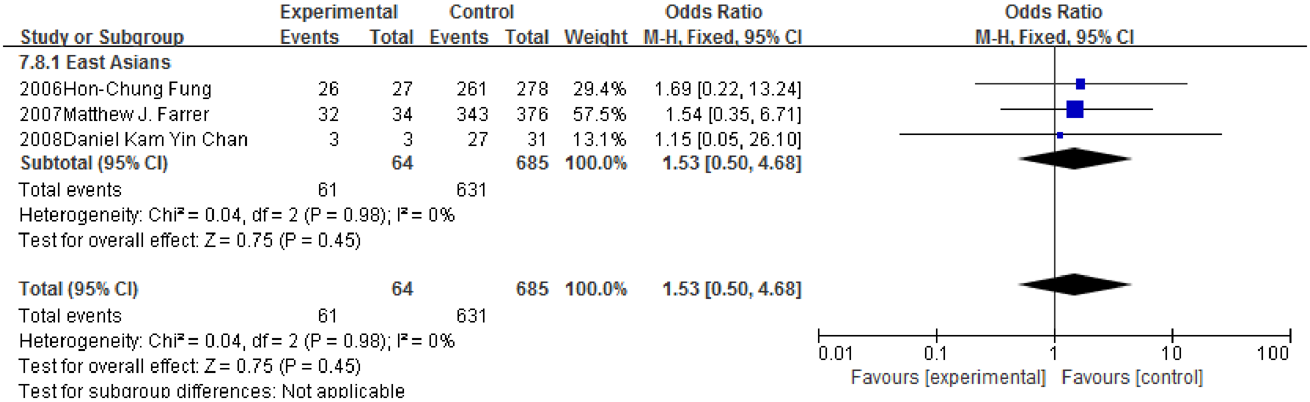


## 1.50 Rigidity of G2385R by ethnicity

| Study or Subgroup | Experimental Mean | Experimental SD | Experimental Total | Control Mean | Control SD | Control Total | Weight | Mean Difference IV, Fixed, 95% CI |
|---|---|---|---|---|---|---|---|---|
| **7.20.1 East Asians** | | | | | | | | |
| 2007Cao Li | 2.3 | 1.3 | 14 | 3.1 | 1.7 | 221 | 24.2% | -0.80 [-1.52, -0.08] |
| 2013Jiangping Cai | 2.57 | 2.45 | 49 | 2.96 | 2.26 | 461 | 24.2% | -0.39 [-1.11, 0.33] |
| 2016Sun, Qian | 1.92 | 1.98 | 76 | 1.67 | 1.58 | 225 | 51.6% | 0.25 [-0.24, 0.74] |
| **Subtotal (95% CI)** | | | **139** | | | **907** | **100.0%** | **-0.16 [-0.51, 0.19]** |
| Heterogeneity: Chi² = 6.14, df = 2 (P = 0.05); I² = 67% | | | | | | | | |
| Test for overall effect: Z = 0.88 (P = 0.38) | | | | | | | | |
| **Total (95% CI)** | | | **139** | | | **907** | **100.0%** | **-0.16 [-0.51, 0.19]** |
| Heterogeneity: Chi² = 6.14, df = 2 (P = 0.05); I² = 67% | | | | | | | | |
| Test for overall effect: Z = 0.88 (P = 0.38) | | | | | | | | |
| Test for subgroup differences: Not applicable | | | | | | | | |

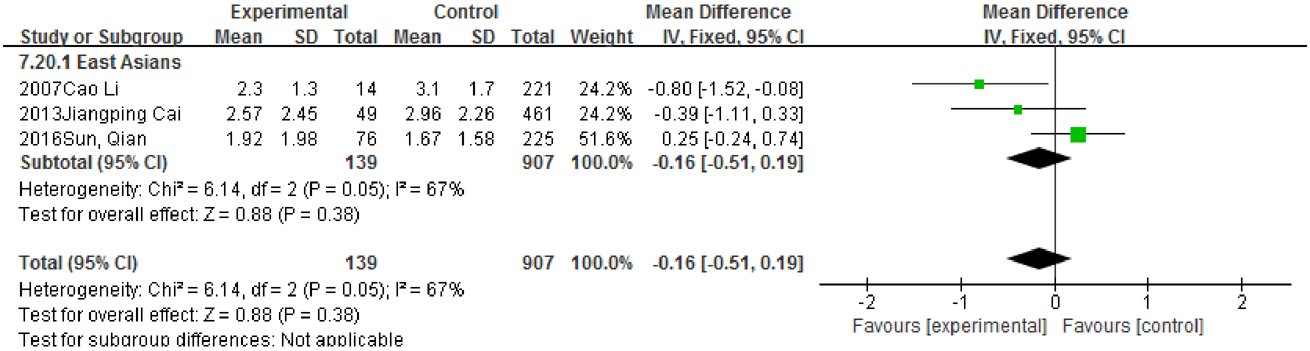


## 1.51 UPDRS Ⅰ of G2385R by ethnicity

| Study or Subgroup | Experimental Mean | Experimental SD | Experimental Total | Control Mean | Control SD | Control Total | Weight | Mean Difference IV, Random, 95% CI |
|---|---|---|---|---|---|---|---|---|
| **7.9.1 East Asians** | | | | | | | | |
| 2007Cao Li | 13.1 | 5.9 | 14 | 16.8 | 6.2 | 221 | 21.8% | -3.70 [-6.90, -0.50] |
| 2013Jiangping Cai | 16.8 | 8.8 | 49 | 17.2 | 8.26 | 461 | 23.7% | -0.40 [-2.98, 2.18] |
| 2016Sun, Qian | 11.16 | 5.71 | 76 | 11.32 | 4.35 | 225 | 26.5% | -0.16 [-1.56, 1.24] |
| **Subtotal (95% CI)** | | | **139** | | | **907** | **72.0%** | **-1.03 [-2.91, 0.85]** |
| Heterogeneity: Tau² = 1.40; Chi² = 4.00, df = 2 (P = 0.14); I² = 50% | | | | | | | | |
| Test for overall effect: Z = 1.08 (P = 0.28) | | | | | | | | |
| **7.9.2 Mixed** | | | | | | | | |
| 2016Marras, C. | 14.74 | 0.19 | 192 | 11.09 | 0.19 | 684 | 28.0% | 3.65 [3.62, 3.68] |
| **Subtotal (95% CI)** | | | **192** | | | **684** | **28.0%** | **3.65 [3.62, 3.68]** |
| Heterogeneity: Not applicable | | | | | | | | |
| Test for overall effect: Z = 235.22 (P < 0.00001) | | | | | | | | |
| **Total (95% CI)** | | | **331** | | | **1591** | **100.0%** | **0.08 [-3.12, 3.27]** |
| Heterogeneity: Tau² = 9.49; Chi² = 58.05, df = 3 (P < 0.00001); I² = 95% | | | | | | | | |
| Test for overall effect: Z = 0.05 (P = 0.96) | | | | | | | | |
| Test for subgroup differences: Chi² = 23.87, df = 1 (P < 0.00001), I² = 95.8% | | | | | | | | |

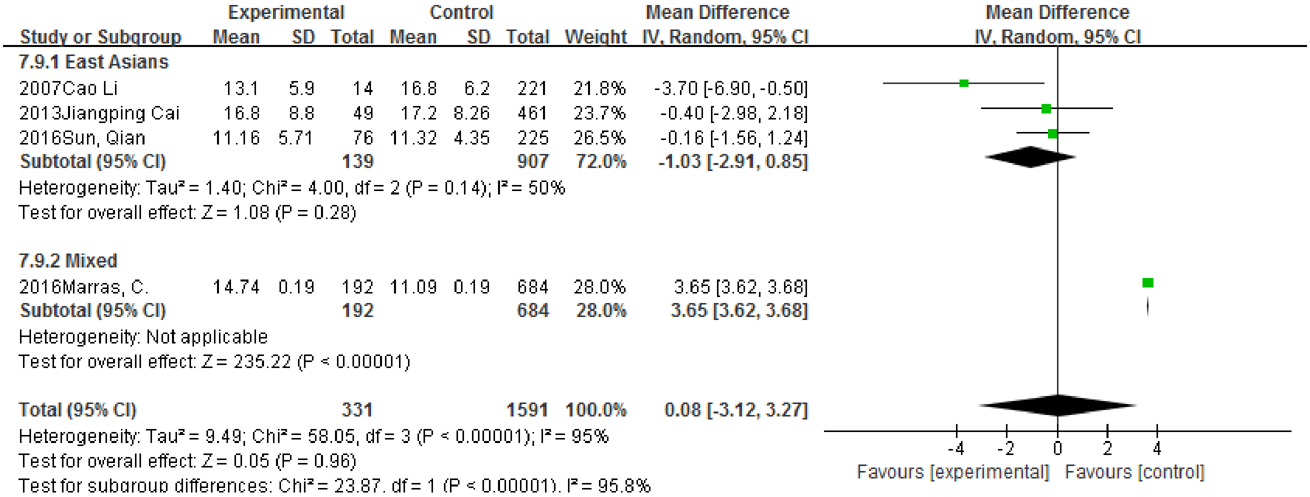


## 1.52 UPDRS Ⅱ of G2385R by ethnicity

| Study or Subgroup | Experimental Mean | Experimental SD | Experimental Total | Control Mean | Control SD | Control Total | Weight | Mean Difference IV, Random, 95% CI |
|---|---|---|---|---|---|---|---|---|
| **7.11.1 East Asians** | | | | | | | | |
| 2007Cao Li | 19.6 | 5.1 | 14 | 20.5 | 7.3 | 221 | 14.6% | -0.90 [-3.74, 1.94] |
| 2007Eng-King Tan | 26 | 11 | 33 | 27 | 11 | 29 | 12.9% | -1.00 [-6.49, 4.49] |
| 2007Matthew J. Farrer | 19.3 | 7.4 | 34 | 19.9 | 9.1 | 376 | 14.7% | -0.60 [-3.25, 2.05] |
| 2013Jiangping Cai | 21.4 | 8.78 | 49 | 22.1 | 8.35 | 461 | 14.7% | -0.70 [-3.27, 1.87] |
| 2016Sun, Qian | 24.55 | 12.08 | 76 | 26.52 | 12.64 | 225 | 14.4% | -1.97 [-5.15, 1.21] |
| 2017Hong, J. H. | 19.8 | 11.2 | 23 | 23.6 | 8.7 | 276 | 13.5% | -3.80 [-8.49, 0.89] |
| **Subtotal (95% CI)** | | | **229** | | | **1588** | **84.7%** | **-1.18 [-2.48, 0.11]** |
| Heterogeneity: Tau² = 0.00; Chi² = 1.79, df = 5 (P = 0.88); I² = 0% | | | | | | | | |
| Test for overall effect: Z = 1.79 (P = 0.07) | | | | | | | | |
| **7.11.2 Mixed** | | | | | | | | |
| 2016Marras, C. | 35.82 | 0.4 | 192 | 27.68 | 1.12 | 652 | 15.3% | 8.14 [8.04, 8.24] |
| **Subtotal (95% CI)** | | | **192** | | | **652** | **15.3%** | **8.14 [8.04, 8.24]** |
| Heterogeneity: Not applicable | | | | | | | | |
| Test for overall effect: Z = 155.02 (P < 0.00001) | | | | | | | | |
| **Total (95% CI)** | | | **421** | | | **2240** | **100.0%** | **-0.00 [-4.99, 4.99]** |
| Heterogeneity: Tau² = 42.42; Chi² = 199.14, df = 6 (P < 0.00001); I² = 97% | | | | | | | | |
| Test for overall effect: Z = 0.00 (P = 1.00) | | | | | | | | |
| Test for subgroup differences: Chi² = 197.34, df = 1 (P < 0.00001), I² = 99.5% | | | | | | | | |

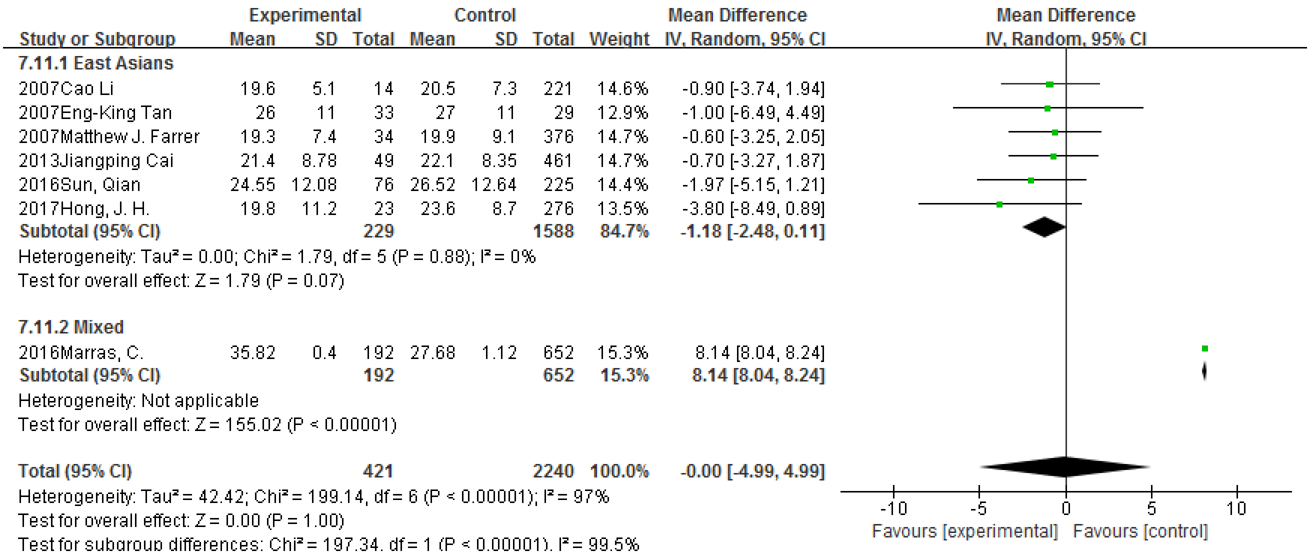


## 1.53 UPDRSⅢ of G2385R by ethnicity

| Study or Subgroup | Experimental Mean | Experimental SD | Experimental Total | Control Mean | Control SD | Control Total | Weight | Mean Difference IV, Fixed, 95% CI |
|---|---|---|---|---|---|---|---|---|
| **7.13.1 East Asians** | | | | | | | | |
| 2007Cao Li | 1.7 | 0.4 | 14 | 2 | 0.8 | 221 | 22.0% | -0.30 [-0.53, -0.07] |
| 2007Matthew J. Farrer | 2.5 | 1.2 | 34 | 2.6 | 0.9 | 376 | 7.1% | -0.10 [-0.51, 0.31] |
| 2008X.-K. An | 2.16 | 0.91 | 71 | 2.33 | 0.98 | 529 | 23.3% | -0.17 [-0.40, 0.06] |
| 2013Jiangping Cai | 2.47 | 0.89 | 49 | 2.57 | 0.93 | 461 | 17.4% | -0.10 [-0.36, 0.16] |
| 2016Cao, M. | 2.2 | 0.8 | 25 | 2.4 | 0.74 | 43 | 8.2% | -0.20 [-0.58, 0.18] |
| 2016Sun, Qian | 2.15 | 0.93 | 76 | 2.08 | 0.81 | 225 | 22.0% | 0.07 [-0.16, 0.30] |
| **Subtotal (95% CI)** | | | **269** | | | **1855** | **100.0%** | **-0.13 [-0.24, -0.02]** |
| Heterogeneity: Chi² = 5.13, df = 5 (P = 0.40); I² = 3% | | | | | | | | |
| Test for overall effect: Z = 2.34 (P = 0.02) | | | | | | | | |
| **Total (95% CI)** | | | **269** | | | **1855** | **100.0%** | **-0.13 [-0.24, -0.02]** |
| Heterogeneity: Chi² = 5.13, df = 5 (P = 0.40); I² = 3% | | | | | | | | |
| Test for overall effect: Z = 2.34 (P = 0.02) | | | | | | | | |
| Test for subgroup differences: Not applicable | | | | | | | | |

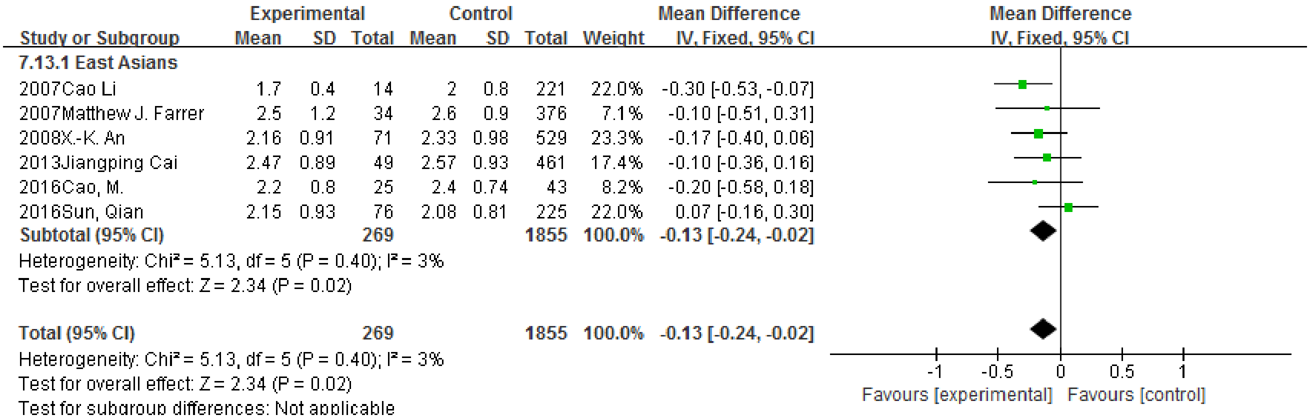


## 1.54 H-Y of G2385R by ethnicity

| Study or Subgroup | Experimental Events | Experimental Total | Control Events | Control Total | Weight | Odds Ratio M-H, Fixed, 95% CI |
|---|---|---|---|---|---|---|
| **7.14.1 East Asians** | | | | | | |
| 2007Cao Li | 3 | 14 | 57 | 221 | 8.3% | 0.78 [0.21, 2.91] |
| 2007Eng-King Tan | 7 | 33 | 11 | 29 | 14.4% | 0.44 [0.14, 1.35] |
| 2007Matthew J. Farrer | 13 | 34 | 117 | 376 | 18.7% | 1.37 [0.66, 2.83] |
| 2016Sun, Qian | 4 | 76 | 8 | 225 | 6.0% | 1.51 [0.44, 5.15] |
| **Subtotal (95% CI)** | | **157** | | **851** | **47.5%** | **1.00 [0.61, 1.66]** |
| Total events | 27 | | 193 | | | |
| Heterogeneity: Chi² = 3.33, df = 3 (P = 0.34); I² = 10% | | | | | | |
| Test for overall effect: Z = 0.01 (P = 0.99) | | | | | | |
| **7.14.2 Mixed** | | | | | | |
| 2016Marras, C. | 16 | 146 | 73 | 419 | 52.5% | 0.58 [0.33, 1.04] |
| **Subtotal (95% CI)** | | **146** | | **419** | **52.5%** | **0.58 [0.33, 1.04]** |
| Total events | 16 | | 73 | | | |
| Heterogeneity: Not applicable | | | | | | |
| Test for overall effect: Z = 1.83 (P = 0.07) | | | | | | |
| **Total (95% CI)** | | **303** | | **1270** | **100.0%** | **0.78 [0.54, 1.14]** |
| Total events | 43 | | 266 | | | |
| Heterogeneity: Chi² = 5.39, df = 4 (P = 0.25); I² = 26% | | | | | | |
| Test for overall effect: Z = 1.28 (P = 0.20) | | | | | | |
| Test for subgroup differences: Chi² = 1.92, df = 1 (P = 0.17), I² = 47.9% | | | | | | |

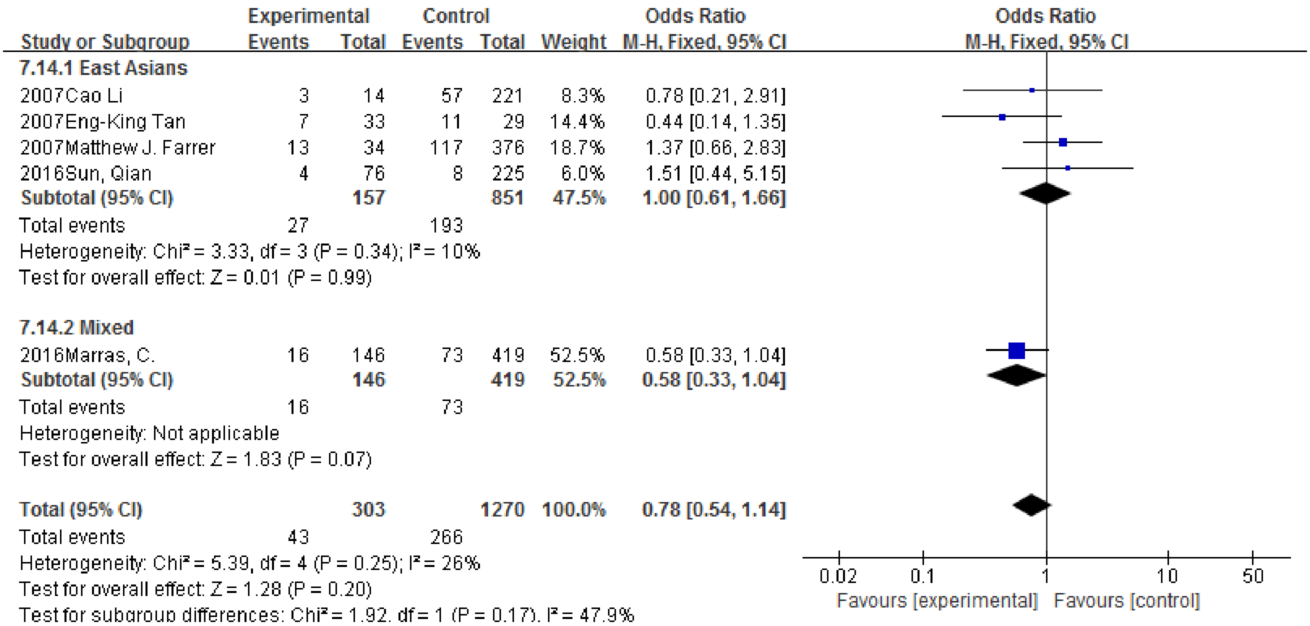


## 1.55 Dyskinesia of G2385R by ethnicity

| Study or Subgroup | Experimental Events | Experimental Total | Control Events | Control Total | Weight | Odds Ratio M-H, Fixed, 95% CI |
|---|---|---|---|---|---|---|
| **7.15.1 East Asians** | | | | | | |
| 2007Matthew J. Farrer | 12 | 34 | 122 | 376 | 33.7% | 1.14 [0.54, 2.37] |
| 2008Daniel Kam Yin Chan | 0 | 3 | 3 | 31 | 1.7% | 1.16 [0.05, 27.54] |
| 2016Sun, Qian | 27 | 27 | 50 | 225 | 0.5% | 191.14 [11.46, 3188.64] |
| **Subtotal (95% CI)** | | **64** | | **632** | **35.9%** | **3.84 [2.30, 6.41]** |
| Total events | 39 | | 175 | | | |
| Heterogeneity: Chi² = 18.49, df = 2 (P < 0.0001); I² = 89% | | | | | | |
| Test for overall effect: Z = 5.15 (P < 0.00001) | | | | | | |
| **7.15.2 Mixed** | | | | | | |
| 2016Marras, C. | 61 | 130 | 95 | 396 | 64.1% | 2.80 [1.85, 4.24] |
| **Subtotal (95% CI)** | | **130** | | **396** | **64.1%** | **2.80 [1.85, 4.24]** |
| Total events | 61 | | 95 | | | |
| Heterogeneity: Not applicable | | | | | | |
| Test for overall effect: Z = 4.87 (P < 0.00001) | | | | | | |
| **Total (95% CI)** | | **194** | | **1028** | **100.0%** | **3.17 [2.30, 4.38]** |
| Total events | 100 | | 270 | | | |
| Heterogeneity: Chi² = 16.38, df = 3 (P = 0.0009); I² = 82% | | | | | | |
| Test for overall effect: Z = 7.05 (P < 0.00001) | | | | | | |
| Test for subgroup differences: Chi² = 0.88, df = 1 (P = 0.35), I² = 0% | | | | | | |

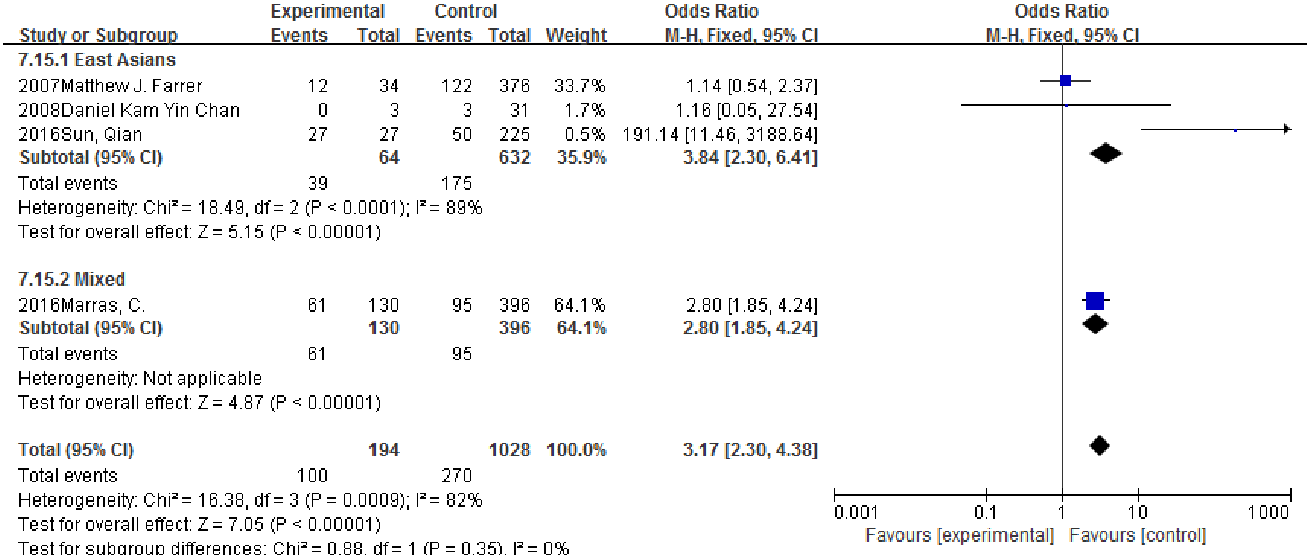


## 1.56 Motor fluctuations of G2385R by ethnicity

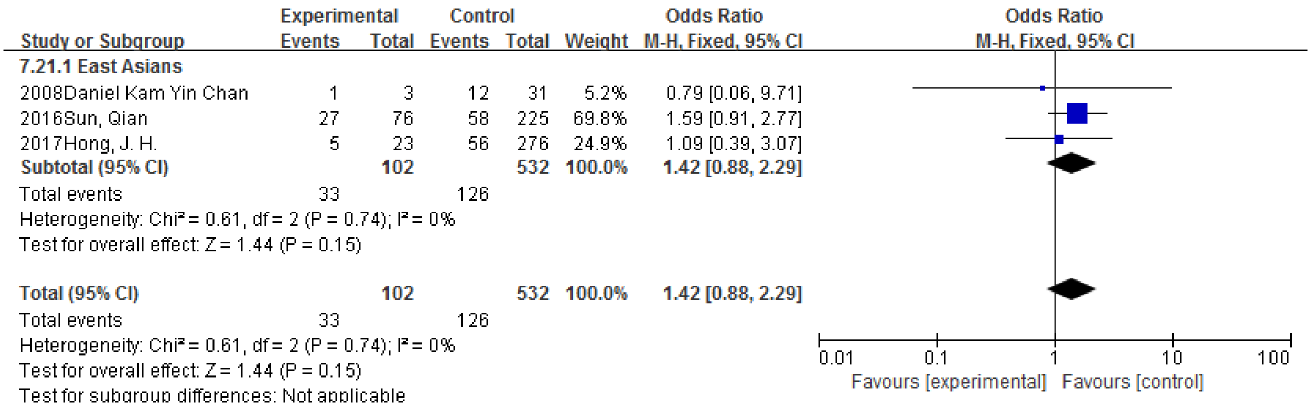


## 1.57 Depression of G2385R by ethnicity

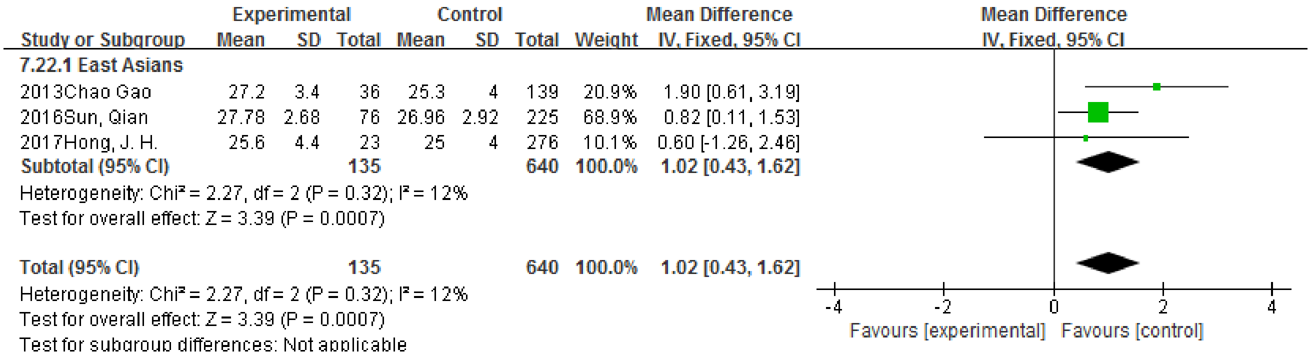


## 1.58 MMSE of G2385R by ethnicity

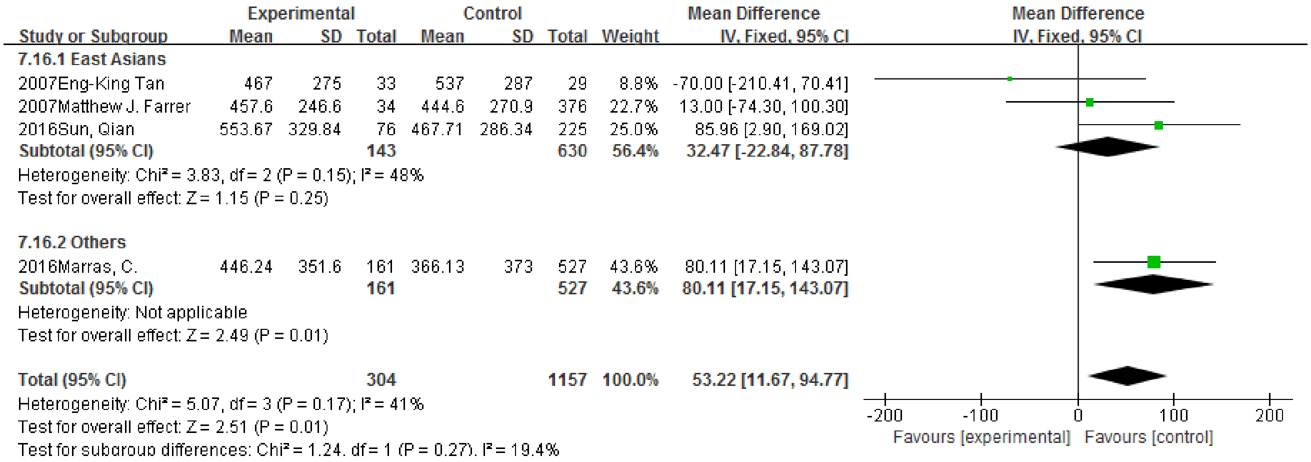


## 1.59 LEDD of G2385R by ethnicity

| Study or Subgroup | Experimental Mean | SD | Total | Control Mean | SD | Total | Weight | Mean Difference IV, Random, 95% CI |
|---|---|---|---|---|---|---|---|---|
| **11.1.1 East Asians** | | | | | | | | |
| 2009Lihua Yu | 53.53 | 10.35 | 328 | 52.6 | 11.55 | 311 | 19.4% | 0.93 [-0.77, 2.63] |
| 2009Zijuan Zhang | 57.33 | 9.13 | 600 | 55.79 | 11.3 | 557 | 20.7% | 1.54 [0.35, 2.73] |
| 2013Jiangping Cai | 55.6 | 10.7 | 510 | 58.6 | 10.9 | 481 | 20.3% | -3.00 [-4.35, -1.65] |
| 2013Xiaoli Fu | 54 | 11.7 | 446 | 55 | 11 | 399 | 19.9% | -1.00 [-2.53, 0.53] |
| 2014Pulkes, T. | 56 | 13 | 485 | 60.1 | 12.2 | 431 | 19.6% | -4.10 [-5.73, -2.47] |
| **Subtotal (95% CI)** | | | **2369** | | | **2179** | **100.0%** | **-1.11 [-3.30, 1.07]** |
| Heterogeneity: Tau² = 5.62; Chi² = 44.73, df = 4 (P < 0.00001); I² = 91% | | | | | | | | |
| Test for overall effect: Z = 1.00 (P = 0.32) | | | | | | | | |
| **Total (95% CI)** | | | **2369** | | | **2179** | **100.0%** | **-1.11 [-3.30, 1.07]** |
| Heterogeneity: Tau² = 5.62; Chi² = 44.73, df = 4 (P < 0.00001); I² = 91% | | | | | | | | |
| Test for overall effect: Z = 1.00 (P = 0.32) | | | | | | | | |
| Test for subgroup differences: Not applicable | | | | | | | | |

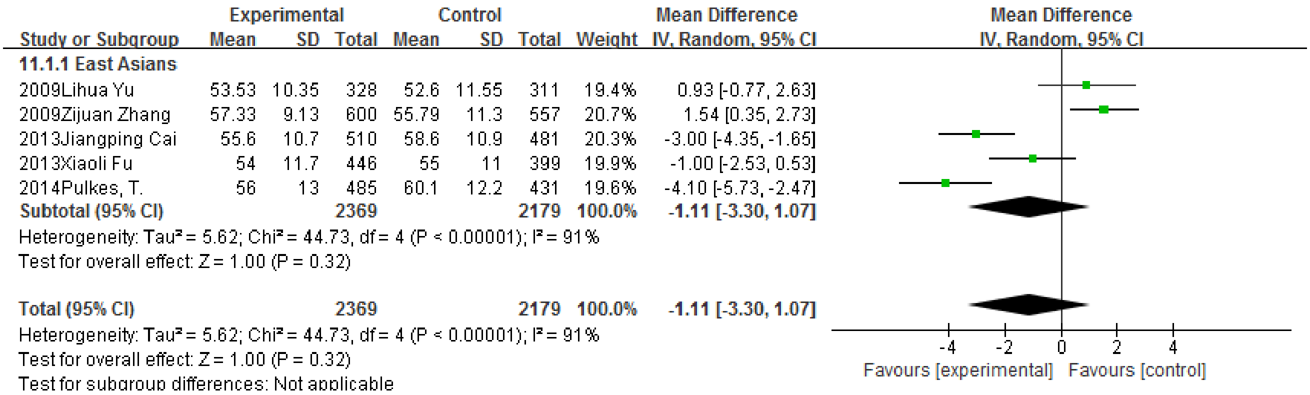


## 1.60 Age at onset of R1628P by ethnicity

| Study or Subgroup | Experimental Events | Total | Control Events | Total | Weight | Odds Ratio M-H, Random, 95% CI |
|---|---|---|---|---|---|---|
| **11.2.1 East Asians** | | | | | | |
| 2009Lihua Yu | 6 | 17 | 132 | 311 | 21.1% | 0.74 [0.27, 2.05] |
| 2009Zijuan Zhang | 5 | 43 | 145 | 557 | 22.6% | 0.37 [0.14, 0.97] |
| 2013Jiangping Cai | 10 | 29 | 106 | 481 | 26.3% | 1.86 [0.84, 4.13] |
| 2013Xiaoli Fu | 15 | 47 | 109 | 399 | 30.1% | 1.25 [0.65, 2.39] |
| **Subtotal (95% CI)** | | **136** | | **1748** | **100.0%** | **0.95 [0.49, 1.83]** |
| Total events | 36 | | 492 | | | |
| Heterogeneity: Tau² = 0.27; Chi² = 7.39, df = 3 (P = 0.06); I² = 59% | | | | | | |
| Test for overall effect: Z = 0.17 (P = 0.87) | | | | | | |
| **Total (95% CI)** | | **136** | | **1748** | **100.0%** | **0.95 [0.49, 1.83]** |
| Total events | 36 | | 492 | | | |
| Heterogeneity: Tau² = 0.27; Chi² = 7.39, df = 3 (P = 0.06); I² = 59% | | | | | | |
| Test for overall effect: Z = 0.17 (P = 0.87) | | | | | | |
| Test for subgroup differences: Not applicable | | | | | | |

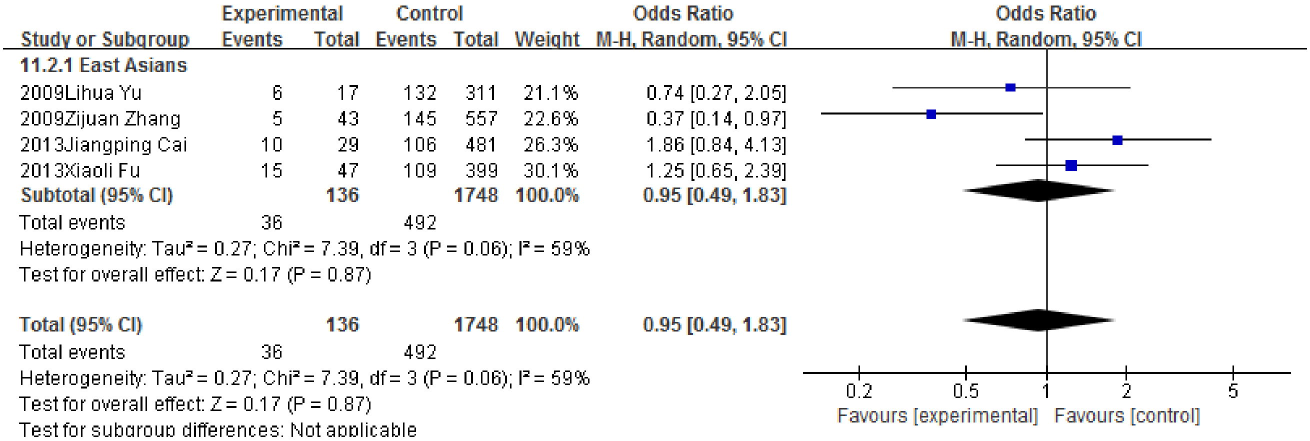


## 1.61 EOPD of R1628P by ethnicity

| Study or Subgroup | Experimental Events | Total | Control Events | Total | Weight | Odds Ratio M-H, Fixed, 95% CI |
|---|---|---|---|---|---|---|
| **11.3.1 East Asians** | | | | | | |
| 2009Lihua Yu | 9 | 17 | 169 | 311 | 24.9% | 0.95 [0.36, 2.51] |
| 2013Jiangping Cai | 18 | 29 | 285 | 481 | 37.1% | 1.13 [0.52, 2.43] |
| 2013Xiaoli Fu | 33 | 47 | 201 | 399 | 38.1% | 2.32 [1.21, 4.47] |
| **Subtotal (95% CI)** | | **93** | | **1191** | **100.0%** | **1.54 [0.99, 2.38]** |
| Total events | 60 | | 655 | | | |
| Heterogeneity: Chi² = 3.10, df = 2 (P = 0.21); I² = 35% | | | | | | |
| Test for overall effect: Z = 1.91 (P = 0.06) | | | | | | |
| **Total (95% CI)** | | **93** | | **1191** | **100.0%** | **1.54 [0.99, 2.38]** |
| Total events | 60 | | 655 | | | |
| Heterogeneity: Chi² = 3.10, df = 2 (P = 0.21); I² = 35% | | | | | | |
| Test for overall effect: Z = 1.91 (P = 0.06) | | | | | | |
| Test for subgroup differences: Not applicable | | | | | | |

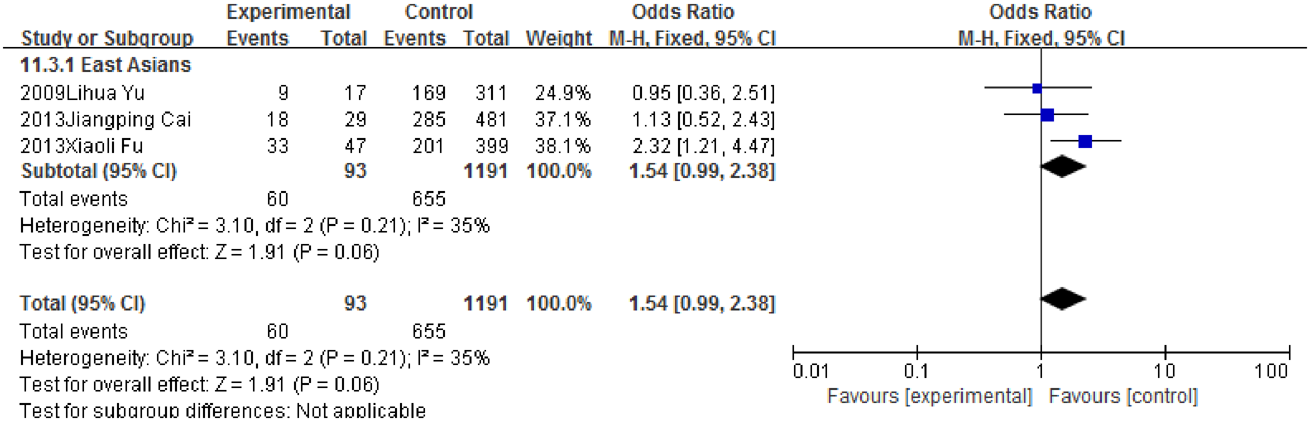


## 1.62 Gender-male of R1628P by ethnicity

| Study or Subgroup | Experimental Events | Experimental Total | Control Events | Control Total | Weight | Odds Ratio M-H, Fixed, 95% CI |
|---|---|---|---|---|---|---|
| **11.4.1 East Asians** | | | | | | |
| 2009Lihua Yu | 9 | 17 | 162 | 311 | 11.9% | 1.03 [0.39, 2.75] |
| 2009Zijuan Zhang | 18 | 43 | 282 | 557 | 35.4% | 0.70 [0.37, 1.32] |
| 2013Jiangping Cai | 19 | 29 | 314 | 481 | 18.5% | 1.01 [0.46, 2.22] |
| 2013Xiaoli Fu | 24 | 47 | 220 | 399 | 34.2% | 0.85 [0.46, 1.55] |
| **Subtotal (95% CI)** | | **136** | | **1748** | **100.0%** | **0.85 [0.60, 1.21]** |
| Total events | 70 | | 978 | | | |
| Heterogeneity: Chi² = 0.70, df = 3 (P = 0.87); I² = 0% | | | | | | |
| Test for overall effect: Z = 0.91 (P = 0.36) | | | | | | |
| **Total (95% CI)** | | **136** | | **1748** | **100.0%** | **0.85 [0.60, 1.21]** |
| Total events | 70 | | 978 | | | |
| Heterogeneity: Chi² = 0.70, df = 3 (P = 0.87); I² = 0% | | | | | | |
| Test for overall effect: Z = 0.91 (P = 0.36) | | | | | | |
| Test for subgroup differences: Not applicable | | | | | | |

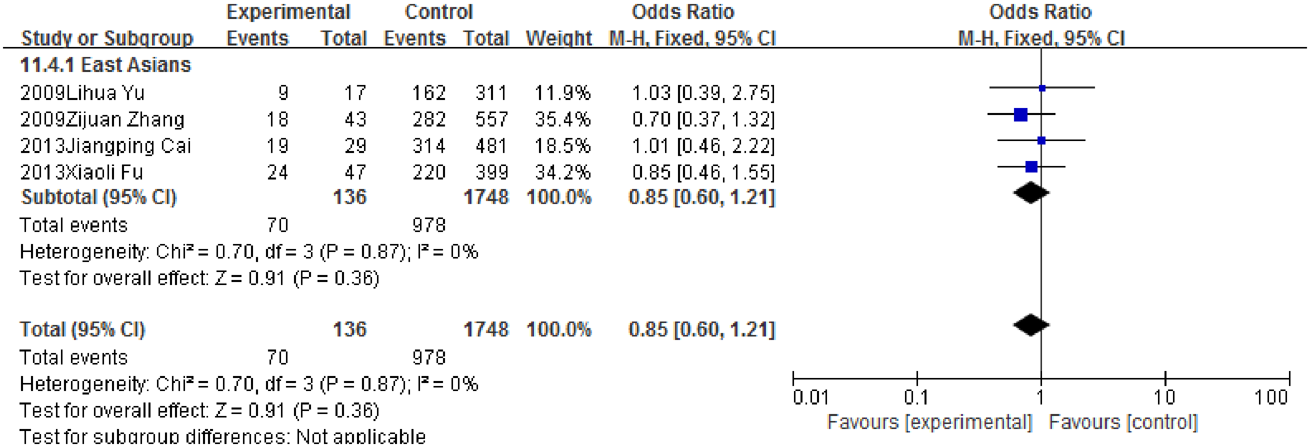


## 1.63 FS-Resting tremor of R1628P by ethnicity

| Study or Subgroup | Experimental Mean | Experimental SD | Experimental Total | Control Mean | Control SD | Control Total | Weight | Mean Difference IV, Random, 95% CI |
|---|---|---|---|---|---|---|---|---|
| **11.5.1 East Asians** | | | | | | | | |
| 2009Lihua Yu | 2.09 | 0.94 | 17 | 2.21 | 0.78 | 311 | 21.4% | -0.12 [-0.58, 0.34] |
| 2009Zijuan Zhang | 2.21 | 1.02 | 43 | 2.32 | 0.96 | 557 | 25.8% | -0.11 [-0.43, 0.21] |
| 2013Jiangping Cai | 2.66 | 1.11 | 29 | 2.55 | 0.91 | 481 | 22.8% | 0.11 [-0.30, 0.52] |
| 2014Pulkes, T. | 3 | 0.5 | 54 | 2.5 | 0.8 | 431 | 30.0% | 0.50 [0.35, 0.65] |
| **Subtotal (95% CI)** | | | **143** | | | **1780** | **100.0%** | **0.12 [-0.25, 0.49]** |
| Heterogeneity: Tau² = 0.11; Chi² = 17.04, df = 3 (P = 0.0007); I² = 82% | | | | | | | | |
| Test for overall effect: Z = 0.64 (P = 0.52) | | | | | | | | |
| **Total (95% CI)** | | | **143** | | | **1780** | **100.0%** | **0.12 [-0.25, 0.49]** |
| Heterogeneity: Tau² = 0.11; Chi² = 17.04, df = 3 (P = 0.0007); I² = 82% | | | | | | | | |
| Test for overall effect: Z = 0.64 (P = 0.52) | | | | | | | | |
| Test for subgroup differences: Not applicable | | | | | | | | |

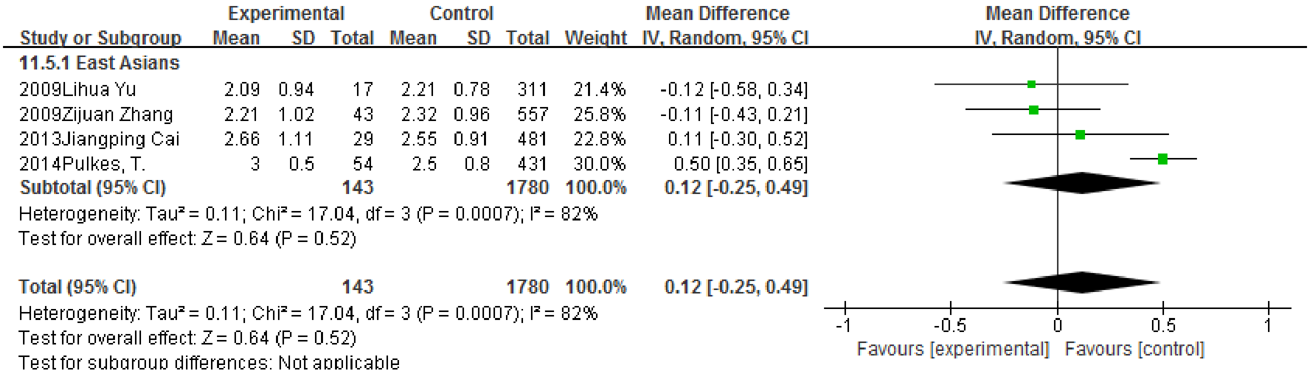


## 1.64 H-Y of R1628P by ethnicity

| Study or Subgroup | Experimental Events | Experimental Total | Control Events | Control Total | Weight | Odds Ratio M-H, Fixed, 95% CI |
|---|---|---|---|---|---|---|
| **9.1.1 European/West Asians** | | | | | | |
| 2014A. Estanga | 14 | 30 | 13 | 30 | 39.0% | 1.14 [0.41, 3.17] |
| 2015Somme, J. H. | 8 | 15 | 18 | 27 | 33.8% | 0.57 [0.16, 2.08] |
| **Subtotal (95% CI)** | | **45** | | **57** | **72.8%** | **0.88 [0.40, 1.95]** |
| Total events | 22 | | 31 | | | |
| Heterogeneity: Chi² = 0.68, df = 1 (P = 0.41); I² = 0% | | | | | | |
| Test for overall effect: Z = 0.32 (P = 0.75) | | | | | | |
| **9.1.2 Hispanics** | | | | | | |
| 2009Mata, I. F.(2) | 0 | 1 | 58 | 119 | 8.1% | 0.35 [0.01, 8.78] |
| **Subtotal (95% CI)** | | **1** | | **119** | **8.1%** | **0.35 [0.01, 8.78]** |
| Total events | 0 | | 58 | | | |
| Heterogeneity: Not applicable | | | | | | |
| Test for overall effect: Z = 0.64 (P = 0.52) | | | | | | |
| **9.1.3 Mixed** | | | | | | |
| 2008Latourelle, J. C. | 1 | 4 | 549 | 965 | 19.1% | 0.25 [0.03, 2.44] |
| **Subtotal (95% CI)** | | **4** | | **965** | **19.1%** | **0.25 [0.03, 2.44]** |
| Total events | 1 | | 549 | | | |
| Heterogeneity: Not applicable | | | | | | |
| Test for overall effect: Z = 1.19 (P = 0.23) | | | | | | |
| **Total (95% CI)** | | **50** | | **1141** | **100.0%** | **0.72 [0.35, 1.47]** |
| Total events | 23 | | 638 | | | |
| Heterogeneity: Chi² = 1.93, df = 3 (P = 0.59); I² = 0% | | | | | | |
| Test for overall effect: Z = 0.91 (P = 0.36) | | | | | | |
| Test for subgroup differences: Chi² = 1.25, df = 2 (P = 0.54), I² = 0% | | | | | | |

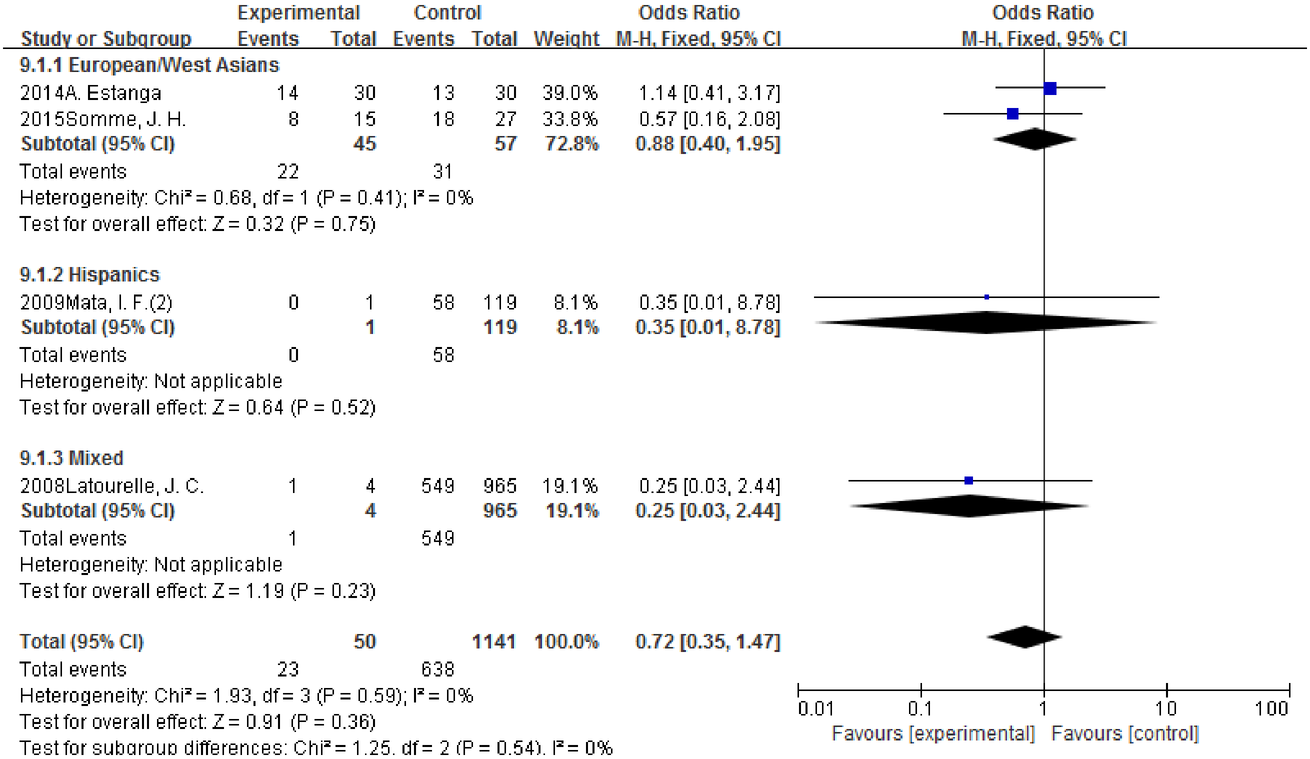


1.65 Gender-male of R1441G by ethnicity

| Study or Subgroup | Experimental Mean | Experimental SD | Experimental Total | Control Mean | Control SD | Control Total | Weight | Mean Difference IV, Fixed, 95% CI |
|---|---|---|---|---|---|---|---|---|
| **9.2.1 European/West Asians** | | | | | | | | |
| 2013Beatriz Tijero | 28.17 | 8.56 | 6 | 26.54 | 7.48 | 13 | 19.4% | 1.63 [-6.34, 9.60] |
| 2014A. Estanga | 18.86 | 10.61 | 30 | 21.17 | 12.56 | 30 | 35.6% | -2.31 [-8.19, 3.57] |
| 2015Somme, J. H. | 28.5 | 8.4 | 15 | 31.3 | 8.1 | 27 | 45.0% | -2.80 [-8.03, 2.43] |
| **Subtotal (95% CI)** | | | **51** | | | **70** | **100.0%** | **-1.77 [-5.28, 1.75]** |
| Heterogeneity: Chi² = 0.88, df = 2 (P = 0.64); I² = 0% | | | | | | | | |
| Test for overall effect: Z = 0.99 (P = 0.32) | | | | | | | | |
| **Total (95% CI)** | | | **51** | | | **70** | **100.0%** | **-1.77 [-5.28, 1.75]** |
| Heterogeneity: Chi² = 0.88, df = 2 (P = 0.64); I² = 0% | | | | | | | | |
| Test for overall effect: Z = 0.99 (P = 0.32) | | | | | | | | |
| Test for subgroup differences: Not applicable | | | | | | | | |

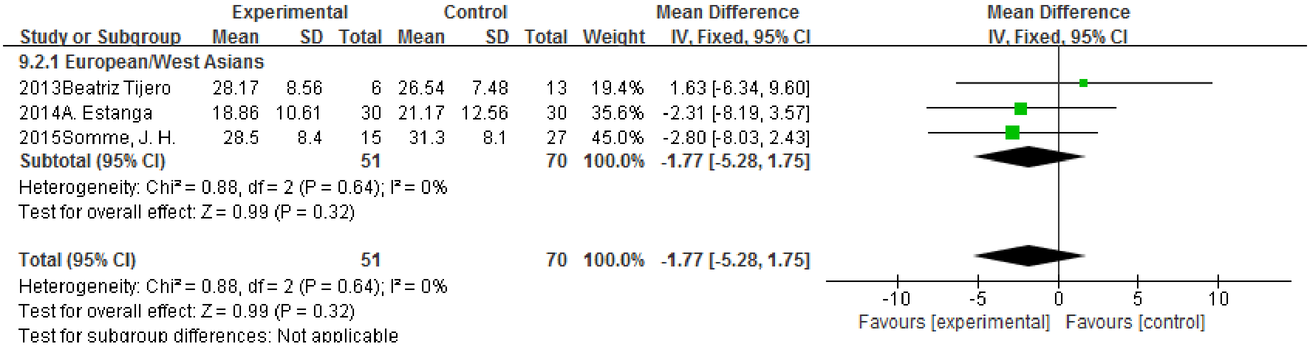


1.66 UPDRSⅢ of R1441G by ethnicity

Supplementary Figure 3. Forest plots of the association between specific variants in *LRRK2* and PD clinical features in different ethnic groups. 1.1-1.39 reflected the pooled results of G2019S-related clinical features in different ethnic groups.1.40-1.59 reflected the pooled results of G2385R-related clinical features in different ethnic groups. 1.60-1.64 represented the pooled results of R1628P-related clinical features in different ethnic groups. 1.65-1.66 represented the pooled results of R1441G-related clinical features in different ethnic groups.
