# Supplementary material for: Clinical Heterogeneity Among LRRK2 Variants in Parkinson's Disease: A Meta-Analysis
Source: Front Aging Neurosci. 2018 Sep 19;10:283. doi: 10.3389/fnagi.2018.00283 (PMC6156433; doi:10.3389/fnagi.2018.00283)
Supplement: Supplementary file 8 [file Image_4.pdf]

Supplementary Material

Clinical heterogeneity among *LRRK2* variants in Parkinson's disease: a meta-analysis

Li Shu[2 †], Yuan Zhang[2 †], Hongxu Pan[2], Qian Xu[2, 3,4], Jifeng Guo[2,3,4,6,7,8], Beisha Tang[1, 2,3,4,5,6,7,8], Qiying Sun[1,3,4*]

[†] These authors have contributed equally to this work and are co-first authors.

[*] **Correspondence**: Qiying Sun sunqiying2015@163.com

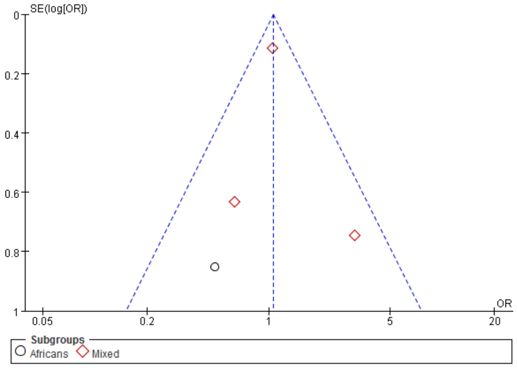

2.1 Asymmetrical onset of G2019S

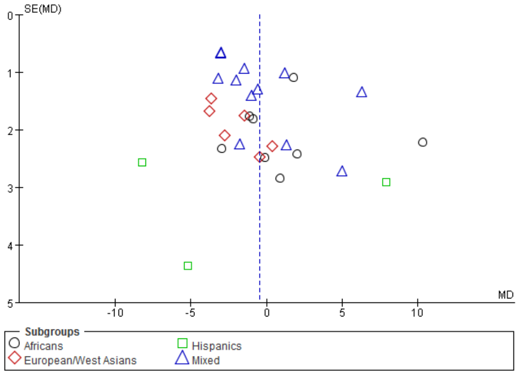

2.2 Age at onset of G2019S

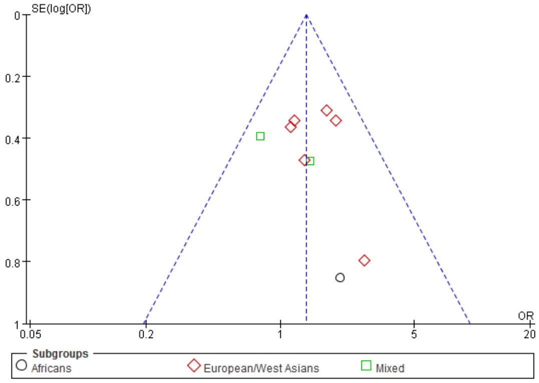

2.3 EOPD of G2019S

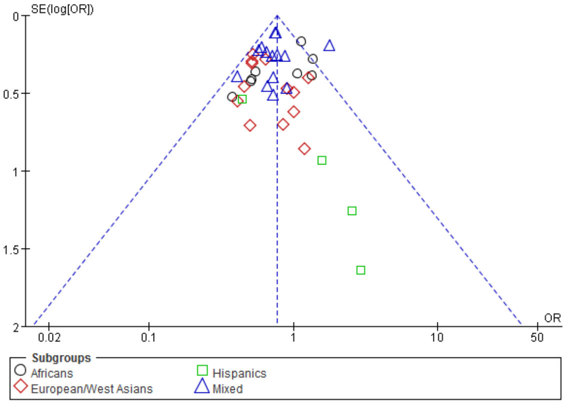

2.4 Gender-male of G2019S

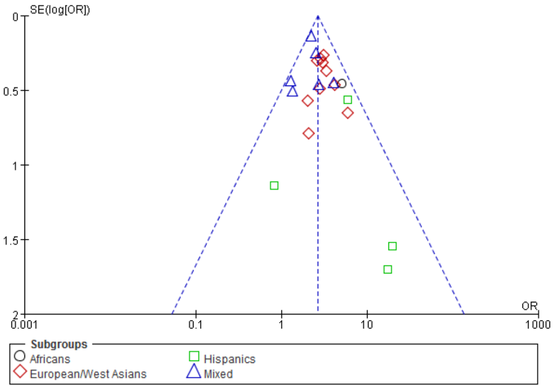

2.5 Family history of G2019S

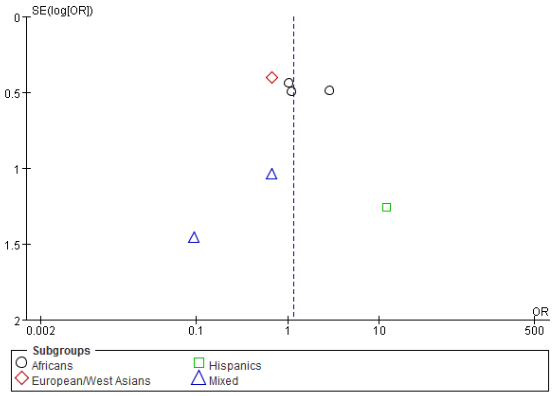

2.6 FS-bradykinesia of G2019S

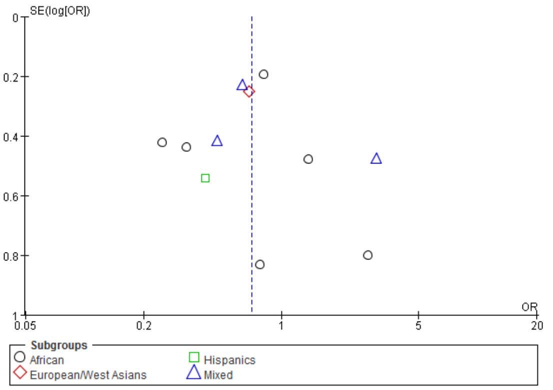

2.7 FS-resting tremor of G2019S

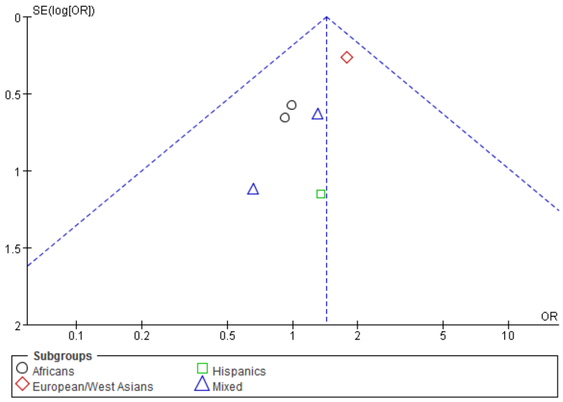

2.8 FS-rigidity of G2019S

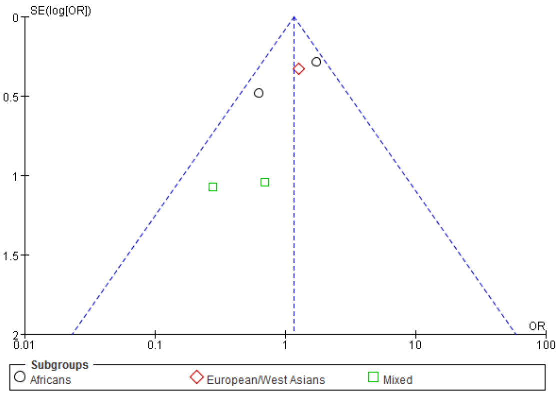

2.9 FS-Postural instability or Gait difficulty of G2019S

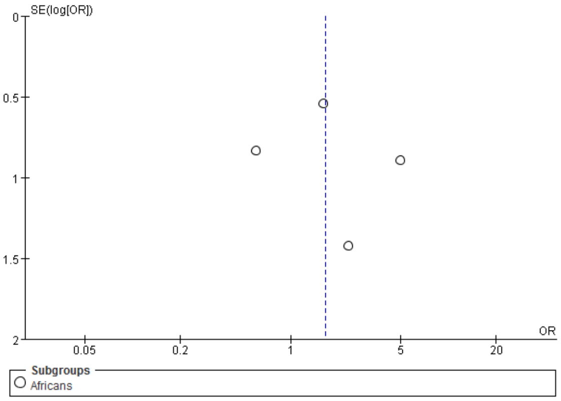

2.10 FS-Dystonia of G2019S

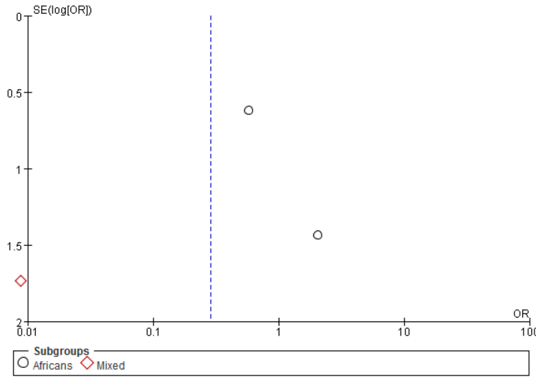

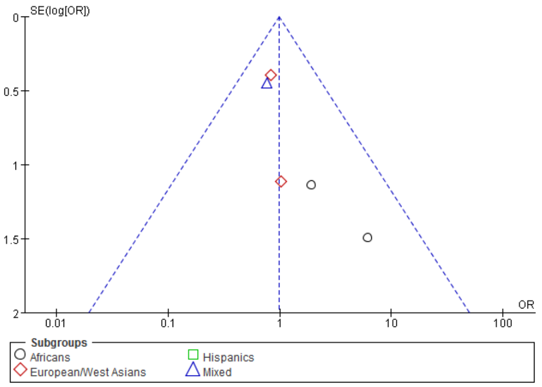

2.11 FS-Micrographia of G2019S

2.12 Bradykinesia of G2019S

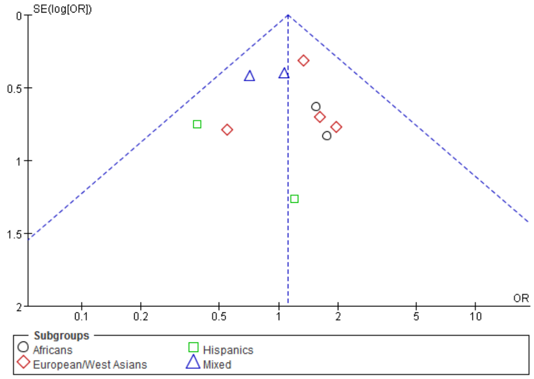

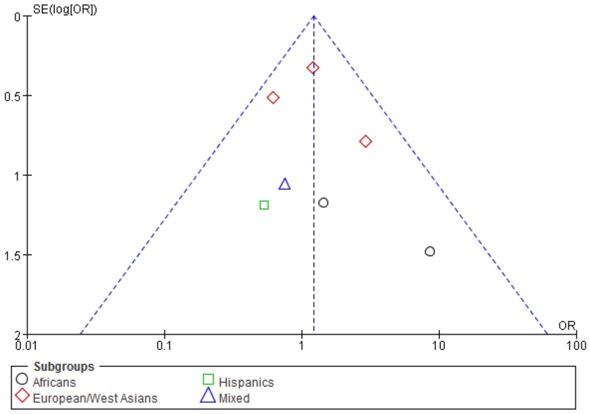

2.13 Resting tremor of G2019S

2.14 Rigidity of G2019S

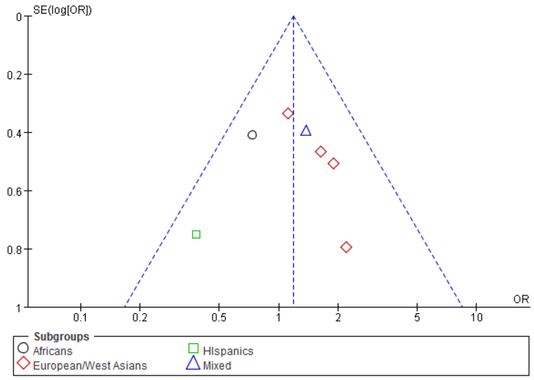

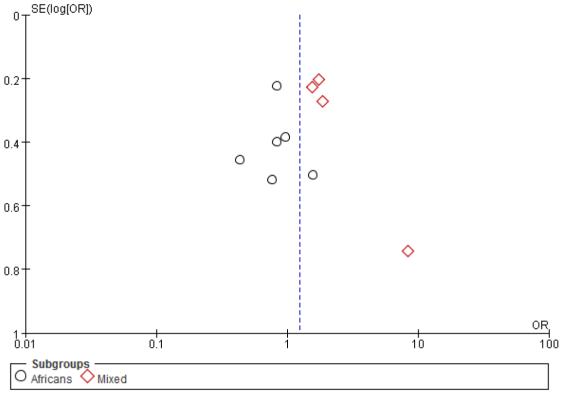

2.15 Postural instability/Gait difficulty of G2019S 2.16T-Akinetic-rigid/PIGD of G2019S

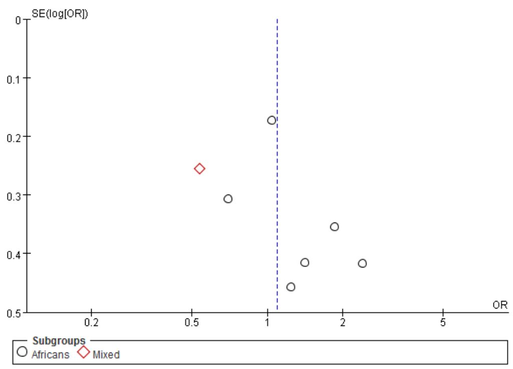

2.17 T-Mixed/Intermediate of G2019S

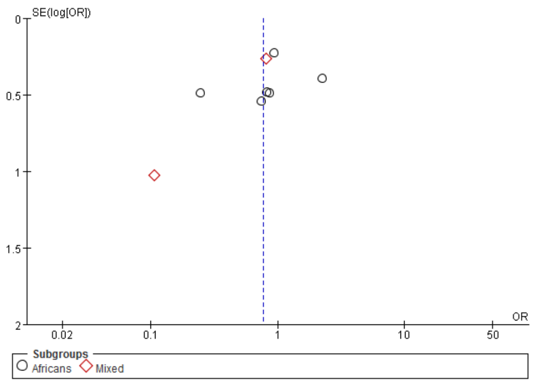

2.18 T-Tremor-dominant of G2019S

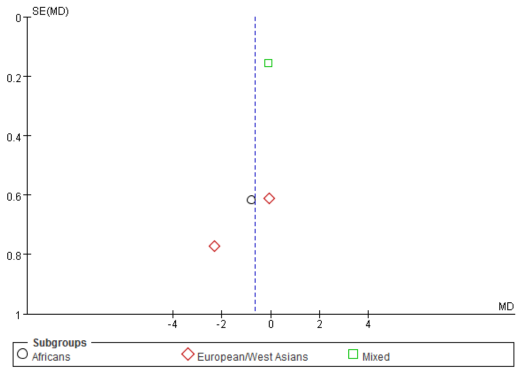

2.19 UPDRS Ⅰ of G2019S

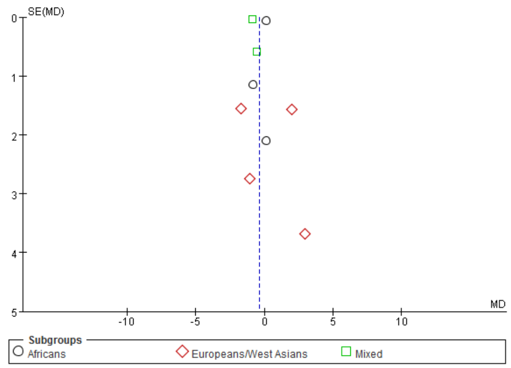

2.20 UPDRS Ⅱ of G2019S

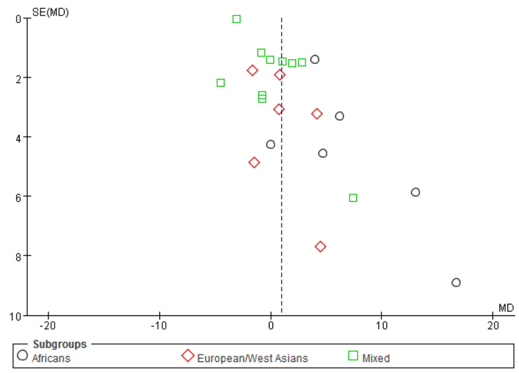

2.21 UPDRSⅢ of G2019S

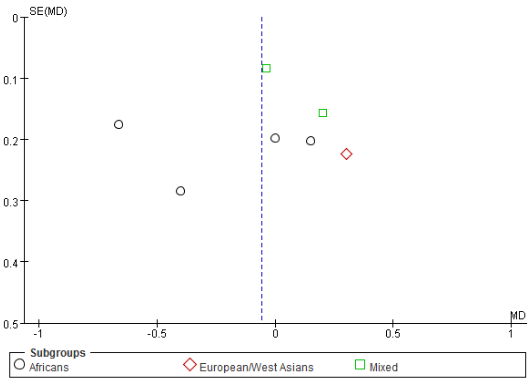

2.22 H-Y of G2019S

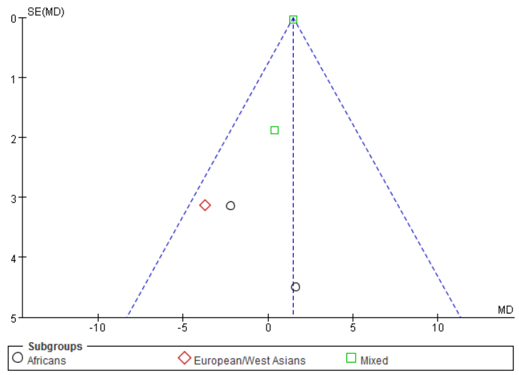

2.23 Schwab & England of G2019S

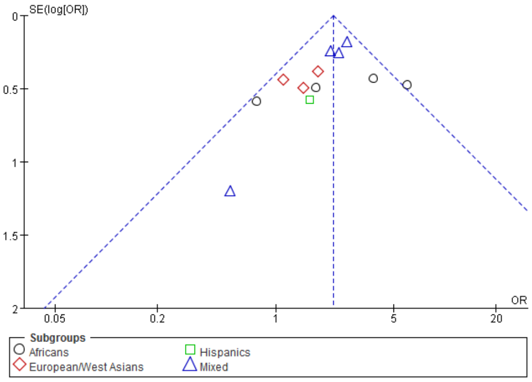

2.24 Dyskinesia of G2019S

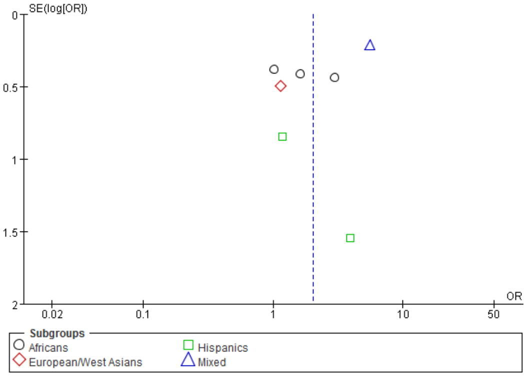

2.25 Motor fluctuations of G2019S

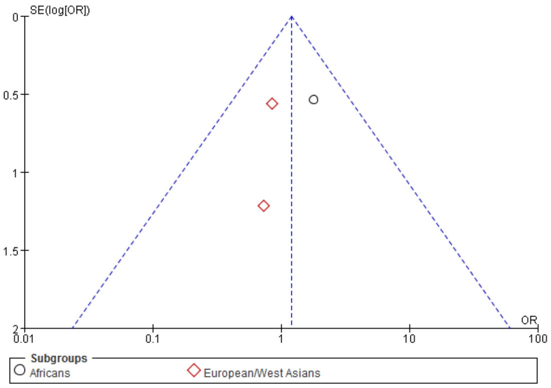

2.26 Anxiety of G2019S

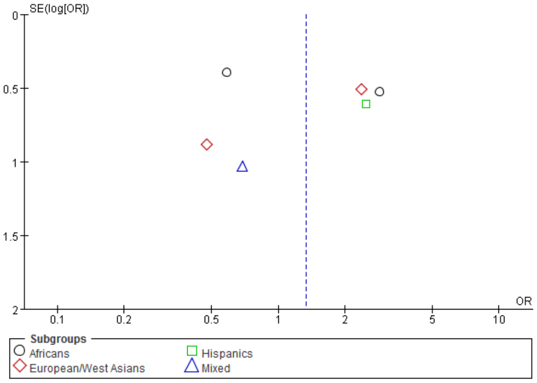

2.27 Depression of G2019S

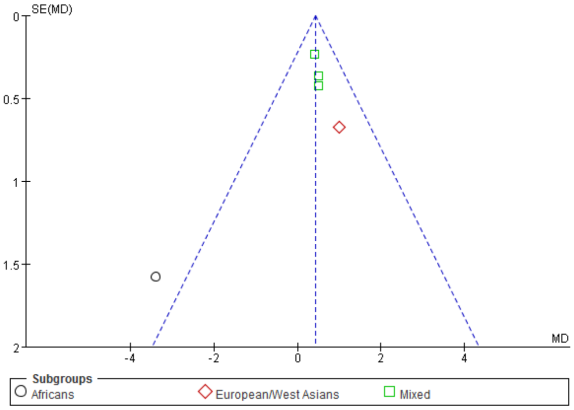

2.28 GDS15 of G2019S

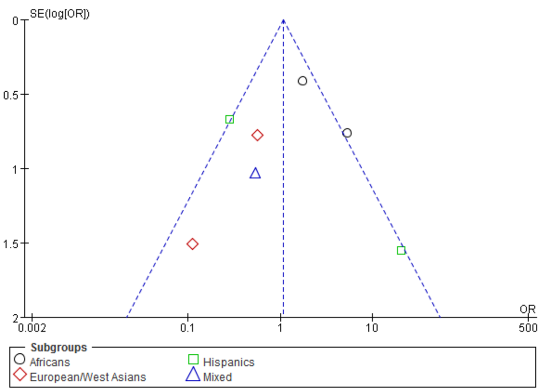

2.29 Hallucination of G2019S

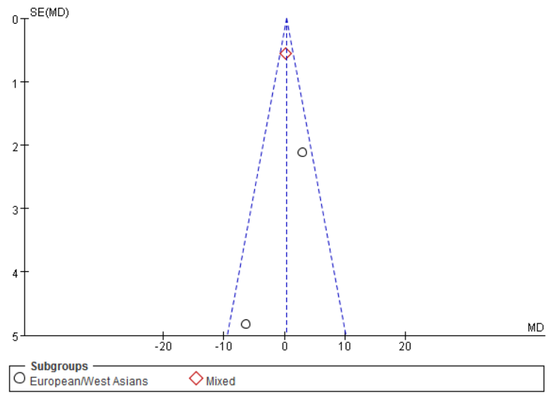

2.30 SCOPA-AUT of G2019S

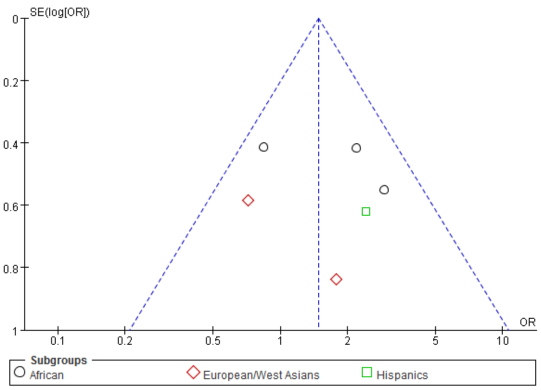

2.31 Cognitive impairments of G2019S

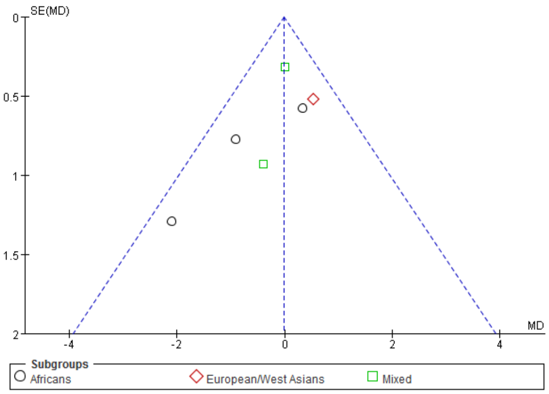

2.32 MMSE of G2019S

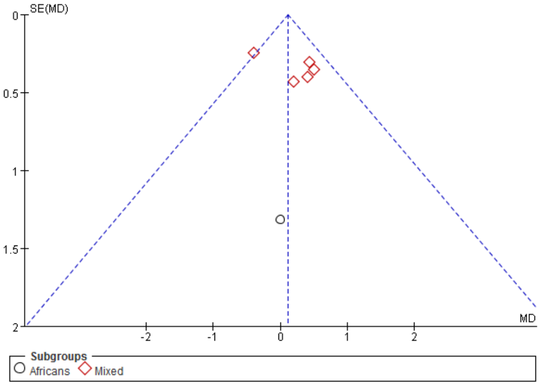

2.33 MoCA of G2019S

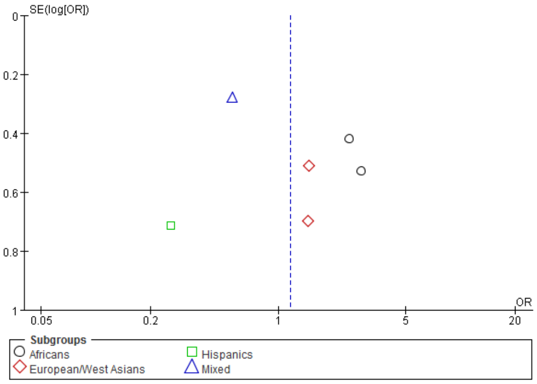

2.34 Sleep disturbances of G2019S

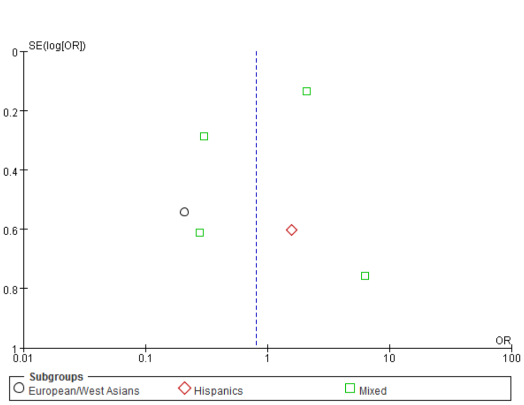

2.35 Olfactory disturbances of G2019S

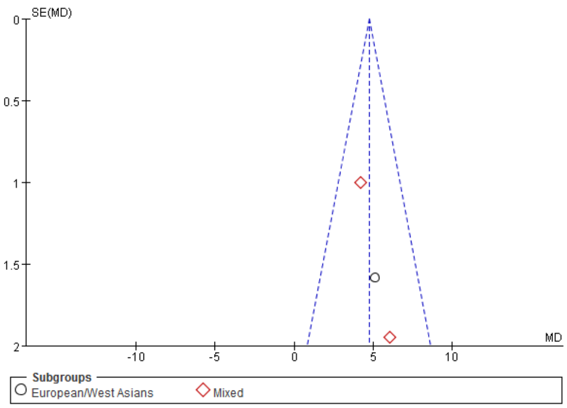

2.36 UPSIT scores of G2019S

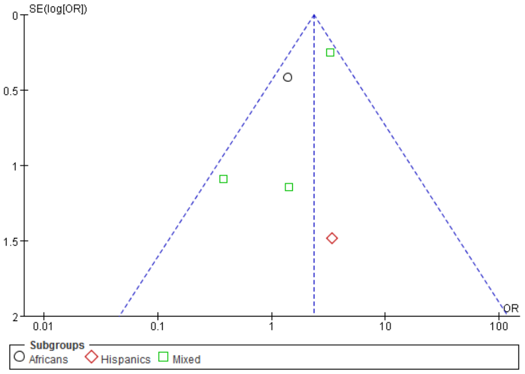

2.37 Good response to l-dopa of G2019S

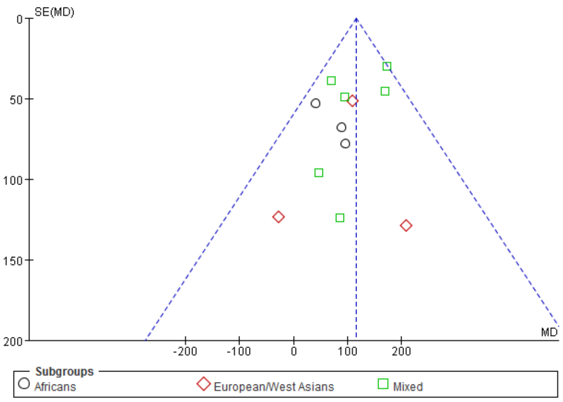

2.38 LEDD of G2019S

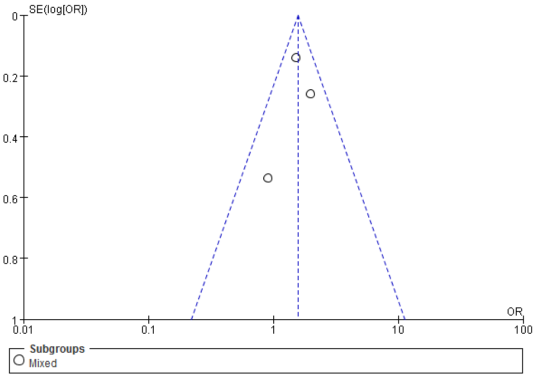

2.39 Smoke of G2019S

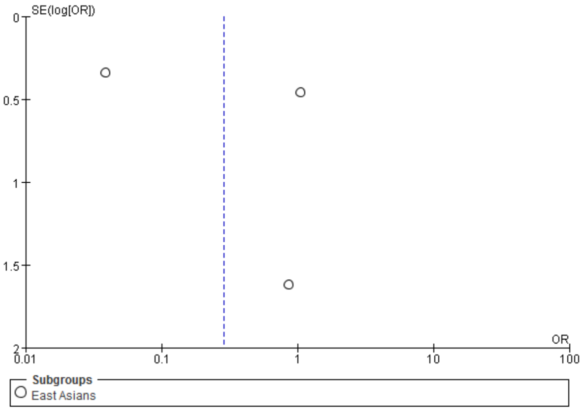

2.40 Asymmetrical onset of G2385R

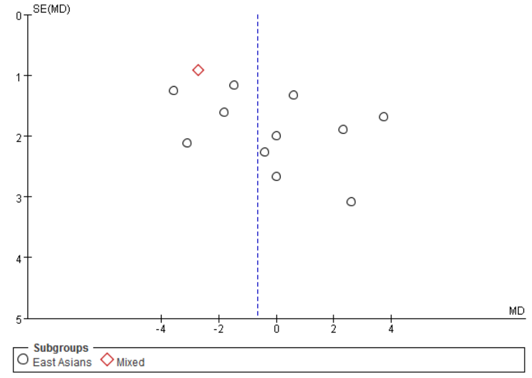

2.41 Age at onset of G2385R

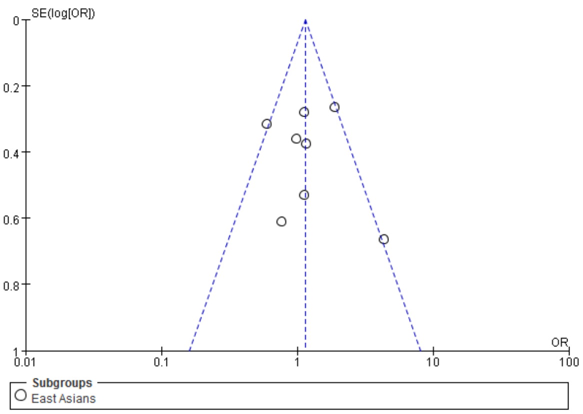

2.42 EOPD of G2385R

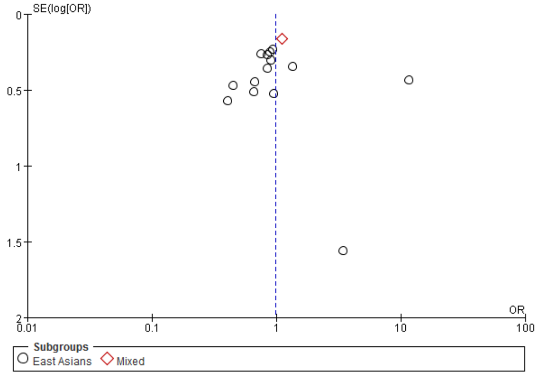

2.43 Gender-male of G2385R

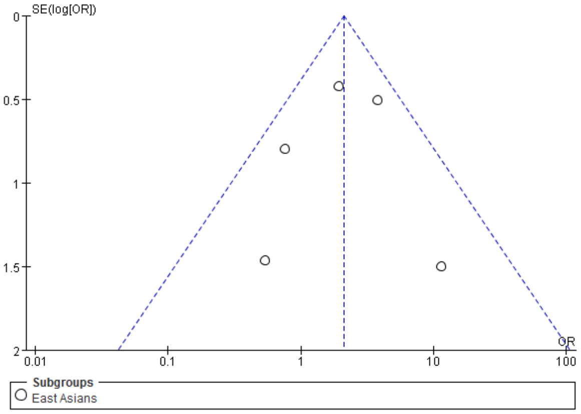

2.44 Family history of G2385R

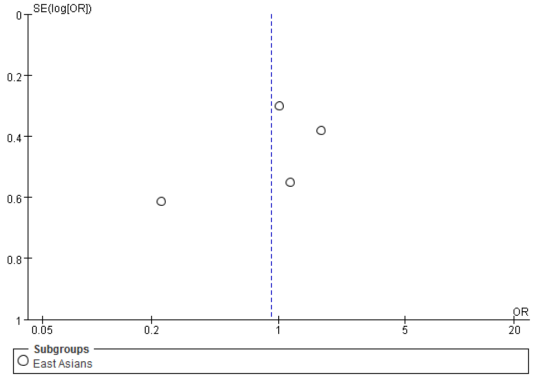

2.45 FS-Bradykinesia of G2385R

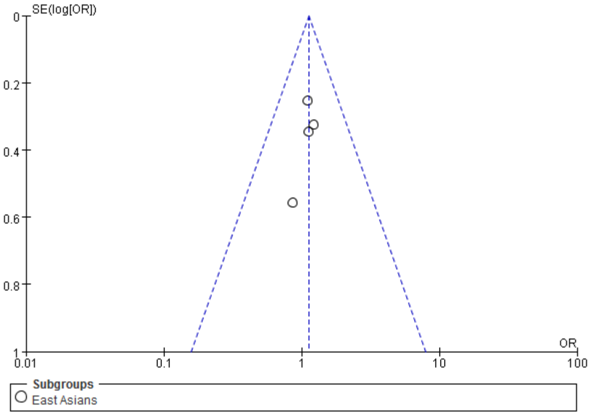

2.46 FS-Resting tremor of G2385R

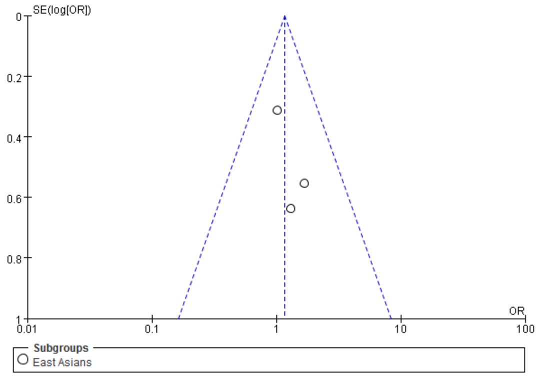

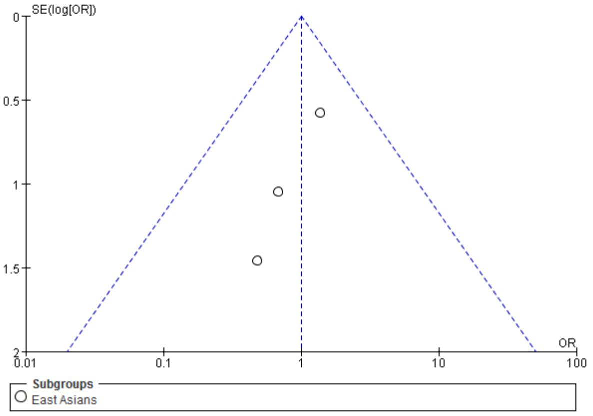

2.47 FS-Rigidity of G2385R

2.48 FS-Postural instability or Gait difficulty of G2385R

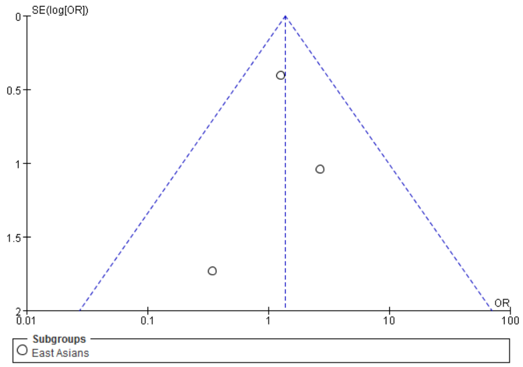

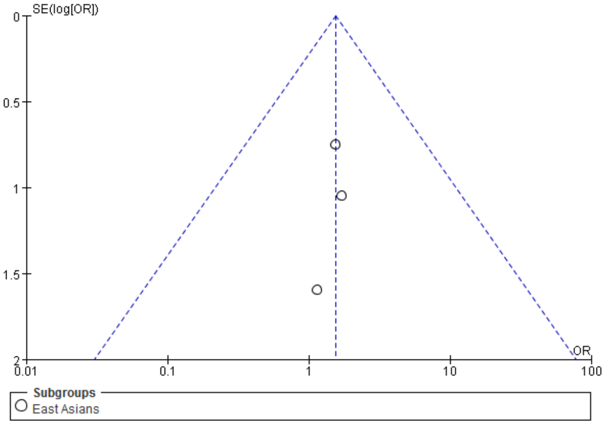

2.49 Resting tremor of G2385R

2.50 Rigidity of G2385R

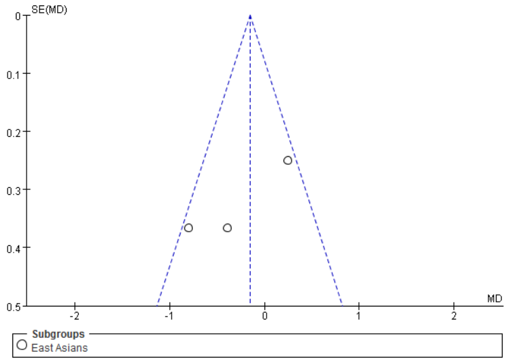

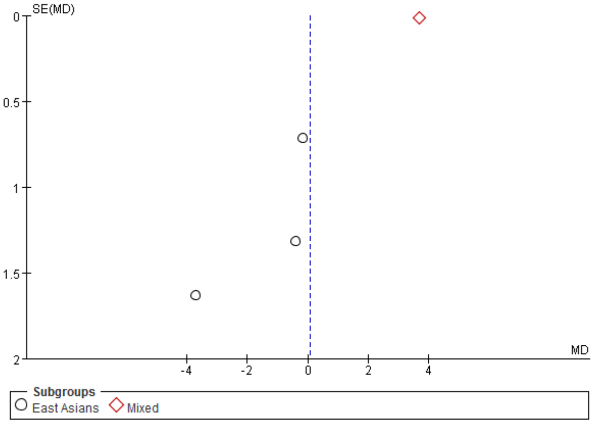

2.51 UPDRS Ⅰ of G2385R

2.52 UPDRS Ⅱ of G2385R

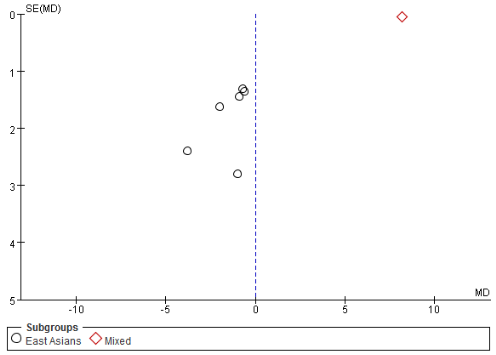

2.53 UPDRSⅢ of G2385R

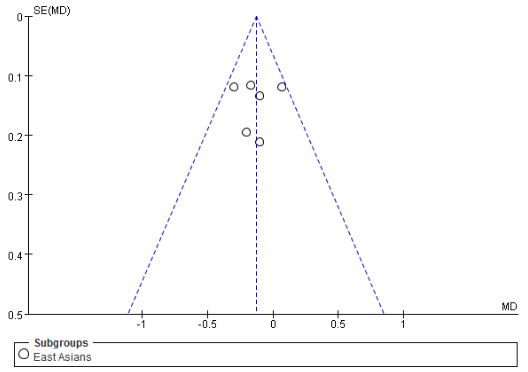

2.54 H-Y of G2385R

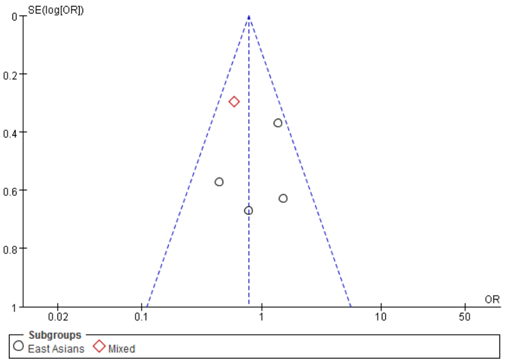

2.55 Dyskinesia of G2385R

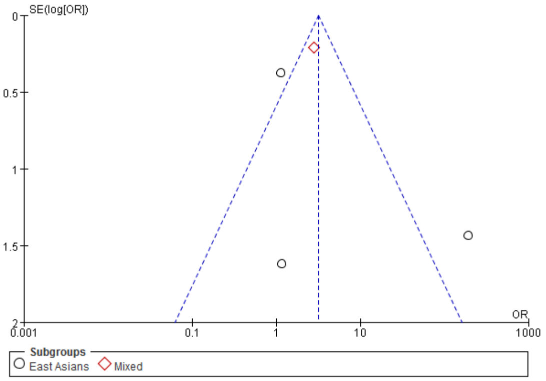

2.56 Motor fluctuations of G2385R

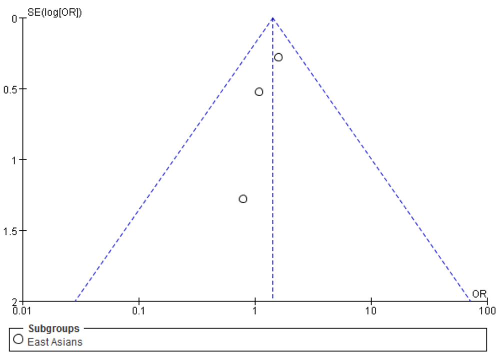

2.57 Depression of G2385R

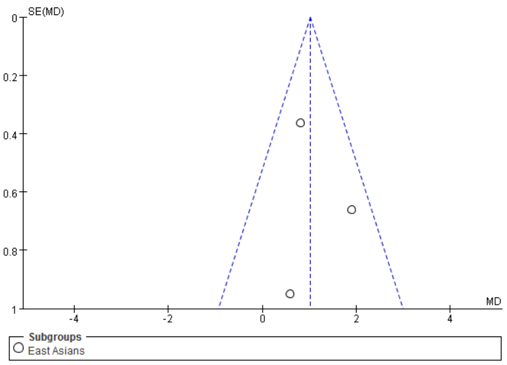

2.58 MMSE of G2385R

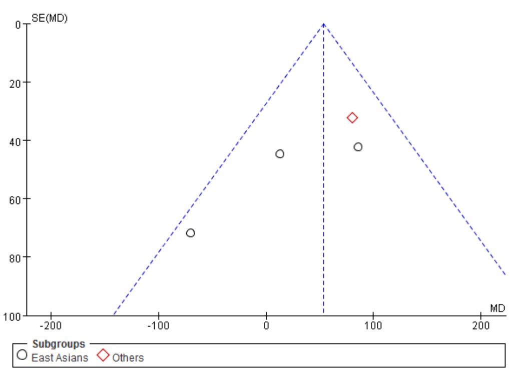

2.59 LEDD of G2385R

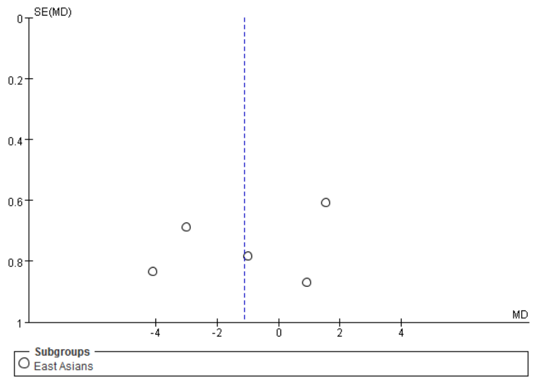

2.60 Age at onset of R1628P

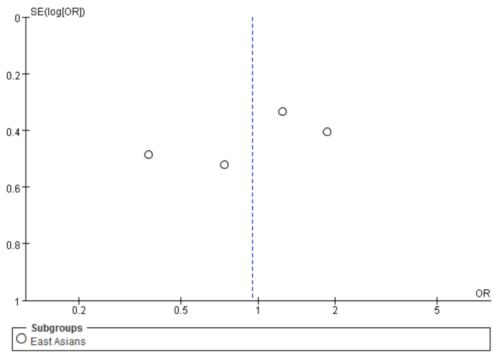

2.61 EOPD of R1628P

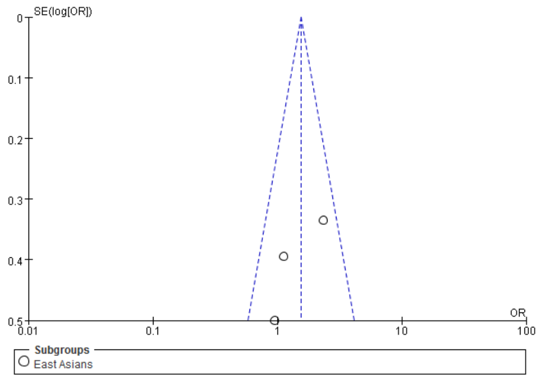

2.62 Gender-male of R1628P

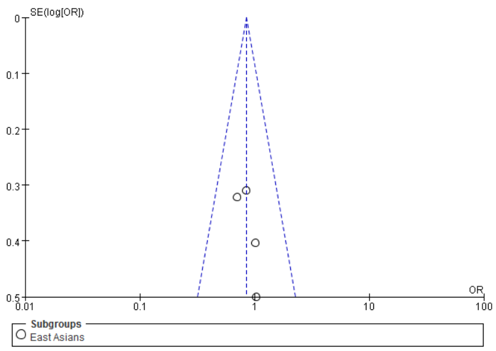

2.63 FS-Resting tremor of R1628P

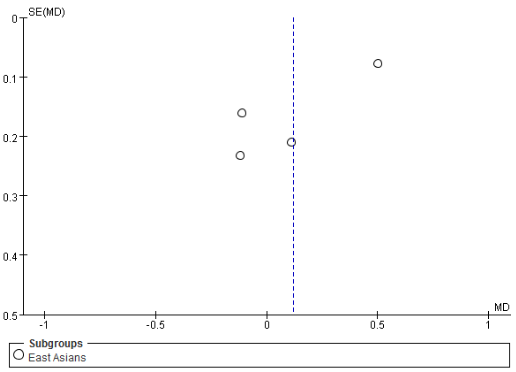

2.64 H-Y of R1628P

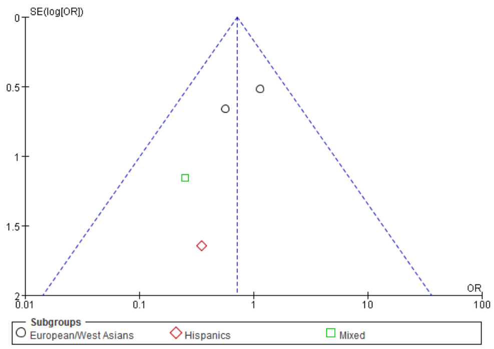

2.65 Gender-male of R1441G

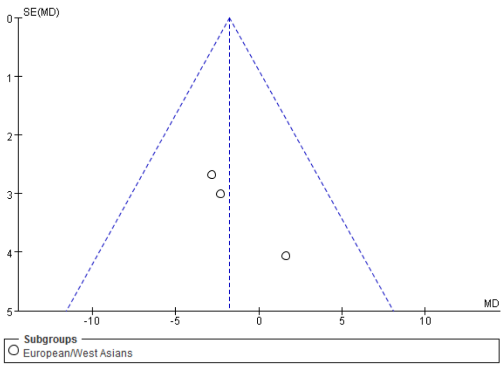

2.66 UPDRSⅢ of R1441G

Supplementary Figure 4. Funnel plots of the association between specific variants in *LRRK2* and PD clinical features in different ethnic groups. 2.1-2.39 reflected the pooled results of G2019S-related clinical features in different ethnic groups.2.40-2.59 reflected the pooled results of G2385R-related clinical features in different ethnic groups. 2.60-2.64 represented the pooled results of R1628P-related clinical features in different ethnic groups. 2.65-2.66 represented the pooled results of R1441G-related clinical features in different ethnic groups.
